# New Antiproliferative Compounds against Glioma Cells from the Marine-Sourced Fungus *Penicillium* sp. ZZ1750

**DOI:** 10.3390/md19090483

**Published:** 2021-08-26

**Authors:** Kuo Yong, Sidra Kaleem, Bin Wu, Zhizhen Zhang

**Affiliations:** Ocean College, Zhejiang University, Zhoushan Campus, Zhoushan 316021, China; yongkuo@zju.edu.cn (K.Y.); kaleemsidra85@yahoo.com (S.K.)

**Keywords:** marine fungus, *Penicillium* sp. ZZ1750, peniresorcinosides A–E, penidifarnesylin A, penipyridinone A, structure elucidation, antiglioma activity

## Abstract

Seven novel compounds, namely peniresorcinosides A–E (**1**–**5**), penidifarnesylin A (**6**), and penipyridinone A (**7**), together with the 11 known ones **8**–**17**, were isolated from a culture of the marine-associated fungus *Penicillium* sp. ZZ1750 in rice medium. The structures of the new compounds were established based on their high-resolution electrospray ionization mass spectroscopy (HRESIMS) data, extensive nuclear magnetic resonance (NMR) spectroscopic analyses, chemical degradation, Mosher’s method, ^13^C-NMR calculations, electronic circular dichroism (ECD) calculations, and single crystal X-ray diffraction. Peniresorcinosides A (**1**) and B (**2**) are rare glycosylated alkylresorcinols and exhibited potent antiglioma activity, with IC_50_ values of 4.0 and 5.6 µM for U87MG cells and 14.1 and 9.8 µM for U251 cells, respectively.

## 1. Introduction

Gliomas are one of the most common types of primary brain tumors and despite advances in cancer therapy, have remained particularly challenging to treat [1]. Gliomas usually locate at many important brain function areas, which makes surgical resection very difficult, therefore, chemotherapy has played a more important role in the treatment and prevention of glioma. However, most of the current anti-glioma drugs, such as temozolomide (the first line chemotherapeutic agent for glioma), carmustine, lomustine, and procarbazine, are DNA cytotoxic alkylating agents with drug resistance, serious toxicity, and other side-effects [2,3]. Although antibodies (such as bevacizumab) and molecularly targeted anticancer drugs are constantly being evaluated for the treatment of glioma, their overall curative effect is poor [4]. Therefore, the discovery and development of new anti-glioma drugs with unique mechanisms of action is an urgent need.

Marine fungi are important resources for the discovery of novel bioactive natural products and drug lead compounds [5,6,7,8,9,10,11]. Among them, *Penicillium* species fungi have been proved to be one of the most novel bioactive compound producers [9,10,11]. For example, 390 new compounds with different structural types were identified from the marine-derived *Penicillium* fungi during 1991–2014, of which 58% showed some form of antitumor, antiviral, antibacterial, and anti-inflammatory activity [9]. It was also reported that 188 secondary metabolites with diverse bioactivities were isolated from marine *Penicillium* fungi from 2015 to 2020 [11].

In recent years, we have carried out research on the discovery of natural products from marine microorganisms with antiproliferative activity against human glioma cells. These studies have resulted in the isolation and identification of a number of novel compounds with potent antiglioma activity, including the polycyclic anthraquinones *N*-acetyl-*N*-demethylmayamycin and streptoanthraquinone A from *Streptomyces* sp. 182SMLY [12], the cyclodepsipeptide streptodepsipeptides P11A and P11B from *Streptomyces* sp. P11-23B [13], bagremycin C from *Streptomyces* sp. Q22 [14], pyrrospirone G and penicipyrroether A from *Penicillium* sp. ZZ380 [15,16], 1-hydroxymethyl-8-hydroxyanthraquinone-3-carboxylic acid from *Streptomyces* sp. ZZ406 [17], streptoglutarimide H from *Streptomyces* sp. ZZ741 [18], and the compounds marinacarbolines G and M and caerulomycin N from *Actinoalloteichus* sp. ZZ1866 [19]. The antiglioma activity of some compounds were related to the downregulation of several important glioma glycolytic enzymes [13,17].

**Figure 1:**
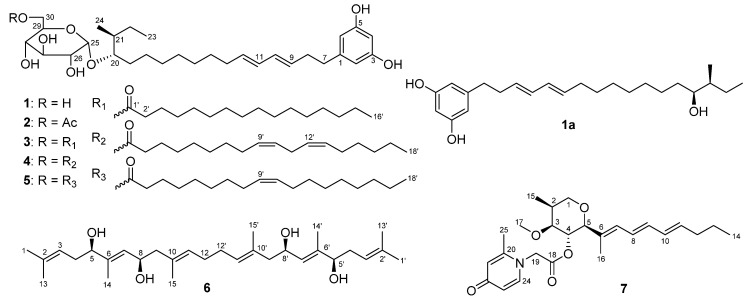
Structures of compounds **1**–**7** isolated from the marine fungus *Penicillium* sp. ZZ1750.

As a part of our ongoing research program to discover novel natural antiglioma products from marine microorganisms, a *Penicillium* sp. ZZ1750 fungus was isolated from a marine mud sample collected from the Arabian Sea, close to Karachi, Pakistan. An ethyl acetate (EtOAc) extract prepared from a scale-up culture of the strain ZZ1750 in rice medium showed antiproliferative activity against human glioma U87MG and U251 cells with inhibition rates of 79.68% and 78.27%, respectively. Chemical investigation of this active extract resulted in the isolation of 17 secondary metabolites, including seven new compounds (Figure 1), namely the peniresorcinosides A–E (compounds **1**–**5**), penidifarnesylin A (**6**), and penipyridinone A (**7**). Herein, we report the details of the isolation, structure elucidation and antiglioma activity evaluation of all isolated compounds.

## 2. Results and Discussion

The marine-sourced strain ZZ1750 (Figure S1, Supplementary Materials) was identified as *Penicillium* sp. ZZ1750 based on its internal transcribed spacer (ITS) rDNA sequence (563 bp, Figure S2), which was a 100% match to those of six other *Penicillium* strains (Table S1). An EtOAc extract prepared from a large-scale culture of the strain ZZ1750 in rich medium was separated by column chromatography, followed by high performance liquid chromatography (HPLC) purification, to afford compounds **1**–**17**.

**Figure 2:**
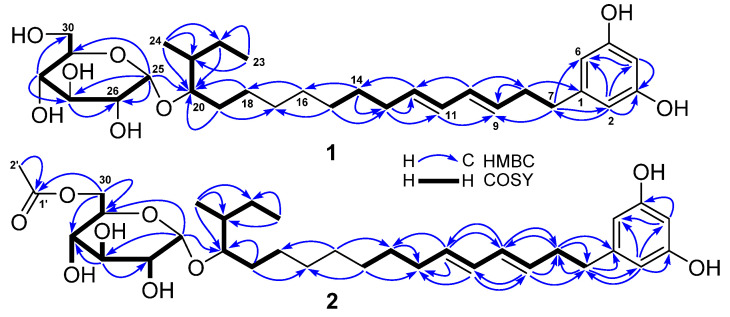
COSY and key HMBC correlations of peniresorcinosides A (**1**) and B (**2**).

Compound **1** was obtained as a yellowish oil with an optical rotation value of + 76 and a UV absorption at 232 nm. Its molecular formula C_30_H_48_O_8_ was deduced from the HRESIMS (high resolution electrospray ionization mass spectroscopy) ion peak at *m*/*z* 559.3248 [M + Na]^+^ (calcd. for C_30_H_48_NaO_8_^+^, 559.3247) as well as its ^13^C-NMR data. Analyses of its ^1^H, ^13^C, distortionless enhancement by polarization transfer (DEPT), and heteronuclear multiple quantum correlation (HMQC) spectra showed the presence of ten olefinic carbons, six oxymethines, one oxymethylene, one methine, ten methylenes, and two methyls (Table 1). A double peak signal at *δ*_H_ 6.02 (2H, d, *J* = 2.0 Hz, H-2 and H-6) showed HMQC correlation with a carbon signal at *δ*_C_ 106.3 (C-2 and C-6) and also showed HMBC (heteronuclear multiple bond correlation) correlation (Figure 2) with the same carbon signal (*δ*_C_ 106.3), indicating the presence of a symmetrical structure unit in **1**. 

Further analysis of HMBC correlations of H-2/6 (*δ*_H_ 6.02) with C-3/5 (*δ*_C_ 158.2), C-4 (*δ*_C_ 100.1), C-6/2 (*δ*_C_ 106.3), and C-7 (*δ*_C_ 35.3) as well as H-4 (*δ*_H_ 6.00, t, 2.0 Hz) with C-2, C-3, C-5, and C-6 demonstrated that the symmetrical structure unit was a resorcinol derivative with a substituent at the C-1 position. This substituent was made up of two hydroxyalkene and hexose moieties. The hydroxyalkene moiety at C-1 was identified as a octadecene with two pairs of double bonds at C_9-10_ and C_11-12_, an oxymethine at C-20, and a methyl at C-21 based on the two COSY (correlation spectroscopy) spin systems of H_2_-7/H_2_-8/H-9/H-10/H-11/H-12/H_2_-13/H_2_-14/H_2_-15 and H_2_-18/H_2_-19/H-20/H-21(H_3_-24)/H_2_-22/H_3_-23 as well as the key HMBC correlations (Figure 2) of H-2/6 with C-7, H_2_-7 with C-2, C-6, and C-9, H_2_-8 with C-1 and C-9, H-10 with C-8 and C-12, H-11 with C-9, H-12 with C-14, H_2_-13 with C-11 and C-12, H_2_-14 with C-13 and C-16, H_2_-15 with C-13 and C-17, H_2_-16 with C-18, H_2_-18 with C-20, H_2_-19 with C-17 and C-20, H_2_-22 with C-20, H_3_-23 with C-21 and C-22, H_3_-24 with C-20, C-21, and C-22. 

**Figure 3:**
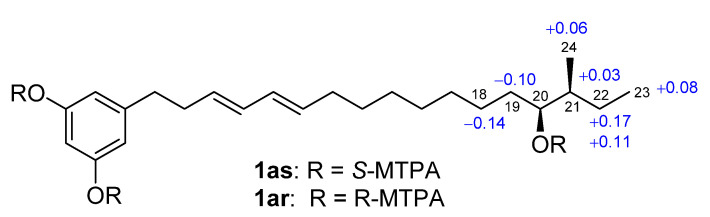
Δ*δ_S_*_-*R*_ values for the MTPA esters **1as** and **1ar** of compound **1a**.

The large coupling constants of ^3^*J*_H9-H10_ (15.3 Hz) and ^3^*J*_H11-H12_ (14.8 Hz) (Table 1) indicated 9*E* and 11*E* geometries. The hexose moiety resonated at *δ*_H_ 4.70 (1H, d, 3.7 Hz, H-25), 3.15 (1H, dd, 9.5, 3.7, H-26), 3.37 (1H, t, 9.5 Hz, H-27), 3.07 (1H, t, 9.5 Hz, H-28), 3.43 (1H, m, H-29), 3.53 (1H, dd, 11.5, 2.0 Hz, H-30a), 3.46 (1H, dd, 11.5, 4.7 Hz, H-30b), and *δ*_C_ 98.2 (CH, C-25), 72.1 (CH, C-26), 73.0 (CH, C-27), 70.1 (CH, C-28), 73.0 (CH, C-29), and 60.8 (CH_2_, C-30), which were assigned by HMQC, COSY and HMBC correlations. Enzymatic hydrolysis of **1** by *α*-glucosidase produced D-glucose and **1a**, a new compound. The aldonitrile acetate of the hydrolytic D-glucose was identified by gas chromatography (GC) analysis [20] (Figure S3) using the authentic aldonitrile acetates of different sugars (D-glucose, L-glucose, D-galactose, L-galactose) as references. A small coupling constant (3.5 Hz, ^3^*J*_H25-H26_) and a upfield ^13^C chemical shift (*δ*_C_ 98.3, C-25, when compared to *δ*_C_ 103.2 for the anomeric carbon of *β*-d-glucosyl moiety) [21] indicated an *α* anomeric configuration for the glucose, which was further supported by the fact that only *α*-glucosidase hydrolyzed this compound. Therefore, the hexose was proved to be an *α*-D-glucopyranosyl moiety, which was linked to C-20 as established by the HMBC correlations of H-20 (*δ*_H_ 3.35) with C-25 and H-25 with C-20 (*δ*_C_ 81.0). The planar structure of the aglycone **1a** was established based on its HRESIMS data as well as ^13^C, ^1^H, COSY, and HMQC spectroscopic analyses. The configuration at C-20 of **1a** was assigned by the Mosher ester NMR method. Treatment of **1a** with (*R*)-α-methoxy-α-(trifluoromethyl) phenylacetyl chloride (*R*-MTPA-Cl) or *S*-MTPA-Cl gave *S*-MTPA ester (**1as**) or *R*-MTPA ester (**1ar**). The ^1^H-NMR chemical shift differences (Δ*δ_S_*_-*R*_, Figure 3 and Table S2) between **1as** and **1ar** in negative values for H-18 and H-19 and positive values for H-21, H-22, H-23, and H-24 were observed, indicating a 20*S*-configuration for **1a**. 

In order to assign the configuration at C-21 and further support the 20*S*-configuration, four possible diastereomers of 20*R*,21*S*-**1a**, 20*S*,21*S*-**1a**, 20*R*,21*R*-**1a**, and 20*S*,21*R*-**1a** were subjected to ^13^C and ^1^H-NMR calculations [22,23]. The results (Tables S3–S5) showed that NMR data of **1a** was very close to those of the model molecule 20*S*,21*S*-**1a** with DP4^+^ probability scores of 99.90% for ^13^C-NMR data, 68.95% for ^1^H-NMR data, and 99.98% for all NMR data, determining a 20*S*,21*S*-configuration for **1a**. Based on the combined foregoing evidence, the structure of **1** was elucidated as a new glycosylated alkylresorcinol analogue, which was named peniresorcinoside A. The full assignment of its ^13^C- and ^1^H-NMR data (Table 1) was made by HMQC, COSY, and HMBC correlations (Figure 2).

Compound **2** was also obtained as a yellowish oil with a similar optical rotation value and UV absorption to those of **1**. The molecular formula C_32_H_50_O_9_ of **2** was deduced from its HRESIMS ion peak at *m*/*z* 601.3346 [M + Na]^+^, 42 mass units higher than that of **1**, corresponding to a -C_2_H_2_O group. The IR spectrum of **2** showed an absorption at 1724 cm^−1^, indicating the presence of a carbonyl group. Detailed comparison of the ^13^C- and ^1^H-NMR spectra of **2** and **1** indicated that their chemical shifts bore a very close resemblance, except for two additional ^13^C-NMR signals (*δ*_C_ 170.2 and 20.6) and one extra ^1^H-NMR signal (*δ*_H_ 1.98, 3H, s) for an acetyl observed in the NMR spectra of **2**. The HMBC correlations of H_2_-30 (*δ*_H_ 4.25, dd, *J* = 11.7, 1.9 Hz; 3.95, dd, *J* = 11.7, 7.3) with C-1′ (*δ*_C_ 170.2) and H_3_-2′ (*δ*_H_ 1.98) with C-1′ demonstrated the acetyl was at the C-30 position. The difference between the ^13^C-NMR chemical shifts for C-30 (Δ +3.2 ppm) between **2** and **1** also supported the location of this acetyl group. Compound **2** was thus elucidated as a new glycosylated alkylresorcinol analogue, named peniresorcinoside B. The ^13^C- and ^1^H-NMR data (Table 1) were fully assigned based on the HMQC, COSY, and HMBC correlations (Figure 2).

Compound **3** was obtained as a yellowish oil. Its HRESIMS spectrum showed ion peaks at *m*/*z* 775.5719 [M + H]^+^ and 797.5543 [M + Na]^+^, corresponding to a molecular formula C_46_H_78_O_9_, which was 16 carbons, 30 protons, and one oxygen more than that of **1**. Careful analyses of the ^13^C- and ^1^H-NMR spectra of **3** and **1** demonstrated that **3** and **1** shared the same glycosylated alkylresorcinol structural backbone. Compared to the NMR signals of **1**, compound **3** showed additional NMR signals for a carbonyl (*δ*_C_ 172.7), a methyl (*δ*_C_ 13.9; *δ*_H_ 0.84, 3H, t, *J* = 7.0 Hz), and some methylenes, whose NMR signals were high overlapped. Based on these characteristic NMR signals, in consideration of the presence of additional 16 carbons and one oxygen in **3**, it was deduced that compound **3** had a structural unit of sixteen-carbon saturated acid (palmitic acid). The HMBC correlations of H_2_-30 (*δ*_H_ 4.26, 1H, d, *J* = 10.8; 3.95, 1H, dd, *J* = 10.8, 7.3 Hz) with C-1′ (*δ*_C_ 172.7) established the linkage of this sixteen-carbon unit. Therefore, compound **3** was elucidated as a new glycosylated alkylresorcinol analogue, and named peniresorcinoside C. Its ^13^C- and ^1^H- NMR data (Table 2) were assigned based on the HMQC, COSY, and HMBC correlations (Figure S4). 

The HRESIMS spectrum of **4** gave ion peaks at *m*/*z* 799.5716 [M + H]^+^ and 821.5536 [M + Na]^+^, matching a molecular formula of C_48_H_78_O_9_, 18 carbons, 30 protons, and one oxygen more than that of **1**. Analyses of the ^13^C- and ^1^H-NMR spectra of **4** and **1** led to the conclusion that **4** also had the same glycosylated alkylresorcinol structural unit of **1**. Compared to the NMR signals of **1**, compound **4** exhibited additional NMR signals for a carbonyl (*δ*_C_ 172.7), two pairs of double bonds (*δ*_C_ 129.7, 129.6, 127.8, 127.7; *δ*_H_ 5.28, 2H, m, 5.31, 2H, m), a methyl (*δ*_C_ 13.9; *δ*_H_ 0.84, 3H, t, *J* = 7.3 Hz), and some signal-overlapped methylenes. These characteristic NMR signals, together with the fact of additional 18 carbons and one oxygen in **4** mentioned above, suggested that compound **4** had an eighteen-carbon unsaturated fatty acid structural unit. Alkaline hydrolysis of **4** gave peniresorcinoside A (**1**) as determined by co-HPLC analysis (Figure S5) with the authentic sample and 9(*Z*),12(*Z*)-octadecadienoic acid (linoleic acid, **4a**) based on its NMR data (Table S9) and the comparison with the reference data [24,25] as well as co-HPLC analysis (Figure S6) with standard linoleic acid. The HMBC correlations of H_2_-30 (*δ*_H_ 4.27, 1H, d, *J* = 11.2; 3.95, 1H, dd, *J* = 11.2, 7.5 Hz) with C-1′ (*δ*_C_ 172.7) demonstrated this linoleic acid unit at C-30 position. The structure of **4** was thus identified as a new glycosylated alkylresorcinol analogue, named peniresorcinoside D. Its ^13^C- and ^1^H-NMR data (Table 2) were assigned based on the HMQC, COSY, and HMBC correlations (Figure S4).

The molecular formula C_48_H_80_O_9_ of **5** was determined based on its HRESIMS ions at *m*/*z* 823.5694 [M + Na]^+^, two mass units higher than that of **4**. Detailed analyses of the ^13^C- and ^1^H-NMR spectra of **5** and **4** indicated that the structures of both compounds had the same glycosylated alkylresorcinol backbone with a difference in the number of double bonds of the eighteen-carbon unsaturated fatty acid unit. The ^13^C- and ^1^H-NMR spectra of **5** only exhibited a pair of double bonds (*δ*_C_ 129.7, 129.6; *δ*_H_ 5.29, 2H, m), instead of the two pairs of double bonds in **4**. 

Alkaline hydrolysis of **5** also gave peniresorcinoside A (**1**) as confirmed by co-HPLC analysis (Figure S5) and octadecenoic acid (oleic acid, **5a**), as determined by analyzing its ^13^C- and ^1^H-NMR data (Table S9), comparing with the reference data [26], and co-HPLC analysis (Figure S7) with an oleic acid standard. This oleic acid unit connected to C-30 was established through the HMBC correlations of H_2_-30 (*δ*_H_ 4.27, 1H, d, *J* = 11.2; 3.95, 1H, dd, *J* = 11.2, 7.5 Hz) with C-1′ (*δ*_C_ 172.7). The structure of **5** was elucidated as a new glycosylated alkylresorcinol analogue, named peniresorcinoside E. The ^13^C- and ^1^H-NMR data (Table 2) of **5** were assigned based on its HMQC, COSY, and HMBC correlations (Figure S4).

**Figure 4:**
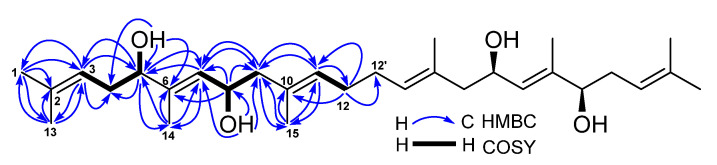
COSY and key HMBC correlations of penidifarnesylin A (**6**).

**Figure 5:**
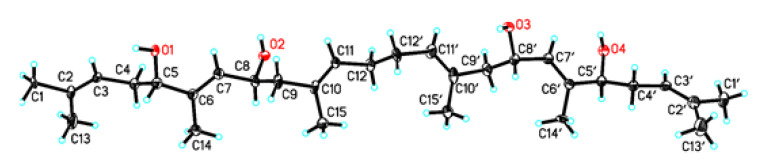
Crystal structure of penidifarnesylin A (**6**) (Cu Kα radiation, displacement ellipsoids are drawn at the 30% probability level).

**Figure 6:**
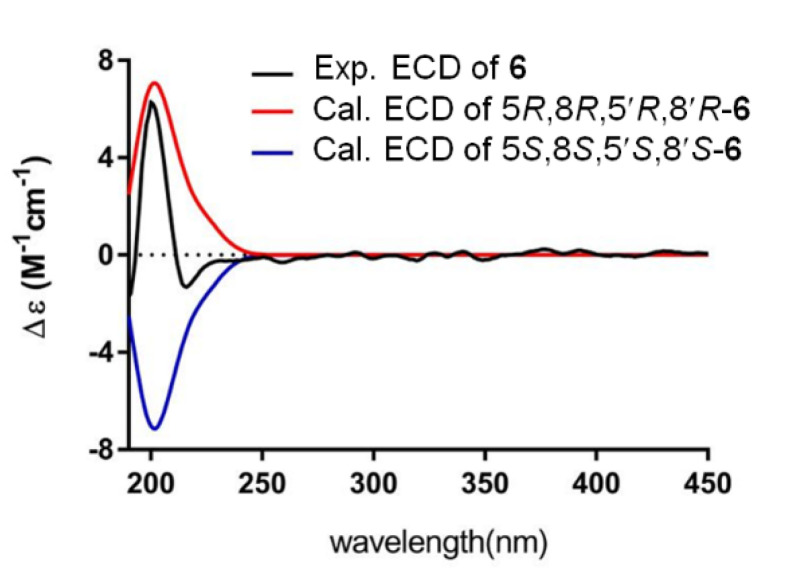
Experimental ECD spectrum of penidifarnesylin A (**6**, 190–450 nm) and calculated ECD spectrum of 5*R*,8*R*,5′*R*,8′*R*-**6** and 5*S*,8*S*,5′*S*,8′*S*-**6** at the b3lyp/6-311+g (d, p) level in MeOH.

Compound **6** was obtained as orthorhombic crystals from a mixture solvent of EtOAc and CHCl_3_ (1:1) and its HRESIMS ion peak at *m*/*z* 497.3604 [M + Na]^+^, corresponding to a molecular formula C_30_H_50_O_4_. However, only 15 carbon signals were observed in the ^13^C NMR spectrum, suggesting its symmetrical structure. The 15 carbon signals were assigned for six olefin carbons, two oxymethine carbons, three methylene carbons, and four methyl carbons (Table 3) based on its HMQC spectrum. The half planar structure (C_1_-C_15_) of **6** was established as a farnesyl derivative by further analyses of its COSY and HMBC correlations (Figure 4). A HMBC correlation of H_2_-12 (*δ*_H_ 1.92, 2H, m) with C-12′ (*δ*_C_ 27.9, CH_2_), in consideration of its molecular formula, suggested the whole planer structure of **6**, which was further confirmed by its crystal structure (Figure 5) obtained from X-ray diffraction analysis. However, the data from the X-ray diffraction was not good enough for the assignment of the absolute configuration of **6** because of a poor Flack parameter of 0.3 (3). Therefore, a computational method was applied to assign the absolute configuration of **6** by comparing its experimental electronic circular dichroism (ECD) spectrum with the calculated ECD spectra. The X-ray CIF profile of **6** (5*R*,8*R*,5′*R*,8′*R*-**6**) and its enantiomer (5*S*,8*S*,5′*S*,8′*S*-**6**) were initially optimized at B3LYP/6-31g (d, p) level in MeOH. The theoretical calculations of ECD were conducted in MeOH using Time-dependent Density functional theory (TD-DFT) at the B3LYP/6-311+g (d, p) level. The results (Figure 6) showed that the experimental ECD spectrum of **6** was agreement with the calculated ECD curve of the model molecule 5*R*,8*R*,5′*R*,8′*R*-**6**, indicating a 5*R*,8*R*,5′*R*,8′*R*-configuration for **6**. Based on the foregoing evidence, the structure of **6** was determined to correspond to a new dimeric farnesene derivative, named penidifarnesylin A. Its ^13^C and ^1^H NMR data (Table 3) were assigned according to the HMQC, COSY, and HMBC correlations (Figure 4).

**Figure 7:**
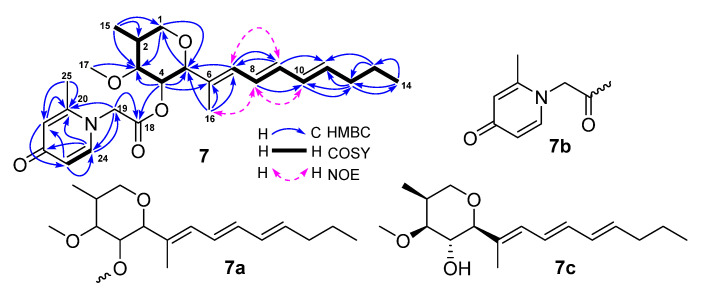
COSY, key HMBC, and NOE correlations of penipyridinone A (**7**).

The molecular formula of **7** was defined as C_25_H_35_NO_5_ based on its HRESIMS ion peaks at *m*/*z* 430.2587 [M + H]^+^ and 452.2406 [M + Na]. Analyses of its ^1^H-, ^13^C- and HMQC NMR spectra demonstrated the presence of two carbonyls, ten olefin carbons, eight olefin protons, three oxymethines, one oxymethylene, one methoxy, one methine, three methylenes, and four methyls (Table 4). The two carbonyls and ten olefin carbons (five pairs of double bonds) accounted for seven out of the nine degrees of unsaturation required by the molecular formula, suggesting that **7** had a structure with two rings. Two COSY spin systems of H_2_-1/H-2(H_3_-15)/H-3/H-4/H-5 and H-7/H-8/H-9/H-10/H-11/H_2_-12/H_2_-13/H_3_-14 and the HMBC correlations as described in Figure 7 determined that the first cycle-related partial structure (**7a**, Figure 7) was a tetrahydropyran derivative. Similarly, the second cycle-related partial structure (**7b**) was established as a pyridinone derivative by the COSY correlation of H-23 (*δ*_H_ 5.93, dd, 8.0, 2.8 Hz) with H-24 (*δ*_H_ 7.41, d, 8.0 Hz) and the HMBC correlations (Figure 7). A HMBC correlation of H-4 (*δ*_H_ 4.84, t, 9.5 Hz) with C-18 (*δ*_C_ 167.5) demonstrated the connection of the two partial structures of **7a** and **7b**.

The relative configurations of **7** were assigned based on the coupling constants and nuclear Overhauser effect (NOE) information. A small coupling constant of 5.5 Hz (^3^*J*_H2-__H3_) and the large coupling constants of 9.5 Hz (^3^*J*_H3-H4_ and ^3^*J*_H4-H5_) (Table 4) suggested a *β*-orientation for H-4 and an *α*-orientation for H-2, H-3, and H-5 [27]. The 6*E* geometry was assigned by the NOE correlations of H-7 (*δ*_H_ 5.97, d, 10.1 Hz) with H-9 (*δ*_H_ 6.24, dd, 15.0, 10.1 Hz) as well as H-8 (*δ*_H_ 6.29, dd, 15.0, 10.1 Hz) with H-10 (*δ*_H_ 6.13, dd, 15.6, 10.1 Hz) and H_3_-16 (*δ*_H_ 1.66, s), whereas the 8*E* and 10*E* geometries were determined based on their *trans*-coupling constants of 15.0 Hz for ^3^*J*_H8-H9_ and 15.6 Hz for ^3^*J*_H10-H11_. These assigned configurations for **7** were the same as those of restrictinol (**7c**) [28,29]. Based on the forgoing evidences, the structure of **7** was elucidated as a new restrictinol analogue with a unique pyridinone functionality, named penipyridinone A. Its ^13^C- and ^1^H-NMR data (Table 4) were assigned based on the HMQC, COSY, HMBC, and NOESY correlations (Figure 7).

Based on the NMR data and optical rotation values as well as the comparison with the reported data, compounds **8**–**17** were identified as known compounds methyl linoleate (**8**) [30], 12*β*-hydroxyverruculogen TR-2 (**9**) [31,32], 12*β*-hydroxy-13*α*-methoxyverruculogen TR-2 (**10**) [31,33], cyclotryprostatin B (**11**) [34], verruculogen (**12**) [34], fumiquinazoline C (**13**) [35], fumiquinazoline J (**14**) [35,36], brevianamide F (**15**) [37,38], 2-[(2*R*-hydroxypropanoyl)amino] benzamide (**16**) [39], and trypacidin (**17**) [40,41]. The ^13^C and ^1^H NMR data of compounds **8**–**17** are reported in Tables S9–S13. 

All isolated compounds **1**–1**7** were tested for their antiproliferative activity against human glioma U87MG and U251 cells by using the Sulforhodamine B (SRB) assay [42]. Doxorubicin (DOX, an anticancer drug) was used as a positive control. The results (Table 5) showed that peniresorcinosides A (**1**) and B (**2**) had potent antiglioma activity with IC_50_ values of 4.0 and 5.6 µM for U87MG cells and 14.1 and 9.8 µM for U251 cells, respectively. Penidifarnesylin A (**6**) also showed antiproliferative activity with IC_50_ values of 5.9 µM against U87MG cells and 27.6 µM against U251 cells. However, peniresorcinosides C–E (**3**–**5**) with a long-chain fatty acid unit only showed antiproliferative activity against U87MG cells with IC_50_ values of 53.0, 19.4, and 22.1 µM, respectively. Other tested compounds were inactive at a concentration of 50 µM.

Marine-derived *Penicillium* fungi are still important sources for the discovery of novel bioactive natural products. The current study described the isolation and structural elucidation of 17 metabolites, including new glycosylated alkylresorcinols of peniresorcinosides A–E (**1**–**5**), penidifarnesylin A (**6**), and penipyridinone A (**7**) as well as some known indole alkaloids with complicated structures, from the marine fungus *Penicillium* sp. ZZ1750 cultured in rice medium.

Alkylresorcinols are resorcinol units alkylated with a long odd-numbered carbon chain and had high levels (over 500 µg/g) in wheat, rye, and triticale [43]. It has been reported that some of alkylresorcinols exhibited antimicrobial, antiparasitic, and cytotoxic activities [44]. Resorcinosides A and B [45], recently isolated from the marine fungus *Penicillium janthinellum*, are the first reported alkylresorcinol derivatives containing a glucose moiety linked to a hydroxy group of the alkyl side chain. Peniresorcinosides A–E (**1–5**) are second example of this type of rare glycosylated alkylresorcinols. Peniresorcinosides C–E (**3**–**5**) have more complicated structures, with a long-chain fatty acid moiety attached to the C-6 position of the glucosyl unit. Evaluation of the antiglioma activity showed that peniresorcinosides A (**1**) and B (**2**) had potent antiproliferative activity against both glioma U87MG and U251 cells, while peniresorcinosides C–E (**3**–**5**) only exhibited moderate antiglioma activity against U87MG cells.

The cyclotryprostatin [34,46], fumiquinazoline [35,46,47,48], and verruculogen [31,34] indole alkaloids were most frequently isolated from *Aspergillus* and *Penicillium* fungi. Some of them have reported antibacterial, antifungal, cytotoxic, and antidiabetic activities [48,49]. In this study, seven such known indole alkaloids (compounds **9**–**15**) were obtained from the metabolites produced by the marine-sourced *Penicillium* fungus ZZ1750. Fumiquinazoline C (**13**) was previously reported to have moderate cytotoxicity against murine lymphocytic leukemia P388 cells [47]. However, none of these isolated indole alkaloids **9**–**15** were active against human glioma U87MG and U251 cells at a concentration of 50 µM.

## 3. Materials and Methods

### 3.1. General Experimental Procedures

Melting points (m.p.) were measured with a WRX-4 microscope apparatus (Shanghai Yice Apparatus & Equipment Co. Ltd., Shanghai, China) and was uncorrected. Ultraviolet (UV), optical rotation (OR), electronic circular dichroism (ECD), and infrared radiation (IR) spectra were measured on a METASH UV-8000 (Shanghai METASH Instruments Co. Ltd., Shanghai, China), an Autopol I Automatic polarimeter (Rudolph Research Analytical, Hackettstown, NJ, USA), a J-815 spectropolarimeter (JASCO Co. Tokyo, Japan), and a Nicolet^TM^ IS^TM^ 10 FT-IR spectrometer (Thermo Fisher Scientific, Waltham, MA, USA), respectively. NMR data were acquired on a JEOL 600 spectrometer (JEOL, Tokyo, Japan) using a standard JEOL pulse sequences for 1D and 2D (HMQC, COSY, HMBC, and NOESY) NMR experiments and chemical shifts were expressed in *δ* (ppm) relative to DMSO-*d*_6_ (*δ*_C_ 39.5, *δ*_H_ 2.50) or MeOH-*d*_4_ (*δ*_C_ 49.0, *δ*_H_ 3.31). HRESIMS data were obtained on a 6230 Time of Flight Liquid Chromatography/Mass Spectrometry (TOF LC/MS) spectrometer (Agilent Technologies, Palo Alto, CA, USA). X-ray diffraction analysis was carried out on an Xcalibur Atlas Gemini Ultra diffractometer (Agilent Technologies) with Cu Kα radiation (λ = 1.54184 Å) at 100 K. Silica gel (200–300 mesh, Qingdao Ocean Chemical Plant, Qingdao, China) and octadecyl-functionalized silica gel (ODS, Cosmosil 75C_18_-Prep, Nacalai Tesque Inc., Kyoto, Japan) were used for column chromatography. HPLC separation was performed on an Agilent 1260 HPLC system using Agilent Zorbax SB-C_18_ column (250 × 9.2 mm, 5 µm) or a CXTH LC-3000 preparative HPLC system (Beijing Chuangxin Tongheng Science & Technology Co. Ltd., Beijing, China) using a CT-30 column (Fuji-C_18_, 280 × 30 mm, 10 µm). GC analysis was conducted on a GC-2010 system (Shimadzu, Kyoto, Japan) equipped with an SH-Rtx-5 capillary column (30 m × 0.32 mm, 0.25 µm). Nitrogen gas was used as the carrier gas and flame ionization detection (FID) was used as detector. Both injection port and detector temperatures were fixed at 280 °C. The column temperature was set at 180 to 280 °C in 10 min with an increase of 10 °C/min. Solvents used for this study were purchased from the Sinopharm Chemical Reagent Co. Ltd. (Shanghai, China). Mosher’s reagents (*R*)-(–)-α-methoxy-α-(trifluoromethyl) phenylacetyl chloride (*R*-MTPA-Cl) and *S*-MTPA-Cl were ordered from Aladdin Industrial Corporation (Shanghai, China). Human glioma U87MG (JDS-2568) and U251 (XB-0439) cells were ordered from the Cell Bank of the Chinese Academy of Sciences, Shanghai, China). Linoleic acid (>95.0%) and oleic acid (>99.0%) were purchased from Shanghai Aladdin Biochemical Technology Co., Ltd, and doxorubicin (DOX, >98.0%) from Sigma-Aldrich (St. Louis, MO, USA). Different culture media were made in the laboratory, including Gauze’s synthetic medium (B, soluble starch 20 g, KNO_3_ 1 g, MgSO_4_⋅7H_2_O 0.5 g, NaCl 0.5 g, K_2_HPO_4_ 0.5 g, FeSO_4_ 0.01 g, agar 20 g, water 1 L), Gauze’s synthetic medium with sea salt (BS, soluble starch 20 g, KNO_3_ 1 g, MgSO_4_⋅7H_2_O 0.5 g, NaCl 0.5 g, K_2_HPO_4_ 0.5 g, FeSO_4_ 0.01 g, agar 20 g, sea salt 35 g, water 1 L), potato dextrose agar medium (PDA, potatoes 200 g, glucose 20 g, agar 20 g, boiled into 1 L of water for 15 min), potato dextrose agar medium with sea salt (PDAS, potatoes 200 g, glucose 20 g, agar 20 g, sea salt 35 g, boiled into 1 L of water for 15 min), William’s E medium (E, yeast 1.0 g, tryptone 5.0 g, FeCl_3_⋅6H_2_O 0.17 g, KH_2_PO_4_ 0.12 g, agar 30 g, water 1 L), William’s E medium with sea salt (ES, yeast 1.0 g, tryptone 5.0 g, FeCl_3_⋅6H_2_O 0.17 g, KH_2_PO_4_ 0.12 g, agar 30 g, sea salt 35 g, water 1 L), international *Streptomyces* project medium-2 (ISP-2, yeast extract 4 g, malt extract 10 g, dextrose 4.0 g, peptone 5 g, agar 20 g, water 1 L), international *Streptomyces* project medium-2 with sea salt (ISP-2S, yeast extract 4 g, malt extract 10 g, dextrose 4.0 g, peptone 5 g, agar 20 g, sea salt, 35 g, water 1 L), international *Streptomyces* project medium-4 (ISP-4, soluble starch 10 g, K_2_HPO_4_ 1 g, MgSO_4_⋅7H_2_O 1 g, NaCl 1 g, (NH_4_)_2_SO_4_ 2 g, CaCO_3_ 2 g, FeSO_4_ 1 mg, MnCl_2_ 1 mg, ZnSO_4_ 1 mg, agar 20 g, water 1 L), international *Streptomyces* project medium-4 with sea salt (ISP-4S, soluble starch 10 g, K_2_HPO_4_ 1 g, MgSO_4_⋅7H_2_O 1 g, NaCl 1 g, (NH_4_)_2_SO_4_ 2 g, CaCO_3_ 2 g, FeSO_4_ 1 mg, MnCl_2_ 1 mg, ZnSO_4_ 1 mg, agar 20 g, sea salt, 35 g, water 1 L).

### 3.2. Isolation and Taxonomic Identification of Strain ZZ1750

The strain ZZ1750 was isolated from a sample of marine mud, which was collected from the Arabian Sea near Karachi, Sindh, Pakistan in January 2019. Briefly, the mud sample was air dried at 28 °C for 7 days and the dried sample (1.0 g) was diluted with sterile water to make up the dilutions of 10^−2^, 10^−3^, and 10^−4^ g/mL. Each dilution (200 µL) was transferred on the surface of ten different solid media of B, BS, PDA, PDAS, E, ES, ISP-2, ISP-2S, ISP-4, and ISP-4S on Petri dishes and then incubated at 28 °C for 14 days. The single colony of ZZ1750 was picked from the 10^−2^ g/mL suspension in PDA medium and then transferred to another PDA medium in dish. After growth for another 7 days at 28 °C, the single pure colony (ZZ1750) that grew well was transferred onto PDA slant medium and stored at 4 °C for further study.

The ITS rDNA sequence analysis of strain ZZ1750 was conducted by Legenomics (Hangzhou Lizhen Biotechnology Co., Ltd., Hangzhou, China). The ITS rDNA sequence was compared to those in the GenBank database using the nucleotide Basic Local Alignment Search Tool (BLAST). The ITS rDNA sequence of strain ZZ1750 has been deposited in GenBank (accession number: MT159428). The strain *Penicillium* sp. ZZ1750 was preserved at the Laboratory of Institute of Marine Biology and Pharmacology, Ocean College, Zhoushan campus, Zhejiang University, Zhoushan, China.

### 3.3. Mass Culture of Strain ZZ1750

The colony of strain ZZ1750 from the PDA slant medium was inoculated into a 500 mL Erlenmeyer flask, which contained 250 mL potato dextrose broth (PDB) medium and then incubated for 3 days in a shaker (180 rpm, 28 °C) to produce the seed broth (Figure S1). The seed broth (5 mL) was transferred into rice medium (40 g rice, 60 mL 3.5% sea salt solution) in 500 mL Erlenmeyer flask and then all these flasks were incubated at 28 °C for 30 days in a static state. For this study, a total of 210 cultured flasks were prepared.

### 3.4. Extraction and Isolation of Compounds **1**–**17**

The culture of strain ZZ1750 in rice medium in each flask was extracted with EtOAc (250 mL) three times. The combined EtOAc extract was dried in vacuo to give an extract (100 g). This extract was fractionated on a column (160 × 10 cm) of silica gel (1600 g) eluting with a mixture of cyclohexane and EtOAc in different ratios (10:1, 5:1, 2:1, 1:1, 1:2, each 1000 mL) to give four fractions of Frs. A–D based on the results of TLC analyses.

Fr. A was separated by using an Agilent 1260 HPLC system equipped with an Agilent Zorbax SB-C_18_ column (250 × 9.4 mm, 5 µm; mobile phase: ACN/H_2_O, 62/38; flow rate: 1.0 mL/min; UV detection: 232 nm) to give **6** (15.0 mg, t_R_ 29.6 min) and **1** (15.2 mg, t_R_ 34.5 min).

Fr. B was first separated on an ODS (200 g) column (530 × 35 mm) eluting with 60%, 70%, 80%, and 90% MeOH (each 1000 mL) to yield four subfractions (SFrs. B_1_–B_4_) according to the TLC analytic results. SFr. B_1_ was further separated on a Fuji-C_18_ CT-30 column (280 × 30 mm, 10 µm; mobile phase: MeOH/H_2_O, 75/25; flow rate: 10 mL/min; UV detection: 210 nm) to give **2** (4.0 mg, t_R_ 34.5 min). SFr.B_2_ was also separated on the same CT-30 column using the same flow rate and same UV detection, but a different mobile phase of MeOH/H_2_O (70/30), to give **10** (2.5 mg; t_R_ 24.0 min) and **12** (12.2. mg; t_R_ 37.5 min). SFr.B_4_ was separated on the Zorbax SB-C_18_ column (mobile phase: MeOH/H_2_O, 95/5; flow rate: 1.0 mL/min, UV detection: 232 nm) to furnish **4** (2.8 mg, t_R_ 58.8 min), **3** (2.6 mg, t_R_ 72.0 min), and **5** (3.3 mg, t_R_ 76.8 min). Compound **8** (3.2 mg, t_R_ 31.1 min) was obtained from SFr.B_5_ through purification on the Zorbax SB-C_18_ column (mobile phase: MeOH/H_2_O, 98/2; flow rate: 1.0 mL/min, UV detection: 210 nm).

In the same way, SFr. B_3_ was further separated on the CT-30 column (mobile phase: MeOH/H_2_O, 67/33; flow rate: 10 mL/min; UV detection: 210 nm) to give parts B_3a_–B_3c_. By using the Zorbax SB-C_18_ column (flow rate: 1.0 mL/min), compounds **9** (20 mg, t_R_ 36.4 min, ACN/H_2_O, 55/45), **11** (1.5 mg; t_R_ 40.4 min, ACN/H_2_O, 50/50; UV detection: 210 nm), and **7** (3.8 mg, t_R_ 41 min, MeOH/H_2_O, 87/13; UV detection of 275 nm) were purified from B_3a_, B_3b_, and B_3c_, respectively.

Similarly, Fr. C was fractionated on the CT-30 column (mobile phase: MeOH/H_2_O, 60/40; flow rate: 10 mL/min; UV detection: 210 nm) to afford parts C_1_ and C_2_. Then, by using the Zorbax SB-C_18_ column (flow rate: 1.0 mL/min; UV detection: 210 nm), compound **17** (8.0 mg, t_R_ 25.0 min, mobile phase: MeOH/H_2_O, 65/35) was purified from part C_1_, **13** (21.0 mg, t_R_ 30.0 min) and **14** (4.7 mg, t_R_ 28.0 min, mobile phase: MeOH/H_2_O, 63/35) were obtained from part C_2_.

Finally, Fr. D was separated by using the Zorbax SB-C_18_ column (mobile phase: ACN/H_2_O, 40/60; flow rate: 1.0 mL/min; UV detection: 232 nm) to give **15** (2.8 mg, t_R_ 26.6 min) and **16** (2.1 mg, t_R_ 36 min). 

Peniresorcinoside A (**1**): Yellowish oil; molecular formula C_30_H_48_O_8_; [*α*]^20^_D_ +76° (*c* 0.10, MeOH); UV (MeOH) λ_max_ (log ε) 232 (3.45) nm; IR (ATR) ν_max_ 3354, 2912, 2843, 1593, 1452, 1155, 1020, 982, 835 cm^−1^; ^13^C-NMR (150 MHz) and ^1^H-NMR (600 MHz) data (in DMSO-*d*_6_), see Table 1; HRESIMS *m*/*z* 559.3248 [M + Na]^+^ (calcd for C_30_H_48_NaO_8_^+^, 559.3247).

Peniresorcinoside B (**2**): Yellowish oil; molecular formula C_32_H_50_O_9_; [*α*]^20^_D_ +80° (*c* 0.10, MeOH); UV (MeOH) λ_max_ (log ε) 232 (3.46) nm; IR (ATR) ν_max_ 3398, 2912, 1724, 1606, 1450, 1146, 1034, 609 cm^−1^; ^13^C-NMR (150 MHz) and ^1^H-NMR (600 MHz) data (in DMSO-*d*_6_), see Table 1; HRESIMS *m*/*z* 601.3346 [M + Na]^+^ (calcd for C_32_H_50_NaO_9_^+^, 601.3353).

Peniresorcinoside C (**3**): White amorphous powder; molecular formula C_46_H_78_O_9_; [α]^20^_D_ +28° (c 0.1, CH_3_OH); UV (MeOH) λ_max_ (log ε) 233 (3.99); IR (ATR) ν_max_ 3333, 2922, 2855, 1714, 1600, 1510, 1455, 1076, 1018, 841 cm^-1^; ^13^C-NMR (150 MHz) and ^1^H-NMR (600 MHz) data (in DMSO-*d*_6_), see Table 2; HRESIMS *m*/*z* 775.5719 [M + H]^+^ (calcd for C_46_H_79_O_9_^+^, 775.5724) and 797.5543 [M + Na]^+^ (calcd for C_46_H_78_NaO_9_^+^, 797.5544).

Peniresorcinoside D (**4**): White amorphous powder; molecular formula C_48_H_78_O_9_; [α]^20^_D_ +42° (c 0.1, CH_3_OH); UV (MeOH) λ_max_ (log ε) 233 (4.34); IR (ATR) ν_max_ 3355, 2924, 2857, 1722, 1592, 1457, 1145, 1104, 1016, 986, 840 cm^-1^; ^13^C-NMR (150 MHz) and ^1^H-NMR (600 MHz) data (in DMSO-*d*_6_), see Table 2; HRESIMS *m*/*z* 799.5716 [M + H]^+^ (calcd for C_48_H_79_O_9_^+^, 799.5724) and 821.5536 [M + Na]^+^ (calcd for C_48_H_78_NaO_9_^+^, 821.5544).

Peniresorcinoside E (**5**): White amorphous powder; molecular formula C_48_H_80_O_9_; [α]^20^_D_ +40° (c 0.1, CH_3_OH); UV (MeOH) λ_max_ (log ε) 233 (4.22); IR (ATR) ν_max_ 3343, 2922, 2855, 1716, 1598, 1459, 1149, 1059, 984, 841 cm^-1^; ^13^C-NMR (150 MHz) and ^1^H-NMR (600 MHz) data (in DMSO-*d*_6_), see Table 2; HRESIMS *m*/*z* 823.5694 [M + Na]^+^ (calcd for C_48_H_80_NaO_9_^+^, 823.5700).

Penidifarnesylin A (**6**): Orthorhombic crystals (EtOAc:CHCl_3_, 1: 1); molecular formula C_30_H_50_O_4;_ m.p. 145–148 °C; [*α*]^20^_D_ +40° (*c* 0.10, MeOH); ECD (200 µg/mL, MeOH) λ_max_ (Δε) 210 (+7.62) nm; UV (MeOH) λ_max_ (log ε) 210 (4.24) nm; IR (ATR) ν_max_ 3347, 2921, 2853, 1440, 1376, 1260, 1020 cm^−1^; ^13^C-NMR (150 MHz) and ^1^H-NMR (600 MHz) data (in DMSO-*d*_6_), see Table 3; HRESIMS *m*/*z* 497.3604 [M + Na]^+^ (calcd for C_30_H_50_NaO_4_^+^, 497.3607). Crystal data of penidifarnesylin A (**6**): C_30_H_50_O_4_ (*M* = 474.70 g/mol), orthorhombic, space group P2_1_2_1_2 (no. 18), *a* = 7.4649(8) Å, *b* = 59.512(10) Å, *c* = 9.9356(16) Å, *V* = 4413.9(12) Å^3^, *Z* = 6, *T* = 100.00(12) K, μ(Cu Kα) = 0.536 mm^-1^, *Dcalc* = 1.072 g/cm^3^, 29803 reflections measured (5.94° ≤ 2Θ ≤ 148.752°), 8796 unique (*R*_int_ = 0.1650, R_sigma_ = 0.1369), which were used in all calculations. The final *R*_1_ was 0.0877 (I > 2σ (I)) and *wR*_2_ was 0.2366 (all data). The crystal data and structure refinement parameters of penidifarnesylin A (**6**) were also reported in Table S6. Crystallographic data of penidifarnesylin A (**6**) has been deposited at the Cambridge Crystallographic Data Centre (CCDC Number: 1976942). Copies of the data can be obtained free of charge from Cambridge Crystallographic Data Centre, 12, Union Road, Cambridge CB2 1EZ, U.K. [fax (+44)1223-336-033; or e-mail: data_request@ccdc.cam.ac.uk].

Penipyridinone A (**7**): Light-yellow powder; molecular formula C_25_H_35_NO_5_; ^13^C-NMR (150 MHz) and ^1^H-NMR (600 MHz) data (in DMSO-*d*_6_), see Table 4; HRESIMS *m*/*z* 430.2587 [M + H]^+^ (calcd for C_25_H_36_NO_5_^+^ 430.2593) and 452.2406 [M + Na]^+^ (calcd for C_25_H_35_NNaO_5_^+^ 452.2413).

Methyl linoleate (**8**): White amorphous powder; molecular formula C_19_H_34_O_2_; ^13^C- NMR (150 MHz) and ^1^H-NMR (600 MHz) data (in DMSO-*d*_6_), see Table S9; HRESIMS *m*/*z* 295.2626 [M + H]^+^ (calcd for C_19_H_35_O_2_^+^, 295.2637) and 317.2451 [M + Na]^+^ (calcd for C_19_H_34_NaO_2_^+^, 317.2457).

12*β*-Hydroxyverruculogen TR-2 (**9**): White amorphous powder; molecular formula C_22_H_27_N_3_O_6_; [*α*]^20^_D_ +51° (*c* 0.10, MeOH); ^13^C-NMR data (150 MHz, in DMSO-*d*_6_), see Table S10, ^1^H-NMR data (600 MHz, in DMSO-*d*_6_), see Table S11; HRESIMS *m*/*z* 430.1977 [M + H]^+^ (calcd for C_22_H_28_N_3_O_6_^+^, 430.1978) and 452.1794 [M + Na]^+^ (calcd for C_22_H_27_N_3_NaO_6_^+^, 452.1798).

12*β*-Hydroxy-13*α*-methoxyverruculogen TR-2 (**10**): White powder; molecular formula C_23_H_29_N_3_O_6_; [*α*]^20^_D_ +45° (*c* 0.10, MeOH); ^13^C-NMR data (150 MHz, in DMSO-*d*_6_), see Table S10, ^1^H-NMR data (600 MHz, in DMSO-*d*_6_), see Table S11; HRESIMS *m*/*z* 444.2123 [M + H]^+^ (calcd for C_23_H_30_N_3_O_6_^+^, 444.2135) and 466.1946 [M + Na]^+^ (calcd for C_23_H_29_N_3_NaO_6_^+^, 466.1954).

Cyclotryprostatin B (**11**): Yellow powder; molecular formula C_23_H_27_N_3_O_5_; [*α*]^20^_D_ +59° (*c* 0.10, MeOH); ^13^C-NMR data (150 MHz, in CDCl_3_), see Table S10, ^1^H-NMR data (600 MHz, in in CDCl_3_), see Table S11; HRESIMS *m*/*z* 426.2029 [M + H]^+^ (calcd for C_23_H_28_N_3_O_5_^+^, 426.2029) and 448.1837 [M + Na]^+^ (calcd for C_23_H_27_N_3_NaO_5_^+^, 448.1848).

Verruculogen (**12**): White powder; molecular formula C_27_H_33_N_3_O_7_; [*α*]^20^_D_ −37.8° (*c* 0.10, MeOH); ^13^C-NMR data (150 MHz, in DMSO-*d*_6_), see Table S10, ^1^H-NMR data (600 MHz, in DMSO-*d*_6_), see Table S11; HRESIMS *m*/*z* 512.2410 [M + H]^+^ (calcd for C_27_H_34_N_3_O_7_^+^, 512.2397) and 534.2202 [M + Na]^+^ (calcd for C_27_H_33_N_3_NaO_7_^+^, 534.2216).

Fumiquinazoline C (**13**): Light-yellow amorphous powder; molecular formula C_24_H_21_N_5_O_4_; [*α*]^20^_D_ −60.8° (*c* 0.10, MeOH); ^13^C-NMR data (150 MHz, in DMSO-*d*_6_), see Table S12, ^1^H-NMR data (600 MHz, in DMSO-*d*_6_), see Table S13; HRESIMS *m*/*z* 444.1666 [M + H]^+^ (calcd for C_24_H_22_N_5_O_4_^+^, 444.1672) and 466.1486 [M + Na]^+^ (calcd for C_24_H_21_N_5_NaO_4_^+^, 466.1491).

Fumiquinazoline J (**14**): White amorphous powder; molecular formula C_21_H_16_N_4_O_2_; [*α*]^20^_D_ −68° (*c* 0.10, MeOH); ^13^C-NMR data (150 MHz, in DMSO-*d*_6_), see Table S12, ^1^H-NMR data (600 MHz, in DMSO-*d*_6_), see Table S13; HRESIMS *m*/*z* 357.1343 [M + H]^+^ (calcd for C_21_H_17_N_4_O_2_^+^, 357.1352) and 379.1156 [M + Na]^+^ (calcd for C_21_H_16_N_4_NaO_2_^+^, 379.1171).

Brevianamide F (**15**): White colorless solid; molecular formula C_16_H_17_N_3_O_2_; [*α*]^20^_D_ −53° (*c* 0.10, MeOH); ^13^C-NMR data (150 MHz, in DMSO-*d*_6_), see Table S12, ^1^H-NMR data (600 MHz, in DMSO-*d*_6_), see Table S13; HRESIMS *m*/*z* 284.1396 [M + H]^+^ (calcd for C_16_H_18_N_3_O_2_^+^, 284.1399) and 306.1213 [M + Na]^+^ (calcd for C_16_H_17_N_3_NaO_2_^+^, 306.1218).

2-[(2*R*-Hydroxypropanoyl) amino] benzamide (**16**): White amorphous powder; molecular formula C_10_H_12_N_2_O_3_; [*α*]^20^_D_ +30° (*c* 0.10, MeOH); ^13^C-NMR data (150 MHz, in DMSO-*d*_6_), see Table S12, ^1^H-NMR data (600 MHz, in DMSO-*d*_6_), see Table S13; HRESIMS *m*/*z* 209.0921 [M + H]^+^ (calcd for C_10_H_13_N_2_O_3_^+^, 209.0926) and 231.0744 [M + Na]^+^ (calcd for C_10_H_12_N_2_NaO_3_^+^, 231.0746).

Trypacidin (**17**): White powder; molecular formula C_18_H_16_O_7_; [*α*]^20^_D_ −50° (*c* 0.10, MeOH); ^13^C-NMR data (150 MHz, in DMSO-*d*_6_), see Table S12, ^1^H-NMR data (600 MHz, in DMSO-*d*_6_), see Table S13; HRESIMS *m*/*z* 345.0969 [M + H]^+^ (calcd for C_18_H_17_O_7_^+^, 345.0974) and 367.0787 [M + Na]^+^ (calcd for C_18_H_16_NaO_7_^+^, 367.0794).

### 3.5. Enzymatic Hydrolysis of Peniresorcinoside A (**1**)

Peniresorcinoside A (**1**, 3.0 mg) was equilibrated at 37 °C for 5 min in a solution of water (2.0 mL) and 0.1 M phosphate buffer (pH = 7.0, 2.0 mL) and then 0.5 mL of α-glucosidase solution (0.2 M potassium phosphate solution containing 1 mM EDTA and 0.05% Tween-20, pH 7.0) was added. The mixture was incubated at 37 °C for 1 h and then 2.0 mL of 0.2 M Na_2_CO_3_ solution were added to terminate the enzymatic reaction. The enzymatic product was extracted with EtOAc (each 5 mL) three times to give an EtOAc extract and a water solution. The EtOAc extract was separated on an Agilent Zorbax SB-C18 column (250 × 9.4 mm, 5 µm; mobile phase: MeOH/H_2_O, 85/15; flow rate: 1.0 mL/min, UV detection: 210 nm) to furnish **1a** (1.8 mg, t_R_ 37.4 min). The aqueous solution was dried under reduced pressure to afford a residue which was first treated with 10 mg hydroxylamine hydrochloride in 2 mL pyridine at 90 °C for 30 min in a water bath and then mixed with 2 mL acetic anhydride at 90 °C for 1 h in a water bath. Finally, the reaction products were dried in vacuo and dissolved in 2 mL chloroform for GC analysis. The aldonitrile acetate of sugar in **1** was identified as the aldonitrile acetate of D-glucose (t_R_ 7.20 min) (Figure S3) by GC analysis with aldonitrile acetates of D-glucose (t_R_ 7.20 min), L-glucose (t_R_ 7.28 min), D-galactose (t_R_ 7.40 min), and L-galactose (t_R_ 7.45 min) as references.

Compound **1a**: White amorphous powder; molecular formula C_24_H_38_O_3_; ^13^C-NMR (150 MHz) and ^1^H-NMR (600 MHz) data (in MeOH-*d*_4_), see Table 1; HRESIMS *m*/*z*: 375.2893 [M + H]^+^ (calcd for C_24_H_39_O_3_^+^, 375.2899) and 397.2715 [M + Na]^+^ (calcd for C_24_H_38_NaO_3_^+^, 397.2719).

### 3.6. MTPA Esterification of Compound **1a**

Compound **1a** (0.8 mg) was dissolved in the anhydrous pyridine (0.5 mL) and then either (*R*)-α-methoxy-α-(trifluoromethyl)-phenylacetyl chloride (*R*-MTPA-Cl, 45 µL) or *S*-MTPA-Cl (45 µL) was added. The mixtures were stirred at 45 °C for 48 h until adding 1 mL MeOH to terminate the reaction. The reaction mixtures were dried under reduced pressure to give a residue. (*S*)-MTPA ester **1as** (0.4 mg, t_R_ 25.1 min, MeOH/H_2_O, 100/0) or (*R*)-MTPA ester **1ar** (0.4 mg, t_R_ 24.9 min, MeOH/H_2_O, 100/0) was obtained from the residue by HPLC purification using an Agilent Zorbax SB-C_18_ column (250 × 9.2 mm, 5 µm) at a flow rate of 1.0 mL/min and UV detection of 210 nm.

Compound **1as**: Molecular formula C_54_H_59_F_9_O_9_; ^1^H-NMR data (600 MHz, in MeOH-*d*_4_), see Table S2; HRESIMS *m*/*z*: 1045.3910 [M + Na]^+^ (calcd for C_54_H_59_F_9_NaO_9_^+^, 1045.3913).

Compound **1ar**: Molecular formula C_54_H_59_F_9_O_9_; ^1^H-NMR data (600 MHz, in MeOH-*d*_4_), see Table S2; HRESIMS *m*/*z*: 1045.3911 [M + Na]^+^ (calcd for C_54_H_59_F_9_NaO_9_^+^, 1045.3913).

### 3.7. Alkaline Hydrolysis of Peniresorcinosides D (**4**) and E (**5**)

Peniresorcinoside D (**4**, 1.6 mg) was hydrolyzed in 4 mL 3 N NaOH at 40 °C for 2 h in a water bath. The reaction mixture was neutralized with 3 N HCl and then extracted with EtOAc (each 5 mL) three times to give an EtOAc extract. This EtOAc extract was separated on a Zorbax SB-C_18_ column (250 × 9.4 mm, 5 µm; mobile phase: MeOH/0.1%TFA-H_2_O, 93/7; flow rate: 1.0 mL/min; UV detection: 210 nm) to give **1** (0.8 mg, t_R_ 12.8 min) and **4a** (0.5 mg, t_R_ 35.1 min). In the same way, alkaline hydrolysis of peniresorcinoside E (**5**, 2.1 mg) produced **1** (1.2 mg, t_R_ 34.5 min) and **5a** (0.7 mg, t_R_ 45.2 min).

Compound **4a**: Light yellow oil; molecular formula C_18_H_32_O_2_; ^13^C NMR (150 MHz) and ^1^H NMR (600 MHz) data (in DMSO-*d*_6_), see Table S9; HRESIMS *m*/*z* 279.2316 [M − H]^+^ (calcd for C_18_H_31_O_2_^−^, 279.2324).

Compound **5a**: Colorless oil; molecular formula C_18_H_34_O_2_; ^13^C NMR (150 MHz) and ^1^H NMR (600 MHz) data (in DMSO-*d*_6_), see Table S9; HRESIMS *m*/*z* 283.2645 [M + H]^+^ (calcd for C_18_H_35_O_2_^+^, 283.2637) and 305.2445 [M + Na]^+^ (calcd for C_18_H_34_NaO_2_^+^, 305.2457).

### 3.8. ^13^C- and ^1^H-NMR Calculations

Monte Carlo conformational searches were carried out by means of the Spartan’s 10 software using Merck Molecular Force Field (MMFF). The conformers with Boltzmann-population of over 5% for NMR calculations were initially optimized at B3LYP/6-31g (d, p) level in MeOH. Gauge-independent atomic orbital (GIAO) calculations of ^13^C and ^1^H NMR chemical shifts were accomplished by density functional theory (DFT) at the mPWLPW91-SCRF (DMSO)/6-311+g (d, p) level with the PCM solvent continuum model in Gaussian 09 software. The calculated NMR data of the lowest energy conformers for model molecules 20*R*,21*S*-**1a**, 20*S*,21*S*-**1a**, 20*R*,21*R*-**1a**, and 20*S*,21*R*-**1a** were averaged according to the Boltzmann distribution theory and their relative Gibbs free energy. The ^13^C-NMR and ^1^H-NMR chemical shifts for TMS were calculated by the same protocol as reported in the reference [23] and the experimental and calculated data of the isomeric compounds were analyzed by the improved probability DP4^+^ method [23]. A significant higher DP4^+^ probability score of the model molecules suggested the correctness of its configuration.

### 3.9. ECD Calculations

The X-ray CIF profile of 5*S*,8*S*,5′*S*,8′*S*-**6** was initially optimized at B3LYP/6-31g (d, p) level in MeOH. The theoretical calculation of ECD was conducted in MeOH using Time-dependent Density functional theory (TD-DFT) at the B3LYP/6-311+g (d, p) level. Under the same conditions, the enantiomer 5*R*,8*R*,5′*R*,8′*R*-**6** was also calculated. Rotatory strengths for a total of 30 excited states were calculated. ECD spectra were generated using the program SpecDis 1.6 (University of Würzburg, Würzburg, Germany) and GraphPad Prism 5 (University of California San Diego, San Diego, CA, USA) from dipole-length rotational strengths by applying Gaussian band shapes with sigma = 0.2 eV.

### 3.10. Sulforhodamine B (SRB) Assay

Human glioma U87MG and U251 cells were cultured in Minimum Essential Medium (MEM, Gibco, Thermo Fisher Scientific Inc., Waltham, MA, USA) and Dulbecco’s Modified Eagle Medium (DMEM, Gibco) with 10% FBS, respectively. All cells were incubated at 37°C in a humidified incubator with 5% CO_2_ incubator. Cells from the third repeated culture were used for experiments. The SRB assay as describe in previous publication [42] was used to evaluate the antiproliferative activity of all isolated compounds **1**–**17** against human glioma U87MG and C251 cells. Doxorubicin (DOX) was used as a positive control. Briefly, glioma cells in logarithmic growth (4 × 10^3^ cells/well) were plated in a 96-well plate, treated with different concentrations of each tested compound after 24 h of cells adhesion, and then incubated for 72 h. After that, the treated cells were fixed with 50 µL of 50% cold TCA (trichloroacetic acid) solution at 4 °C for 1 h, washed with distilled water five times, and then dried at 37 °C in a drying oven. The dried cells were stained with 50 µL of 0.4% SRB for 15 min, rinsed with 1% glacial acetic acid solution five times, then dried at 37 °C. Finnally, the dried dye was dissolved in 100 µL of 10 mM Tris buffer and the optical density (OD) value measured at 515 nm on a microplate reader (BioTech, Winooski, VT, USA). The cell viability (%) was calculated from the formula of T_OD_/C_OD_ × 100% (T_OD_: OD value of tested compound; C_OD_: OD value of negative control) and IC_50_ value was obtained based on the cell viability (%) by logistic calculation using SPSS software.

## 4. Conclusions

Chemical investigation of the metabolites produced by the marine-sourced fungus *Penicillium* sp. ZZ1750 cultured in rice medium resulted in the characterization of seven new compounds: peniresorcinosides A–E (**1**–**5**), penidifarnesylin A (**6**), and penipyridinone A (**7**), which enrich the structural diversity of the metabolites of marine *Penicillium* fungi. Peniresorcinosides A (**1**) and C (**2**) are rare glycosylated alkylresorcinols and had potent antiproliferative activity against both human glioma U87MG and U251 cells and might be the main components responsible for the antiglioma activity of the crude extract prepared the culture of strain ZZ1750 in rice medium.

## Figures and Tables

**Table 1:** ^13^C-NMR (150 MHz) and ^1^H-NMR (600 MHz) data of compounds **1**, **1a**, and **2**.

No.	1 *^a^*		1a *^b^*		2 *^a^*	
	*δ*_C_, Type	*δ*_H_, *J* in Hz	*δ*_C_, Type	*δ*_H_, *J* in Hz	*δ*_C_, Type	*δ*_H_, *J* in Hz
1	143.4, C	–	145.5, C	–	143.4, C	–
2	106.3, CH	6.02, d (2.0)	107.9, CH	6.13, d (2.0)	106.3, CH	6.02, d (2.3)
3	158.2, C	–	159.4, C	–	158.2, C	–
4	100.1, CH	6.00, t (2.0)	101.1, CH	6.08, t (2.0)	100.1, CH	6.01, t (2.3)
5	158.2, C	–	159.4, C	–	158.2, C	–
6	106.3, CH	6.02, d (2.0)	107.9, CH	6.13, d (2.0)	106.3, CH	6.02, d (2.3)
7	35.3, CH_2_	2.42, t (7.5)	37.1, CH_2_	2.50, t (7.5)	35.3, CH_2_	2.42, t (7.6)
8	33.6, CH_2_	2.23, q (7.5)	35.5 *^c^*, CH_2_	2.28, q (7.5)	33.6, CH_2_	2.23, q (7.6)
9	131.3, CH	5.55, m	132.2, CH	5.56, m	131.3, CH	5.55, m
10	130.5, CH	5.96, dd (15.3, 10.7)	131.8, CH	5.99, dd (14.8, 10.5)	130.6, CH	5.96, dd (14.8, 10.3)
11	130.3, CH	5.93, dd (14.8, 10.7)	132.2, CH	5.96, dd (14.8, 10.5)	130.3, CH	5.93, dd (14.8, 10.3)
12	132.3, CH	5.53, m	133.4, CH	5.52, m	132.3, CH	5.53, m
13	31.9, CH_2_	1.99, q (7.3)	30.8 *^e^*, CH_2_	2.03, q (7.1)	31.9, CH_2_	1.99, q (7.3)
14	28.9, CH_2_	1.32, m	30.6 *^d^*, CH_2_	1.37, m	28.9, CH_2_	1.32, m
15	28.6 *^c^*, CH_2_	1.24 *^e^*, m	30.3 *^d^*, CH_2_	1.29 *^e^*, m	28.6 *^c^*, CH_2_	1.24 *^e^*, m
16	28.9 *^c^*, CH_2_	1.24 *^e^*, m	30.5 *^d^*, CH_2_	1.29 *^e^*, m	28.9 *^c^*, CH_2_	1.24 *^e^*, m
17	29.3 *^c^*, CH_2_	1.24 *^e^*, m	30.8 *^e^*, CH_2_	1.29 *^e^*, m	29.5 *^c^*, CH_2_	1.24 *^e^*, m
18	29.2 *^c^*, CH_2_	1.24 *^e^*, m	27.4, CH_2_	1.29 *^e^*, m	29.2 *^c^*, CH_2_	1.24 *^e^*, m
19	24.9 *^d^*, CH_2_	1.41, m; 1.37, m	35.4 *^c^*, CH_2_	1.41, m	24.9 *^d^*, CH_2_	1.42, m; 1.38, m
20	81.0, CH	3.35, m	75.4, CH	3.42, m	81.7, CH	3.35, m
21	37.5, CH	1.58, m	41.5, CH	1.35, m	37.5, CH	1.57, m
22	24.8 *^d^*, CH_2_	1.48, m; 1.08, m	27.1, CH_2_	1.49, m; 1.15, m	24.7 *^d^*, CH_2_	1.48, m; 1.08, m
23	12.0, CH_3_	0.84, t (7.3)	12.2, CH_3_	0.90, t (7.3)	11.9, CH_3_	0.85, t (7.5)
24	14.5, CH_3_	0.81, d (6.9)	13.9, CH_3_	0.86, d (6.7)	14.4, CH_3_	0.82, d (6.8)
25	98.2, CH	4.70, d (3.7)			98.5, CH	4.70, d (3.7)
26	72.1, CH	3.15, dd (9.5, 3.7)			71.9, CH	3.17, m
27	73.0, CH	3.37, t (9.5)			72.9, CH	3.37, t (9.3)
28	70.1, CH	3.07, t (9.5)			70.5, CH	3.00, t (9.3)
29	73.0, CH	3.43, m			70.2, CH	3.66, m
30	60.8, CH_2_	3.53, dd (11.5, 2.0); 3.46, dd (11.5, 4.7)			64.0, CH_2_	4.25, dd (11.7, 1.9); 3.95, dd (11.7, 7.3)
1′					170.2, C	–
2′					20.6, CH_3_	1.98, s
OH-3					–	9.06, br s
OH-26					–	4.59, d (4.9)
OH-27					–	4.89, br s
OH-28					–	5.17, br s

*^a,b^* The data were recorded in DMSO-*d*_6_ and MeOH-*d*_4_, respectively; *^c,d^* the data with the same label in each column may be interchanged; *^e^* The data with the same label in each column were overlapped.

**Table 2:** ^13^C-NMR (150 MHz) and ^1^H-NMR (600 MHz) data (in DMSO-*d*_6_) of compounds **3**–**5**.

No.	3		4		5	
	*δ*_C_, Type	*δ*_H_, *J* in Hz			*δ*_C_, Type	*δ*_H_, *J* in Hz
1	143.4, C	–	143.4, C	–	143.4, C	–
2	106.3, CH	6.02, d (1.9)	106.3, CH	6.02, d (1.9)	106.3, CH	6.02, d (1.8)
3	158.2, C	–	158.2, C	–	158.2, C	–
4	100.1, CH	6.01, t (1.9)	100.1, CH	6.01, t (1.9)	100.1, CH	6.01, t (1.8)
5	158.2, C	–	158.2, C	–	158.2, C	–
6	106.3, CH	6.02, d (1.9)	106.3, CH	6.02, d (1.9)	106.3, CH	6.02, d (1.8)
7	35.3, CH_2_	2.42, t (7.3)	35.3, CH_2_	2.42, t (7.4)	35.3, CH_2_	2.42, t (7.3)
8	33.6 *^a^*, CH_2_	2.22, q (7.5)	33.7 *^a^*, CH_2_	2.23 *^e^*, q (7.4)	33.6 *^a^*, CH_2_	2.22, q (7.3)
9	131.3, CH	5.55, m	131.3, CH	5.55, m	131.3, CH	5.54, m
10	130.5, CH	5.95, dd (15.0, 10.5)	130.5, CH	5.95, dd (15.1, 10.7)	130.5, CH	5.95, dd (15.6, 10.6)
11	130.2, CH	5.93, dd (15.0, 10.5)	130.2, CH	5.93, dd (15.1, 10.7)	130.3, CH	5.93, dd (15.6, 10.6)
12	132.3, CH	5.52, m	132.3, CH	5.52, m	132.3, CH	5.52, m
13	31.9, CH_2_	1.99, q (7.5)	32.0, CH_2_	2.00 *^f^*, q (7.3)	32.0, CH_2_	1.98, q (7.4)
14	28.9 *^b^*, CH_2_	1.32, m	28.9 *^b^*, CH_2_	1.32, m	28.9 *^b^*, CH_2_	1.32, m
15	28.6 *^b^*, CH_2_	1.23 *^e^*, m	28.6 *^b^*, CH_2_	1.24 *^g^*, m	28.6 *^b^*, CH_2_	1.23 *^e^*, m
16	28.9 *^b^*, CH_2_	1.23 *^e^*, m	28.9 *^b^*, CH_2_	1.24 *^g^*, m	28.9 *^b^*, CH_2_	1.23 *^e^*, m
17	29.3 *^b^*, CH_2_	1.23 *^e^*, m	29.3 *^b^*, CH_2_	1.24 *^g^*, m	29.3 *^b^*, CH_2_	1.23 *^e^*, m
18	29.2 *^b^*, CH_2_	1.23 *^e^*, m	29.2 *^b^*, CH_2_	1.24 *^g^*, m	29.2 *^b^*, CH_2_	1.23 *^e^*, m
19	24.9 *^e^*, CH_2_	1.41, m; 1.37, m	24.9 *^e^*, CH_2_	1.40, m; 1.36, m	24.9 *^e^*, CH_2_	1.41, m; 1.36, m
20	81.0, CH	3.35, m	81.1, CH	3.35, m	81.1, CH	3.35, m
21	37.5, CH	1.57, m	37.5, CH	1.56, m	37.5, CH	1.57, m
22	24.9 *^e^*, CH_2_	1.49, m; 1.12, m	24.9 *^e^*, CH_2_	1.49, m; 1.11, m	24.9 *^e^*, CH_2_	1.49, m; 1.11, m
23	12.0, CH_3_	0.85, t (7.3)	12.0, CH_3_	0.85, t (7.4)	12.0, CH_3_	0.85, t (7.3)
24	14.3, CH_3_	0.82, d (6.7)	14.4, CH_3_	0.82, d (6.9)	14.3, CH_3_	0.82, d (6.8)
25	98.0, CH	4.70, d (3.8)	98.1, CH	4.70, d (3.9)	98.0, CH	4.70, d (3.7)
26	71.8, CH	3.16, m	71.9, CH	3.18, m	71.9, CH	3.17, m
27	72.7, CH	3.39, m	72.8, CH	3.39, m	72.8, CH	3.39, m
28	70.6, CH	2.98, m	70.6, CH	2.98, m	70.6, CH	2.98, m
29	70.2, CH	3.66, t (8.6)	70.2, CH	3.66, t (8.5)	70.2, CH	3.66, t (8.8)
30	64.3, CH_2_	4.26, d (10.8); 3.95, dd (10.8, 7.3)	64.1, CH_2_	4.27, d (11.2); 3.95, dd (11.2, 7.5)	64.1, CH_2_	4.27, d (11.2); 3.95, dd (11.2, 7.5)
1′	172.7, C	–	172.7, C	–	172.7, C	–
2′	33.5 *^a^*, CH_2_	2.25, t (7.2)	33.6 *^a^*, CH_2_	2.23 *^e^*, t (7.4)	33.6 *^a^*, CH_2_	2.23, t (7.3)
3′	24.4, CH_2_	1.50, m	24.4, CH_2_	1.50, m	24.4, CH_2_	1.50, m
4′	29.0 *^b^*, CH_2_	1.23 *^e^*, m	28.7 *^b^*, CH_2_	1.24 *^g^*, m	28.7 *^b^*, CH_2_	1.23 *^e^*, m
5′	29.0 *^b^*, CH_2_	1.23 *^e^*, m	28.5 *^b^*, CH_2_	1.24 *^g^*, m	28.5 *^b^*, CH_2_	1.23 *^e^*, m
6′	29.0 *^b^*, CH_2_	1.23 *^e^*, m	28.5 *^b^*, CH_2_	1.24 *^g^*, m	28.5 *^b^*, CH_2_	1.23 *^e^*, m
7′	29.0 *^b^*, CH_2_	1.23 *^e^*, m	28.4 *^b^*, CH_2_	1.24 *^g^*, m	28.4 *^b^*, CH_2_	1.23 *^e^*, m
8′	28.9 *^b^*, CH_2_	1.23 *^e^*, m	26.6 *^f^*, CH_2_	2.00 *^f^*, q (7.3)	26.5 *^f^*, CH_2_	1.95 *^f^*, q (7.0)
9′	28.8 *^b^*, CH_2_	1.23 *^e^*, m	129.7 *^c^*, CH	5.28 *^h^*, m	129.7 *^c^*, CH	5.29 *^g^*, m
10′	28.8 *^b^*, CH_2_	1.23 *^e^*, m	127.8 *^d^*, CH	5.31 *^i^*, m	129.6 *^c^*, CH	5.29 *^g^*, m
11′	28.8 *^b^*, CH_2_	1.23 *^e^*, m	25.2, CH_2_	2.72, t (6.6)	26.5 *^f^*, CH_2_	1.95 *^f^*, q (7.0)
12′	28.7 *^b^*, CH_2_	1.23 *^e^*, m	127.7 *^d^*, CH	5.28 *^h^*, m	29.0 *^b^*, CH_2_	1.23 *^e^*, m
13′	28.4 *^b^*, CH_2_	1.23 *^e^*, m	129.6 *^c^*, CH	5.31 *^i^*, m	28.8 *^b^*, CH_2_	1.23 *^e^*, m
14′	31.3, CH_2_	1.23 *^e^*, m	26.6 *^f^*, CH_2_	2.00 *^f^*, q (7.3)	28.6 *^b^*, CH_2_	1.23 *^e^*, m
15′	22.1, CH_2_	1.23 *^e^*, m	29.0 *^b^*, CH_2_	1.24 *^g^*, m	29.0 *^b^*, CH_2_	1.23 *^e^*, m
16′	13.9, CH_3_	0.84, t (7.0)	30.9, CH_2_	1.24 *^g^*, m	31.3, CH_2_	1.23 *^e^*, m
17′	–	–	22.0, CH_2_	1.24 *^g^*, m	22.1, CH_2_	1.23 *^e^*, m
18′	–	–	13.9, CH_3_	0.84, t (7.3)	13.9, CH_3_	0.83, t (7.0)
OH-3/5	–	9.04, s	–	9.05, s	–	9.06, s
OH-26	–	4.57, d (6.1)	–	4.57, d (6.1)	–	4.59, d (6.0)
OH-27	–	4.85, d (4.2)	–	4.85, br s	–	4.88, br s
OH-28	–	5.15, d (5.6)	–	5.15, d (5.5)	–	5.17, d (5.3)

*^a^*^–*d*^ The data with the same label in each column may be interchanged; *^e^*^–*i*^ The data with the same label in each column were overlapped.

**Table 3:** ^13^C NMR (150 MHz) and ^1^H NMR (600 MHz) data of penidifarnesylin A (**6**) (in DMSO-*d*_6_).

No.	^13^C, Type	^1^H (*J* in Hz)	No.	^13^C, Type	^1^H (*J* in Hz)
1/1′	25.6, CH_3_	1.63, s	10/10′	131.9, C	–
2/2′	131.2, C	–	11/11′	126.1, CH	5.08, t (6.3)
3/3′	121.6, CH	5.02, t (7.6)	12/12′	27.9, CH_2_	1.92 *^a^*, m
4/4′	34.2, CH_2_	2.06, m; 1.99, m	13/13′	17.7, CH_3_	1.54, s
5/5′	76.1, CH	3.73, m	14/14′	11.2, CH_3_	1.50, s
6/6′	137.2, C	–	15/15′	16.5, CH_3_	1.56, s
7/7′	129.5, CH	5.16, d (8.2)	OH-5/5′	–	4.64, d (3.6)
8/8′	65.7, CH	4.25, m	OH-8/8′	–	4.41, d (4.6)
9/9′	48.1, CH_2_	2.10, m; 1.92 *^a^*, m			

*^a^* Data with the same label in each column were overlapped.

**Table 4:** ^13^C-NMR (150 MHz) and ^1^H-NMR (600 MHz) data of penipyridinone A (**7**, in DMSO-*d*_6_).

No	^13^C, Type	^1^H (*J* in Hz)	No	^13^C, Type	^1^H (*J* in Hz)
1	69.7, CH_2_	3.69, dd (11.5, 1.7); 3.56, dd (11.5, 2.3)	14	13.6, CH_3_	0.87, t (7.4)
2	31.5, CH	2.27, m	15	10.5, CH_3_	0.94, d (7.0)
3	80.3, CH	3.42, dd (9.5, 5.5)	16	11.5, CH_3_	1.66, s
4	70.0, CH	4.84, t (9.5)	17	55.7, CH_3_	3.25, s
5	83.7, CH	3.58, d (9.5)	18	167.5, C	–
6	132.8, C	–	19	52.7, CH_2_	4.85, d (18.2); 4.75, d (18.2)
7	129.3, CH	5.97, d (10.1)	20	148.7, C	–
8	125.9, CH	6.29, dd (15.0, 10.1)	21	117.7, CH	5.98, d (2.8)
9	134.2, CH	6.24, dd (15.0, 10.1)	22	177.8, C	–
10	130.7, CH	6.13, dd (15.6, 10.1)	23	116.0, CH	5.93, dd (8.0, 2.8)
11	135.3, CH	5.74, m	24	142.7, CH	7.41, d (8.0)
12	34.3, CH_2_	2.05, m	25	18.4, CH_3_	2.01, s
13	21.9, CH_2_	1.36, m			

**Table 5:** Antiproliferative activity of compounds against human glioma cells (IC_50_: µM).

Compound	U87MG	U251	Compound	U87MG	U251
**1**	4.0 ± 0.2	14.1 ± 0.4	**5**	22.1 ± 0.3	NA
**2**	5.6 ± 0.5	9.8 ± 0.9	**6**	5.9 ± 0.3	27.6 ± 1.8
**3**	53.0 ± 1.0	NA	Doxorubicin	0.5 ± 0.0	3.5 ± 0.4
**4**	19.4 ± 1.6	NA			

NA: No activity at a concentration of 50 µM.

## Supplementary Materials

The following are available online at https://www.mdpi.com/article/10.3390/md19090483/s1, Figure S1: The colonies of *Penicillium* sp. ZZ1750 in PDA medium and the state of *Penicillium* sp. ZZ1750 in PDB medium; Figure S2: ITS rDNA sequence of *Penicillium* sp. ZZ1750; Figure S3: GC analytic results of aldonitrile acetates of sugars; Figure S4: COSY and HMBC correlations of peniresorcinosides C–E (**3**–**5**); Figure S5: Chromatogram of co-HPLC analysis of hydrolytic peniresorcinoside A with standard peniresorcinoside A (**1**); Figure S6: Chromatogram of co-HPLC analysis of compound **4a** with standard linoleic acid; Figure S7: Chromatogram of co-HPLC analysis of compound **5a** with standard oleic acid; Figures S8–S152: NMR, HRESIMS, UV, and IR spectra of compounds **1**–**7**, **1a**, **1as**, and **1ar**; Table S1: Sequences producing significant alignments of strain ZZ1750; Table S2: ^1^H NMR data of the MTPA esters **1as** (*S*-MTPA) and **1ar** (*R*-MTPA) of compound **1a**; Table S3: Experimental ^13^C NMR data of **1a** and calculated ^13^C NMR data of 20*R*,21*S*-**1a**, 20*S*,21*S*-**1a**, 20*R*,21*R*-**1a**, and 20*S*,21*R*-**1a**; Table S4: Experimental ^1^H NMR data of **1a** and calculated ^1^H NMR data of 20*R*,21*S*-**1a**, 20*S*,21*S*-**1a**, 20*R*,21*R*-**1a**, and 20*S*,21*R*-**1a**; Table S5. Results analyzed by the improved probability DP4^+^ method based on the experimental NMR data of **1a** and calculated NMR data of 20*R*,21*S*-**1a**, 20*S*,21*S*-**1a**, 20*R*,21*R*-**1a**, and 20*S*,21*R*-**1a****;** Table S6: Crystal data and structure refinement parameters of penidifarnesylin A (**6**); Table S7–S8: Cartesian coordinates for the low-energy reoptimized MMFF conformers of 5*R*,8*R*,5′*R*,8′*R*-**6** and 5*S*,8*S*,5′*S*,8′*S*-**6** at B3LYP/6-311+G (d,p) level of theory in MeOH; Table S9–S13: ^13^C and ^1^H NMR data of known compounds **8**–**17**.
