# Supplementary material for: New Antiproliferative Compounds against Glioma Cells from the Marine-Sourced Fungus Penicillium sp. ZZ1750
Source: Mar Drugs. 2021 Aug 26;19(9):483. doi: 10.3390/md19090483 (PMC8465473; doi:10.3390/md19090483)
Supplement: Supplementary file 1 [file marinedrugs-19-00483-s001.zip › marinedrugs-1354325-supplementary.pdf]

SUPPLEMENTARY MATERIALS FOR

New Antiproliferative Metabolites against Glioma Cells from the Marine-Sourced Fungus *Penicillium* sp. ZZ1750

Kuo Yong [1], Sidra Kaleem [1], Bin Wu * and Zhizhen Zhang *

Ocean College, Zhoushan Campus, Zhejiang University, Zhoushan 316021, China; yongkuo@zju.edu.cn (K.Y.), kaleemsidra85@yahoo.com (S. K.)

[1] These authors contributed equally.

***** Correspondence: wubin@zju.edu.cn (B.W.); zzhang88@zju.edu.cn (Z.Z.); Tel.: +86-13675859706 (Z.Z.)

CONTENT

Table S1. Sequences producing significant alignments of strain ZZ1750…………………..…5
Table S2. ^{1}H NMR data of the MTPA esters **1as** (*S*-MTPA) and **1ar** (*R*-MTPA) of compound **1a** (600 MHz, in MeOH-d_4, *J* in Hz)…………………………………………………………6
Table S3. Experimental ^{13}C NMR data of **1a** and calculated ^{13}C NMR data of 20*R*,21*S*-**1a**, 20*S*,21*S*-**1a**, 20*R*,21*R*-**1a**, and 20*S*,21*R*-**1a** (ppm)………………………………………..7
Table S4. Experimental ^{1}H NMR data of **1a** and calculated ^{1}H NMR data of 20*R*,21*S*-**1a**, 20*S*,21*S*-**1a**, 20*R*,21*R*-**1a**, and 20*S*,21*R*-**1a** (ppm)………………………………………..8
Table S5. Results analyzed by the improved probability DP4^{+} method based on the experimental NMR data of **1a** and calculated NMR data of 20*R*,21*S*-**1a**, 20*S*,21*S*-**1a**, 20*R*,21*R*-**1a**, and 20*S*,21*R*-**1a**……………………..………………………..……..……8
Table S6. Crystal data and structure refinement for penidifarnesylin A (**6**)…………………9
Table S7. Cartesian coordinates for the low-energy reoptimized MMFF conformers of 5*R*,8*R*,5′*R*,8′*R*-**6** at B3LYP/6-311+G (d,p) level of theory in MeOH………………………9
Table S8. Cartesian coordinates for the low-energy reoptimized MMFF conformers of 5*S*,8*S*,5′*S*,8′*S*-**6** at B3LYP/6-311+G (d,p) level of theory in MeOH………………………11
Table S9. ^{13}C NMR (150 MHz) and ^{1}H NMR (600 MHz) data of compounds **4a**, **5a**, and **8** (in DMSO-d_6)……...………………………………………………………………..…….14
Table S10. ^{13}C NMR data of compounds **9**–**12** (150 MHz)……………………….……15
Table S11. ^{1}H NMR data of compounds **9**–**12** (600 MHz, *J* in Hz)……………………..16
TableS12. ^{13}C NMR data of compounds **13**–**17** (150 MHz, in DMSO-d_6)…………………17
Table S13. ^{1}H NMR data of compounds **13**–**17** (600 MHz, *J* in Hz, in DMSO-d_6)………18
Figure S1. The colonies of *Penicillium* sp. ZZ1750 in PDA medium (**A**) and the state of *Penicillium* sp. ZZ1750 in PDB medium (**B**)…………………………………………..19
Figure S2. ITS rDNA sequence of *Penicillium* sp. ZZ1750……………………….………19
Figure S3. GC analytic results of aldonitrile acetates of sugars…………………………...20
Figure S4. COSY and key HMBC correlations of peniresorcinosides C–E (**3**–**5**)………...20
Figure S5. Chromatogram of co-HPLC analysis of hydrolytic peniresorcinoside A with standard peniresorcinoside A (**1**)…………………………………………………………..…21
Figure S6. Chromatogram of co-HPLC analysis of compound **4a** with standard linoleic acid……………………………………………………………………………………..21
Figure S7. Chromatogram of co-HPLC analysis of compound **5a** with standard oleic acid...21

Figure S8-S11. ^{1}H NMR spectrum of peniresorcinoside A (**1**, 600 MHz, in DMSO-d_6)…....22
Figure S12-S15. ^{13}C NMR spectrum of peniresorcinoside A (**1**, 150 MHz, in DMSO-d_6).…24
Figure S16-S19. DEPT spectrum of peniresorcinoside A (**1**, in DMSO-d_6)………….….…26
Figure S20-S22. HMQC spectrum of peniresorcinoside A (**1**, in DMSO-d_6)……..………...28
Figure S23. COSY spectrum of peniresorcinoside A (**1**, in DMSO-d_6)……..……………..29
Figure S24-S26. HMBC spectrum of peniresorcinoside A (**1**, in DMSO-d_6)…………..……30
Figure S27. HRESIMS spectrum of peniresorcinoside A (**1**)………………………………31
Figure S28. UV (MeOH) spectrum of peniresorcinoside A (**1**)……………………………32
Figure S29. IR (ATR) spectrum of peniresorcinoside A (**1**)……………….………………32
Figure S30-S32. ^{1}H NMR spectrum of compound **1a** (600 MHz, in MeOH-d_4)…………..33
Figure S33-S34. ^{13}C NMR spectrum of compound **1a** (150 MHz, in MeOH-d_4)…………..34
Figure S35. HMQC spectrum of compound **1a** (in MeOH-d_4)……………………………35
Figure S36. COSY spectrum of compound **1a** (in MeOH-d_4)………………………...…36
Figure S37. HRESIMS spectrum of compound **1a**………………………………………36
Figure S38. ^{1}H NMR spectrum of compound **1as** (600 MHz, in MeOH-d_4)………………37
Figure S39. COSY spectrum of compound **1as** (in MeOH-d_4)…………………………..…37
Figure S40. HRESIMS spectrum of compound **1as**……………………….…………………38
Figure S41. ^{1}H NMR spectrum of compound **1ar** (600 MHz, in MeOH-d_4)………………38
Figure S42. COSY spectrum of compound **1ar** (in MeOH-d_4)……………………………39
Figure S43. HRESIMS spectrum of compound **1ar**……………………….…………………39
Figure S44-S46. ^{1}H NMR spectrum of peniresorcinoside B (**2**, 600 MHz, in DMSO-d_6)….40
Figure S47-S50. ^{13}C NMR spectrum of peniresorcinoside B (**2**, 150 MHz, in DMSO-d_6)….41
Figure S51-S53. DEPT spectrum of peniresorcinoside B (**2**, in DMSO-

d_6)……..………..…43
Figure S54-S57. HMQC spectrum of peniresorcinoside B (**2**, in DMSO-d_6)…..…………..45
Figure S58. COSY spectrum of peniresorcinoside B (**2**, in DMSO-d_6)……..……..…..…..…47
Figure S59-S63. HMBC spectrum of peniresorcinoside B (**2**, in DMSO-d_6)…..……..…..…47
Figure S64. HRESIMS spectrum of peniresorcinoside B (**2**) ………………..…………50
Figure S65. UV (MeOH) spectrum of peniresorcinoside B (**2**)……………………………50
Figure S66. IR (ATR) spectrum of peniresorcinoside B (**2**)……….…………………………51
Figure S67-S70. ^{1}H NMR spectrum of peniresorcinoside C (**3**, 600 MHz, in DMSO-d_6)..….51
Figure S71-S73. ^{13}C NMR spectrum of peniresorcinoside C (**3**, 150 MHz, in DMSO-d_6)…..53
Figure S74-S75. HMQC spectrum of peniresorcinoside C (**3**, in DMSO-d_6)……………..…55
Figure S76. COSY spectrum of peniresorcinoside C (**3**, in DMSO-d_6)……………..……..…56
Figure S77-S78. HMBC spectrum of peniresorcinoside C (**3**, in DMSO-d_6)……...……...…56
Figure S79. HRESIMS spectrum of peniresorcinoside C (**3**) ……………………….….……57
Figure S80. UV (MeOH) spectrum of peniresorcinoside C (**3**)……………………………58
Figure S81. IR (ATR) spectrum of peniresorcinoside C (**3**)…………………..……………58
Figure S82-S85. ^{1}H NMR spectrum of peniresorcinoside D (**4**, 600 MHz, in DMSO-d_6)..…59
Figure S86-S89. ^{13}C NMR spectrum of peniresorcinoside D (**4**, 150 MHz, in DMSO-d_6)…..61
Figure S90-S92. HMQC spectrum of peniresorcinoside D (**4**, in DMSO-d_6)……………..…63
Figure S_{93}-S_{94}. COSY spectrum of peniresorcinoside D (**4**, in DMSO-d_6)……..…………..…64
Figure S95-S97. HMBC spectrum of peniresorcinoside D (**4**, n DMSO-d_6)………...…...…66
Figure S98. HRESIMS spectrum of peniresorcinoside D (**4**) ………………………….…...…67
Figure S99. UV (MeOH) spectrum of peniresorcinoside D (**4**)……………………………67

Figure S100. IR (ATR) spectrum of peniresorcinoside D (**4**)…..……………………..……67
Figure S101-S103. ^{1}H NMR spectrum of peniresorcinoside E (**5**, 600 MHz, in DMSO-d_6)…………………………………………………………………………………....68
Figure S104-S107. ^{13}C NMR spectrum of peniresorcinoside E (**5**, 150 MHz, in DMSO-d_6)...69
Figure S108-S109. HMQC spectrum of peniresorcinoside E (**5**, in DMSO-d_6)….……..……71
Figure S110. COSY spectrum of peniresorcinoside E (**5**, in DMSO-d_6)………..…..……72
Figure S111-S112. HMBC spectrum of peniresorcinoside E (**5**, in DMSO-d_6)…..…..………73
Figure S113. HRESIMS spectrum of peniresorcinoside E (**5**). ……………………..……74
Figure S114. UV (MeOH) spectrum of peniresorcinoside E (**5**)…….……………………74
Figure S115. IR (ATR) spectrum of peniresorcinoside E (**5**)……………….………………75
Figure S116-S118. ^{1}H NMR spectrum of penidifarnesylin A (**6**, 600 MHz, in DMSO-d_6)…..75
Figure S119-S121. ^{13}C NMR spectrum of penidifarnesylin A (**6**, 150 MHz, in DMSO-d_6)…..77
Figure S122-S123. HMQC spectrum of penidifarnesylin A (**6**, in DMSO-d_6)………....……78
Figure S124. COSY spectrum of penidifarnesylin A (**6**, in DMSO-d_6)………….………79
Figure S125-S129. HMBC spectrum of penidifarnesylin A (**6**, in DMSO-d_6)…….…………80
Figure S130. HRESIMS spectrum of penidifarnesylin A (**6**)………………………….…82
Figure S131. UV (MeOH) spectrum of penidifarnesylin A (**6**)…………….…………………83
Figure S132. IR (ATR) spectrum of penidifarnesylin A (**6**)…………………………………83
Figure S133-S135. ^{1}H NMR spectrum of penipyridinone A (**7**, 600 MHz, in DMSO-d_6)...…84

Figure S136-S139. 13C NMR spectrum of penipyridinone A (7, 150 MHz, in DMSO-d6)...…85
Figure S140-S143. HMQC spectrum of penipyridinone A (7, in DMSO-d6)………..…..…87
Figure S144-S146. COSY spectrum of penipyridinone A (7, in DMSO-d6)……...……...…89
Figure S147-S150. HMBC spectrum of penipyridinone A (7, in DMSO-d6)………...……91
Figure S151. NOESY spectrum of penipyridinone A (7, in DMSO-d6)……..……..………..93
Figure S152. HRESIMS spectrum of penipyridinone A (**7**)………………….……………..93

Table S1. Sequences producing significant alignments of strain ZZ1750

Accession	Description	Max score	Total score	Query coverage	Evalue	Ident
MT364482.1	*Penicillium rubens* strain EF5 small subunit ribosomal RNA gene, partial sequence; internal transcribed spacer 1, 5.8S ribosomal RNA gene, and internal transcribed spacer 2, complete sequence; and large subunit ribosomal RNA gene, partial sequence	1040	1040	100%	0.0	100%
MK830090.1	*Penicillium chrysogenum* strain CD3 small subunit ribosomal *RNA* gene, partial sequence; internal transcribed spacer 1, 5.8S ribosomal *RNA* gene	1040	1040	100%	0.0	100%
MH863913.1	*Penicillium chrysogenum* strain CBS 126337 small subunit ribosomal *RNA* gene, partial sequence; internal transcribed spacer 1, 5.8S ribosomal *RNA* gene	1040	1040	100%	0.0	100%
MN341258.1	*Penicillium rubens* isolate KoRLI047057 small subunit ribosomal RNA gene, partial sequence; internal transcribed spacer 1, 5.8S ribosomal RNA gene, and internal transcribed spacer 2, complete sequence; and large subunit ribosomal RNA gene, partial sequence	1040	1040	100%	0.0	100%
MN413181.1	*Penicillium rubens* strain DTO269E3 small subunit ribosomal RNA gene, partial sequence; internal transcribed spacer 1, 5.8S ribosomal RNA gene, and internal transcribed spacer 2, complete sequence; and large subunit ribosomal RNA gene, partial sequence	1040	1040	100%	0.0	100%
MN413180.1	*Penicillium rubens* strain DTO269C2 small subunit ribosomal RNA gene, partial sequence; internal transcribed spacer 1, 5.8S ribosomal RNA gene, and internal transcribed spacer 2, complete sequence; and large subunit ribosomal RNA gene, partial sequence	1040	1040	100%	0.0	100%

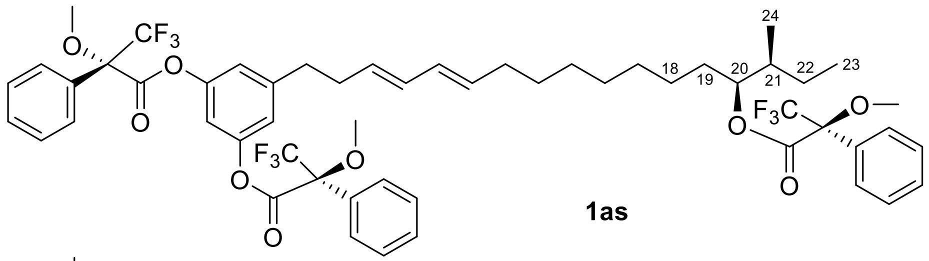

1as

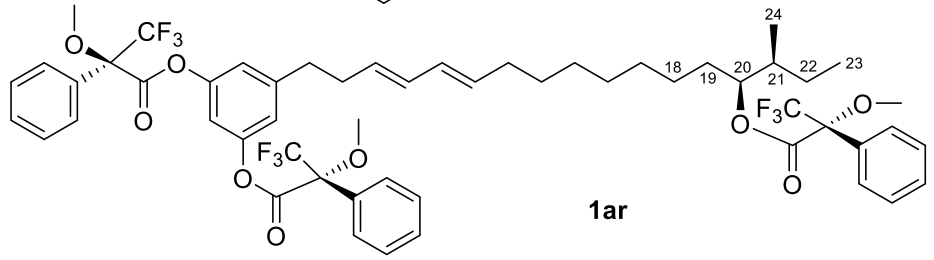

1ar

Table S2. ^{1}H NMR data of the MTPA esters **1as** (*S*-MTPA) and **1ar** (*R*-MTPA) of compound **1a** (600 MHz, in MeOH-d_4, J in Hz)

No.	**1as** (*S*-MTPA)	**1ar** (*R*-MTPA)	$\Delta\delta_{S\text{-}R}$
2	6.96, 1H, d (1.8)	6.96, 1H, d (2.1)	0
4	6.83, 1H, t (1.8)	6.83, 1H, t (2.1)	0
6	6.96, 1H, d (1.8)	6.96, 1H, d (1.8)	0
7	2.76, 2H, t (7.5)	2.75, 2H, t (7.5)	0
8	2.36, 2H, q (7.5)	2.36, 2H, q (7.)	0
9	5.55, 1H, m	5.55, 1H, m	0
10	5.99, 1H, m	5.99, 1H, m	0
11	5.96, 1H, m	5.96, 1H, m	0
12	5.53, 1H, m	5.53, 1H, m	0
13	2.01, 2H, q (7.3)	2.02, 2H, q (7.3)	–0.01
14	1.34, 2H, m	1.36, 2H, m	–0.02
15	1.29[a], 2H, m	1.29[a], 2H, m	0
16	1.29[a], 2H, m	1.29[a], 2H, m	0
17	1.29[a], 2H, m	1.29[a], 2H, m	0
18	1.15, 2H, m	1.29, 2H, m	–0.14
19	1.55, 2H, m	1.65, 2H, m	–0.10
20	5.07, 1H, m	5.08, 1H, m	–0.01
21	1.63, 1H, m	1.60, 1H, m	+0.03
22	1.43, 1H, m; 1.17, 1H, m	1.26, 1H, m; 1.06, 1H, m	+0.17; +0.11
23	0.91, 3H, t (7.3)	0.83, 3H, t (7.5)	+0.08
24	0.90, 3H, d (7.3)	0.84, 3H, d (6.5)	+0.06
MTPA	7.62, 4H, m; 7.50, 8H, m; 7.40, 3H, m; 3.69, 6H, s; 3.52, 3H, s	7.62, 4H, m; 7.50, 8H, m; 7.40, 3H, m; 3.68, 6H, s; 3.53, 3H, s	

[a] The data with the same label in each column were overlapped.

Table S3. Experimental ^{13}C NMR data of **1a** and calculated ^{13}C NMR data of 20*R*,21*S*-**1a**, 20*S*,21*S*-**1a**, 20*R*,21*R*-**1a**, and 20*S*,21*R*-**1a** (ppm)

No.	**1a**	20*R*,21*S*-**1a**	20*S*,21*S*-**1a**	20*R*,21*R*-**1a**	20S,21*R***1a**
1	145.5	148.9915	149.4535	149.246	149.4629
2	107.9	109.3812	109.8104	108.701	109.6127
3	159.4	164.7215	164.3768	161.9534	163.9774
4	101.1	101.6212	101.3839	101.7902	101.4194
5	159.4	163.9533	164.2801	165.7164	164.5564
6	107.9	109.8828	109.3916	111.5481	109.3892
7	37.1	40.72122	40.3334	38.23818	41.11264
8	35.5	39.4452	39.25168	38.29247	39.05133
9	132.3	139.5868	138.9278	135.3962	139.8048
10	132.2	135.997	137.2303	140.26	136.708
11	131.8	140.2181	140.0304	137.2433	140.3823
12	133.4	137.163	138.0762	139.8182	137.5467
13	30.8	32.57383	36.17204	34.73373	36.04422
14	30.6	29.61611	31.88739	35.55337	31.57447
15	30.3	26.31633	28.98226	27.69833	29.1165
16	30.5	28.73977	31.55154	32.0887	31.52192
17	30.8	28.81714	32.68721	31.7095	32.62244
18	27.4	24.29688	27.32133	27.8516	27.42727
19	35.4	35.86634	38.62371	39.67647	36.51792
20	75.4	76.89977	76.56021	73.97195	81.64929
21	41.5	43.55021	44.20764	43.86623	44.42652
22	27.1	23.20592	28.03516	28.40769	23.54765
23	12.2	11.78566	12.05581	11.82026	12.48054
24	13.9	16.93455	12.90461	13.19907	16.09433

Table S4. Experimental ^{1}H NMR data of **1a** and calculated ^{1}H NMR data of 20*R*,21*S*-**1a**, 20*S*,21*S*-**1a**, 20*R*,21*R*-**1a**, and 20*S*,21*R*-**1a** (ppm)

No.	**1a**	20*R*,21*S*-**1a**	20*S*,21*S*-**1a**	20*R*,21*R*-**1a**	20S,21*R*-**1a**
2	6.13	6.4200	6.1800	5.5700	6.4200
4	6.08	5.9847	5.950831	6.092276	5.97041
6	6.13	6.2442	6.233749	6.456771	6.092167
7	2.50	2.3470	2.409346	2.341043	2.383495
8	2.28	2.2216	2.262218	2.252705	2.207107
9	5.56	5.7559	5.723883	5.558711	5.697835
10	5.99	6.2936	6.242509	5.78209	6.281862
11	5.96	6.3198	6.184353	6.092052	6.181304
12	5.52	5.5968	5.594026	5.725047	5.624758
13	2.03	2.1233	2.030067	1.968123	2.019488
14	1.37	1.4540	1.18019	1.108007	1.159557
15	1.29	1.1517	1.172965	1.01428	1.152621
16	1.29	1.1542	1.238328	1.237042	1.222301
17	1.29	1.2170	1.267971	1.227709	1.268734
18	1.29	1.3804	1.46207	1.545717	1.452348
19	1.41	1.3018	1.207868	1.247249	1.272971
20	3.42	3.6973	3.77923	3.974277	3.603474
21	1.35	1.0736	1.117087	1.234182	1.129767
22	1.49;	1.2266;	1.718564;	1.048043;	1.182025;
	1.15	1.1880	1.085838	1.677845	1.282698
23	0.90	0.8283	0.8499	0.927689	0.866745
24	0.86	1.0492	0.768271	0.721791	0.926324

Table S5. Results analyzed by the improved probability DP4$^+$ method based on the experimental NMR data of **1a** and calculated NMR data of 20*R*,21*S*-**1a**, 20*S*,21*S*-**1a**, 20*R*,21*R*-**1a**, and 20*S*,21*R*-**1a**

	20*R*,21*S*-**1a**	20*S*,21*S*-**1a**	20*R*,21*R*-**1a**	20*S*,21*R*-**1a**
sDP4$^+$ (^{1}H NMR data)	1.51 %	93.27 %	0.00 %	5.22 %
sDP4$^+$ (^{13}C NMR data)	0.05 %	98.80 %	0.60 %	0.55 %
sDP4$^+$ (all NMR data)	0.00 %	99.97 %	0.00 %	0.03 %
uDP4$^+$ (^{1}H NMR data)	75.51 %	5.13 %	0.00 %	19.36 %
uDP4$^+$ (^{13}C NMR data)	0.00 %	84.58 %	0.37 %	15.05 %
uDP4$^+$ (all NMR data)	0.00 %	59.82 %	0.00 %	40.18 %
DP4$^+$ (^{1}H NMR data)	16.47 %	68.95 %	0.00 %	14.58 %
DP4$^+$ (^{13}C NMR data)	0.00 %	99.90 %	0.00 %	0.10 %
DP4$^+$ (all NMR data)	0.00 %	99.98 %	0.00 %	0.02 %

Table S6. Crystal data and structure refinement for penidifarnesylin A (**6**)

Identification code	1750
Empirical formula	$C_{30}H_{50}O_4$
Formula weight	474.70
Temperature/K	100.00(12)
Crystal system	orthorhombic
Space group	$P2_12_12$
a/Å	7.4649(8)
b/Å	59.512(10)
c/Å	9.9356(16)
α/°	90
β/°	90
γ/°	90
Volume/Å^3	4413.9(12)
Z	6
ρ_{calc}g/cm^3	1.072
μ/mm^{-1}	0.536
F(000)	1572.0
Crystal size/mm^3	0.14 × 0.13 × 0.12
Radiation	Cu Kα (λ = 1.54184)
2Θ range for data collection/°	5.94 to 148.752
Index ranges	-9 ≤ h ≤ 6, -73 ≤ k ≤ 68, -12 ≤ l ≤ 12
Reflections collected	29803
Independent reflections	8796 [R_{int} = 0.1650, R_{sigma} = 0.1369]
Data/restraints/parameters	8796/62/479
Goodness-of-fit on F^2	1.030
Final R indexes [I>=2σ (I)]	R_1 = 0.0877, wR_2 = 0.1862
Final R indexes [all data]	R_1 = 0.1085, wR_2 = 0.2366
Largest diff. peak/hole / e Å^{-3}	0.55/-0.50
Flack parameter	0.3(3)

Table S7. Cartesian coordinates for the low-energy reoptimized MMFF conformers of 5*R*,8*R*,5′*R*,8′*R*-**6** at B3LYP/6-311+G (d,p) level of theory in MeOH

5*R*,8*R*,5′*R*,8′*R* -**6**			Standard Orientation (Ångstroms)		
Center number	Atomic number	Atomic type	X	Y	Z
1.	8.	0.	-8.737120	0.952671	-1.451051
2.	1.	0.	-8.676159	1.715035	-0.858544
3.	8.	0.	-4.776845	0.819918	1.204861
4.	1.	0.	-3.837042	0.665329	1.387112
5.	6.	0.	-7.761258	-0.711023	0.086253
6.	6.	0.	-5.282809	-0.387584	0.638002
7.	1.	0.	-5.414507	-1.142009	1.428627
8.	6.	0.	-6.602289	-0.046076	0.003317

9.	1.	0.	-6.566989	0.846388	-0.616513
10.	6.	0.	-4.288233	-0.946436	-0.418515
11.	1.	0.	-4.210471	-0.206292	-1.222217
12.	1.	0.	-4.720623	-1.856440	-0.852356
13.	6.	0.	-2.919497	-1.238765	0.164529
14.	6.	0.	-1.934167	-0.327331	0.057850
15.	1.	0.	-2.158468	0.577078	-0.511021
16.	6.	0.	-10.180420	0.069573	0.313488
17.	1.	0.	-9.840284	0.757395	1.097412
18.	1.	0.	-10.429490	-0.871108	0.823247
19.	6.	0.	-11.397330	0.591706	-0.400516
20.	1.	0.	-11.670100	0.027111	-1.293076
21.	6.	0.	-8.996212	-0.191866	-0.650377
22.	1.	0.	-9.310103	-0.964209	-1.368715
23.	6.	0.	-12.170880	1.638522	-0.076460
24.	6.	0.	-0.522034	-0.399196	0.573167
25.	1.	0.	-0.327278	0.472855	1.214042
26.	1.	0.	-0.361800	-1.280350	1.201128
27.	6.	0.	-13.368900	2.001445	-0.921192
28.	1.	0.	-13.485260	1.328962	-1.775275
29.	1.	0.	-14.294710	1.967241	-0.331362
30.	1.	0.	-13.284230	3.026774	-1.305086
31.	6.	0.	-11.958530	2.535375	1.118620
32.	1.	0.	-11.074480	2.278980	1.705349
33.	1.	0.	-11.864030	3.583075	0.804373
34.	1.	0.	-12.825450	2.493526	1.791230
35.	6.	0.	-7.966011	-1.994316	0.858170
36.	1.	0.	-8.573751	-1.836639	1.757472
37.	1.	0.	-8.498259	-2.733211	0.245666
38.	1.	0.	-7.024905	-2.447803	1.172807
39.	6.	0.	-2.791179	-2.563943	0.876670
40.	1.	0.	-2.949941	-3.389586	0.171171
41.	1.	0.	-1.818281	-2.708862	1.349008
42.	1.	0.	-3.557997	-2.671255	1.653868
43.	8.	0.	8.737196	0.952720	1.451030
44.	1.	0.	8.676309	1.715089	0.858523
45.	8.	0.	4.776812	0.820353	-1.204752
46.	1.	0.	3.837002	0.665801	-1.386997
47.	6.	0.	7.761180	-0.710876	-0.086282
48.	6.	0.	5.282764	-0.387215	-0.638035
49.	1.	0.	5.414413	-1.141565	-1.428741
50.	6.	0.	6.602282	-0.045803	-0.003369
51.	1.	0.	6.567066	0.846679	0.616437
52.	6.	0.	4.288205	-0.946145	0.418453
53.	1.	0.	4.210430	-0.206043	1.222195
54.	1.	0.	4.720601	-1.856170	0.852247

55.	6.	0.	2.919474	-1.238452	-0.164612
56.	6.	0.	1.934083	-0.327110	-0.057735
57.	1.	0.	2.158334	0.577202	0.511305
58.	6.	0.	10.180430	0.069487	-0.313495
59.	1.	0.	9.840370	0.757348	-1.097419
60.	1.	0.	10.429410	-0.871217	-0.823254
61.	6.	0.	11.397380	0.591499	0.400531
62.	1.	0.	11.670090	0.026858	1.293077
63.	6.	0.	8.996180	-0.191839	0.650357
64.	1.	0.	9.309997	-0.964210	1.368698
65.	6.	0.	12.171000	1.638270	0.076512
66.	6.	0.	0.521946	-0.398977	-0.573043
67.	1.	0.	0.327098	0.473240	-1.213662
68.	1.	0.	0.361796	-1.279963	-1.201262
69.	6.	0.	13.369050	2.001084	0.921262
70.	1.	0.	13.485360	1.328564	1.775321
71.	1.	0.	14.294850	1.966849	0.331434
72.	1.	0.	13.284430	3.026403	1.305197
73.	6.	0.	11.958720	2.535181	-1.118535
74.	1.	0.	11.074670	2.278838	-1.705292
75.	1.	0.	11.864250	3.582871	-0.804245
76.	1.	0.	12.825650	2.493327	-1.791126
77.	6.	0.	7.965786	-1.994220	-0.858155
78.	1.	0.	8.573503	-1.836636	-1.757489
79.	1.	0.	8.497987	-2.733140	-0.245643
80.	1.	0.	7.024625	-2.447632	-1.172737
81.	6.	0.	2.791219	-2.563532	-0.876953
82.	1.	0.	2.950030	-3.389269	-0.171575
83.	1.	0.	1.818324	-2.708437	-1.349301
84.	1.	0.	3.558036	-2.670692	-1.654174

Table S8. Cartesian coordinates for the low-energy reoptimized MMFF conformers of 5*S*,8*S*,5′*S*,8′*S*-**6** at B3LYP/6-311+G (d,p) level of theory in MeOH

6*S*,8*S*,6′*S*,8′*S*-**6**		Standard Orientation (Ångstroms)			
Center number	Atomic number	Atomic type	X	Y	Z
1.	8.	0.	8.737120	0.952671	-1.451051
2.	1.	0.	8.676159	1.715035	-0.858544
3.	8.	0.	4.776845	0.819918	1.204861
4.	1.	0.	3.837042	0.665329	1.387112
5.	6.	0.	7.761258	-0.711023	0.086253
6.	6.	0.	5.282809	-0.387584	0.638002
7.	1.	0.	5.414507	-1.142009	1.428627
8.	6.	0.	6.602289	-0.046076	0.003317
9.	1.	0.	6.566989	0.846388	-0.616513

10.	6.	0.	4.288233	-0.946436	-0.418515
11.	1.	0.	4.210471	-0.206292	-1.222217
12.	1.	0.	4.720623	-1.856440	-0.852356
13.	6.	0.	2.919497	-1.238765	0.164529
14.	6.	0.	1.934167	-0.327331	0.057850
15.	1.	0.	2.158468	0.577078	-0.511021
16.	6.	0.	10.180420	0.069573	0.313488
17.	1.	0.	9.840284	0.757395	1.097412
18.	1.	0.	10.429490	-0.871108	0.823247
19.	6.	0.	11.397330	0.591706	-0.400516
20.	1.	0.	11.670100	0.027111	-1.293076
21.	6.	0.	8.996212	-0.191866	-0.650377
22.	1.	0.	9.310103	-0.964209	-1.368715
23.	6.	0.	12.170880	1.638522	-0.076460
24.	6.	0.	0.522034	-0.399196	0.573167
25.	1.	0.	0.327278	0.472855	1.214042
26.	1.	0.	0.361800	-1.280350	1.201128
27.	6.	0.	13.368900	2.001445	-0.921192
28.	1.	0.	13.485260	1.328962	-1.775275
29.	1.	0.	14.294710	1.967241	-0.331362
30.	1.	0.	13.284230	3.026774	-1.305086
31.	6.	0.	11.958530	2.535375	1.118620
32.	1.	0.	11.074480	2.278980	1.705349
33.	1.	0.	11.864030	3.583075	0.804373
34.	1.	0.	12.825450	2.493526	1.791230
35.	6.	0.	7.966011	-1.994316	0.858170
36.	1.	0.	8.573751	-1.836639	1.757472
37.	1.	0.	8.498259	-2.733211	0.245666
38.	1.	0.	7.024905	-2.447803	1.172807
39.	6.	0.	2.791179	-2.563943	0.876670
40.	1.	0.	2.949941	-3.389586	0.171171
41.	1.	0.	1.818281	-2.708862	1.349008
42.	1.	0.	3.557997	-2.671255	1.653868
43.	8.	0.	-8.737196	0.952720	1.451030
44.	1.	0.	-8.676309	1.715089	0.858523
45.	8.	0.	-4.776812	0.820353	-1.204752
46.	1.	0.	-3.837002	0.665801	-1.386997
47.	6.	0.	-7.761180	-0.710876	-0.086282
48.	6.	0.	-5.282764	-0.387215	-0.638035
49.	1.	0.	-5.414413	-1.141565	-1.428741
50.	6.	0.	-6.602282	-0.045803	-0.003369
51.	1.	0.	-6.567066	0.846679	0.616437
52.	6.	0.	-4.288205	-0.946145	0.418453
53.	1.	0.	-4.210430	-0.206043	1.222195

54.	1.	0.	-4.720601	-1.856170	0.852247
55.	6.	0.	-2.919474	-1.238452	-0.164612
56.	6.	0.	-1.934083	-0.327110	-0.057735
57.	1.	0.	-2.158334	0.577202	0.511305
58.	6.	0.	-10.180430	0.069487	-0.313495
59.	1.	0.	-9.840370	0.757348	-1.097419
60.	1.	0.	-10.429410	-0.871217	-0.823254
61.	6.	0.	-11.397380	0.591499	0.400531
62.	1.	0.	-11.670090	0.026858	1.293077
63.	6.	0.	-8.996180	-0.191839	0.650357
64.	1.	0.	-9.309997	-0.964210	1.368698
65.	6.	0.	-12.171000	1.638270	0.076512
66.	6.	0.	-0.521946	-0.398977	-0.573043
67.	1.	0.	-0.327098	0.473240	-1.213662
68.	1.	0.	-0.361796	-1.279963	-1.201262
69.	6.	0.	-13.369050	2.001084	0.921262
70.	1.	0.	-13.485360	1.328564	1.775321
71.	1.	0.	-14.294850	1.966849	0.331434
72.	1.	0.	-13.284430	3.026403	1.305197
73.	6.	0.	-11.958720	2.535181	-1.118535
74.	1.	0.	-11.074670	2.278838	-1.705292
75.	1.	0.	-11.864250	3.582871	-0.804245
76.	1.	0.	-12.825650	2.493327	-1.791126
77.	6.	0.	-7.965786	-1.994220	-0.858155
78.	1.	0.	-8.573503	-1.836636	-1.757489
79.	1.	0.	-8.497987	-2.733140	-0.245643
80.	1.	0.	-7.024625	-2.447632	-1.172737
81.	6.	0.	-2.791219	-2.563532	-0.876953
82.	1.	0.	-2.950030	-3.389269	-0.171575
83.	1.	0.	-1.818324	-2.708437	-1.349301
84.	1.	0.	-3.558036	-2.670692	-1.654174

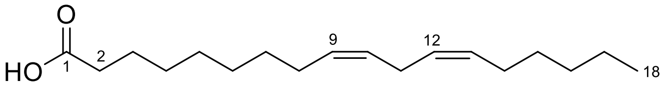

4a

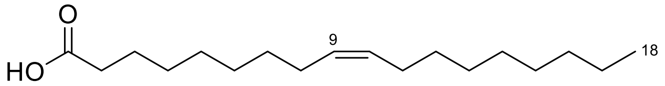

5a

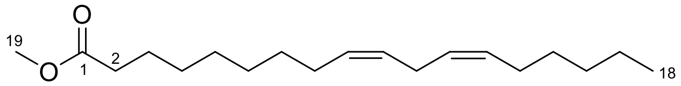

8

Table S9. ^{13}C NMR (150 MHz) and ^{1}H NMR (600 MHz) data of compounds **4a**, **5a**, and **8** (in DMSO-d_6)

No.	4a		5a		8	
	δ_C, type	δ_H, J in Hz	δ_C, type	δ_H, J in Hz	δ_C, type	δ_H, J in Hz
1	174.3, C	–	174.3, C	–	173.3, C	–
2	33.7, CH$_2$	2.14, t (7.5)	33.7, CH$_2$	2.14, t (7.4)	33.2, CH$_2$	2.27, t (7.5)
3	24.6, CH$_2$	1.47, m	24.5, CH$_2$	1.46, m	24.4, CH$_2$	1.49, m
4	28.7[a], CH$_2$	1.23–1.32[e], m	28.7[a], CH$_2$	1.23–1.20[e], m	28.4[a], CH$_2$	1.23–1.34[e], m
5	28.7[a], CH$_2$	1.23–1.32[e], m	28.7[a], CH$_2$	1.23–1.20[e], m	28.5[a], CH$_2$	1.23–1.34[e], m
6	28.8[a], CH$_2$	1.23–1.32[e], m	29.1[a], CH$_2$	1.23–1.20[e], m	28.5[a], CH$_2$	1.23–1.34[e], m
7	29.1[a], CH$_2$	1.23–1.32[e], m	29.2[a], CH$_2$	1.23–1.20[e], m	28.9[a], CH$_2$	1.23–1.34[e], m
8	26.7[b], CH$_2$	1.98[f], q (7.1)	26.6[b], CH$_2$	1.95[f], q (6.5)	26.6[b], CH$_2$	2.00[f], q (6.9)
9	129.6[c], CH	5.25–5.34[g], m	129.5[c], CH	5.27–5.32[g], m	129.7[c], CH	5.28–5.36[g], m
10	127.7[d], CH	5.25–5.34[g], m	129.5[c], CH	5.27–5.32[g], m	127.7[d], CH	5.28–5.36[g], m
11	25.2, CH$_2$	2.71, t (6.8)	26.6[b], CH$_2$	1.95[f], q (6.5)	25.1, CH$_2$	2.72, t (6.5)
12	127.7[d], CH	5.25–5.34[g], m	29.0[a], CH$_2$	1.23–1.20[e], m	127.7[d], CH	5.28–5.36[g], m
13	129.5[c], CH	5.25–5.34[g], m	28.7[a], CH$_2$	1.23–1.20[e], m	129.7[c], CH	5.28–5.36[g], m
14	26.7[b], CH$_2$	1.98[f], q (7.1)	28.6[a], CH$_2$	1.23–1.20[e], m	26.6[b], CH$_2$	2.00[f], q (6.9)
15	28.8[a], CH$_2$	1.23–1.32[e], m	28.8[a], CH$_2$	1.23–1.20[e], m	28.7[a], CH$_2$	1.23–1.34[e], m
16	31.0, CH$_2$	1.23–1.32[e], m	31.4, CH$_2$	1.23–1.20[e], m	30.9, CH$_2$	1.23–1.34[e], m
17	22.1, CH$_2$	1.23–1.32[e], m	22.1, CH$_2$	1.23–1.20[e], m	22.0, CH$_2$	1.23–1.34[e], m
18	13.8, CH$_3$	0.83, t (7.3)	13.8, CH$_3$	0.83, t (7.0)	13.9, CH$_3$	0.84, t (7.4)
19	–	–	–	–	51.1, CH$_3$	3.57, s
OH-1	–	11.93, s	–	11.93, s	–	–

[a,b,c,d] The data with the same label in each column may be interchanged; [e,f,g] The data with the same label in each column were overlapped.

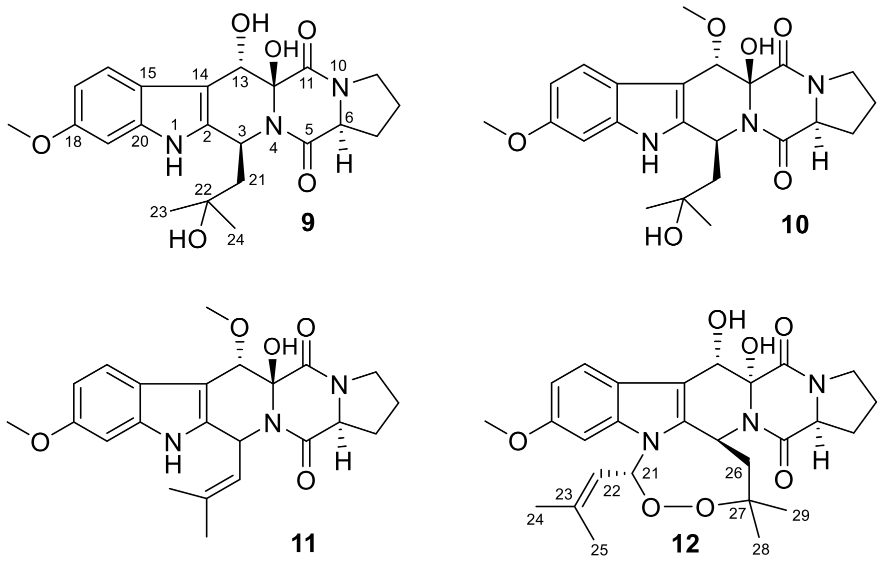

Table S10. ^{13}C NMR data of compounds **9**–**12** (150 MHz)

No.	**9**[a]	**10**[a]	**11**[b]	**12**[a]
2	134.5, C	135.5, C	133.9, C	130.9, C
3	46.6, CH	46.5, CH	49.3, C	50.6, CH
5	166.6, C	166.6, C	167.2, C	165.9, C
6	58.9, CH	58.8, CH	60.2, CH	58.3, CH
7	29.7, CH_2	29.7, CH_2	29.9, CH_2	28.9, CH_2
8	21.2, CH_2	21.2, CH_2	22.3, CH_2	22.1, CH_2
9	44.6, CH_2	44.7, CH_2	46.0, CH_2	47.8, CH_2
11	165.6, C	165.1, C	166.1, C	170.6, C
12	87.0, C	85.3, C	84.8, C	82.7, C
13	66.9, CH	75.9, CH	77.2, CH	67.8, CH
OCH_3-13	–	56.4, CH_3	56.8, CH_3	–
14	107.0, C	103.9, C	105.5, C	107.3, C
15	120.9, C	122.0, C	123.8, C	120.7, C
16	118.1, CH	118.2, CH	118.8, CH	121.3, CH
17	108.5, CH	109.1, CH	110.2, CH	108.9, CH
18	155.3, C	155.3, C	156.7, C	155.5, C
OCH_3-18	55.2, CH_3	55.3, CH_3	56.0, CH_3	55.3, CH_3
19	95.0, CH	95.2, CH	95.4, CH	93.6, CH
20	136.3, C	136.2, C	136.8, C	135.8, C
21	49.3, CH_2	49.6, CH_2	123.8, CH	85.1, CH
22	69.2, C	69.2, C	138.1, C	118.3, CH
23	31.2, CH_3	31.2, CH_3	26.3, CH_3	142.6, C
24	28.5, CH_3	28.4, CH_3	18.5, CH_3	18.5, CH_3
25				24.1, CH_3
26				45.1, CH_2
27				81.6, C
28				26.7, CH_3
29				25.2, CH_3

[a] Data were recorded in DMSO-d_6; [b] Data were recorded in $CDCl_3$.

Table S11. ^{1}H NMR data of compounds **9–12** (600 MHz, J in Hz)

No.	**9**[a]	**10**[a]	**11**[b]	**12**[a]
1	10.52, 1H, s	10.70, 1H, s	7.87, 1H, s	–
3	5.87, 1H, t (5.8)	5.88, 1H, t (5.9)	6.64, 1H, d (9.9)	5.86, 1H, d (10.0)
6	4.18, 1H, dd (10.6, 6.0)	4.14, 1H, dd (12.2, 6.6)	4.39, 1H, dd (10.6, 6.5)	4.43, 1H, dd (9.1, 6.9)
7	a: 2.28, 1H, m;<br>b: 1.95, 1H, m	a: 2.27, 1H, m;<br>b: 1.96, 1H, m	a: 2.49, 1H, m;<br>b: 2.12, 1H, m	a: 2.30, 1H, m;<br>b: 1.97, 1H, m
8	a: 1.88, 1H, m;<br>b: 1.70, 1H, m	a: 1.90, 1H, m;<br>b: 1.71, 1H, m	a: 2.03, 1H, m;<br>b: 2.00, 1H, m	1.89–1.96[c], 2H, m
9	a: 3.60, 1H, m;<br>b: 3.41, 1H, m	a: 3.60, 1H, m;<br>b: 3.44, 1H, m	a: 3.75, 1H, m;<br>b: 3.69, 1H, m	a: 3.49, 1H, m;<br>b: 3.44, 1H, m
OH-12	4.11, 1H, br s	4.89, 1H, br s	–	4.10, 1H, br s
13	4.80, 1H, s	4.64, 1H, s	4.75, 1H, s	5.41, 1H, s
OCH_3-13	–	3.26, 3H, s	3.38, 3H, s	–
OH-13	5.54, 1H, br s	–	–	6.26, 1H, br s
16	7.31, 1H, d (8.3)	7.43, 1H, d (8.5)	7.45, 1H, d (8.5)	7.72, 1H, d (8.3)
17	6.65, 1H, t (8.3)	6.68, 1H, dd (8.5, 2.8)	6.64, 1H, dd (8.5, 2.9)	6.69, 1H, dd (8.3, 2.6)
OCH_3-18	3.75, 3H, s	3.76, 3H, s	3.85, 3H, s	3.76, 3H, s
19	6.94, 1H, s	6.98, 1H, d (2.8)	6.90, 1H, d (2.9)	6.76, 1H, d (2.6)
21	a: 2.12, 1H, dd (15.1, 5.8);<br>b: 2.02, 1H, dd (15.1, 5.8)	a: 2.08, 1H, dd (15.0, 5.9);<br>b: 2.02, 1H, dd (15.0, 5.9)	5.57, 1H, d (9.9)	5.0, 1H, d (8.0)
22	–	–	–	6.81, 1H, d (8.0)
23	1.19, 3H, s	1.19, 3H, s	1.80, 3H, s	–
24	1.32, 3H, s	1.33, 3H, s	2.07, 3H, s	1.70, 3H, s
25	–	–	–	1.99, 3H, s
26	–	–	–	1.89–1.96[c], 2H, m
28	–	–	–	0.95, 3H, s
29	–	–	–	1.57, 3H, s

[a] Data were recorded in DMSO-d_6; [b] Data were recorded in $CDCl_3$; [c] Data with the same label in each column were overlapped.

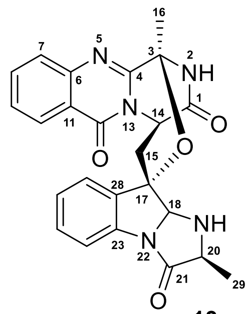

13

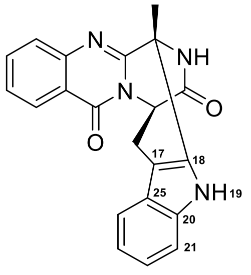

14

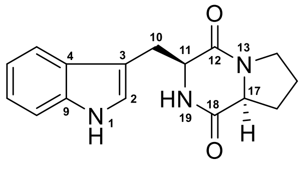

15

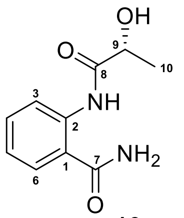

16

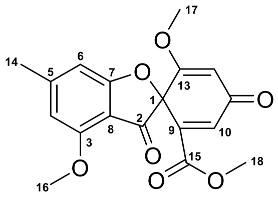

17

Table S12. ^{13}C NMR data of compounds **13**–**17** (150 MHz, in DMSO-d_6)

No.	**13**	**14**	**15**	**16**	**17**
1	172.3, C	169.1, C	–	121.3, C	83.6, C
2	–	–	124.4, CH	139.1, C	189.2, C
3	83.8, C	54.6, C	109.3, C	122.8, CH	157.9, C
4	150.3, C	154.3, C	127.3, C	132.2, CH	105.3, CH
5	–	–	118.6, CH	120.2, CH	152.4, C
6	146.3, C	146.6, C	118.1, CH	128.9, CH	105.9, CH
7	128.0, CH	127.3, CH	120.9, CH	170.6, C	173.6, C
8	134.6, CH	134.8, CH	111.2, CH	174.5, C	107.3, C
9	128.1, CH	127.4, CH	136.0, C	68.3, CH	138.3, C
10	126.1, CH	126.4, CH	25.8, CH_2	21.3, CH_3	135.7, CH
11	121.1, C	119.4, C	55.2, CH		184.9, C
12	159.3, C	159.3, C	165.5, C		103.8, CH
13	–	–	–		169.0, C
14	51.1, CH	54.1, CH	44.6, CH_2		22.5, CH_3
15	31.2, CH_2	25.6, CH_2	21.9, CH_2		163.1, C
16	23.9, CH_3	18.3, CH_3	27.7, CH_2		56.0, CH_3
17	87.0, C	105.5, C	58.4, CH		57.4, CH_3
18	85.6, CH	134.0, C	169.0, C		52.9, CH_3
20	58.1, CH	134.8, C			
21	170.0, C	111.7, CH			
22	–	122.3, CH			
23	136.2, C	120.0, CH			
24	114.5, CH	118.1, CH			
25	129.9, CH	127.4, C			
26	125.5, CH				
27	125.1, CH				
28	138.3, C				
29	17.5, CH_3				

Table S13. ^{1}H NMR data of compounds **13**–**17** (600 MHz, J in Hz, in DMSO-d_6)

No.	**13**	**14**	**15**	**16**	**17**
NH-1	–	–	10.86, 1H, s	–	–
2	9.97, 1H, s	9.59, 1H, s	7.17, 1H, d (1.8)	–	–
NH-2	–	–	–	12.0, 1H, s	–
3	–	–	–	8.56, 1H, dd (8.3, 1.7)	–
4	–	–	–	7.46, 1H, td (8.3, 1.7)	6.62, 1H, s
5	–	–	7.56, 1H, d (7.9)	7.10, 1H, td (8.3, 1.7)	–
6	–	–	6.95, 1H, t (7.9)	7.73, 1H, td (8.3, 1.7)	6.69, 1H, s
7	7.78, 1H, d (8.0)	7.63, 1H, d (8.0)	7.04, 1H, t (7.9)	–	–
NH_2-7	–	–	–	8.17, 1H, s; 7.60, 1H, s	–
8	7.87, 1H, t (8.0)	7.79, 1H, t (8.0)	7.32, 1H, d (7.9)	–	–
9	7.62, 1H, t (8.0)	7.51, 1H, t (8.0)	–	4.08, 1H, d (6.4)	–
OH-9	–	–	–	5.99, br s	–
10	8.19, 1H, d (8.0)	8.13, 1H, d (8.0)	a: 3.22^a, 1H, m; b: 3.05, 1H, dd (14.8, 5.6)	1.29, 3H, d (6.4)	6.96, 1H, d (1.8)
11	–	–	4.29, 1H, t 5.6)		–
12	–	–	–		5.98, 1H, d (1.8)
14	5.36, 1H, d (47.3)	5.70, 1H, d (4.6)	a: 3.37, 1H, m; b: 3.25^a, 1H, m		2.41, 3H, s
15	a: 2.91, 1H, dd (15.1, 7.3); b: 2.04, 1H, d (15.1)	a: 3.41, 1H, dd (17.1, 2.8); b: 3.21, 1H, dd (17.1, 4.6)	a: 1.61, 1H, m; b: 1.36, 1H, m		–
16	1.88, 3H, s	2.12, 3H, s	a: 1.95, 1H, m; b: 1.68, 1H, m		3.88, 3H, s
17	–	–	4.05, 1H, dd (8.9, 7.5)		3.64, 3H, s
18	5.07, 1H, d (9.0)	–	7.75, 1H, s		3.67, 3H, s
19	2.33, 1H, t (9.0)	11.24, 1H, s			
20	3.49, 1H, m	–			
21	–	7.36, 1H, d (8.0)			
22	–	7.10, 1H, t (8.0)			
23	–	6.97, 1H, t (8.0)			
24	7.33, 1H, d (7.5)	7.39, 1H, d (8.0)			
25	7.34, 1H, t (7.5)				
26	7.20, 1H, t (7.5)				
27	7.30, 1H, d (7.5)				
29	0.81, 3H, d (7.5)				

^{a} Data with the same label in each column were overlapped.

Figure S1. The colonies of *Penicillium* sp. ZZ1750 in PDA medium (**A**) and the state of *Penicillium* sp. ZZ1750 in PDB medium (**B**)

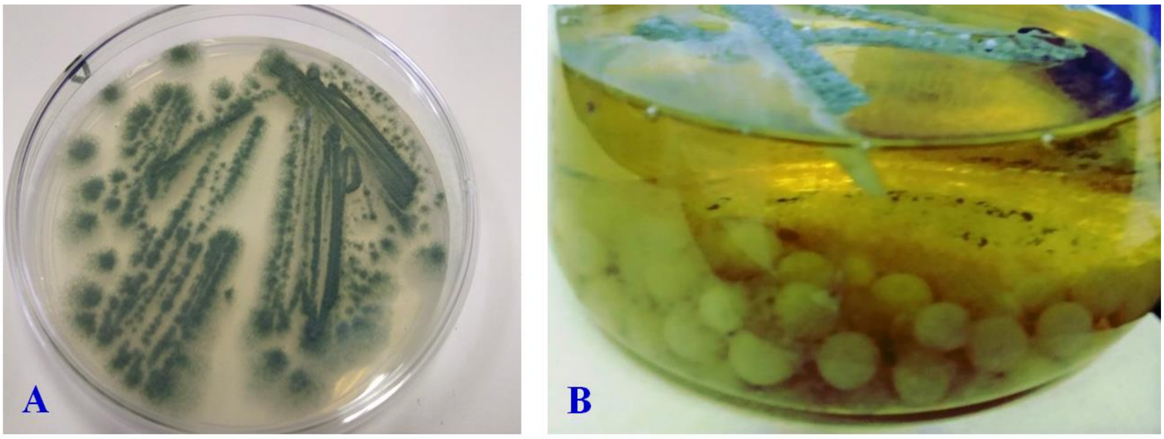

Figure S2. ITS rDNA sequence of *Penicillium* sp. ZZ1750

GAACCTGCGGAAGGATCATTACCGAGTGAGGGCCCTCTGGGTCCAACCTCCCA
CCCGTGTTTATTTTACCTTGTTGCTTCGGCGGGCCCGCCTTAACTGGCCGCCGG
GGGGCTTACGCCCCCGGGCCCGCGCCCGCCGAAGACACCCTCGAACTCTGTCT
GAAGATTGTAGTCTGAGTGAAAATATAAATTATTTAAAACTTTCAACAACGGAT
CTCTTGGTTCCGGCATCGATGAAGAACGCAGCGAAATGCGATACGTAATGTGAA
TTGCAAATTCAGTGAATCATCGAGTCTTTGAACGCACATTGCGCCCCCTGGTAT
TCCGGGGGGCATGCCTGTCCGAGCGTCATTTCTGCCCTCAAGCACGGCTTGTGT
GTTGGGCCCCGTCCTCCGATCCCGGGGGACGGGCCCGAAAGGCAGCGGCGGC
ACCGCGTCCGGTCCTCGAGCGTATGGGGCTTTGTCACCCGCTCTGTAGGCCCGG
CCGGCGCTTGCCGATCAACCCAAATTTTTATCCAGGTTGACCTCGGATCAGGTA
GGGATACCCGCTGAACTTAAGCATATC (563 bp).

Figure S3. GC analytic results of aldonitrile acetates of sugars

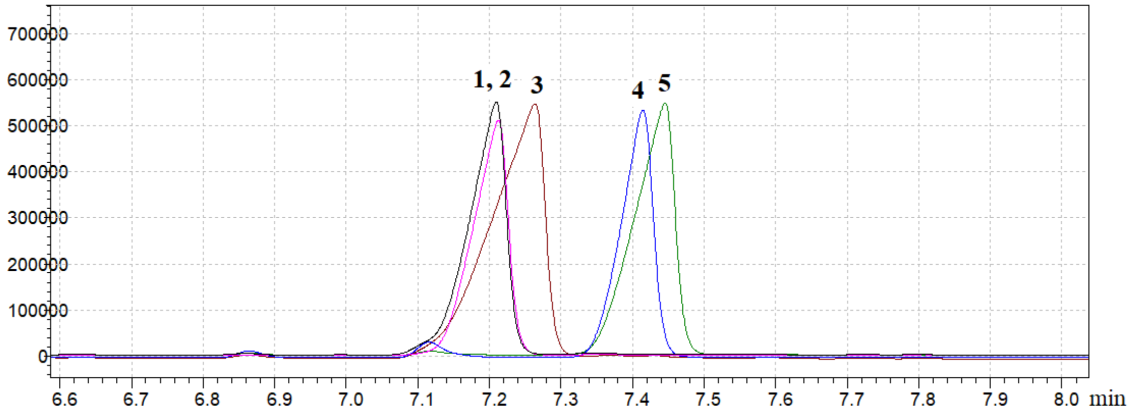

1: aldonitrile acetate of hydrolytic D-glucose; **2**: aldonitrile acetate of standard D-glucose, t_R 7.20 min; **3**: aldonitrile acetate of standard L-glucose, t_R 7.28 min; **4**: aldonitrile acetate of standard D-galactose, t_R 7.40 min; **5**: aldonitrile acetate of standard L-galactose, t_R 7.45 min).

Figure S4. COSY and key HMBC correlations of peniresorcinosides C–E (**3**–**5**)

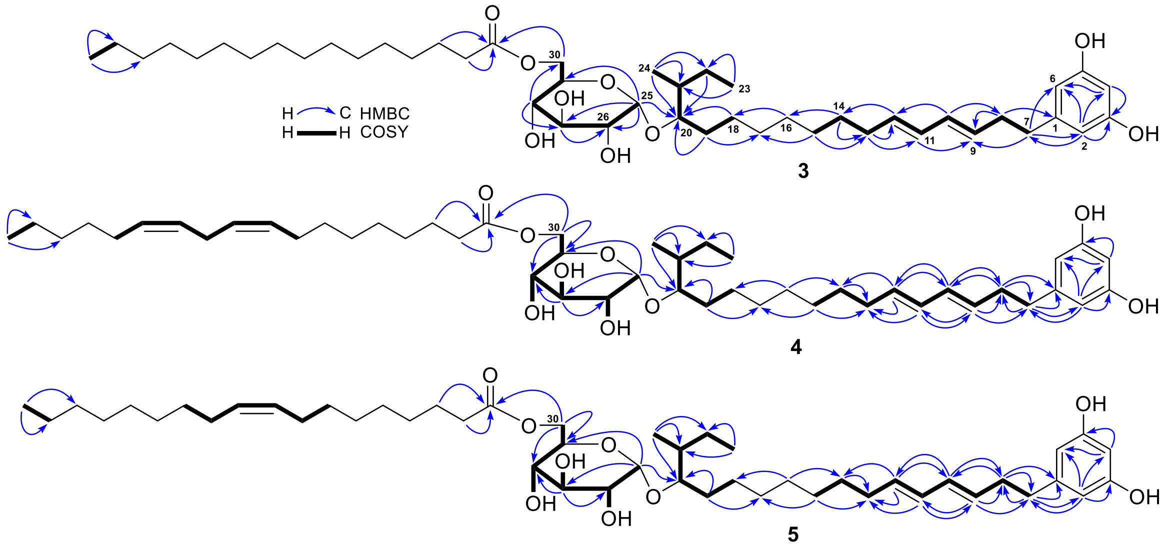

Figure S5. Chromatogram of co-HPLC analysis of hydrolytic peniresorcinoside A with standard peniresorcinoside A (**1**)

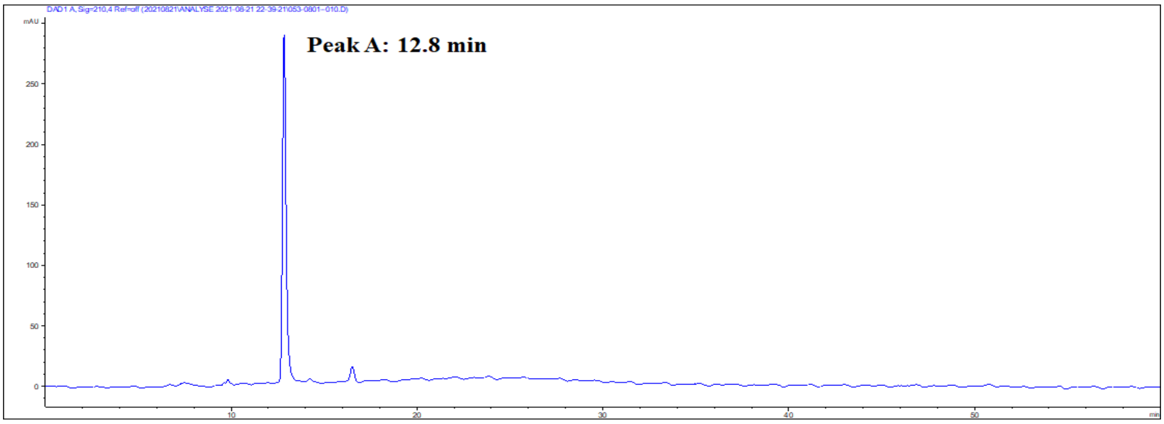

Peak A: the hydrolytic peniresorcinoside A and standard peniresorcinoside A (**1**); Zorbax SB-C_{18} column: 250 × 9.4 mm, 5 µm; mobile phase: MeOH/0.1%TFA in water (93/7); flow rate:1.0 mL/min; UV detection: 210 nm.

Figure S6. Chromatogram of co-HPLC analysis of compound **4a** with standard linoleic acid

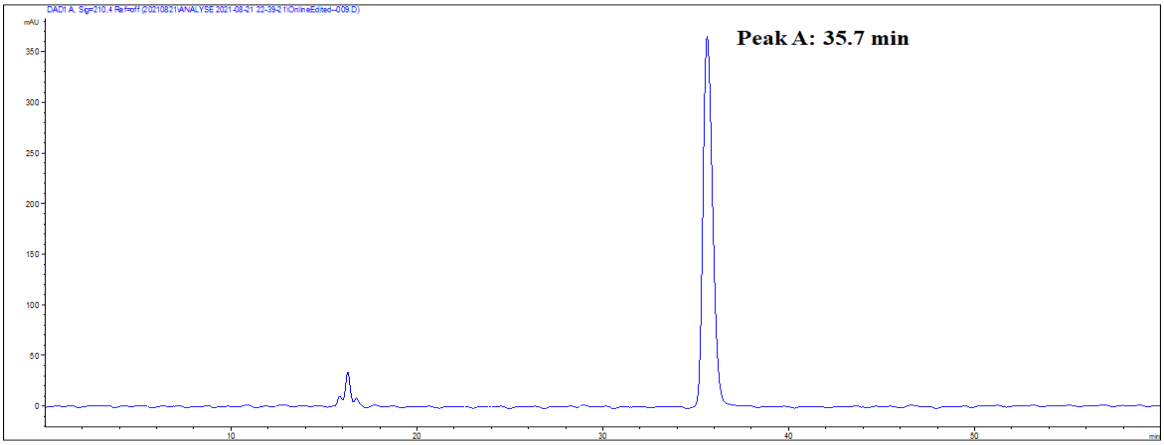

Peak A: Compound **4a** and linoleic acid; Zorbax SB-C_{18} column: 250 × 9.4 mm, 5 µm; mobile phase: MeOH/0.1%TFA in water (93/7); flow rate:1.0 mL/min; UV detection: 210 nm.

Figure S7. Chromatogram of co-HPLC analysis of compound **5a** with standard oleic acid

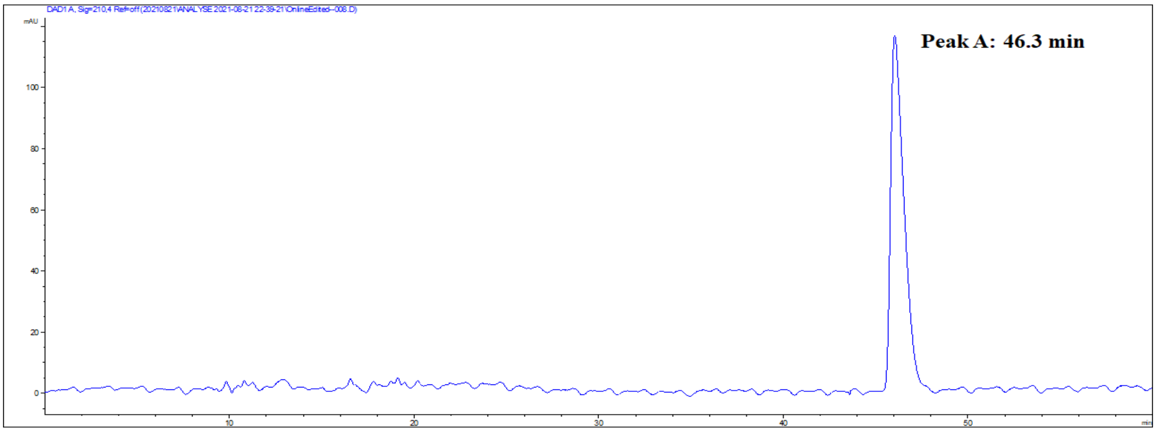

Peak A: Compound **5a** and oleic acid; Zorbax SB-C_{18} column: 250 × 9.4 mm, 5 µm; mobile phase: MeOH/0.1%TFA in water (93/7); flow rate:1.0 mL/min; UV detection: 210 nm.

Figure S8. ^{1}H NMR spectrum of peniresorcinoside A (**1**, 600 MHz, in DMSO-d_6)

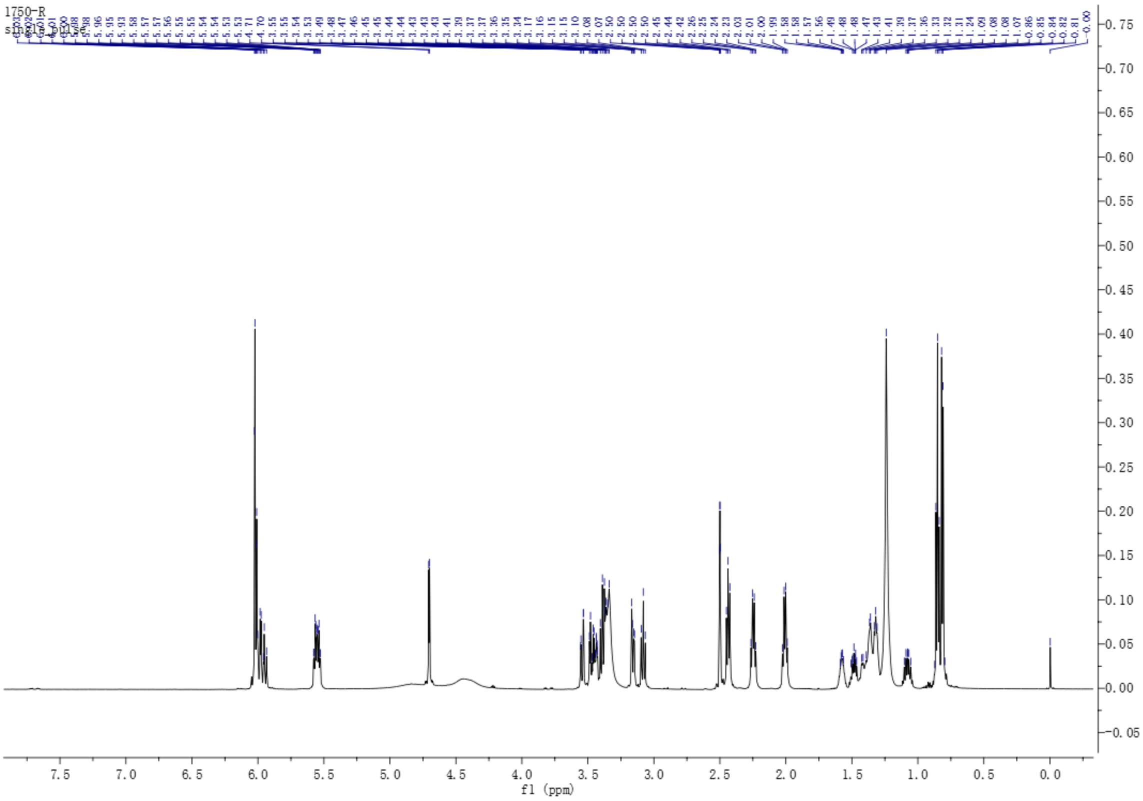

Figure S9. ^{1}H NMR spectrum of peniresorcinoside A (**1**, 600 MHz, in DMSO-d_6)

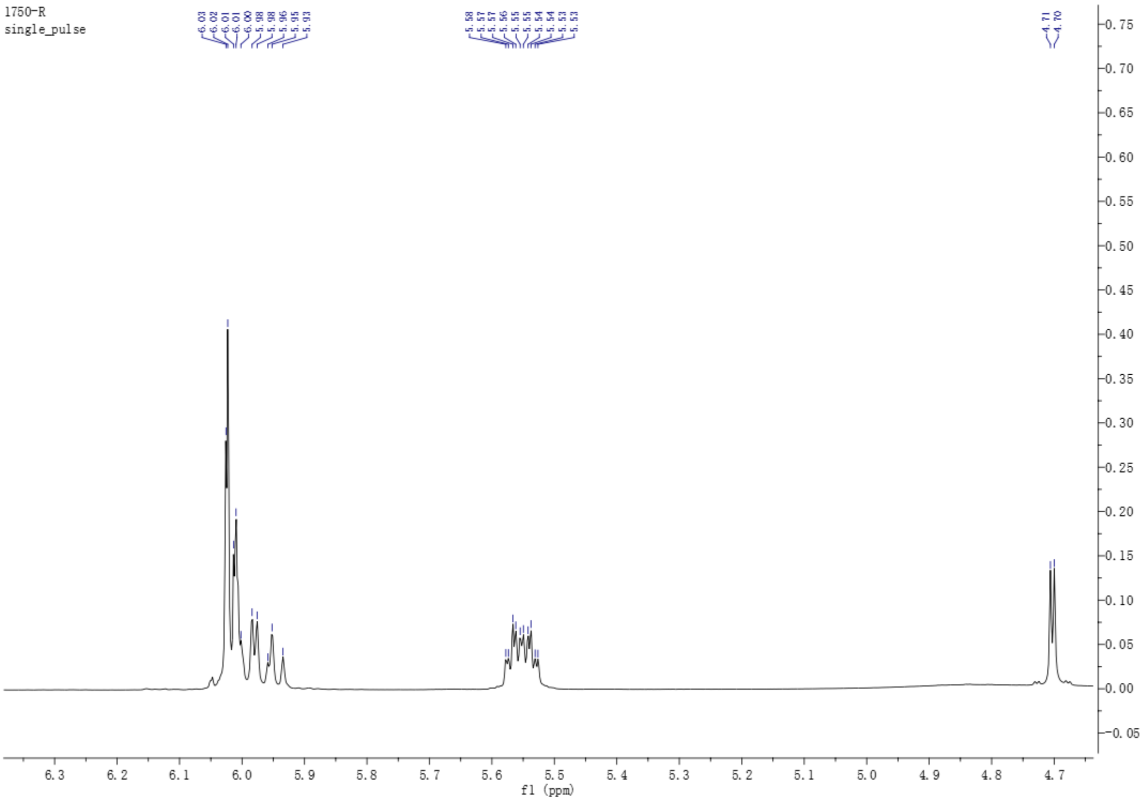

Figure S10. ^{1}H NMR spectrum of peniresorcinoside A (**1**, 600 MHz, in DMSO-d_6)

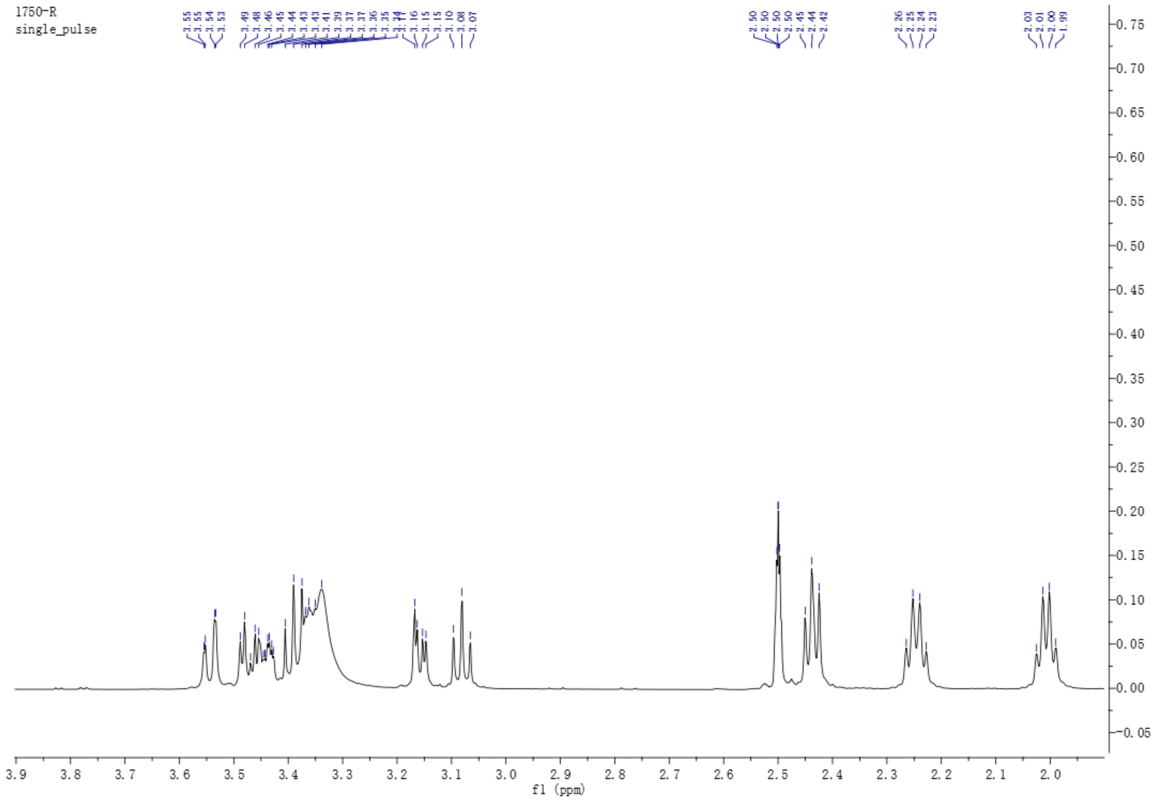

Figure S11. ^{1}H NMR spectrum of peniresorcinoside A (**1**, 600 MHz, in DMSO-d_6)

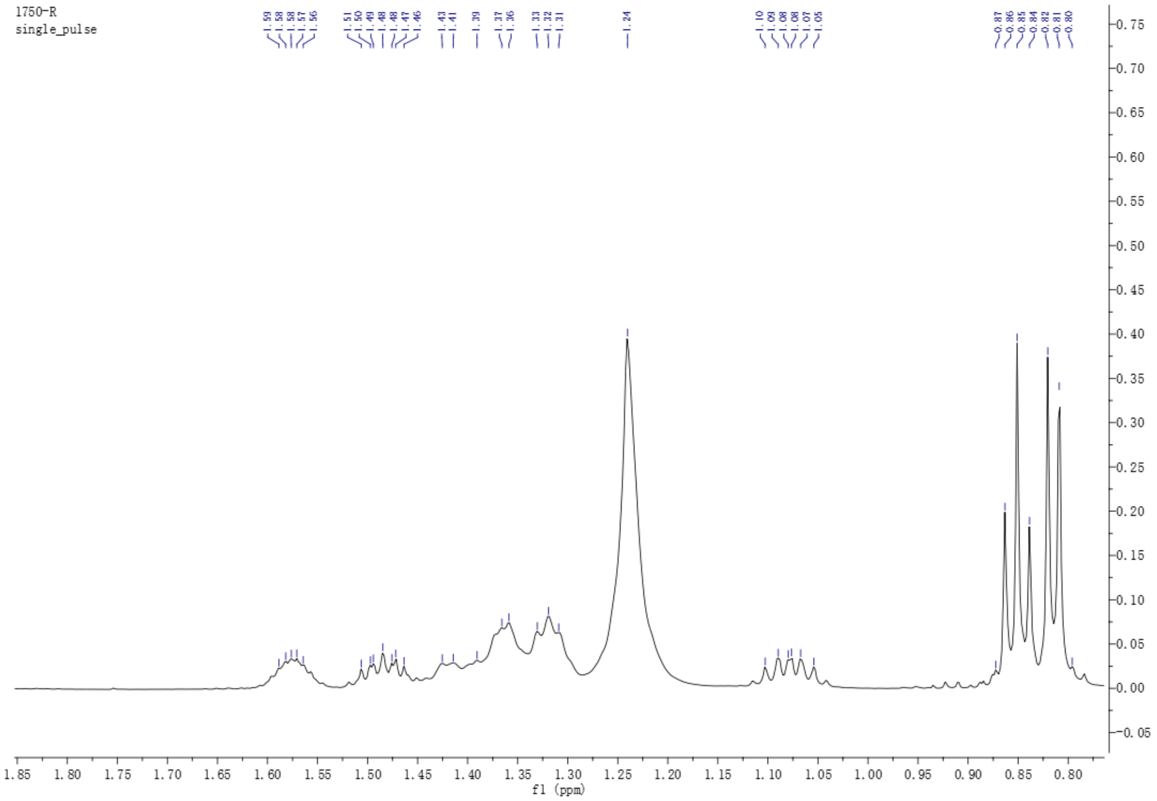

Figure S12. ^{13}C NMR spectrum of peniresorcinoside A (**1**, 150 MHz, in DMSO-d_6)

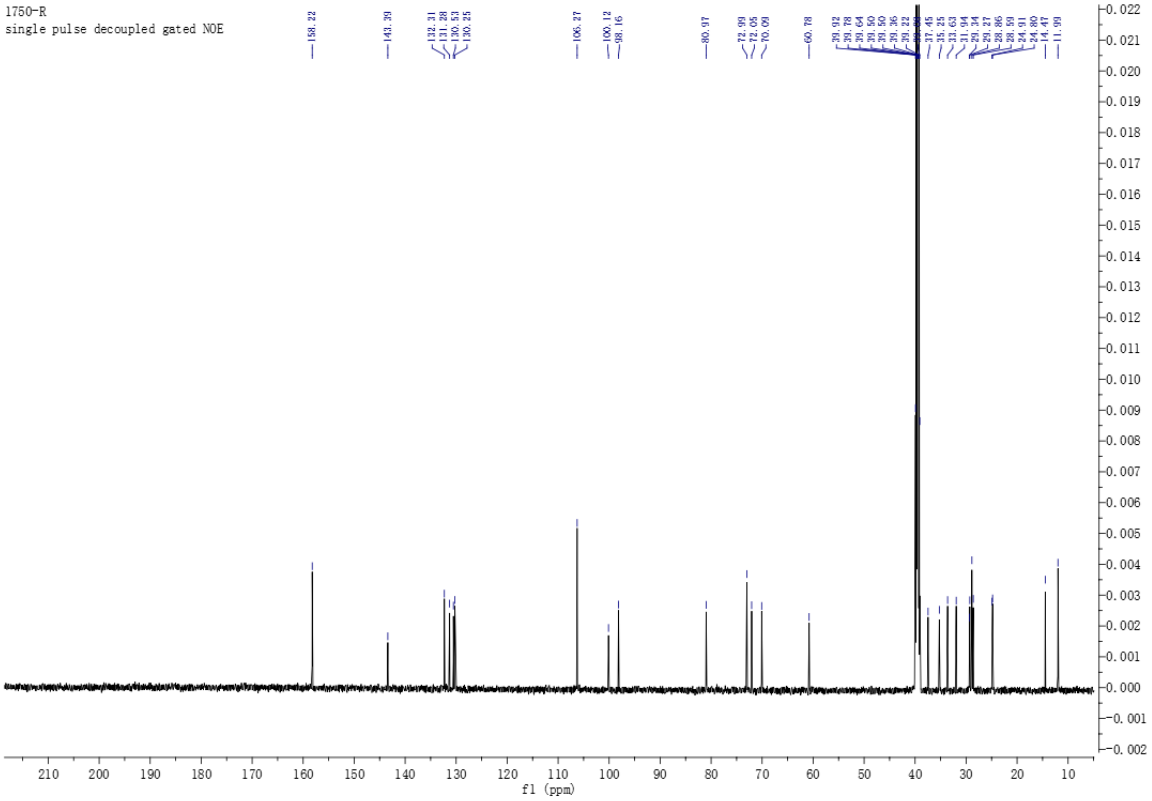

Figure S13. ^{13}C NMR spectrum of peniresorcinoside A (**1**, 150 MHz, in DMSO-d_6)

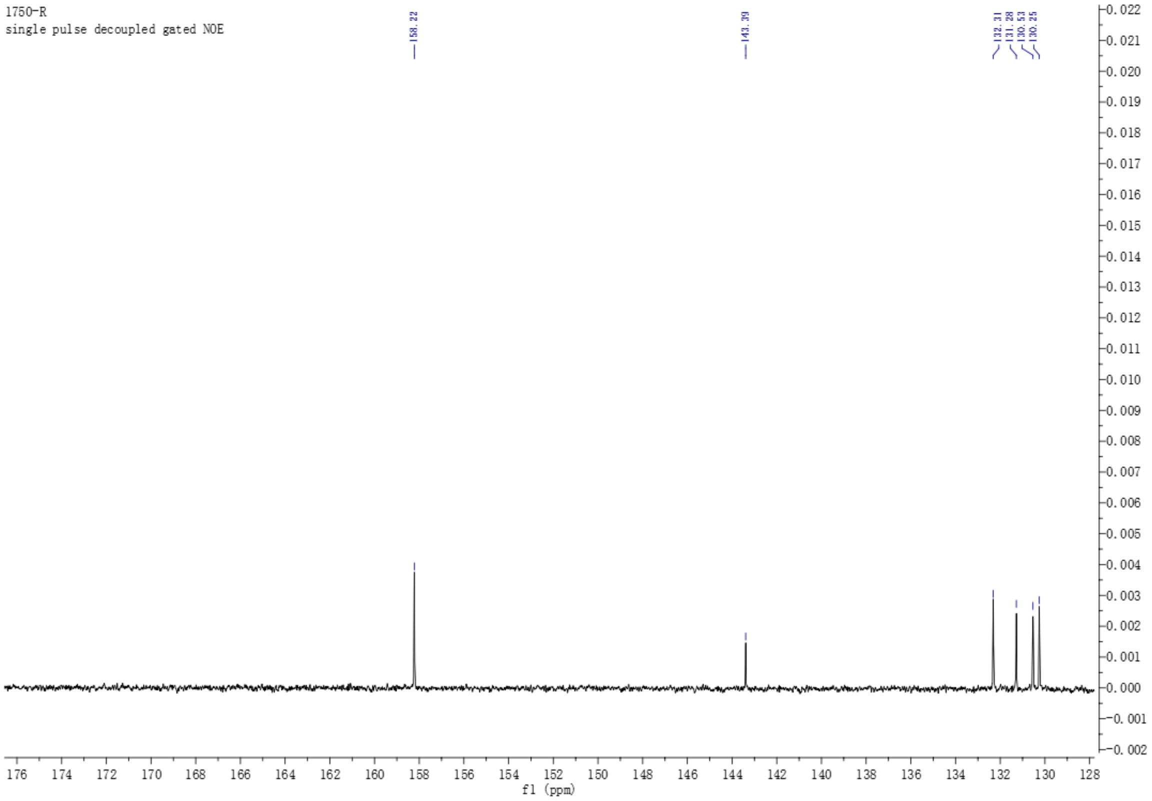

Figure S14. ^{13}C NMR spectrum of peniresorcinoside A (**1**, 150 MHz, in DMSO-d_6)

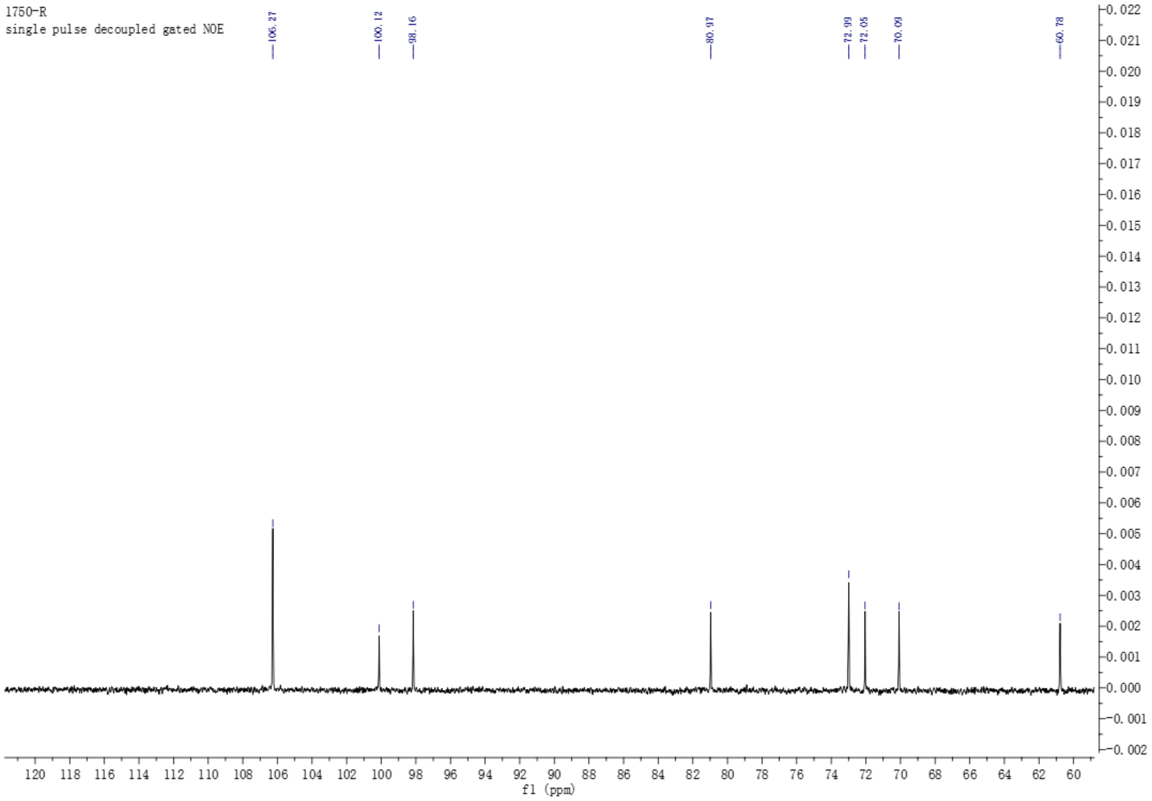

Figure S15. ^{13}C NMR spectrum of peniresorcinoside A (**1**, 150 MHz, in DMSO-d_6)

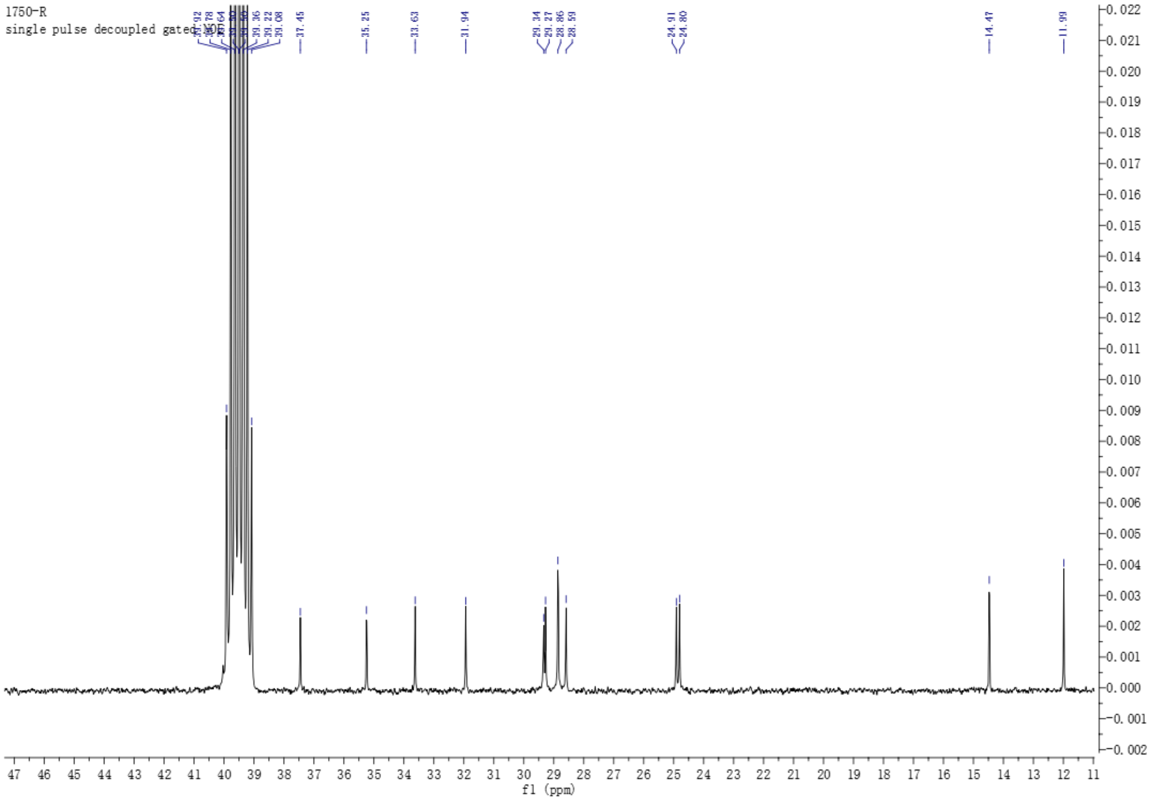

Figure S16. DEPT spectrum of peniresorcinoside A (**1**, in DMSO-d_6)

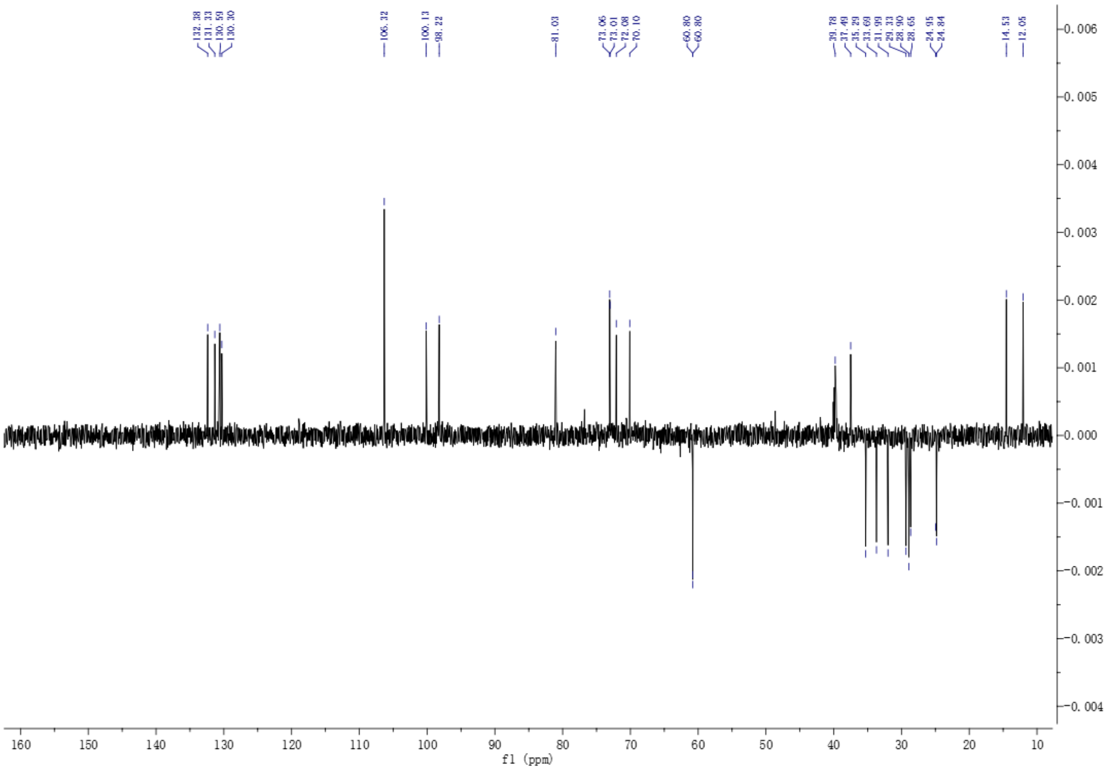

Figure S17. DEPT spectrum of peniresorcinoside A (**1**, in DMSO-d_6)

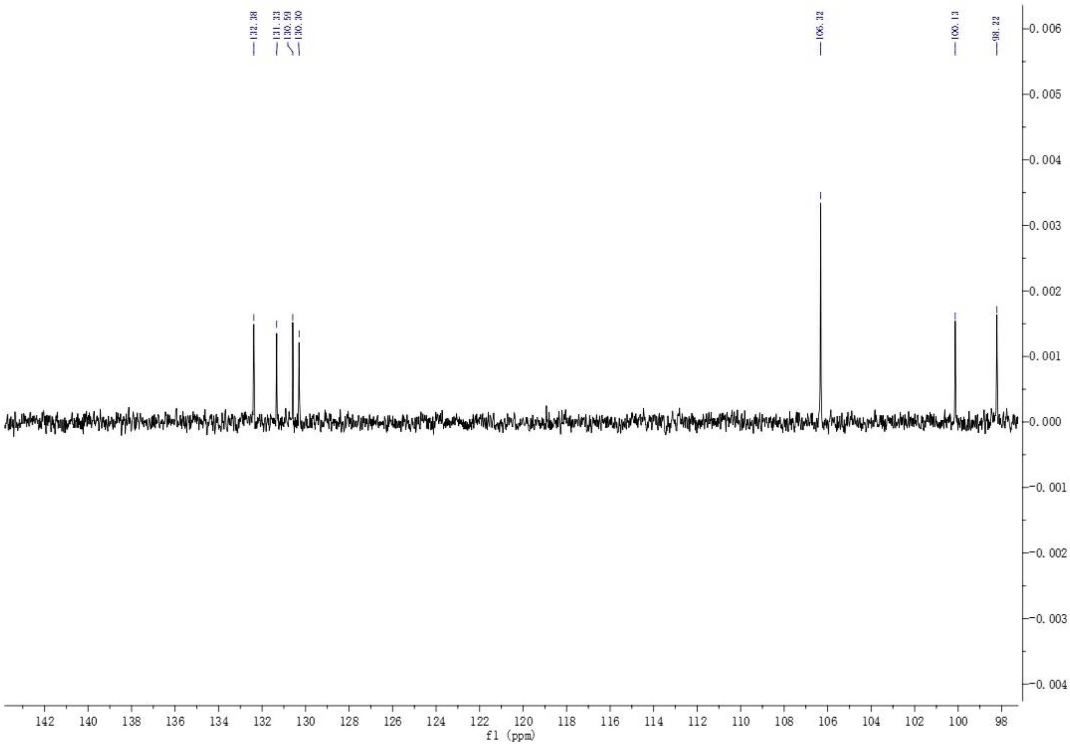

Figure S18. DEPT spectrum of peniresorcinoside A (**1**, in DMSO-d_6)

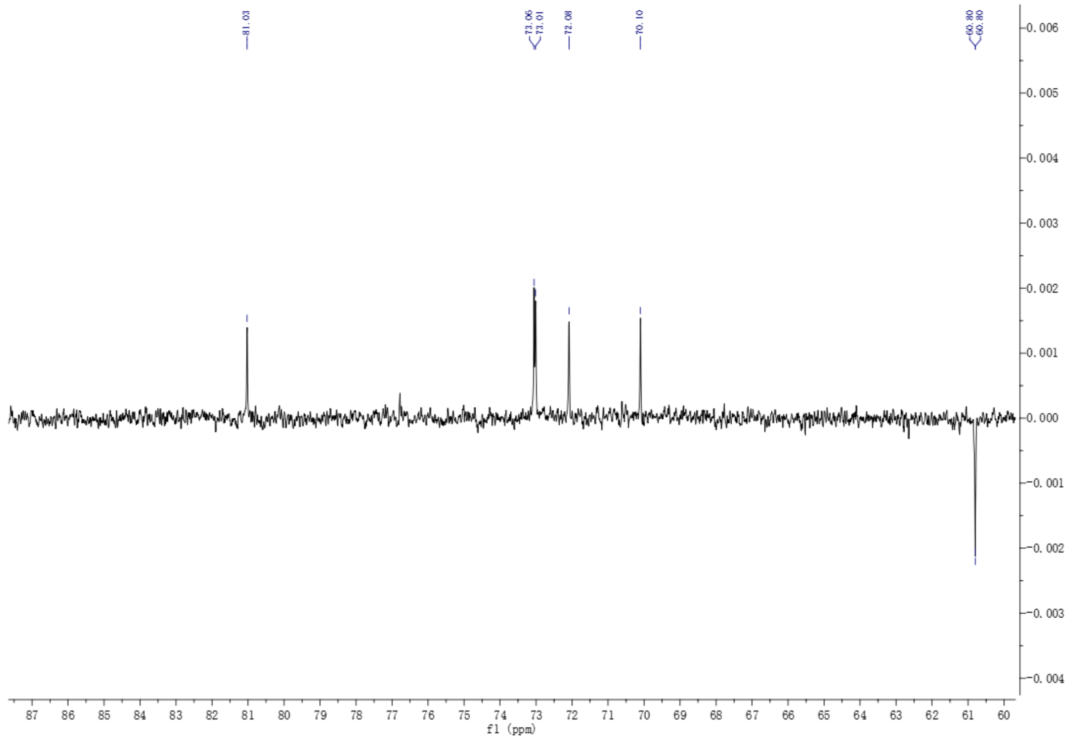

Figure S19. DEPT spectrum of peniresorcinoside A (**1**, in DMSO-d_6)

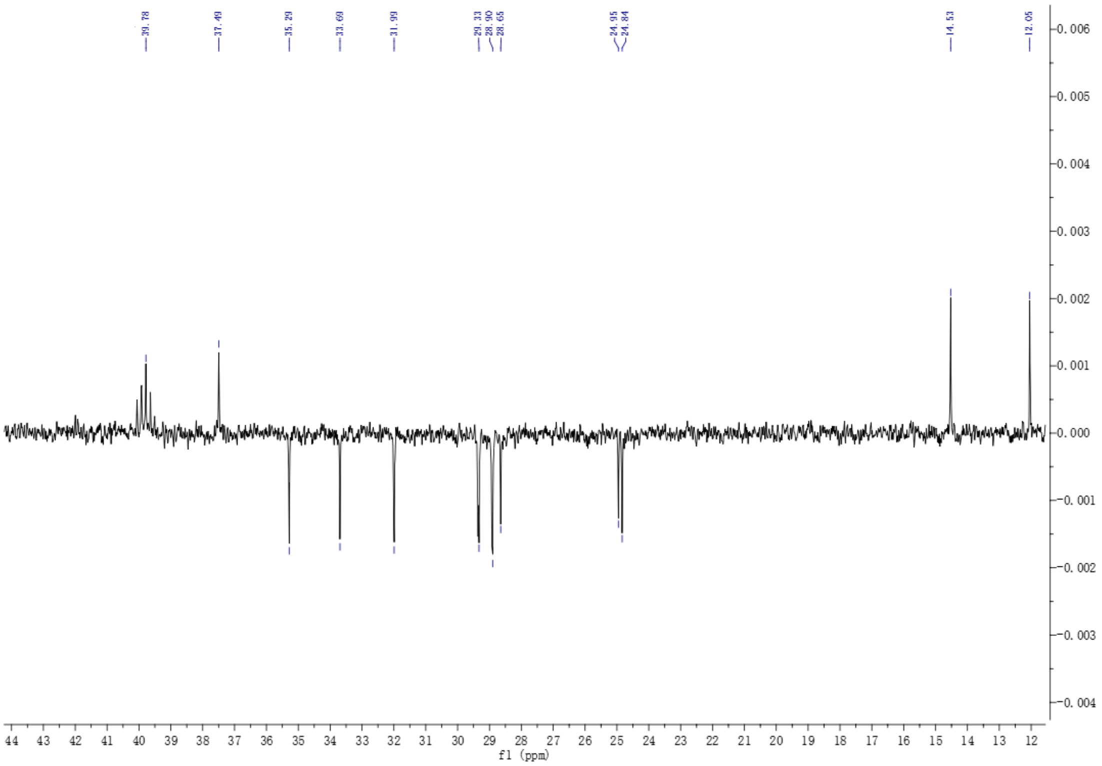

Figure S20. HMQC spectrum of peniresorcinoside A (**1**, in DMSO-d_6)

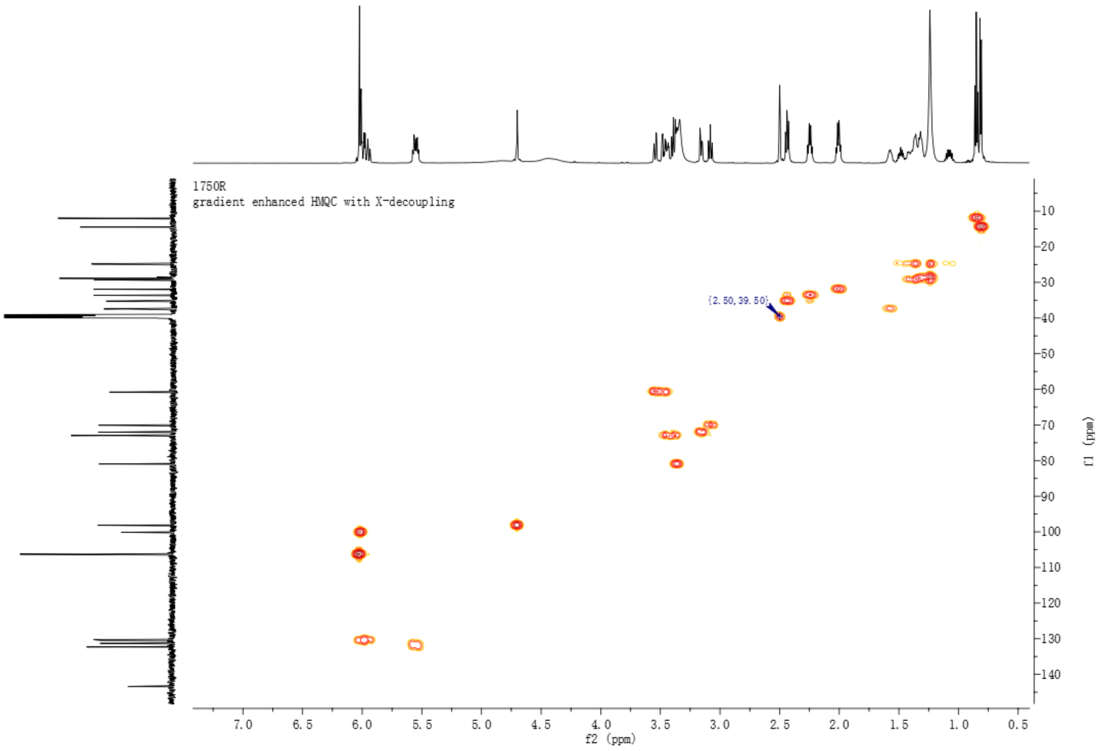

Figure S21. HMQC spectrum of peniresorcinoside A (**1**, in DMSO-d_6)

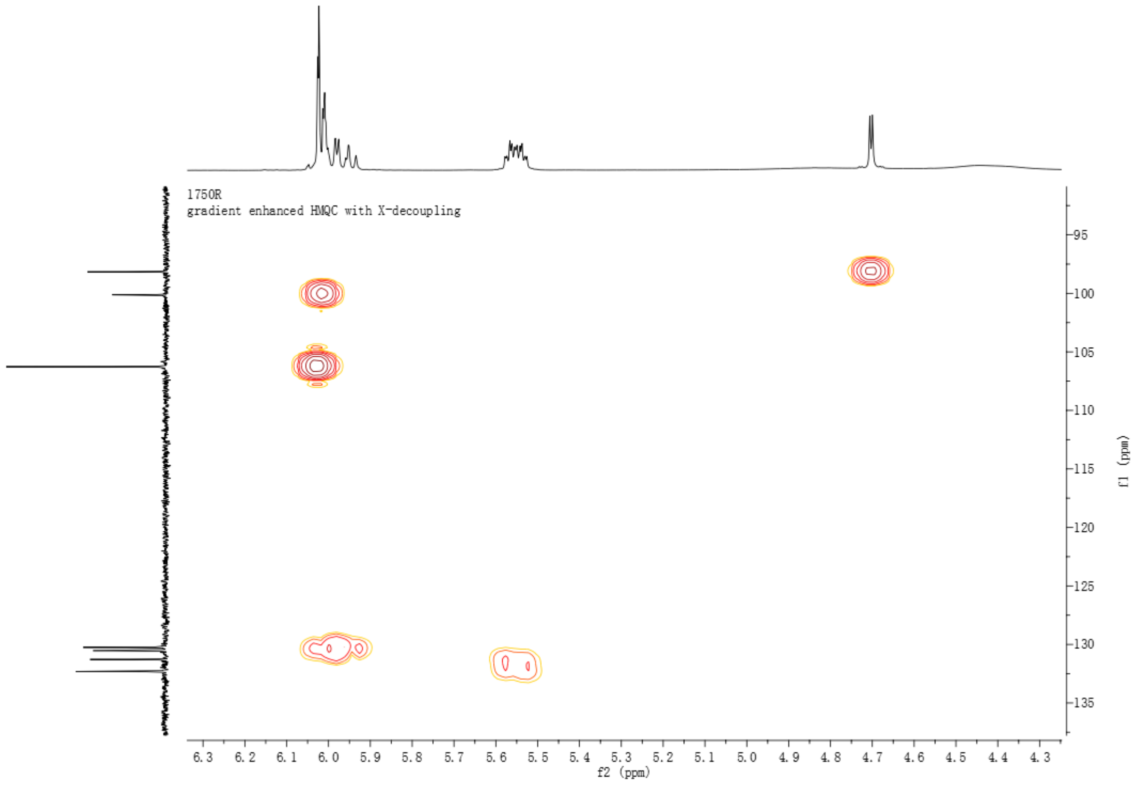

Figure S22. HMQC spectrum of peniresorcinoside A (**1**, in DMSO-d_6)

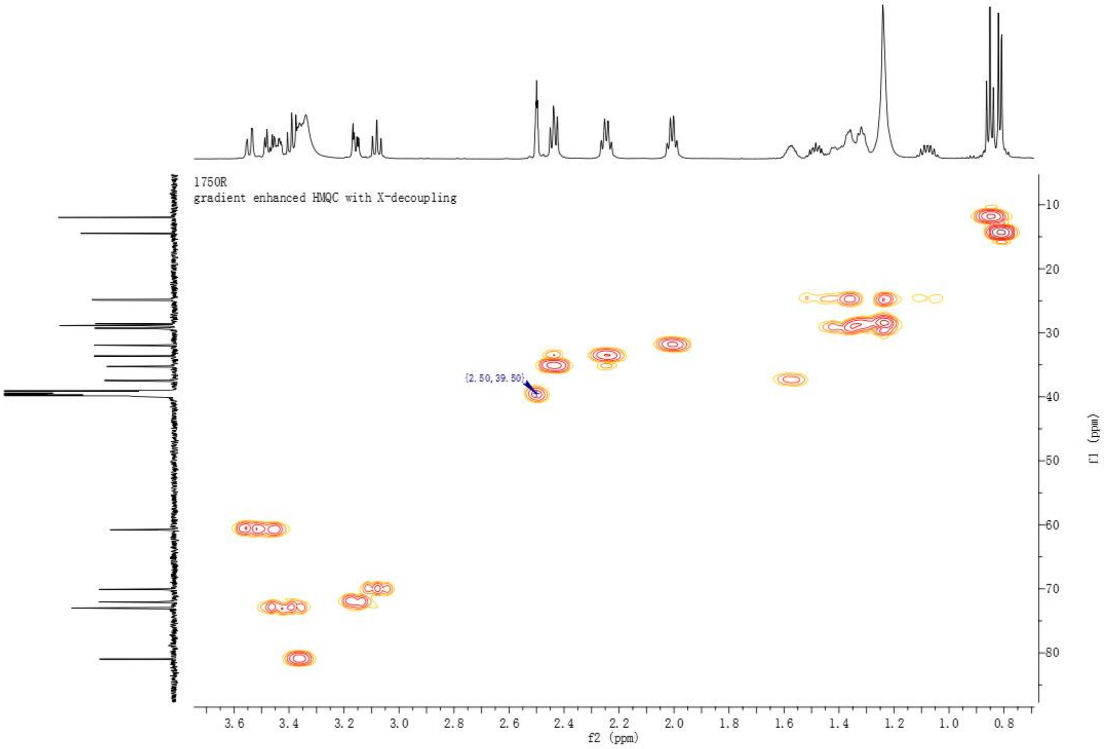

Figure S23. COSY spectrum of peniresorcinoside A (**1**, in DMSO-d_6)

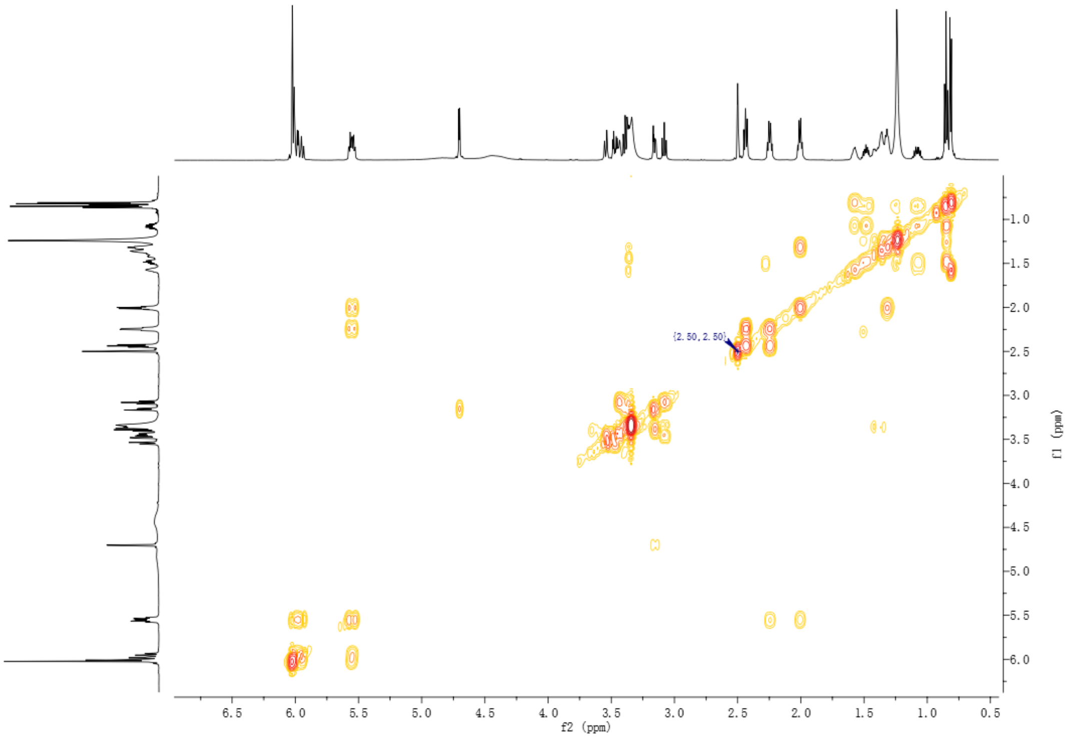

Figure S24. HMBC spectrum of peniresorcinoside A (**1**, in DMSO-d_6)

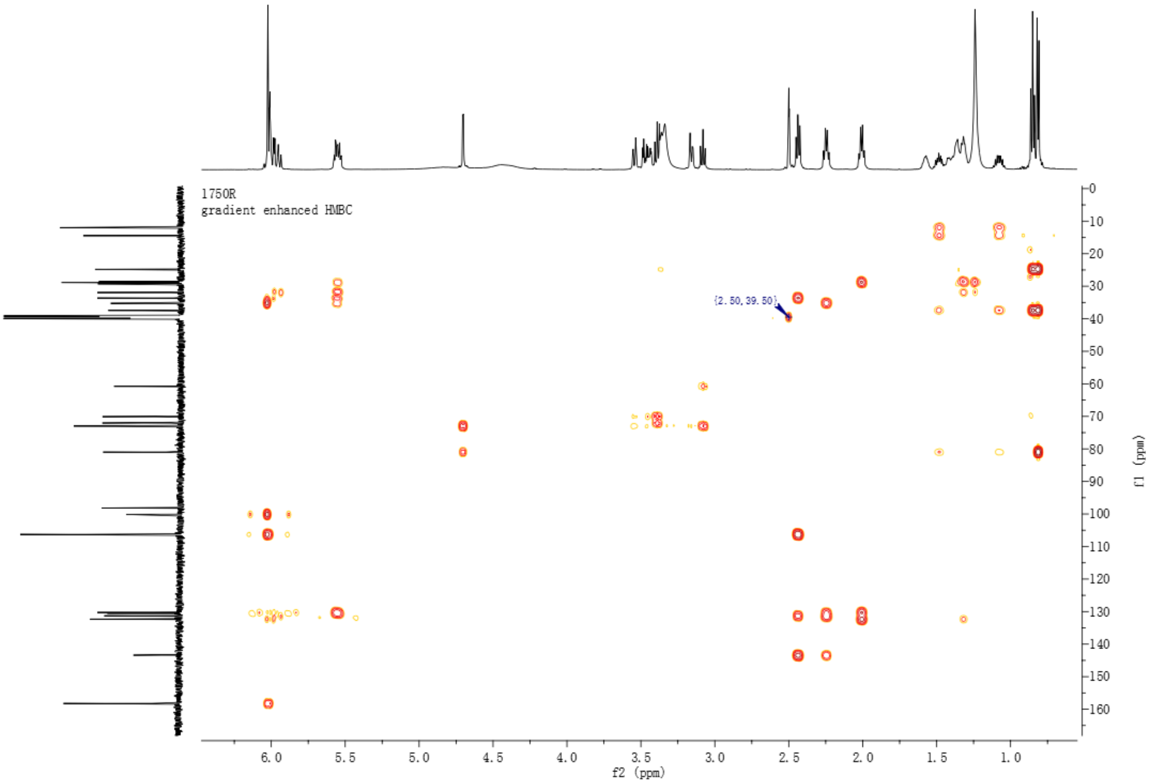

Figure S25. HMBC spectrum of peniresorcinoside A (**1**, in DMSO-d_6)

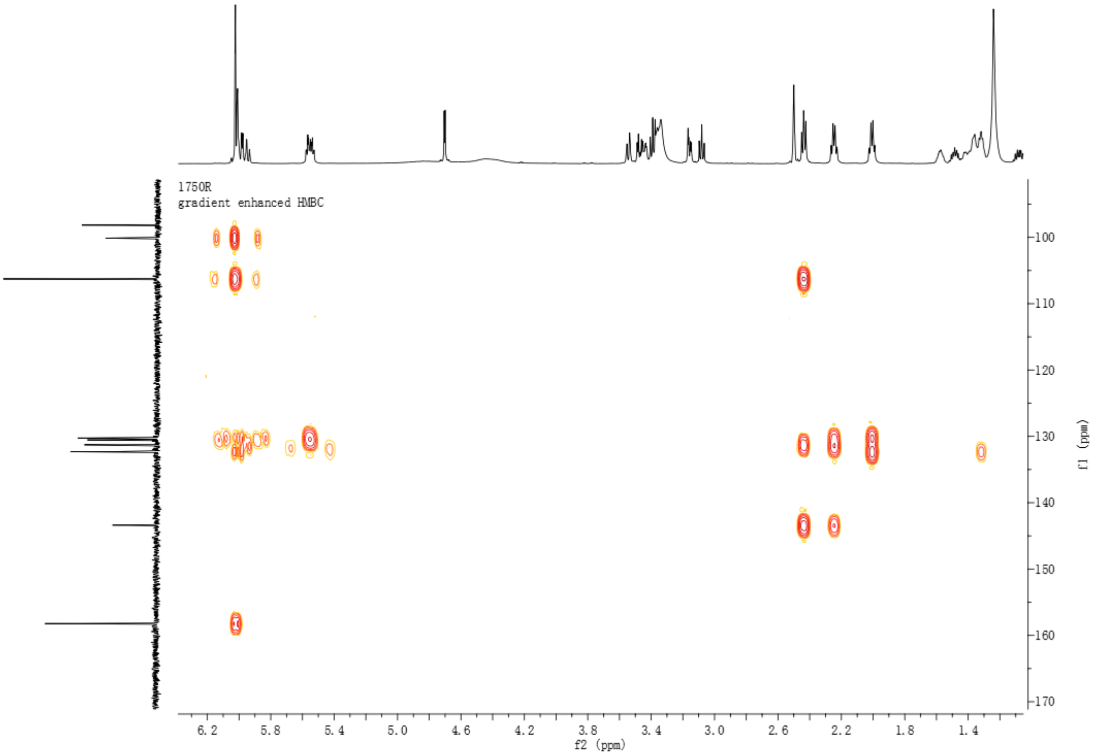

Figure S26. HMBC spectrum of peniresorcinoside A (**1**, in DMSO-d_6)

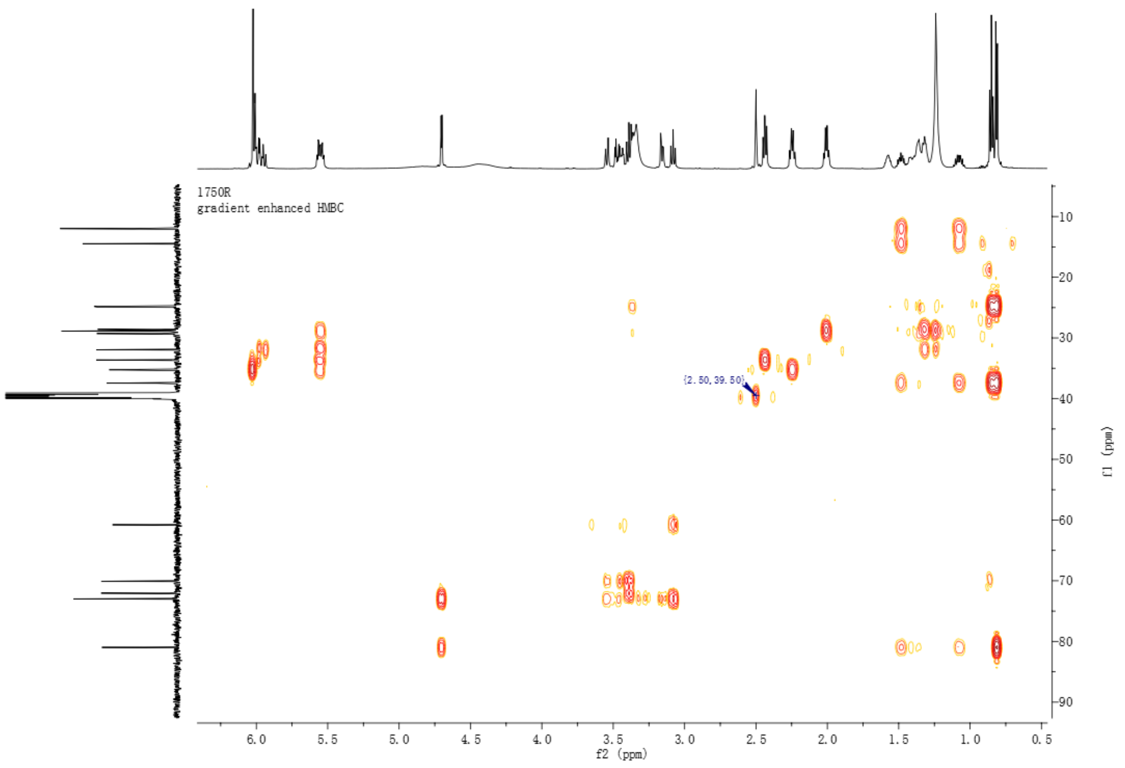

Figure S27. HRESIMS spectrum of peniresorcinoside A (**1**)

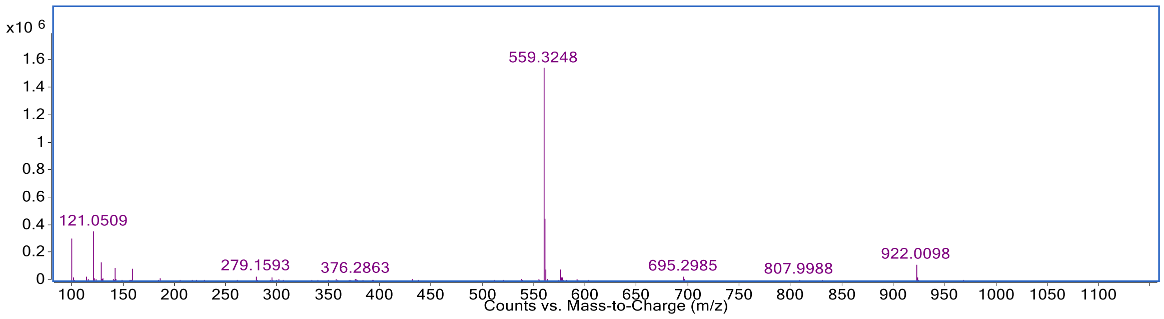

[M+Na]$^+$: 559.3248 (calcd for $C_{30}H_{48}NaO_8$, 559.3247).

Figure S28. UV (MeOH) spectrum of peniresorcinoside A (**1**)

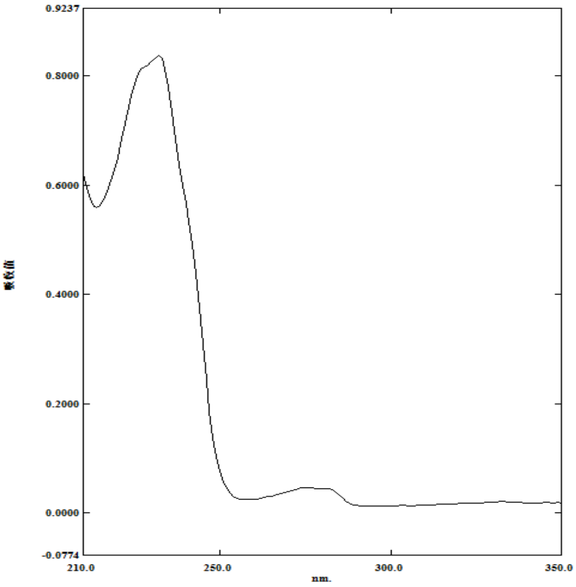

Figure S29. IR (ATR) spectrum of peniresorcinoside A (**1**)

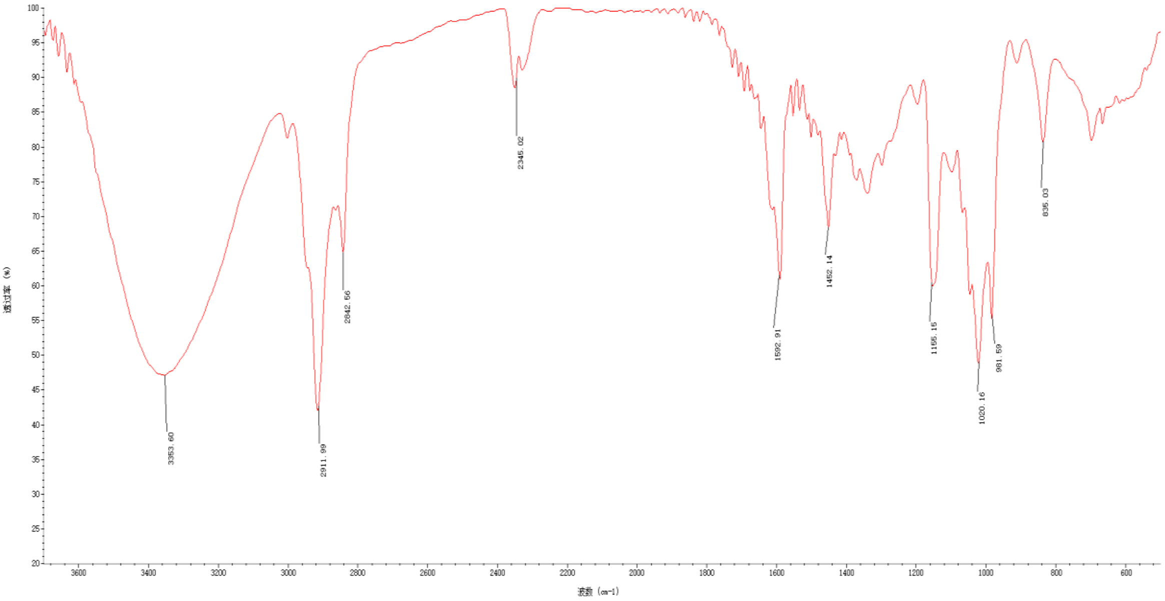

Figure S30. ^{1}H NMR spectrum of compound **1a** (600 MHz, in MeOH-d_4)

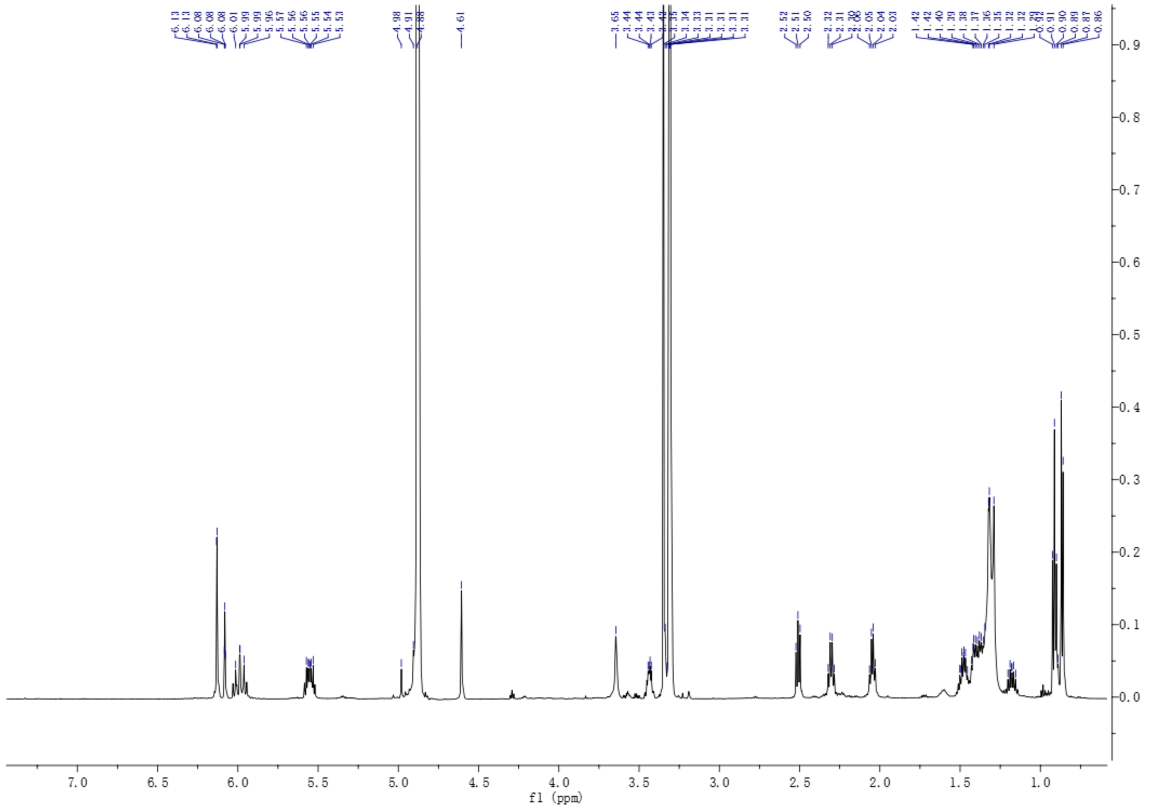

Figure S31. ^{1}H NMR spectrum of compound **1a** (600 MHz, in MeOH-d_4)

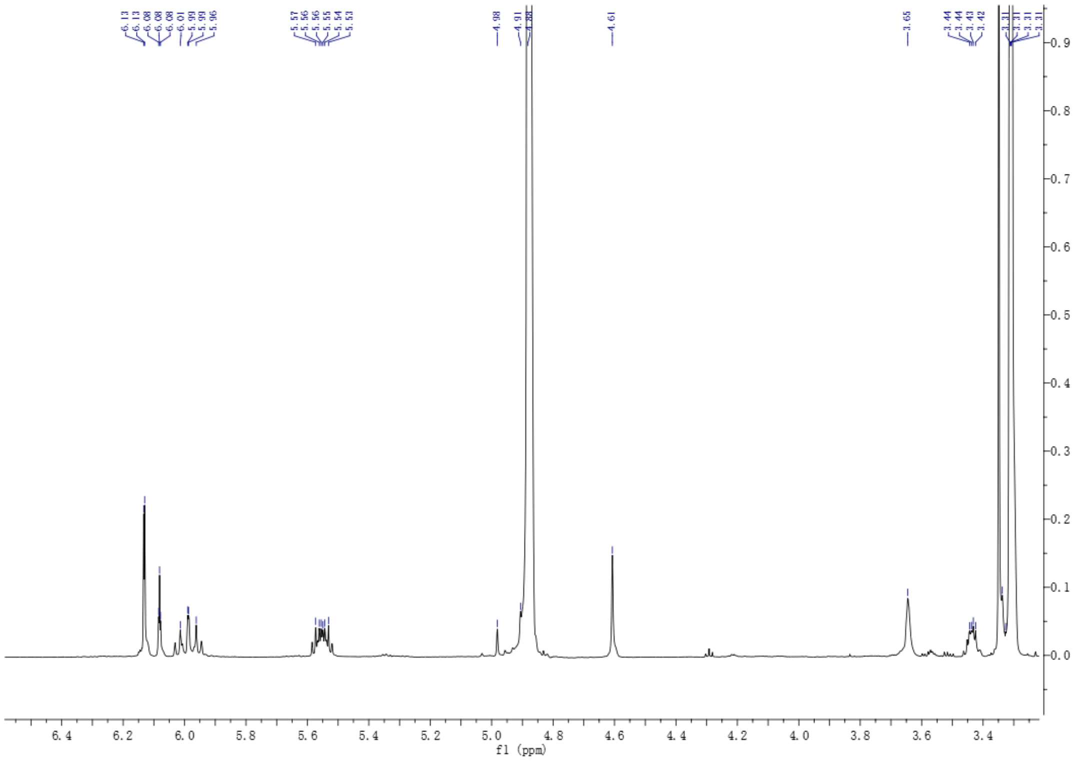

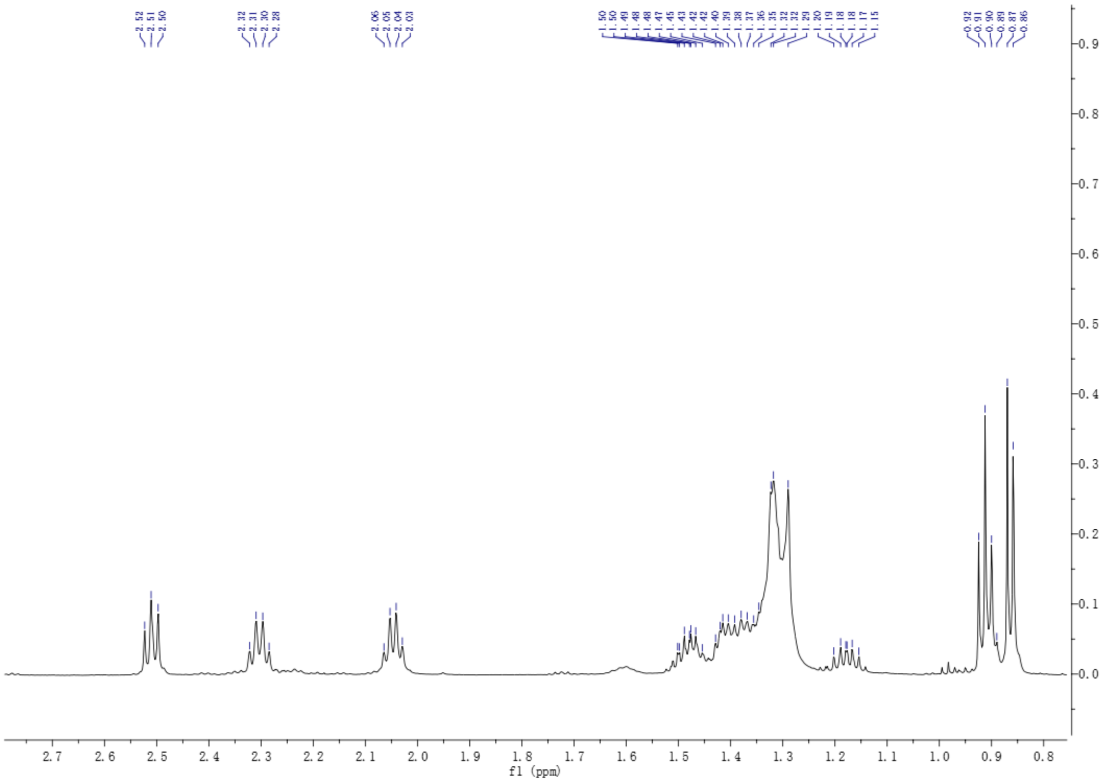

Figure S32. ^{1}H NMR spectrum of compound **1a** (600 MHz, in MeOH-d_4)

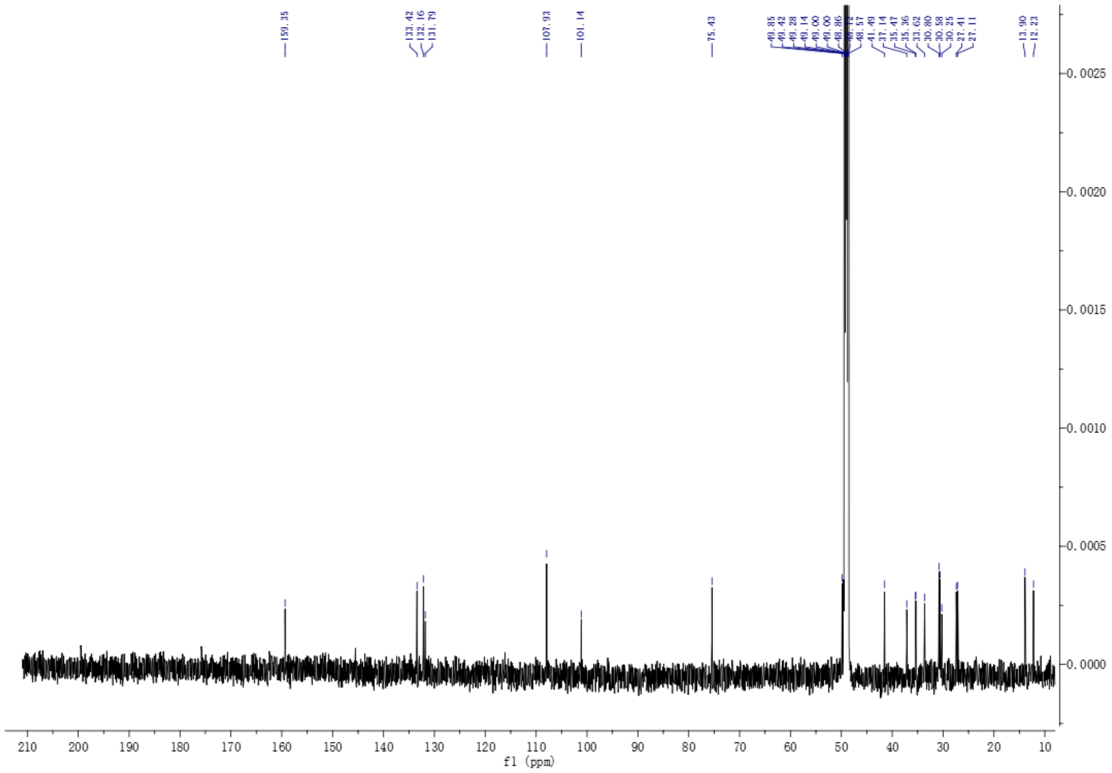

Figure S33. ^{13}C NMR spectrum of compound **1a** (150 MHz, in MeOH-d_4)

Figure S34. ^{13}C NMR spectrum of compound **1a** (150 MHz, in MeOH-d_4)

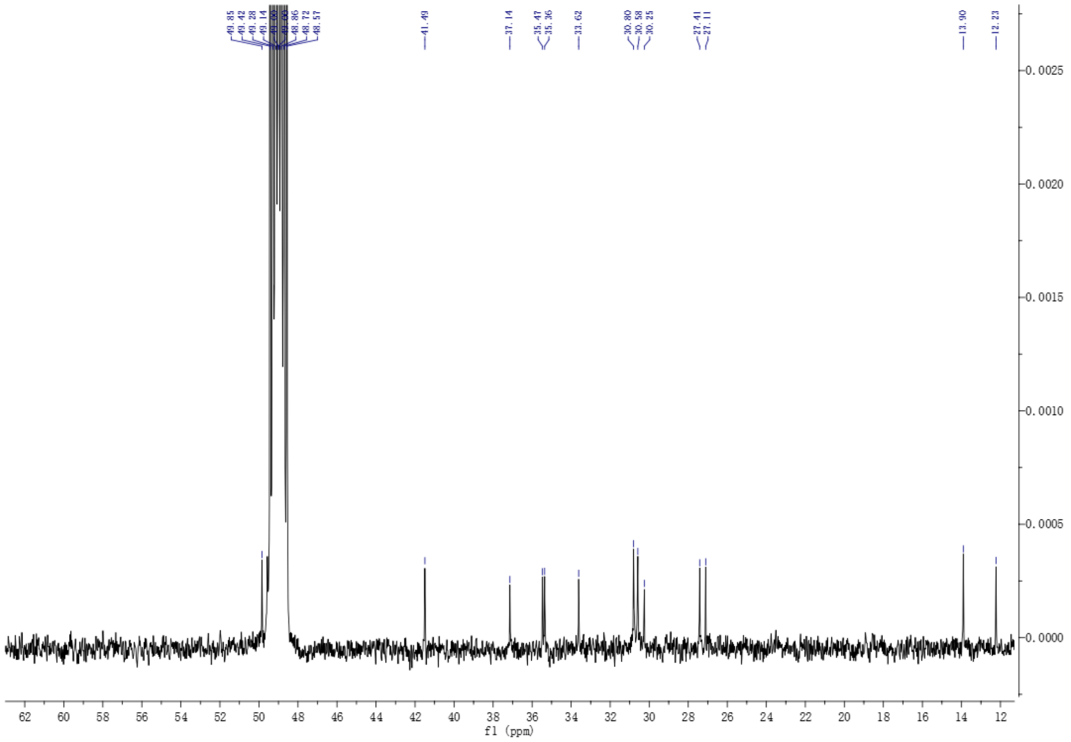

Figure S35. HMQC spectrum of compound **1a** (in MeOH-d_4)

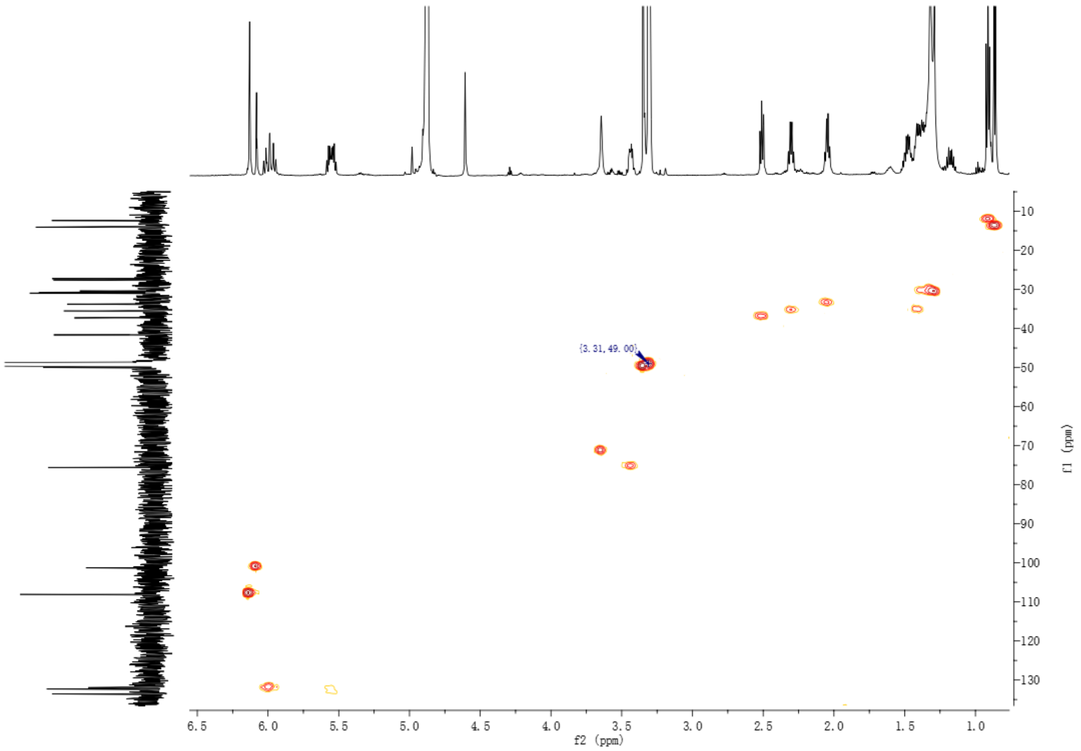

Figure S36. COSY spectrum of compound **1a** (in MeOH-d_4)

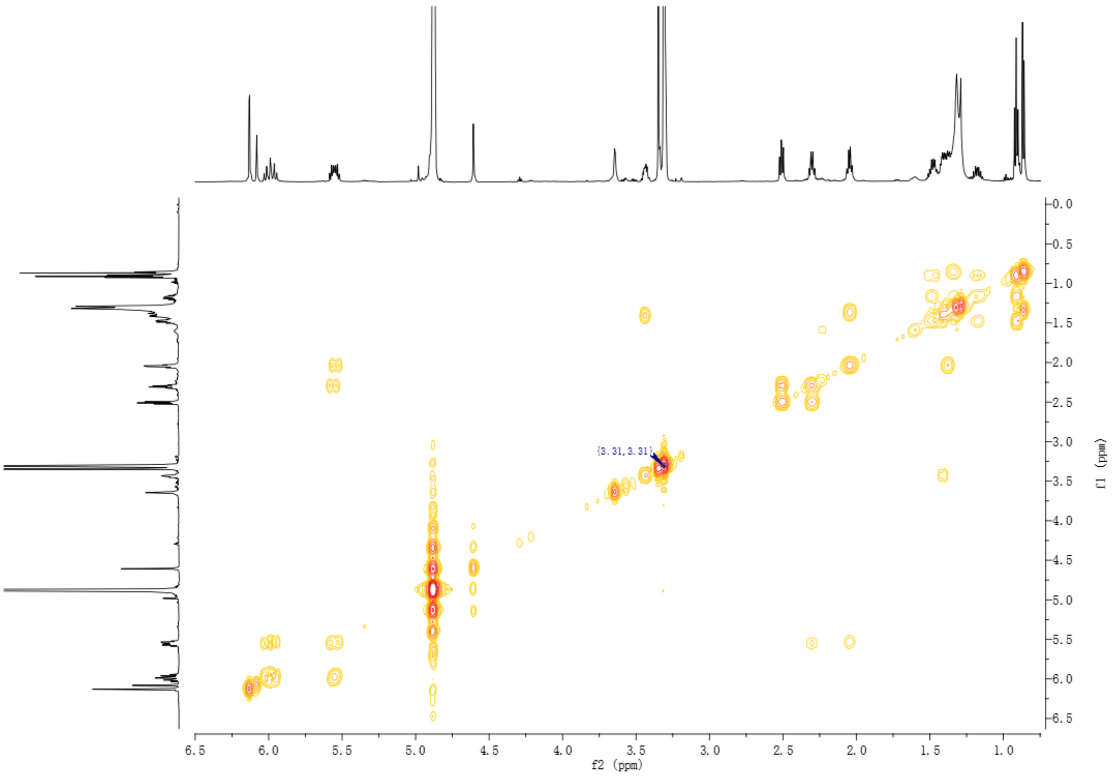

Figure S37. HRESIMS spectrum of compound **1a**

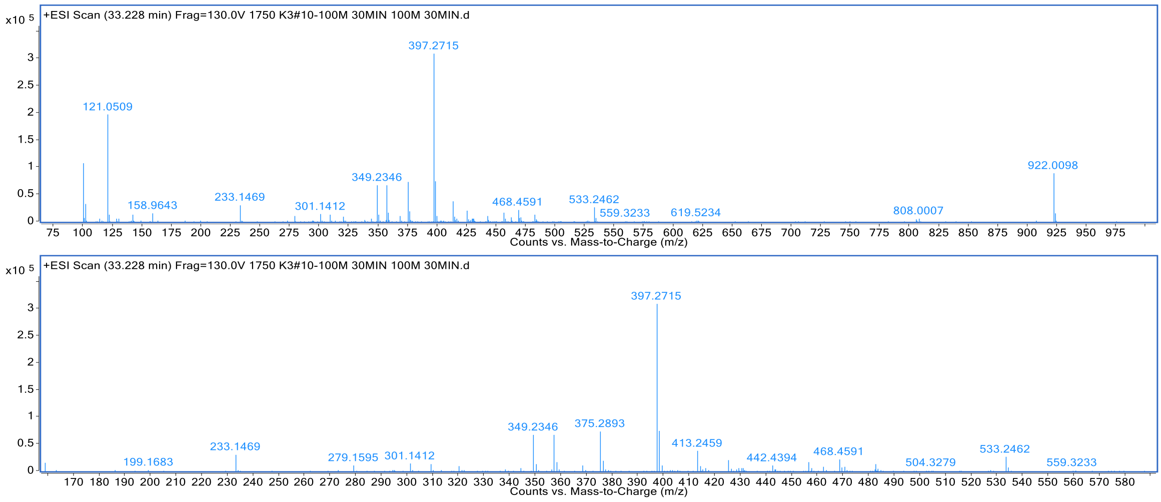

$[M+H]^+$: 375.2893 (calcd for $C_{24}H_{39}O_3$ 375.2899); $[M+Na]^+$: 397.2715 (calcd for $C_{24}H_{38}NaO_3$ 397.2719).

Figure S38. ^{1}H NMR spectrum of compound **1as** (600 MHz, in MeOH-d_4)

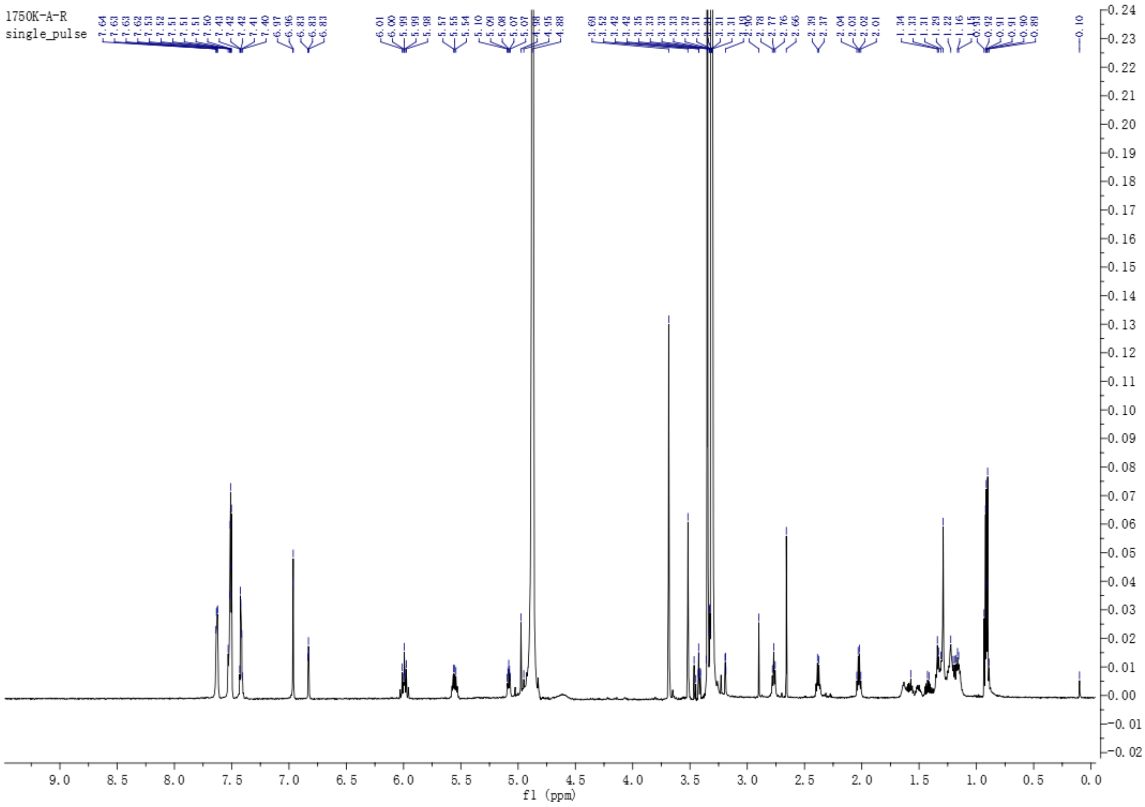

Figure S39. COSY spectrum of compound **1as** (in MeOH-d_4)

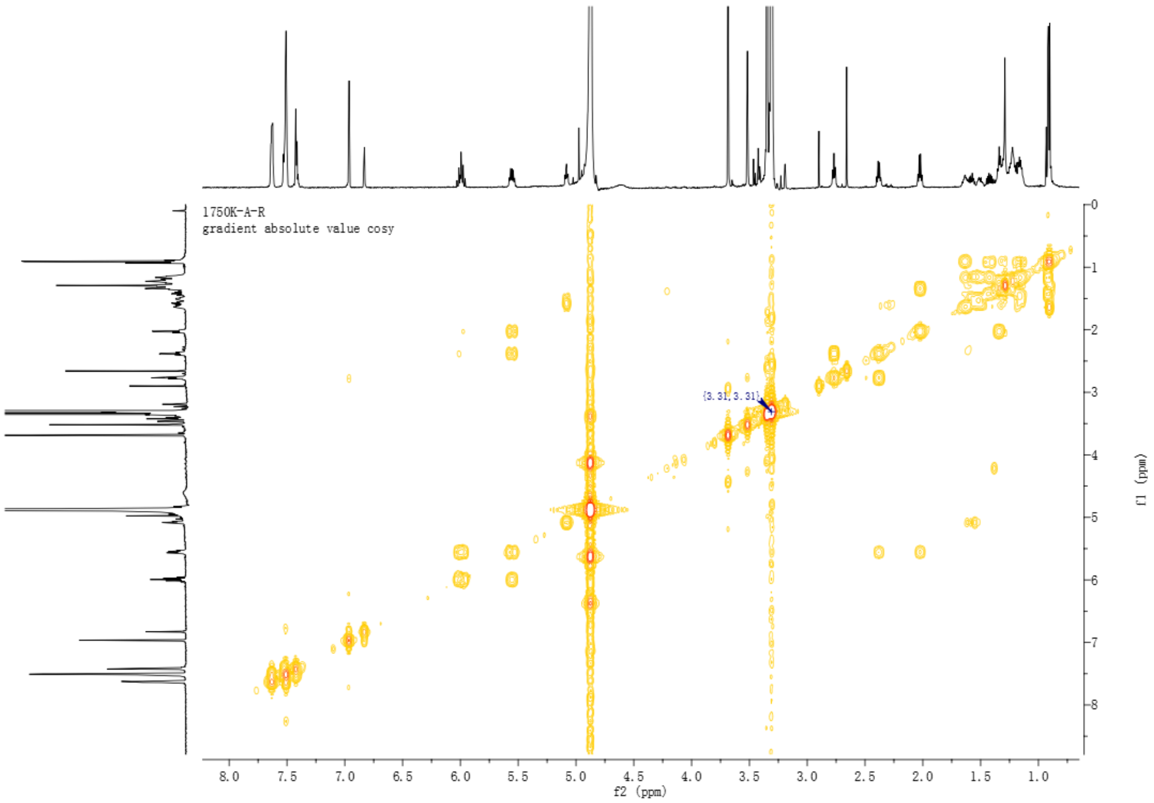

Figure S40. HRESIMS spectrum of compound **1as**

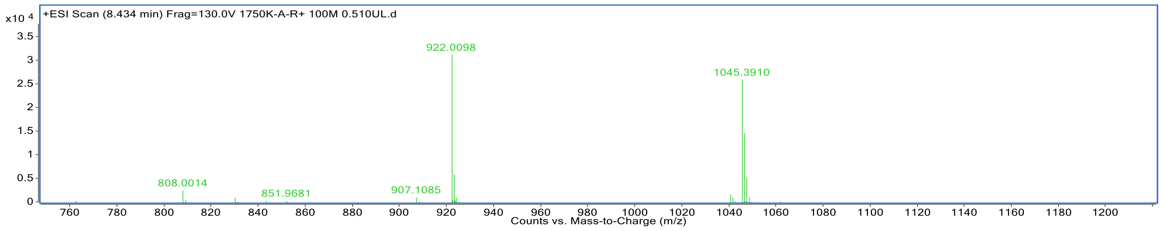

[M+Na]+: 1045.3910 (calcd for $C_{54}H_{59}F_9NaO_9$ 1045.3913).

Figure S41. ^{1}H NMR spectrum of compound **1ar** (600 MHz, in MeOH-d_4)

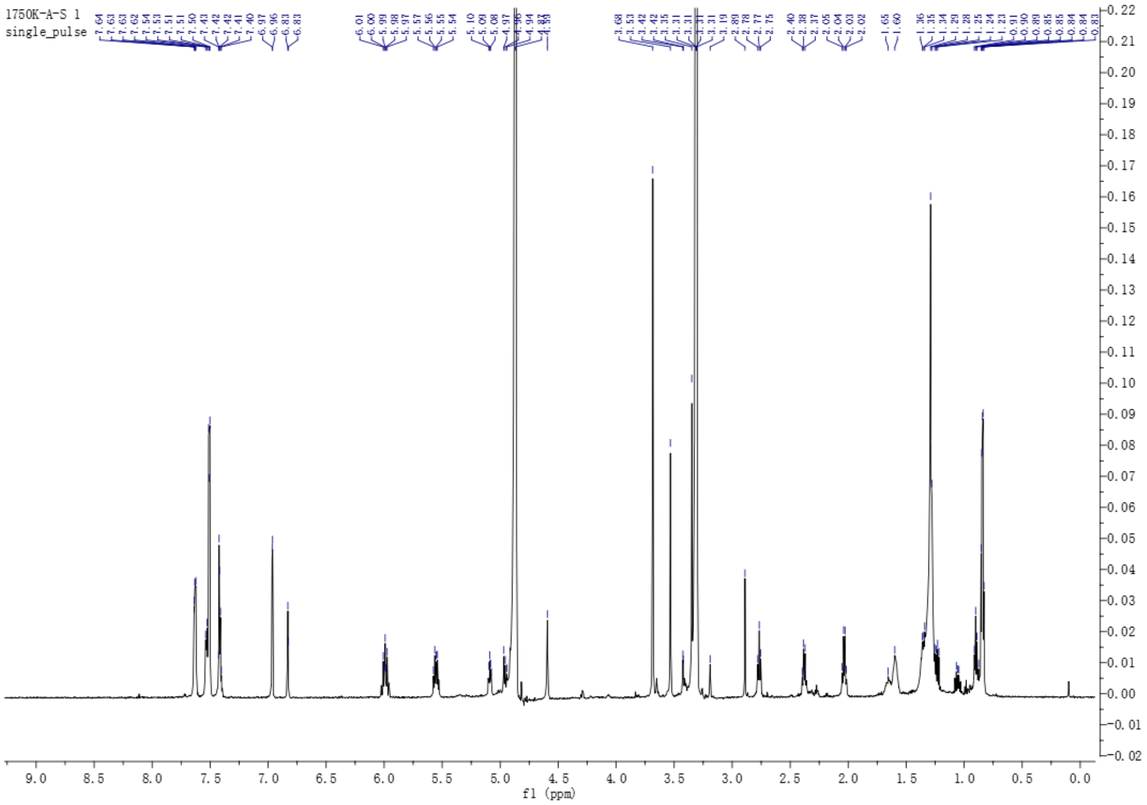

Figure S42. COSY spectrum of compound **1ar** (in MeOH-d_4)

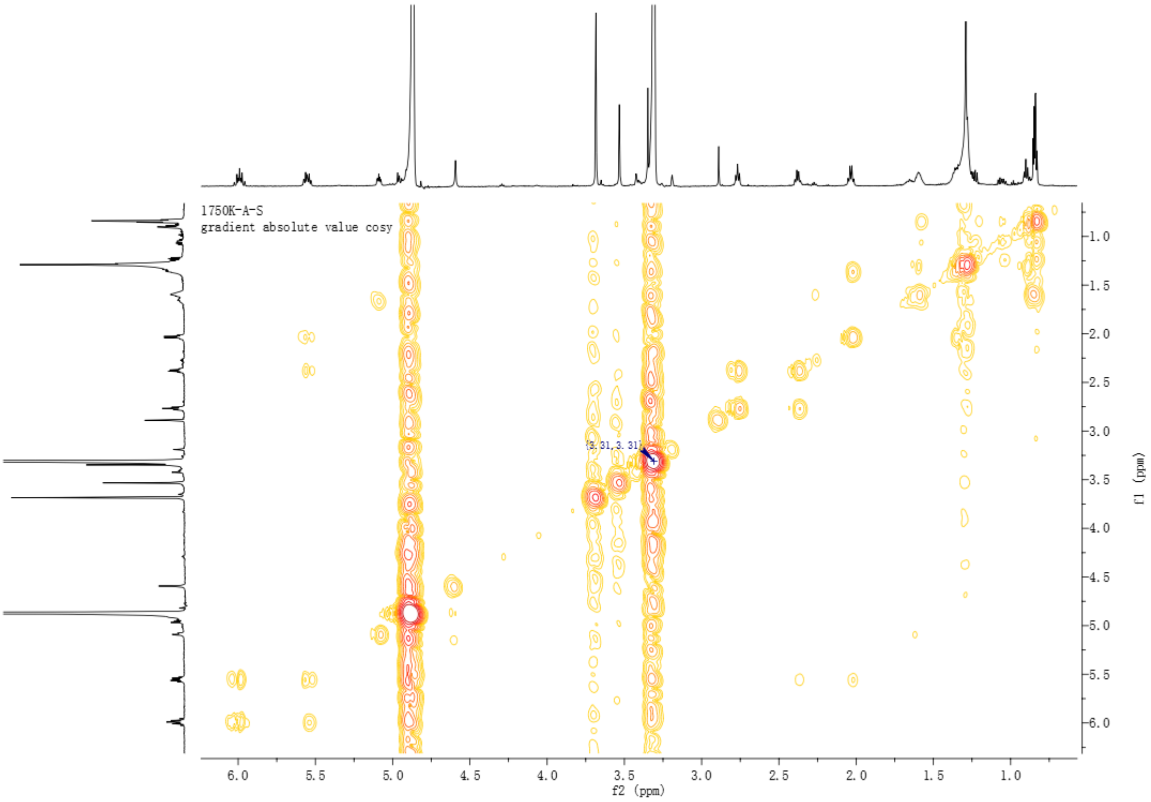

Figure S43. HRESIMS spectrum of compound **1ar**

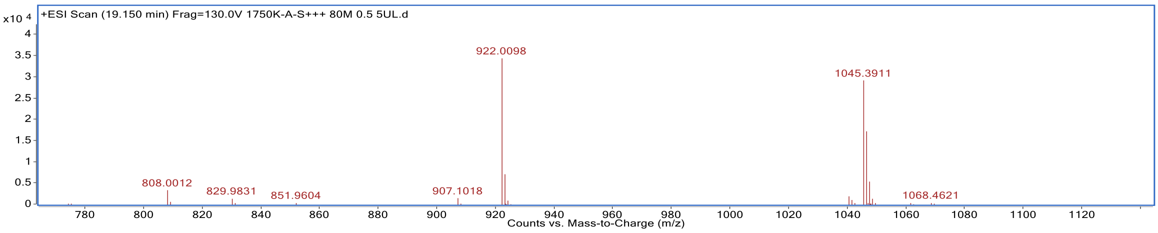

$[M+Na]^+$: 1045.3911 (calcd for $C_{54}H_{59}F_9NaO_9$ 1045.3913).

Figure S44. ^{1}H NMR spectrum of peniresorcinoside B (**2**, 600 MHz, in DMSO-d_6)

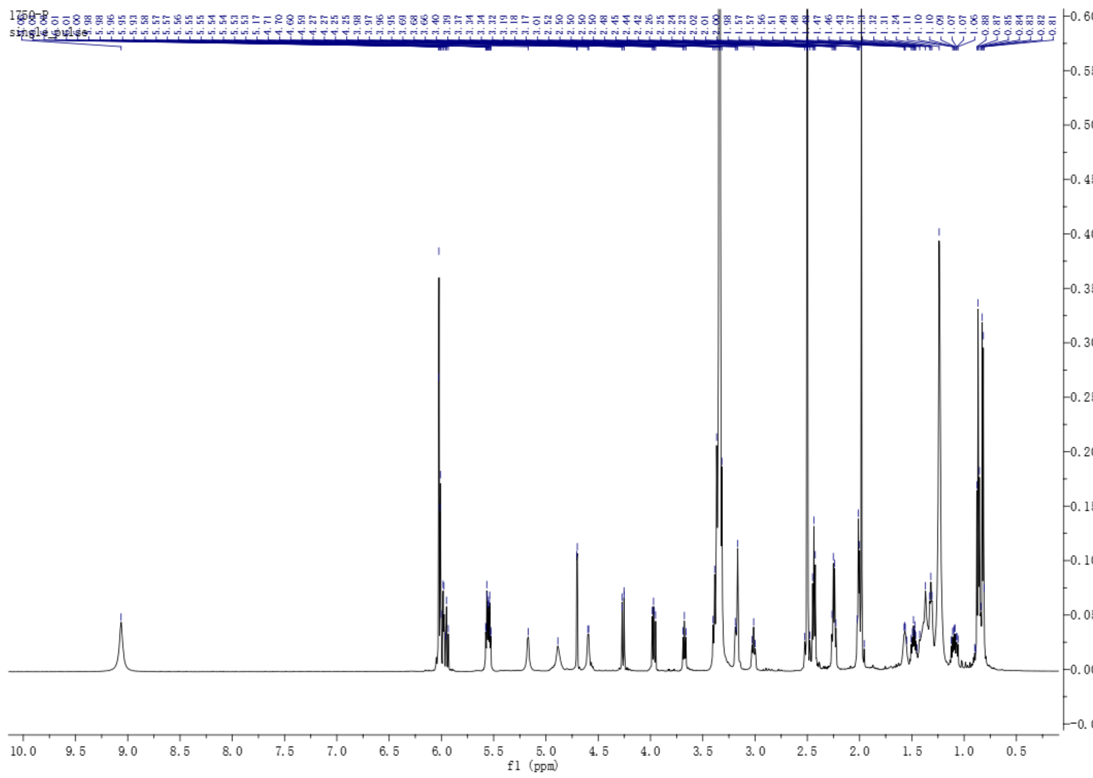

Figure S45. ^{1}H NMR spectrum of peniresorcinoside B (**2**, 600 MHz, in DMSO-d_6)

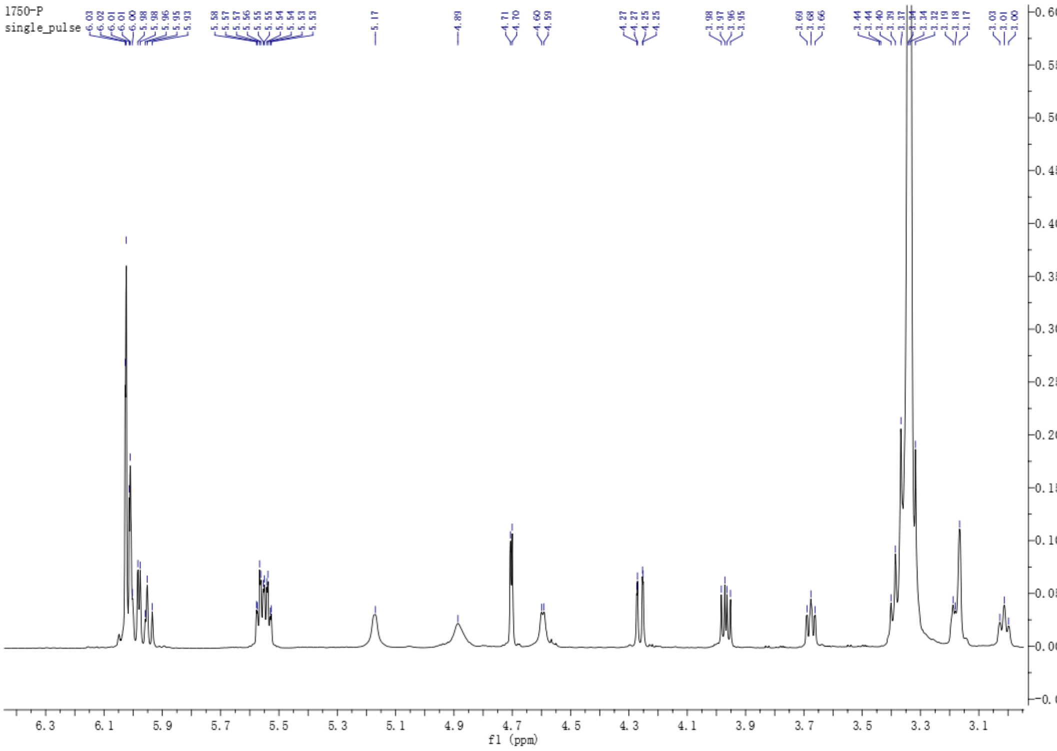

Figure S46. ^{1}H NMR spectrum of peniresorcinoside B (**2**, 600 MHz, in DMSO-d_6)

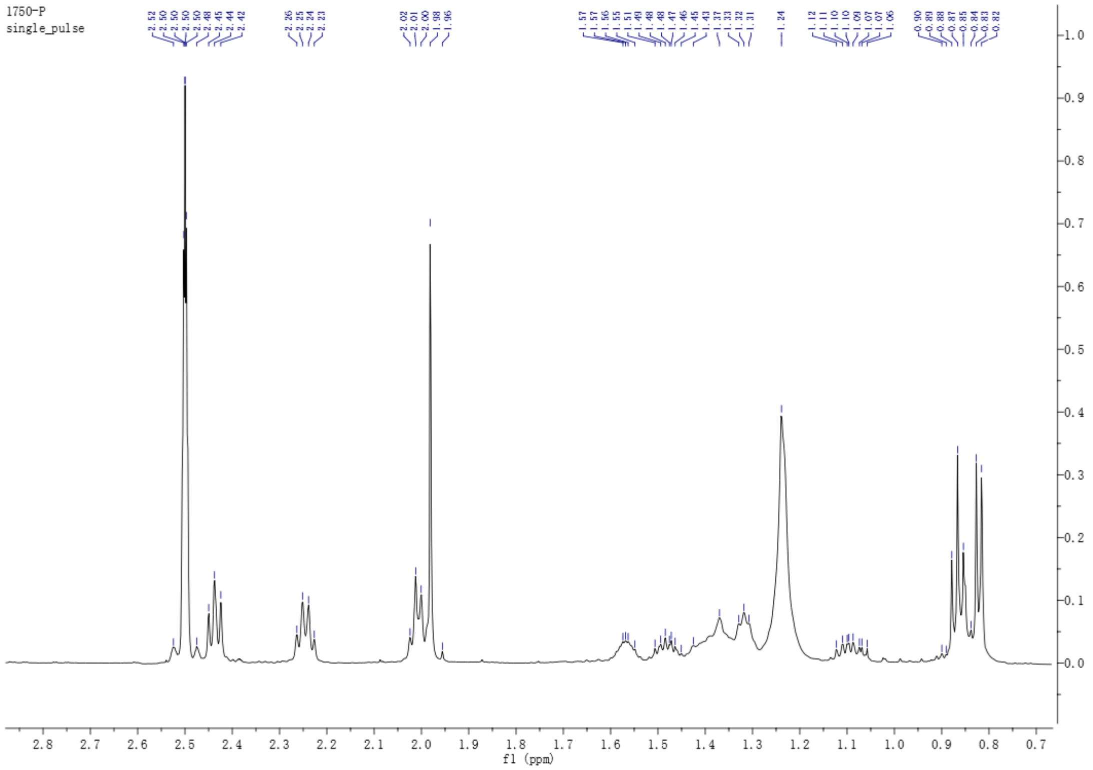

Figure S47. ^{13}C NMR spectrum of peniresorcinoside B (**2**, 150 MHz, in DMSO-d_6)

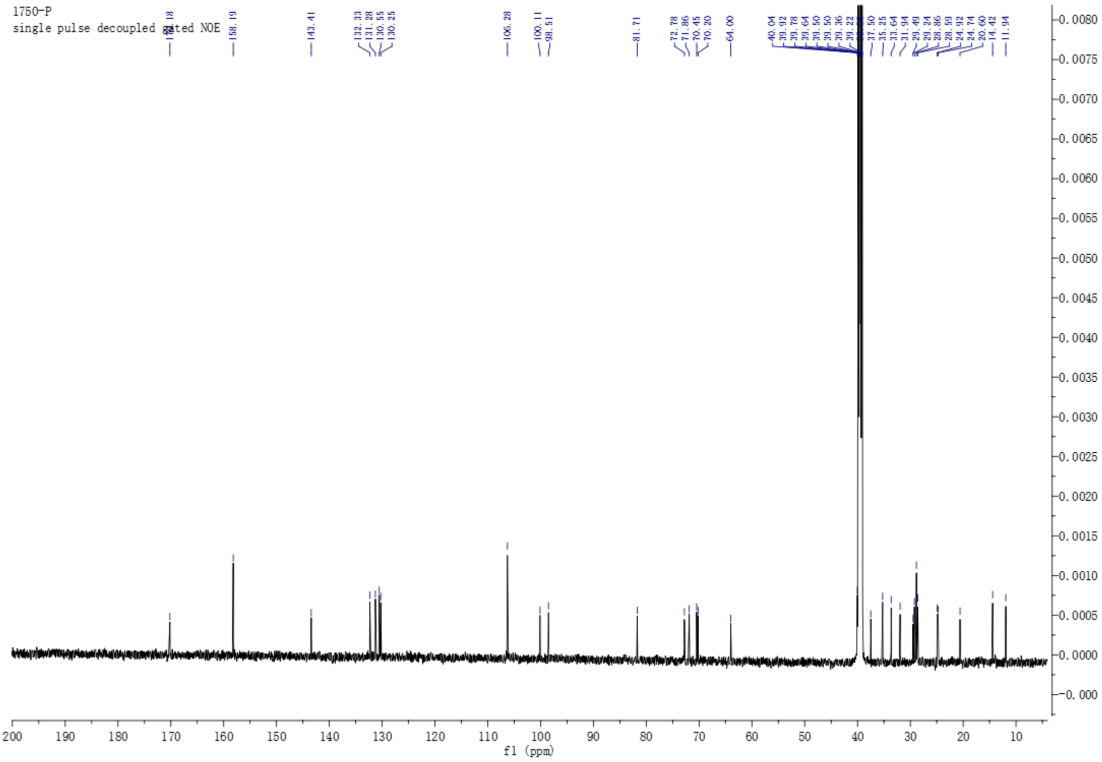

Figure S48. ^{13}C NMR spectrum of peniresorcinoside B (**2**, 150 MHz, in DMSO-d_6)

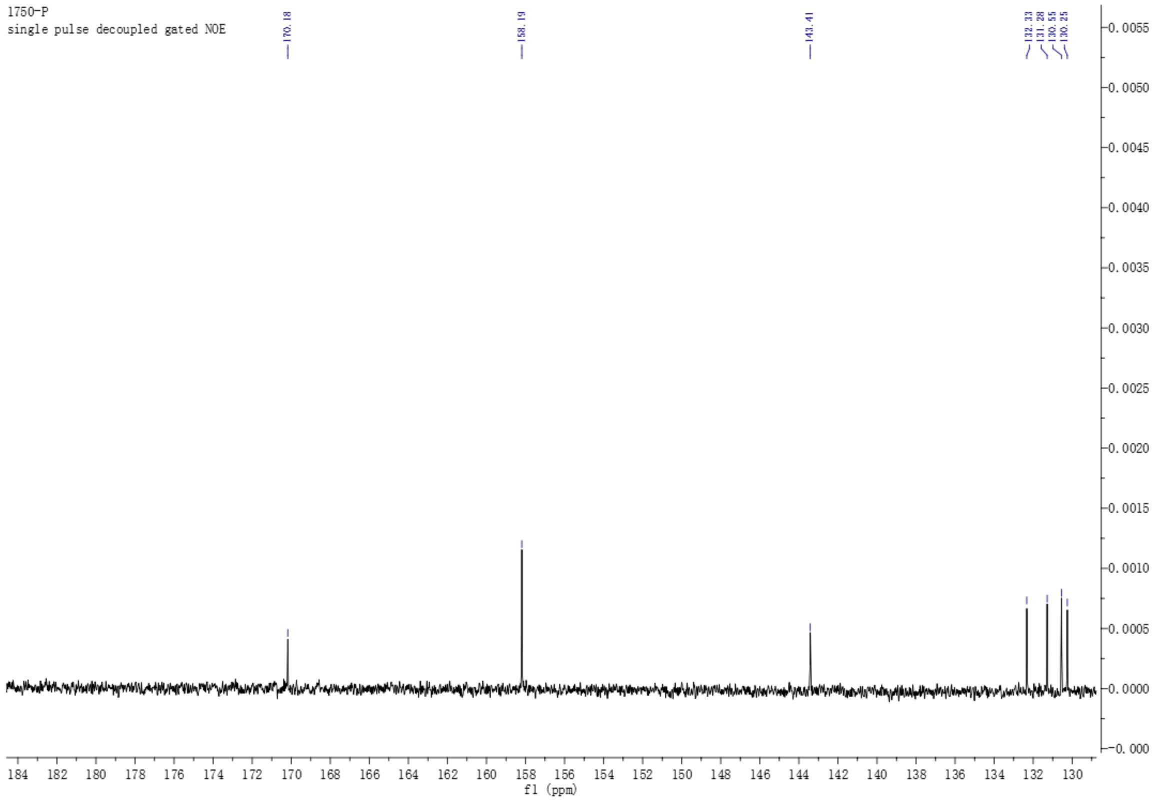

Figure S49. ^{13}C NMR spectrum of peniresorcinoside B (**2**, 150 MHz, in DMSO-d_6)

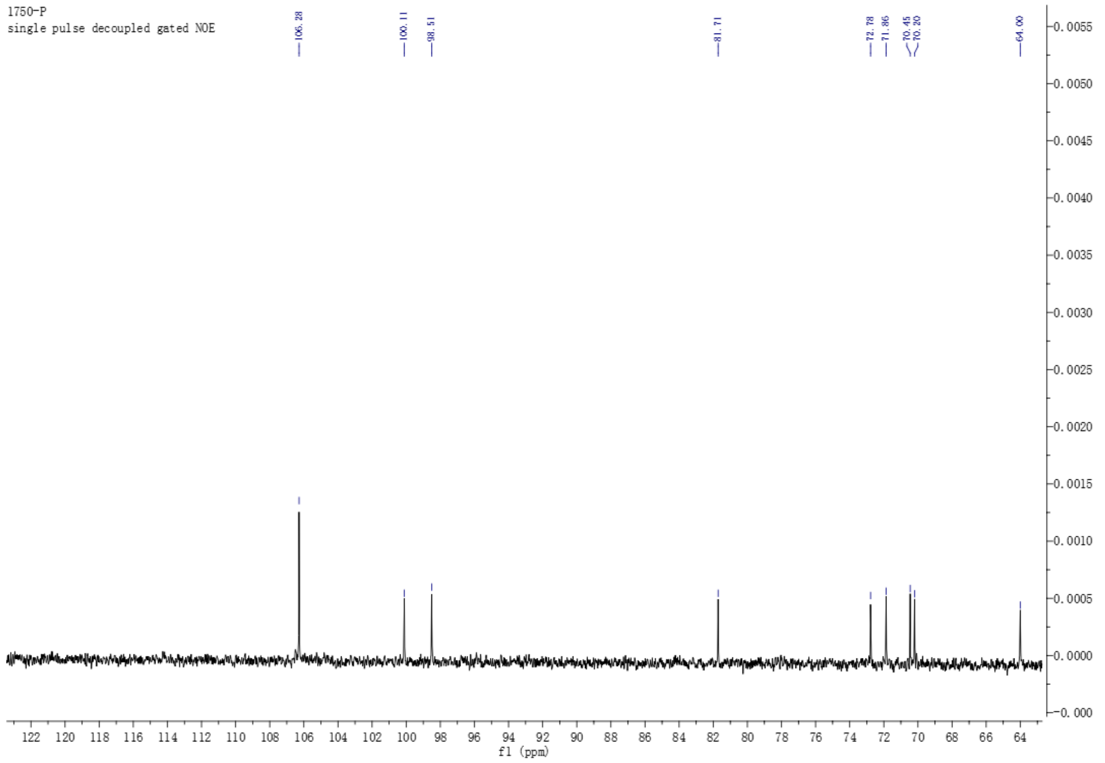

Figure S50. ^{13}C NMR spectrum of peniresorcinoside B (**2**, 150 MHz, in DMSO-d_6)

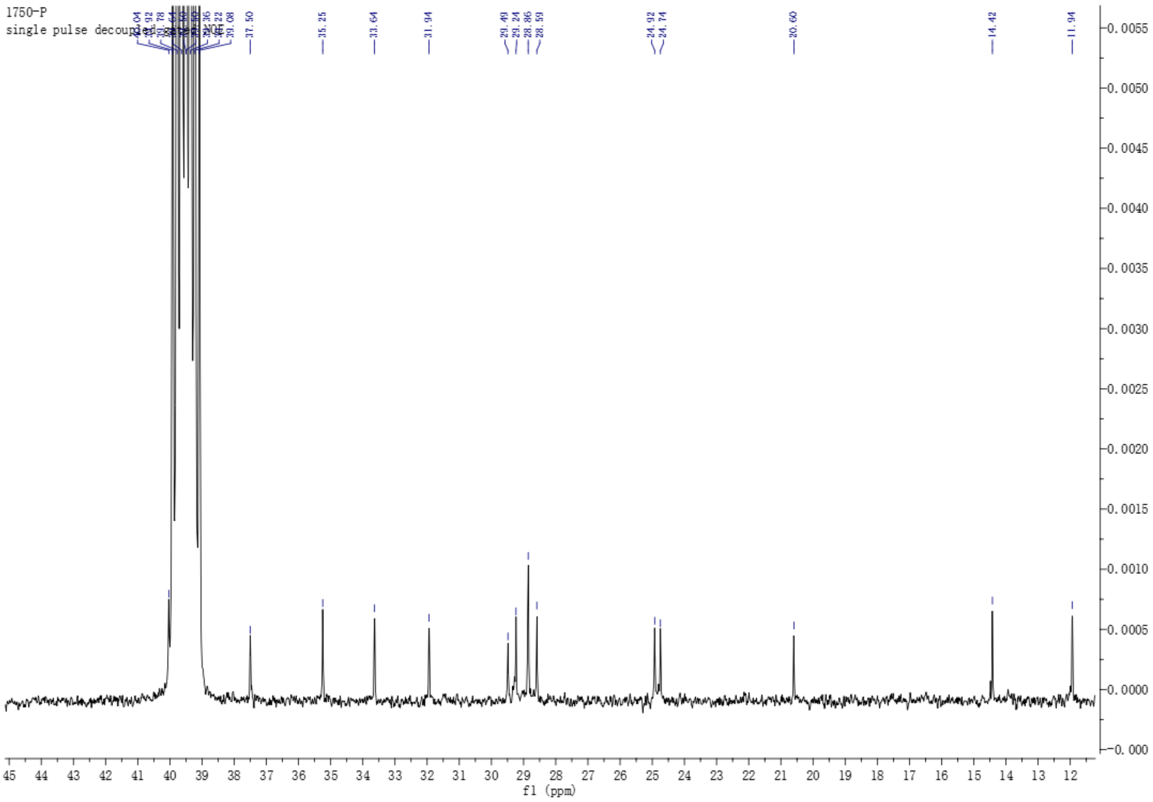

Figure S51. DEPT spectrum of peniresorcinoside B (**2**, in DMSO-d_6)

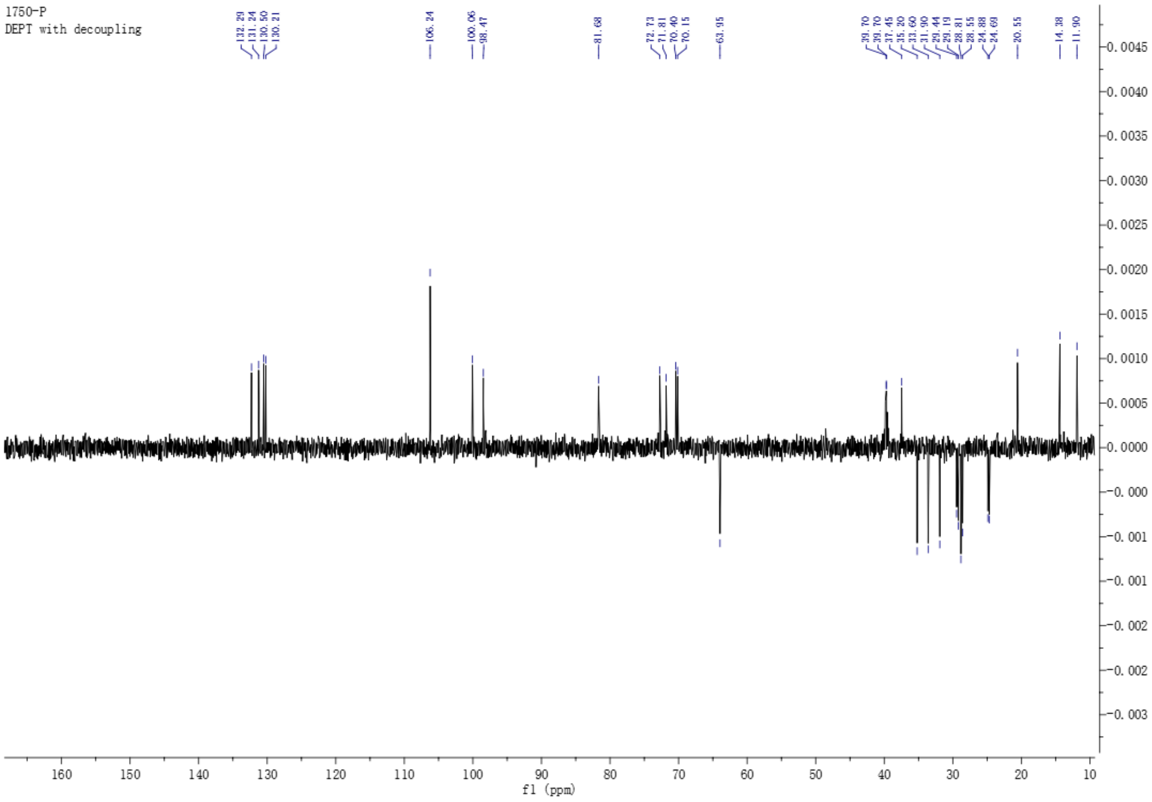

Figure S52. DEPT spectrum of peniresorcinoside B (**2**, in DMSO-d_6)

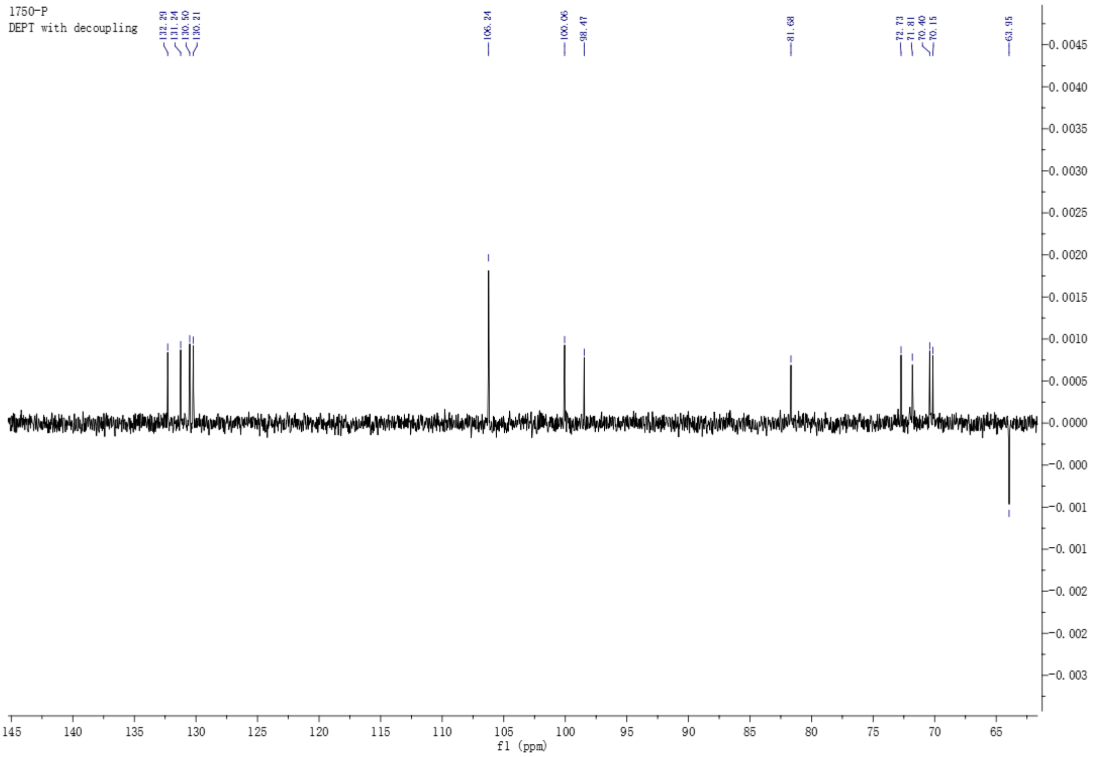

Figure S53. DEPT spectrum of peniresorcinoside B (**2**, in DMSO-d_6)

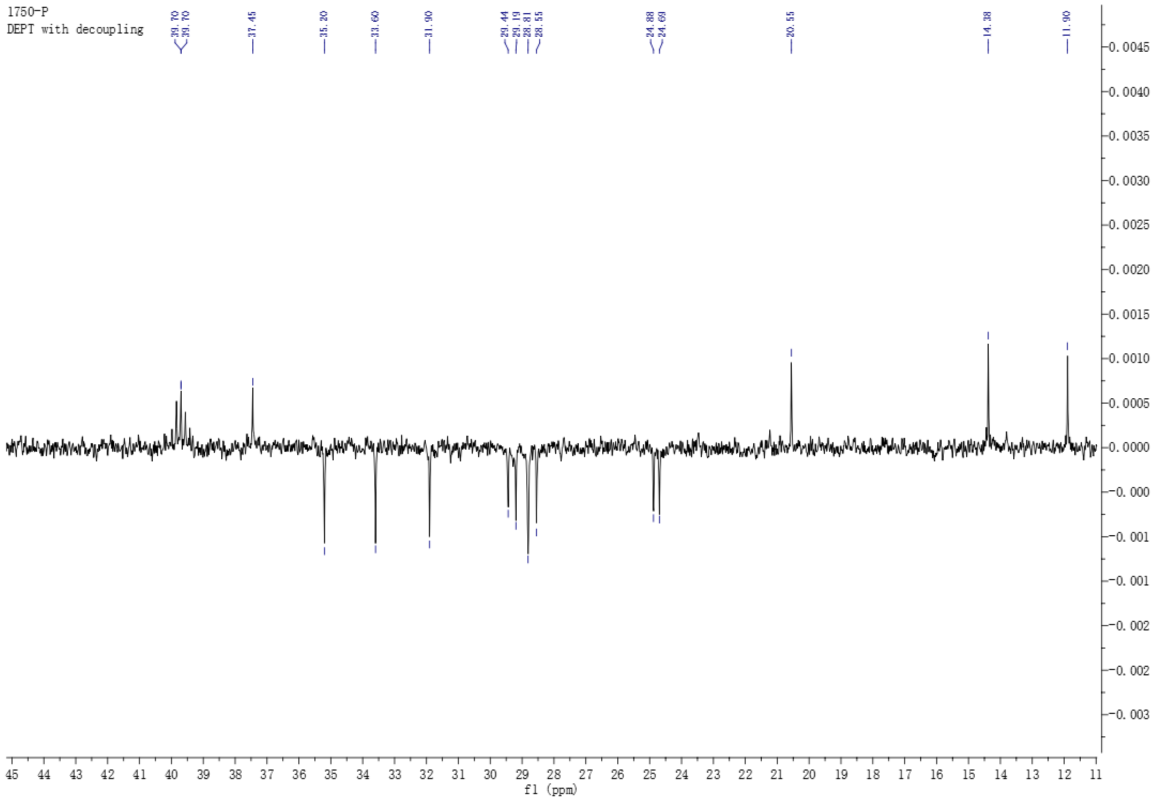

Figure S54. HMQC spectrum of peniresorcinoside B (**2**, in DMSO-d_6)

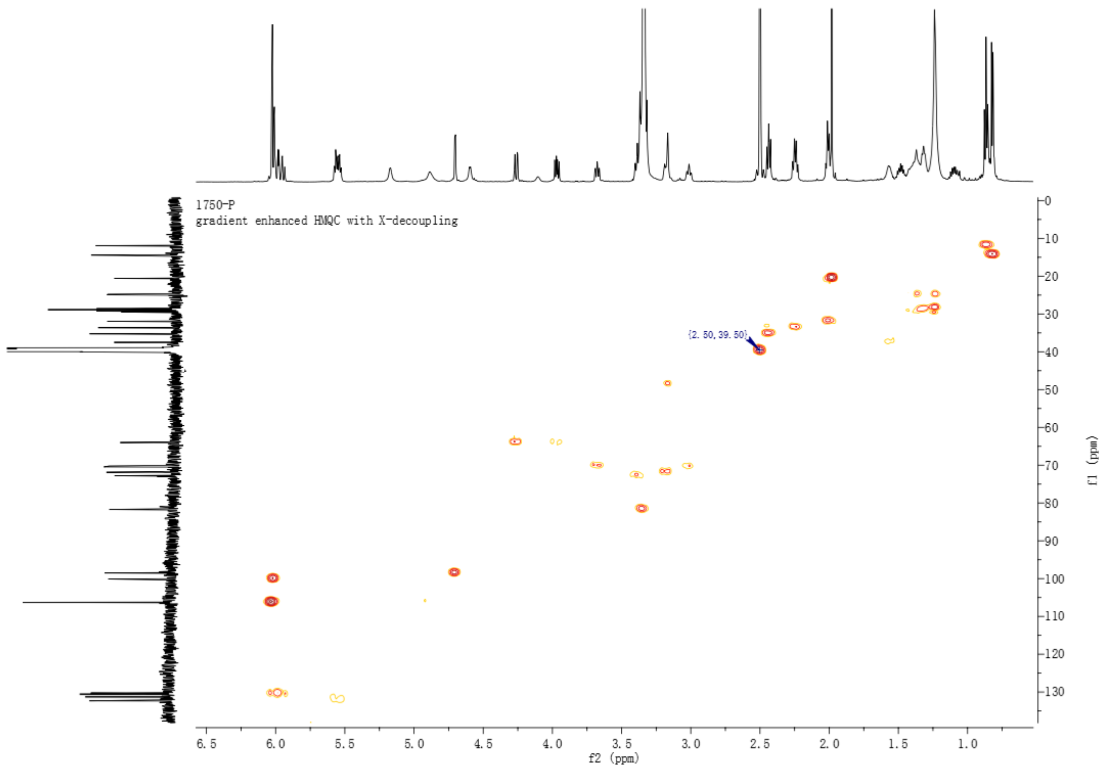

Figure S55. HMQC spectrum of peniresorcinoside B (**2**, in DMSO-d_6)

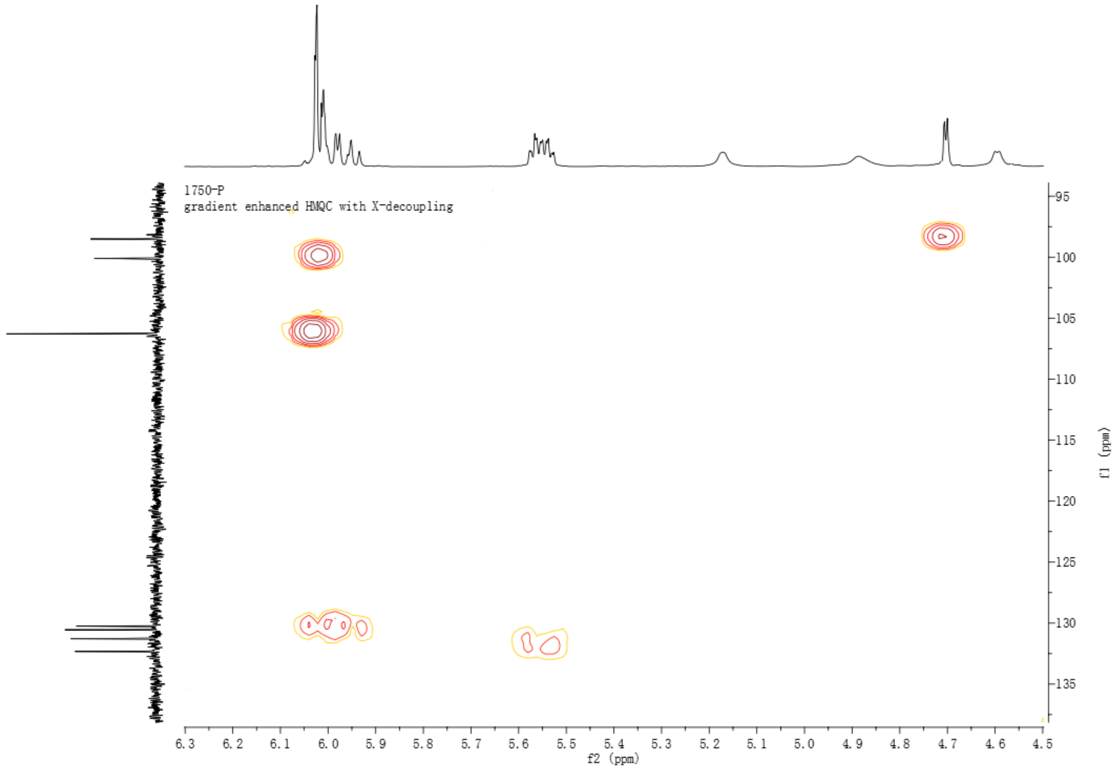

Figure S56. HMQC spectrum of peniresorcinoside B (**2**, in DMSO-d_6)

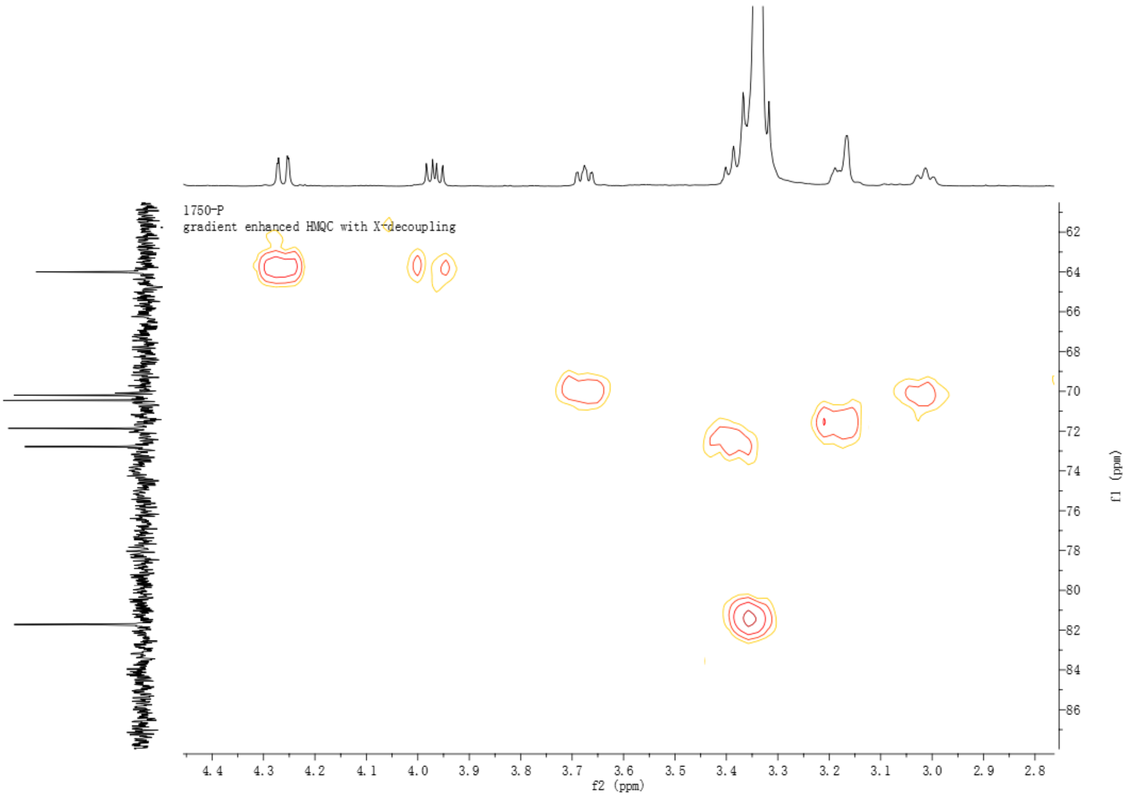

Figure S57. HMQC spectrum of peniresorcinoside B (**2**, in DMSO-d_6)

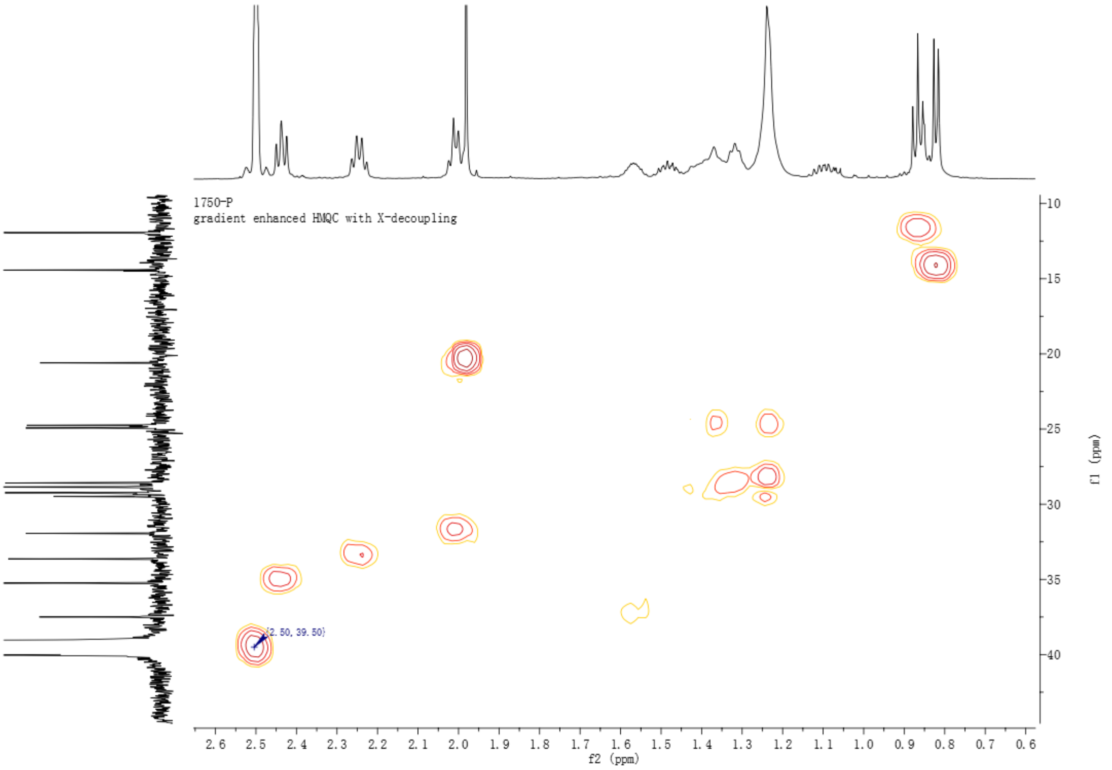

Figure S58. COSY spectrum of peniresorcinoside B (**2**, in DMSO-d_6)

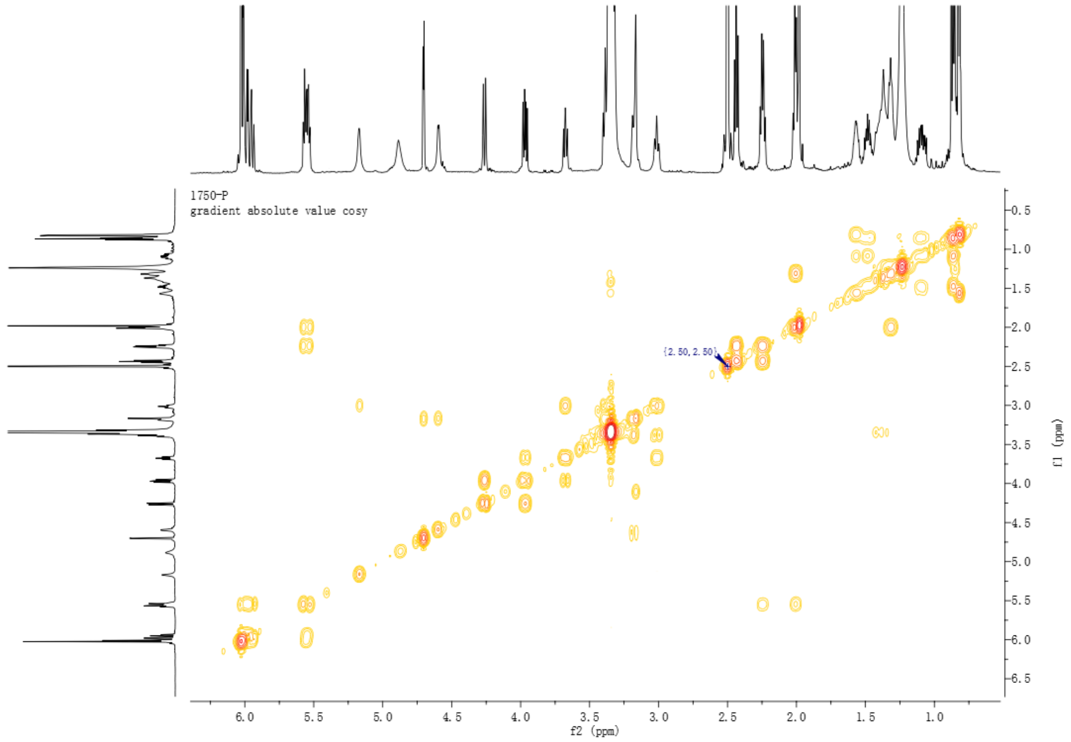

Figure S59. HMBC spectrum of peniresorcinoside B (**2**, in DMSO-d_6)

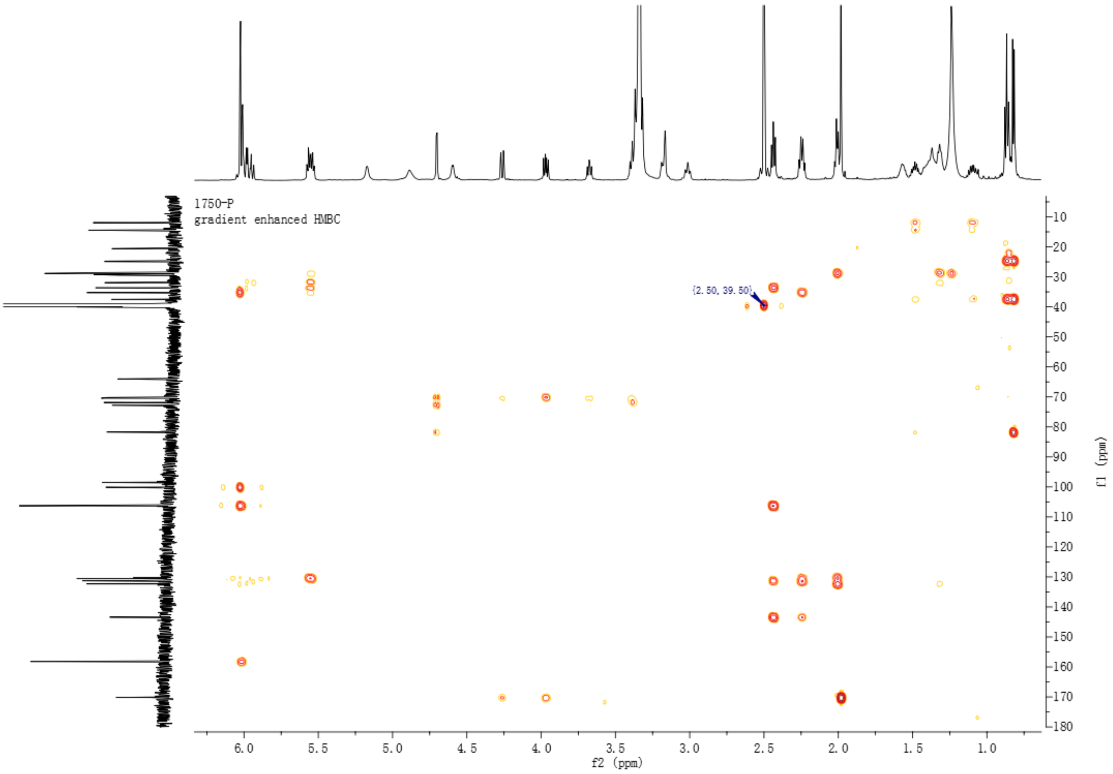

Figure S60. HMBC spectrum of peniresorcinoside B (**2**, in DMSO-d_6)

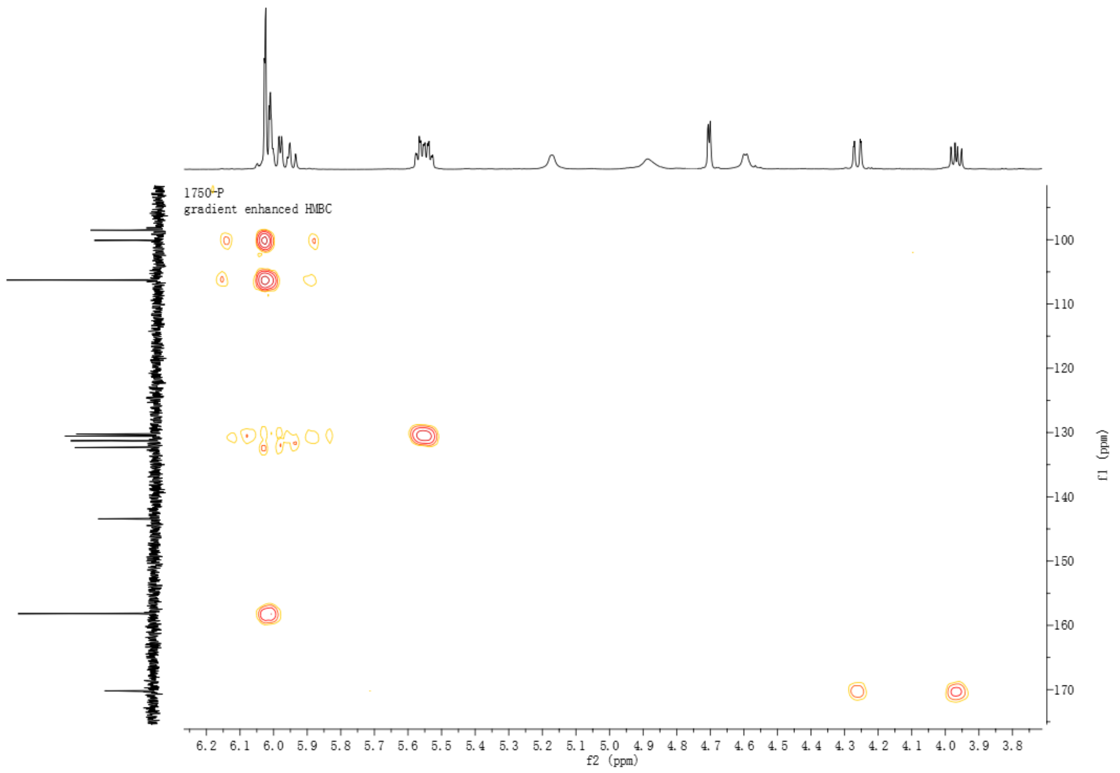

Figure S61. HMBC spectrum of peniresorcinoside B (**2**, in DMSO-d_6)

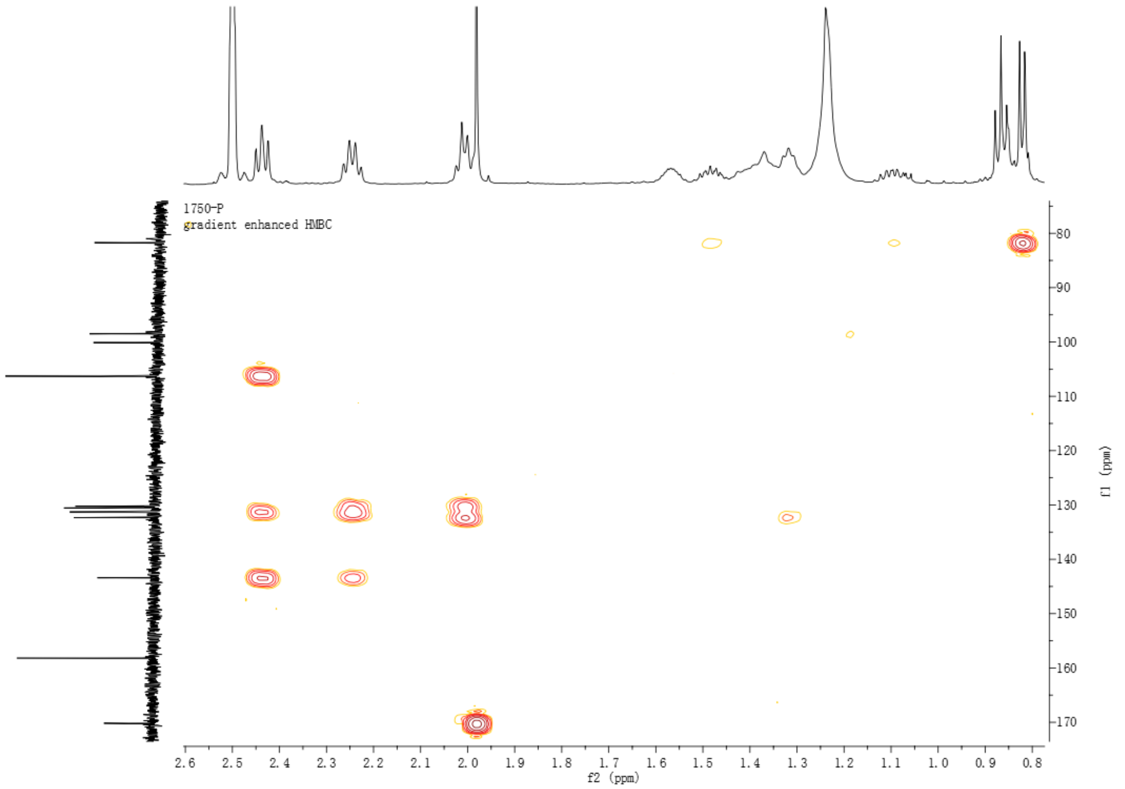

Figure S62. HMBC spectrum of peniresorcinoside B (**2**, in DMSO-d_6)

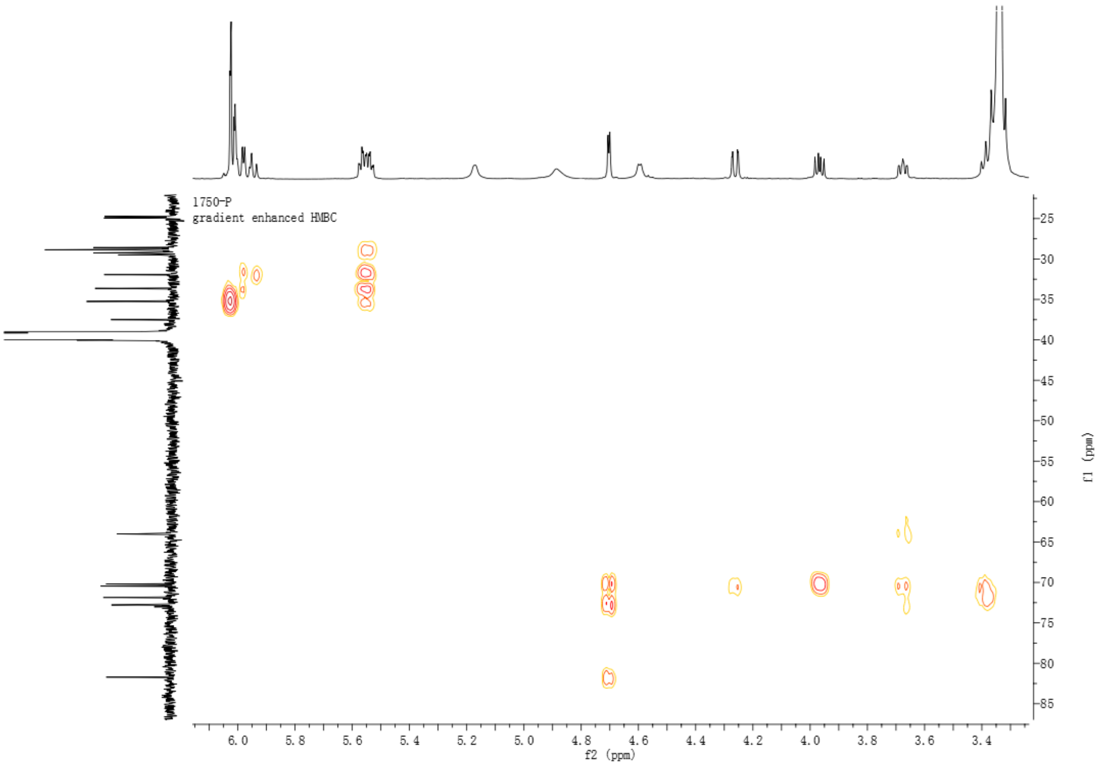

Figure S63. HMBC spectrum of peniresorcinoside B (**2**, in DMSO-d_6)

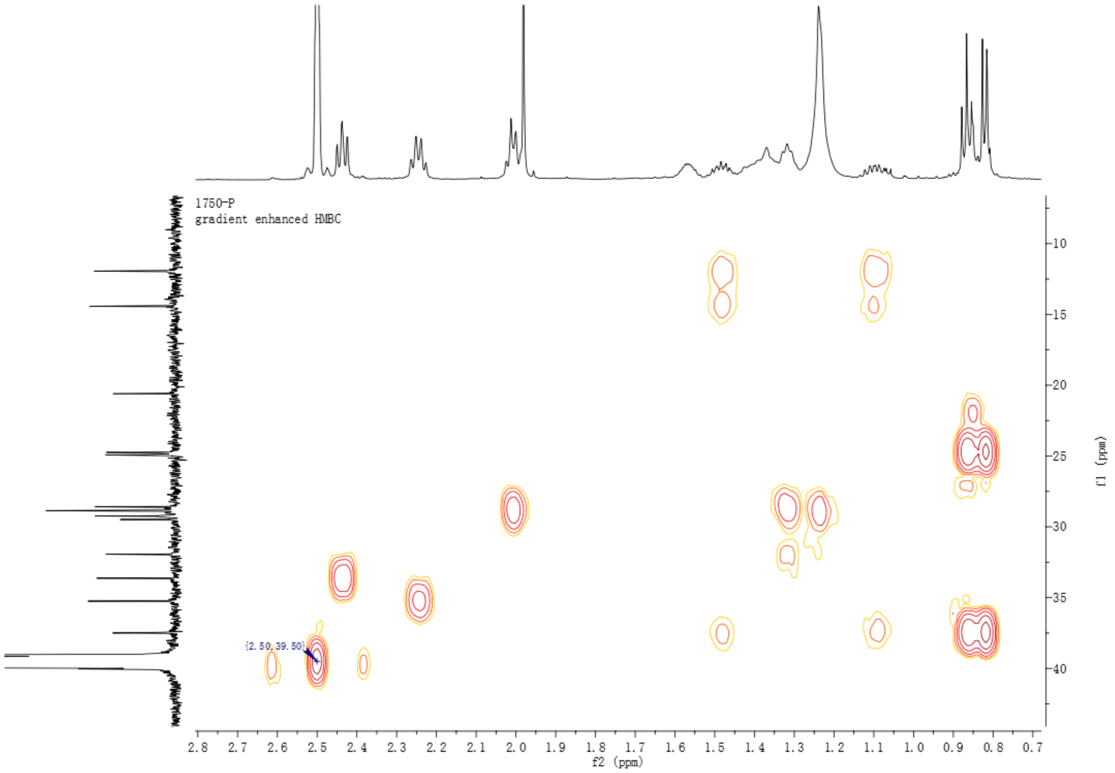

Figure S64. HRESIMS spectrum of peniresorcinoside B (**2**)

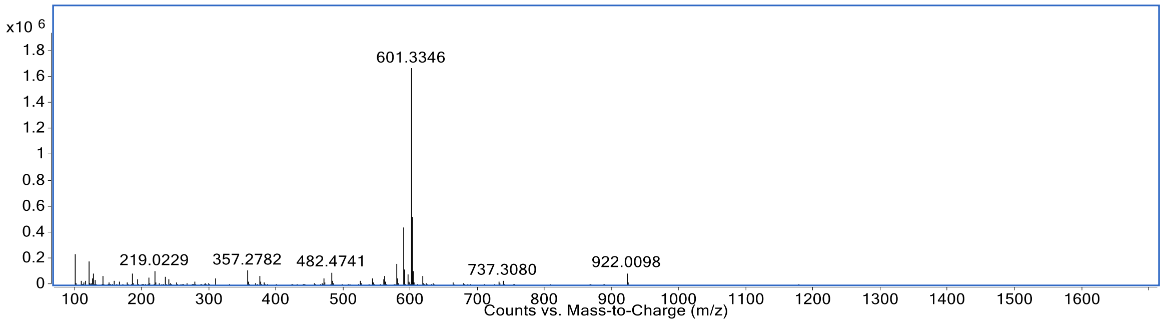

[M+Na]$^{+}$: 601.3346 (calcd for $C_{32}H_{50}NaO_9$, 601.3353).

Figure S65. UV (MeOH) spectrum of peniresorcinoside B (**2**)

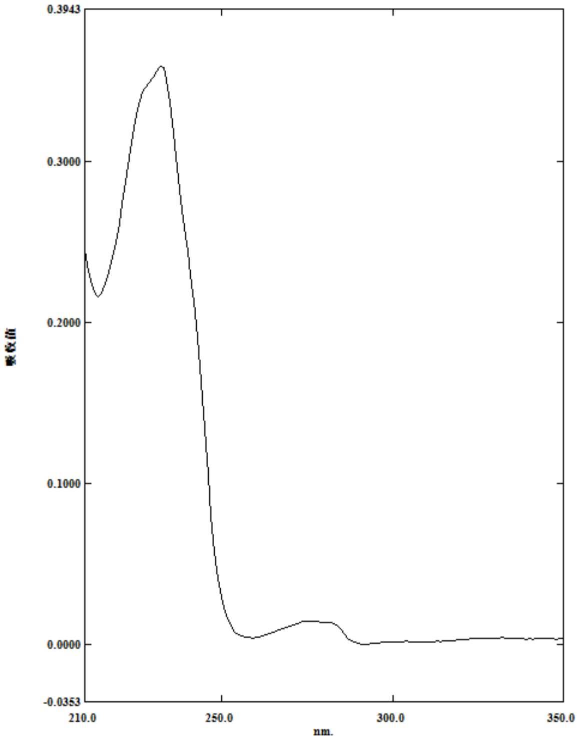

Figure S66. IR (ATR) spectrum of peniresorcinoside B (**2**)

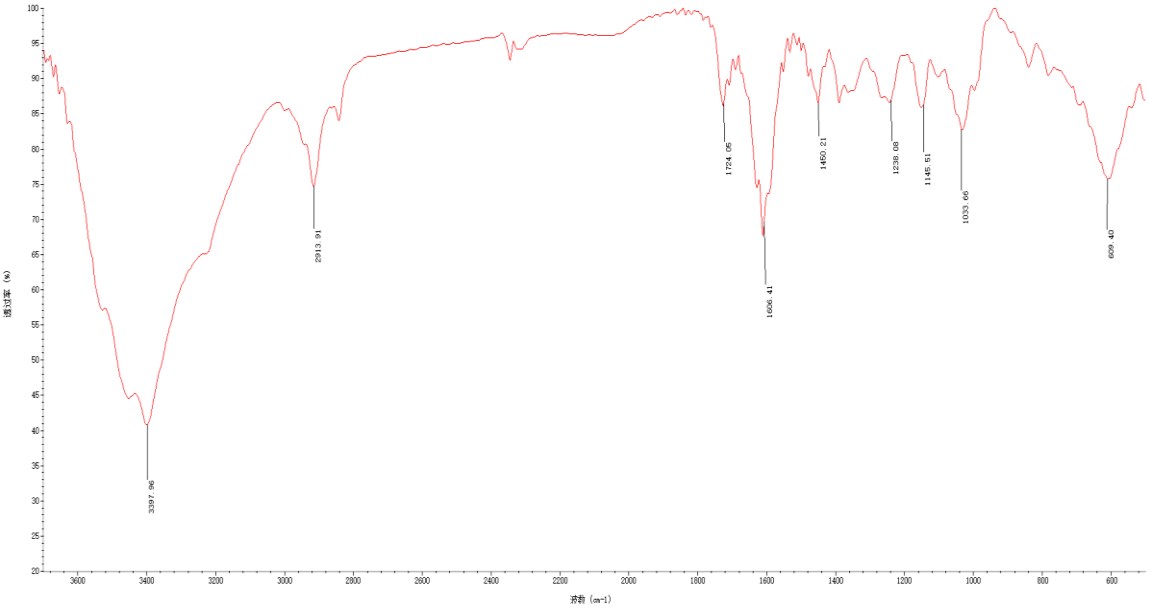

Figure S67. ^{1}H NMR spectrum of peniresorcinoside C (**3**, 600 MHz, in DMSO-d_6)

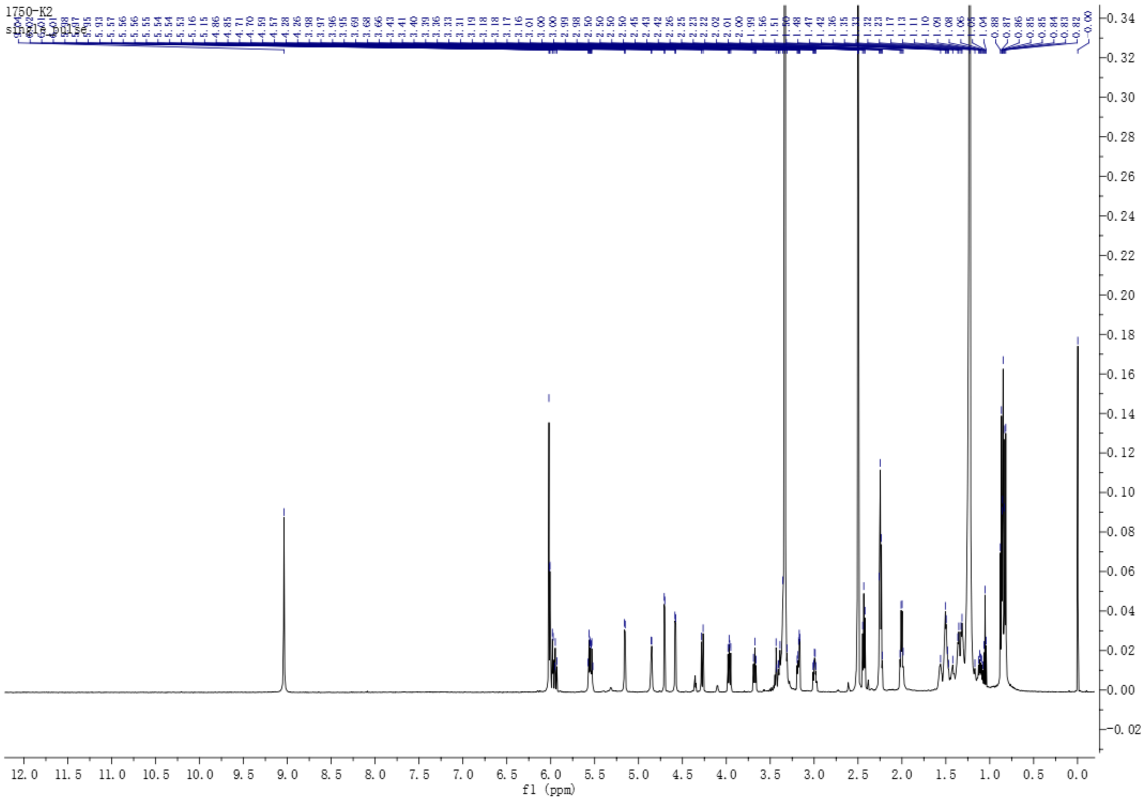

Figure S68. ^{1}H NMR spectrum of peniresorcinoside C (**3**, 600 MHz, in DMSO-d_6)

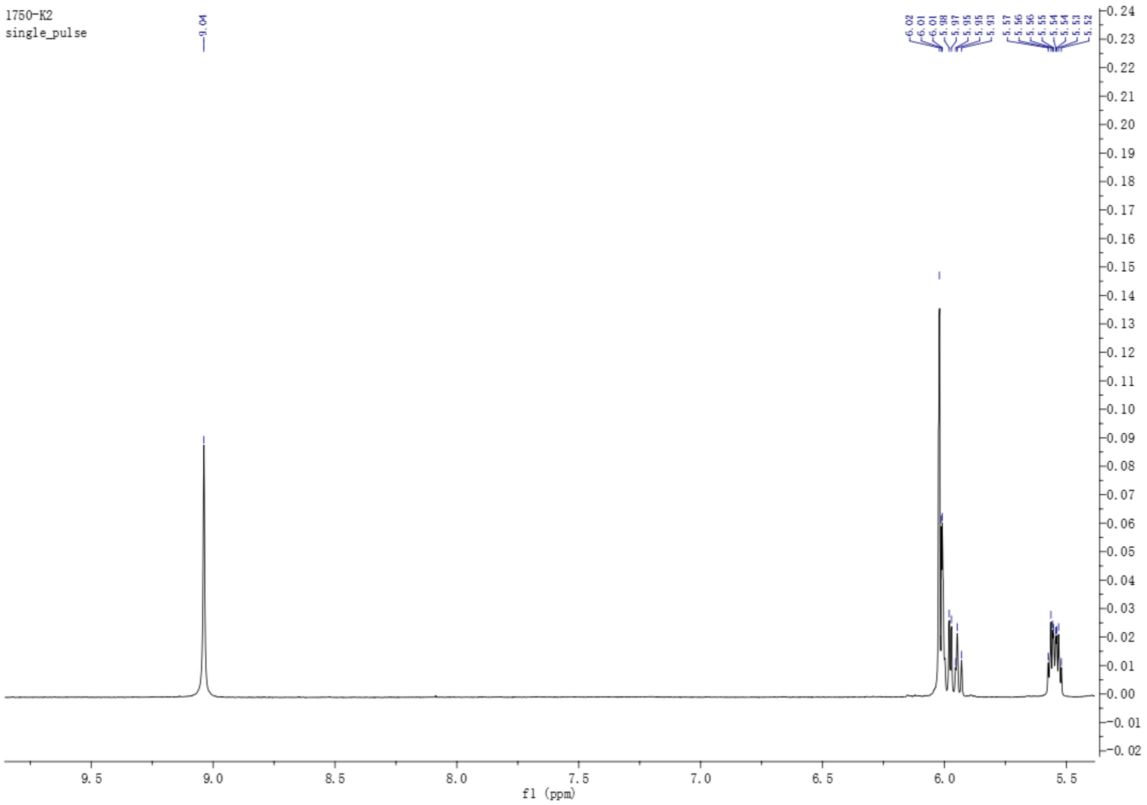

Figure S69. ^{1}H NMR spectrum of peniresorcinoside C (**3**, 600 MHz, in DMSO-d_6)

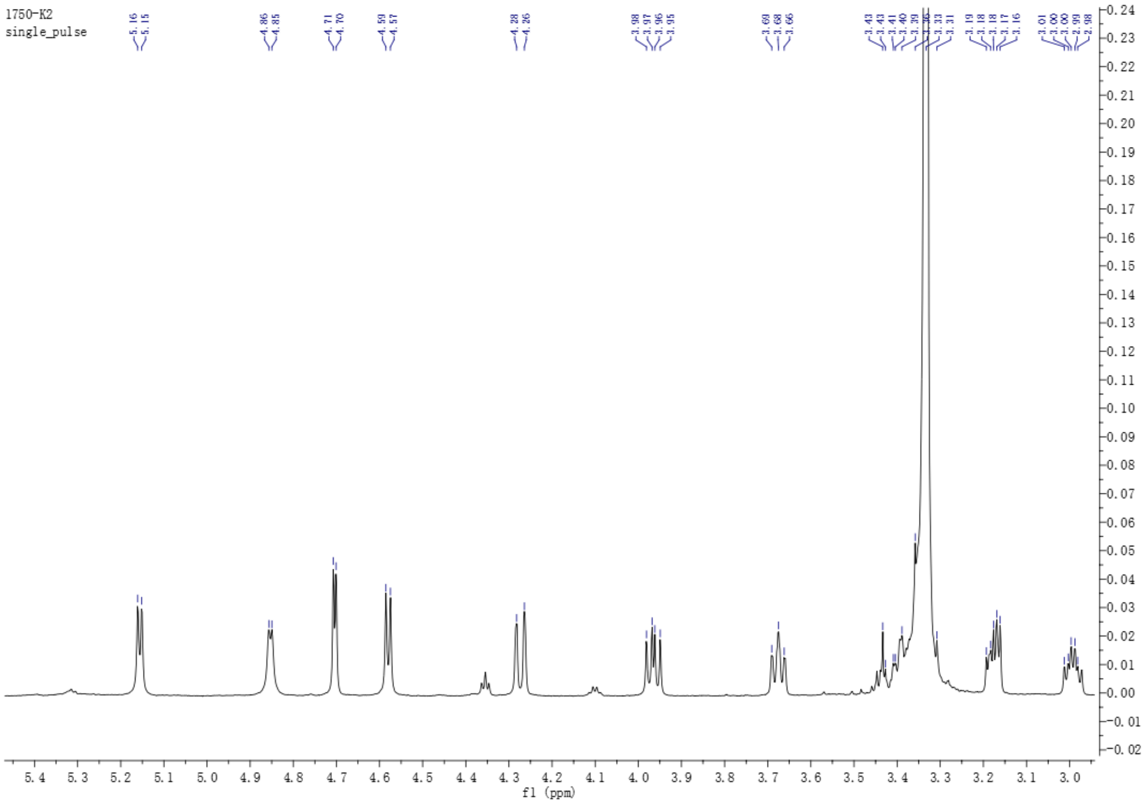

Figure S70. ^{1}H NMR spectrum of peniresorcinoside C (**3**, 600 MHz, in DMSO-d_6)

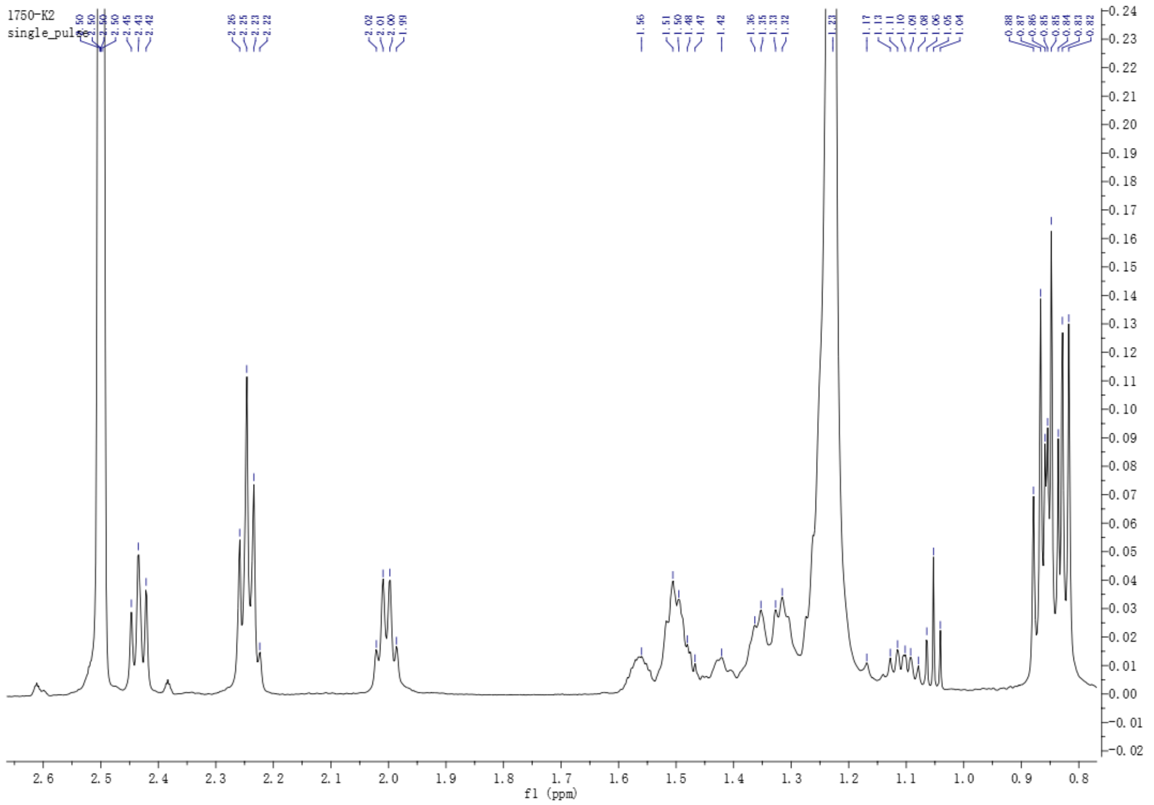

Figure S^{71}. ^{13}C NMR spectrum of peniresorcinoside C (**3**, 150 MHz, in DMSO-d_6)

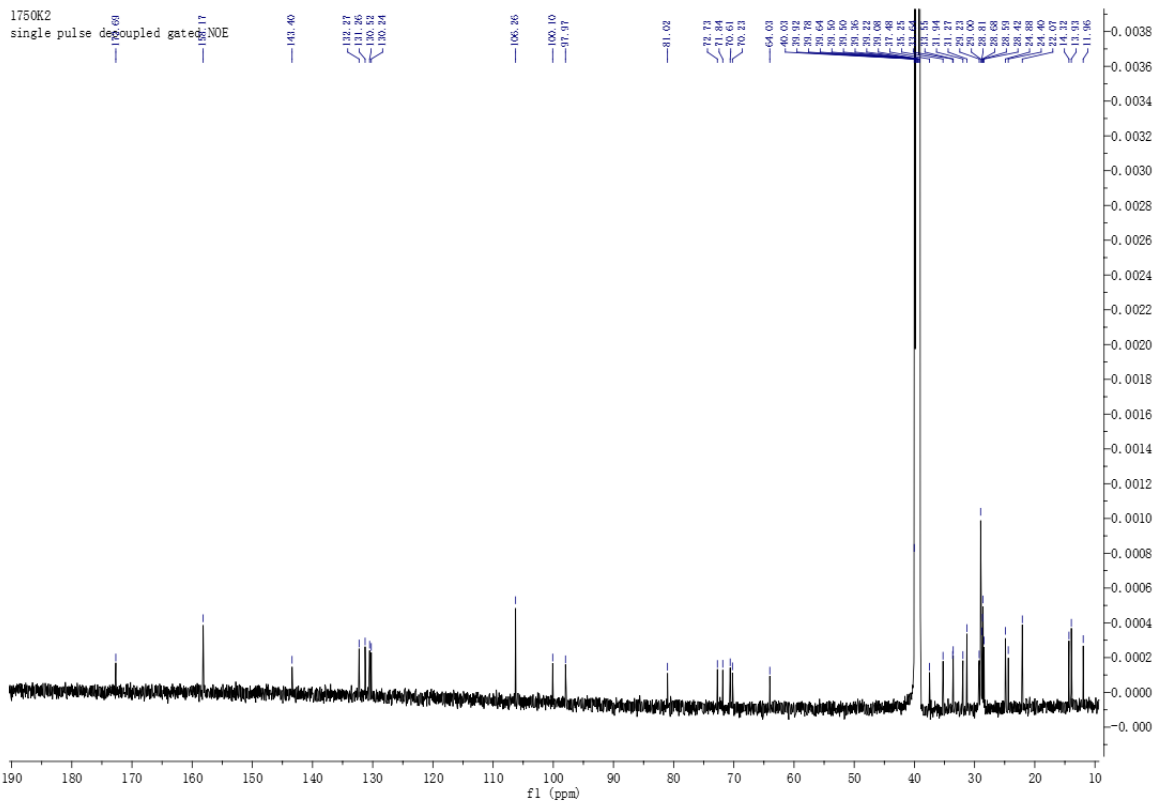

Figure S72. ^{13}C NMR spectrum of peniresorcinoside C (**3**, 150 MHz, in DMSO-d_6)

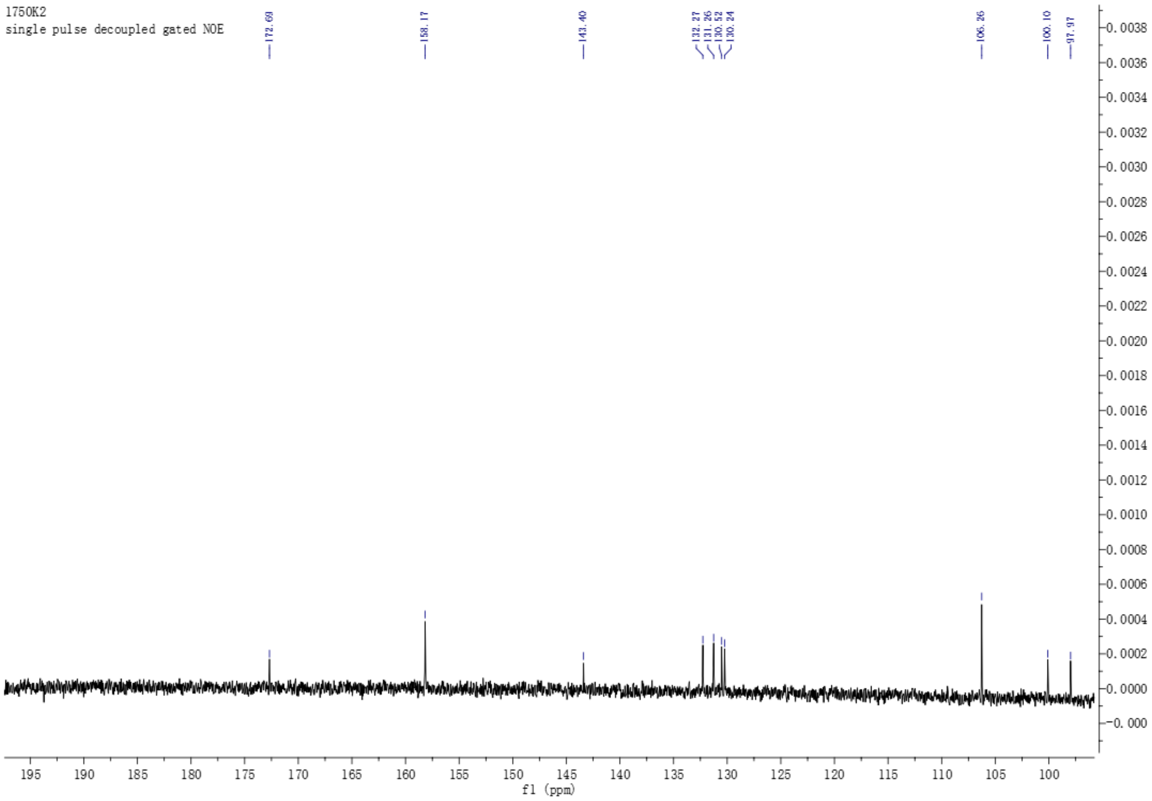

Figure S73. ^{13}C NMR spectrum of peniresorcinoside C (**3**, 150 MHz, in DMSO-d_6)

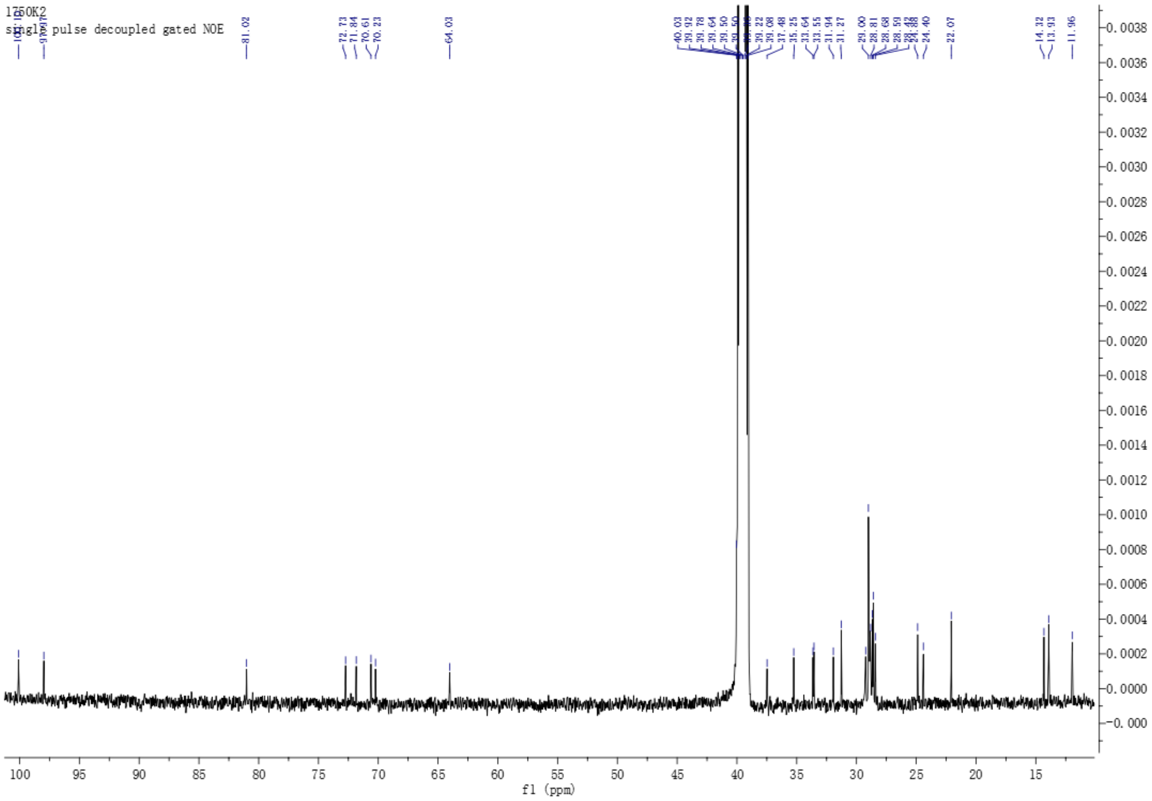

Figure S74. HMQC spectrum of peniresorcinoside C (**3**, in DMSO-d_6)

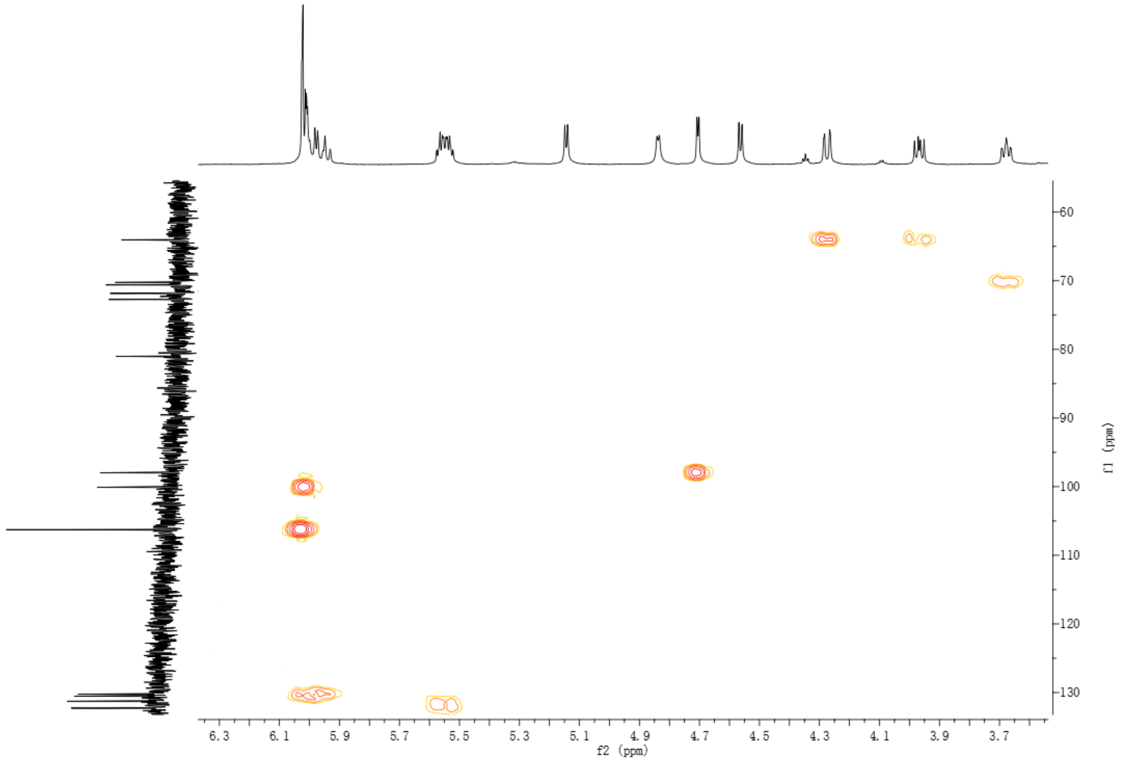

Figure S75. HMQC spectrum of peniresorcinoside C (**3**, in DMSO-d_6)

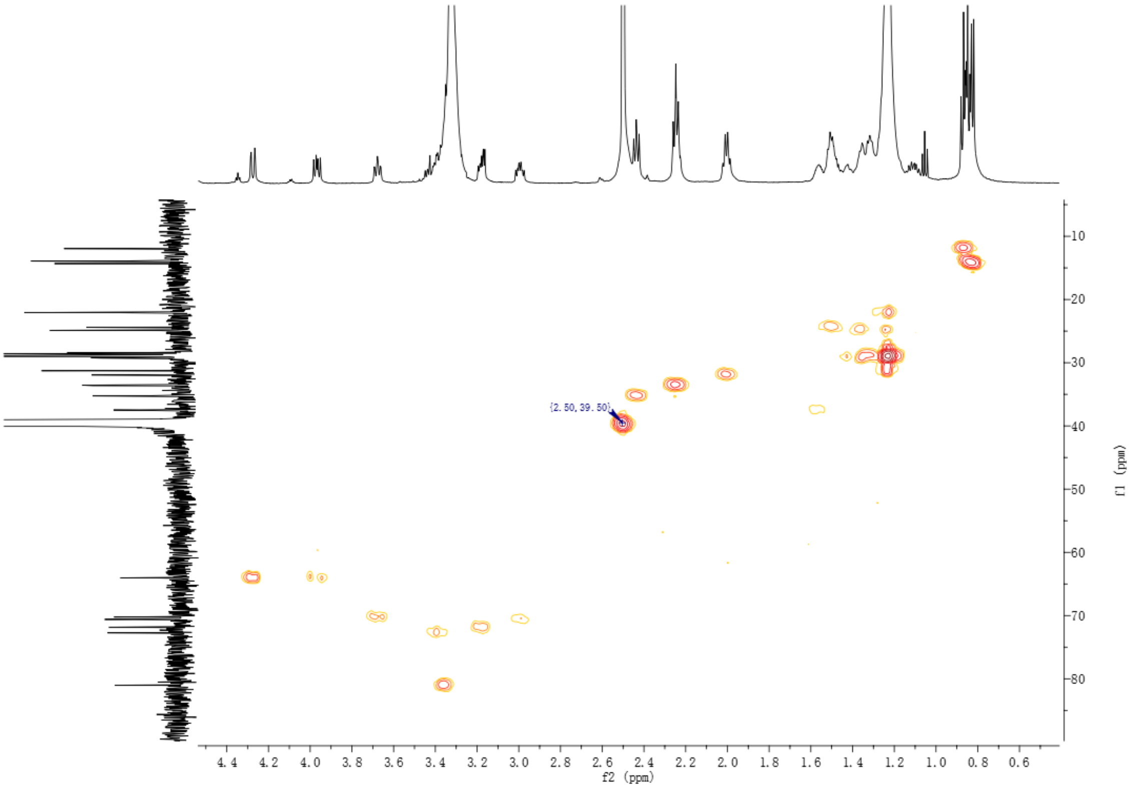

Figure S76. COSY spectrum of peniresorcinoside C (**3**, in DMSO-d_6)

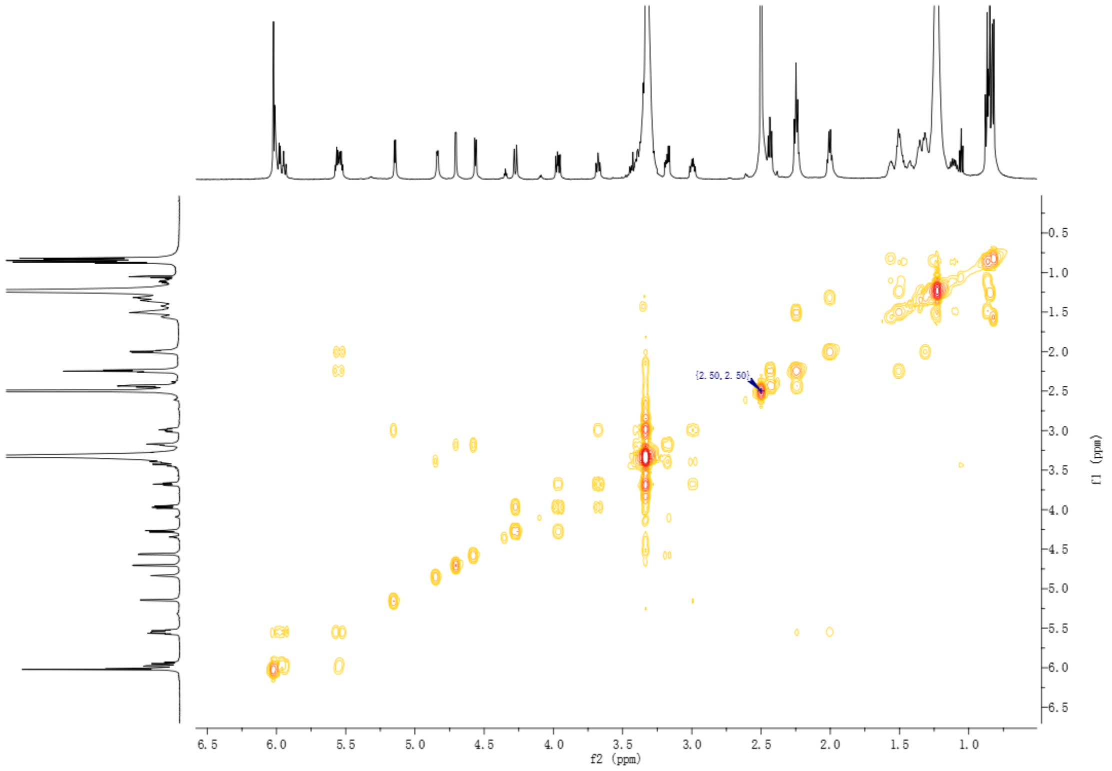

Figure S77. HMBC spectrum of peniresorcinoside C (**3**, in DMSO-d_6)

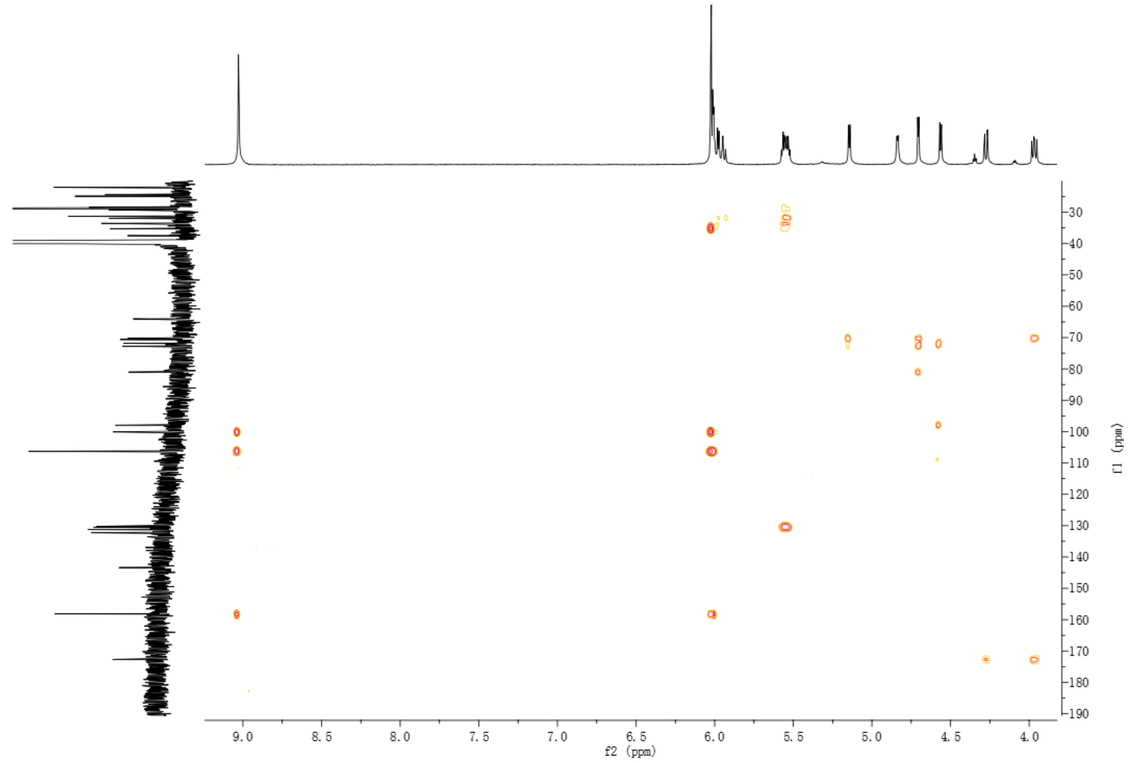

Figure S78. HMBC spectrum of peniresorcinoside C (**3**, in DMSO-d_6)

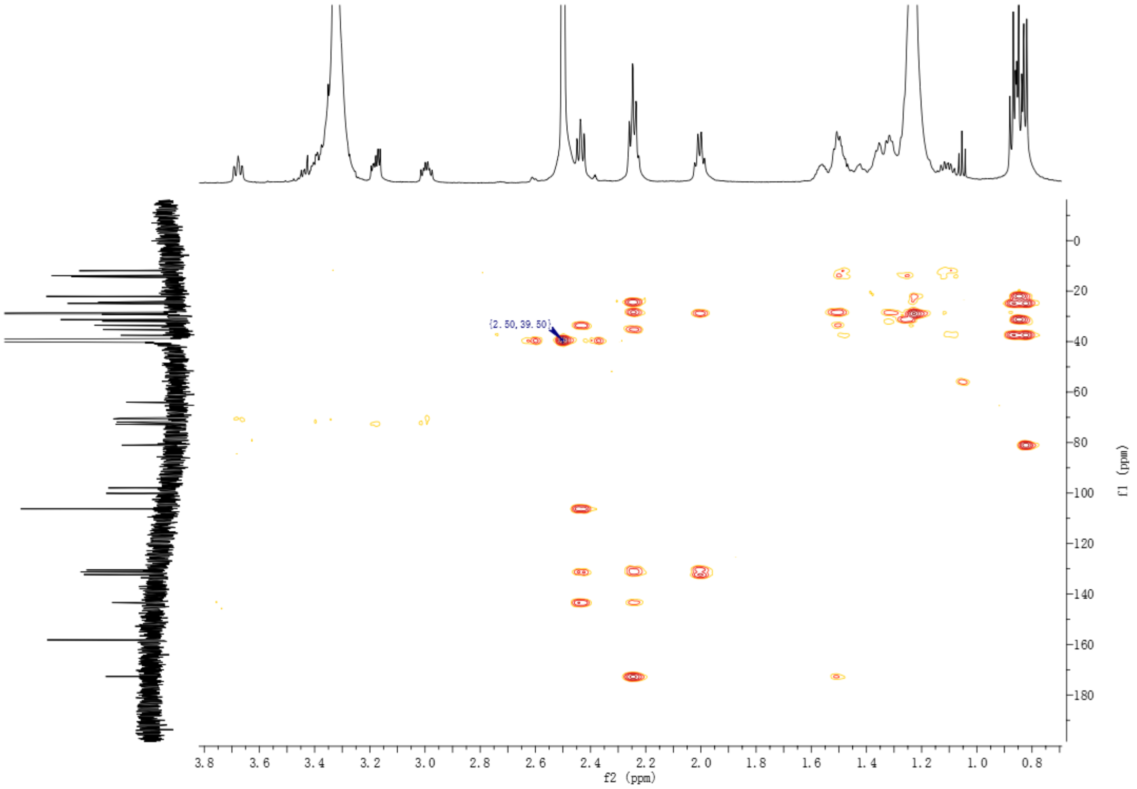

Figure S79. HRESIMS spectrum of peniresorcinoside C (**3**)

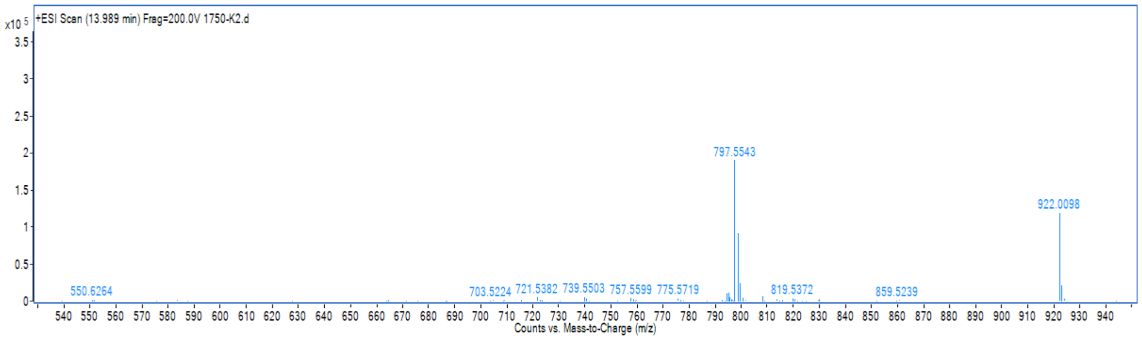

[M+H]$^+$: 775.5719 (calcd for $C_{46}H_{79}O_9$, 775.5724); [M+Na]$^+$: 797.5543 (calcd for $C_{46}H_{78}NaO_9$, 797.5544).

Figure S80. UV (MeOH) spectrum of peniresorcinoside C (**3**)

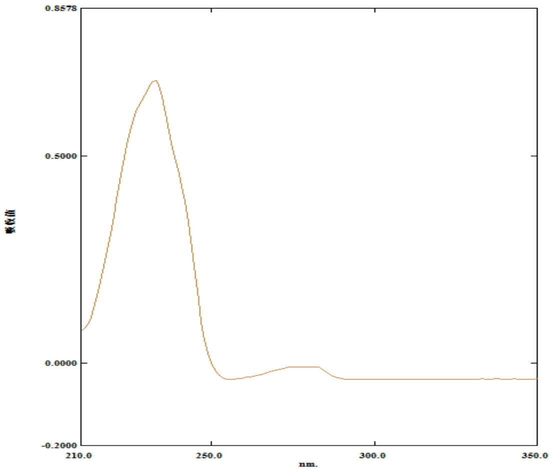

Figure S81. IR (ATR) spectrum of peniresorcinoside C (**3**)

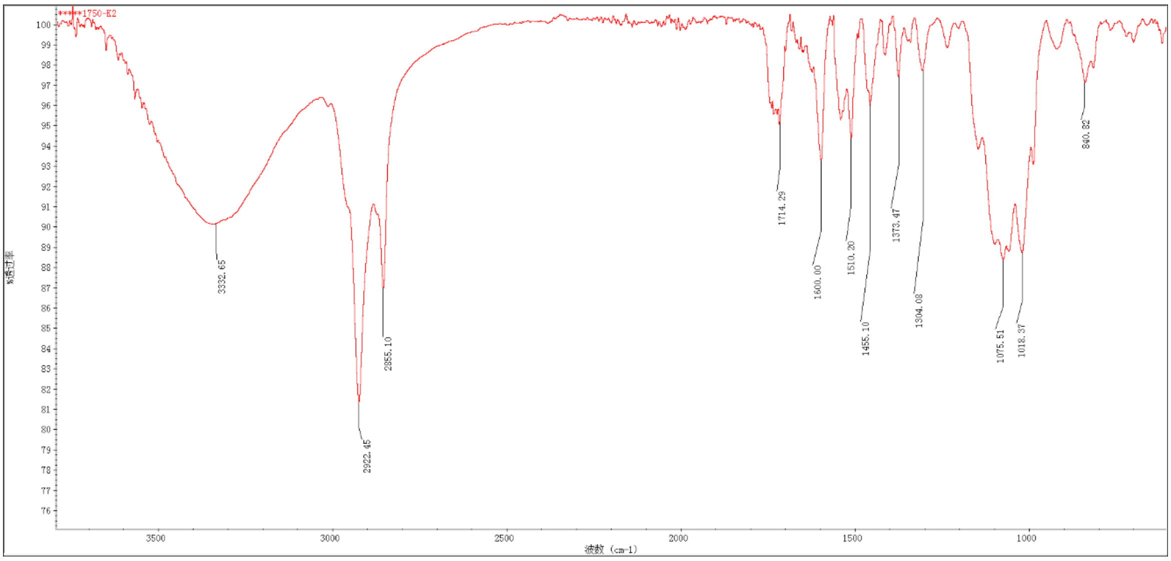

Figure S82. ^{1}H NMR spectrum of peniresorcinoside D (**4**, 600 MHz, in DMSO-d_6)

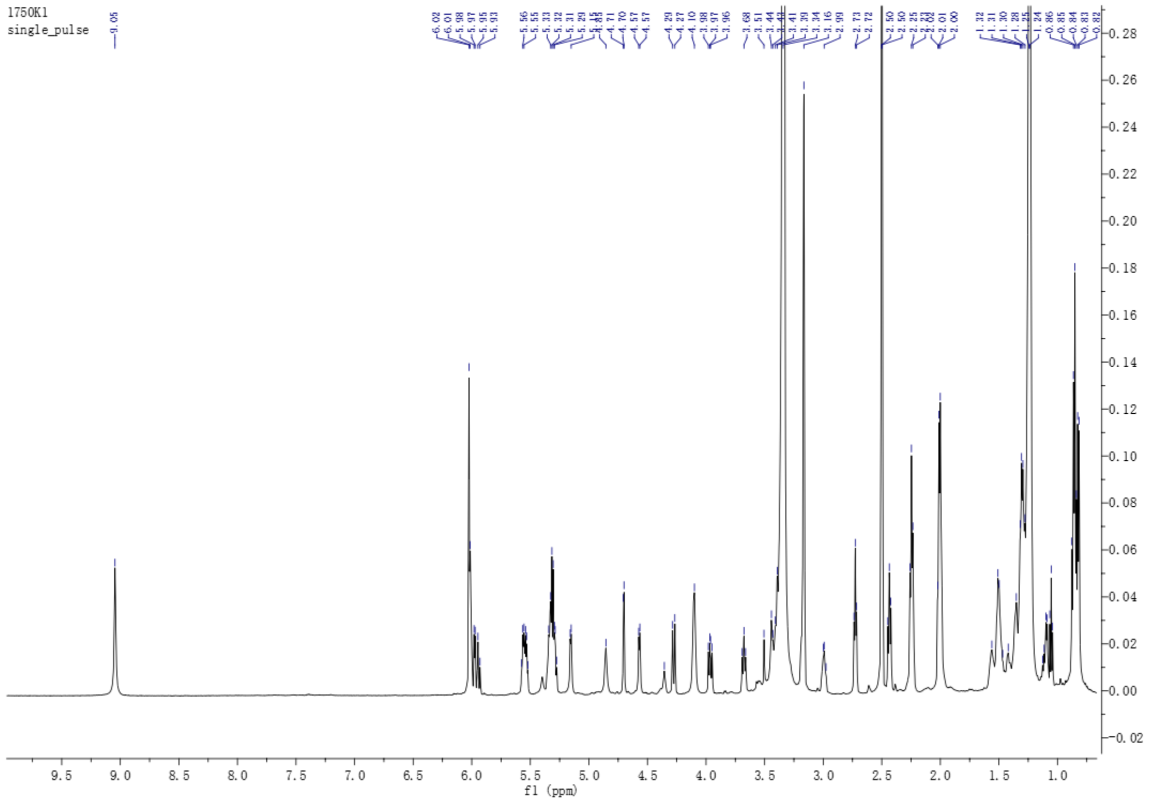

Figure S83. ^{1}H NMR spectrum of peniresorcinoside D (**4**, 600 MHz, in DMSO-d_6)

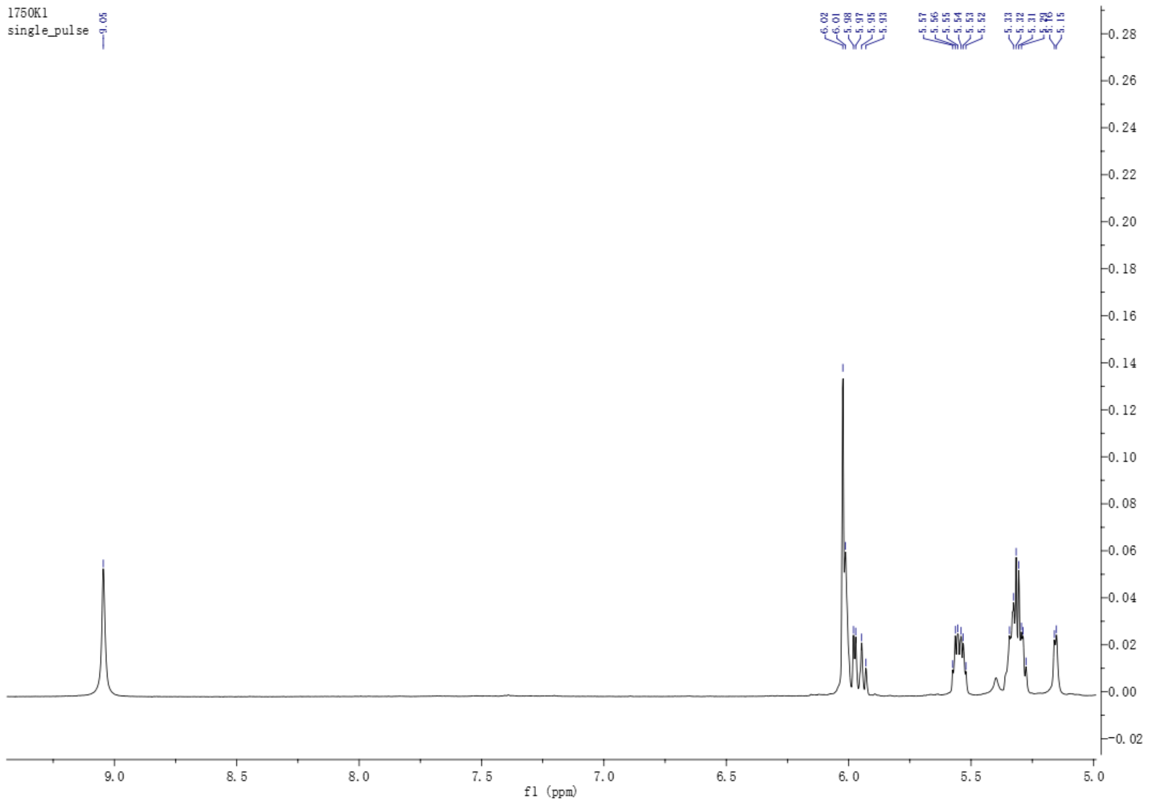

Figure S84. ^{1}H NMR spectrum of peniresorcinoside D (**4**, 600 MHz, in DMSO-d_6)

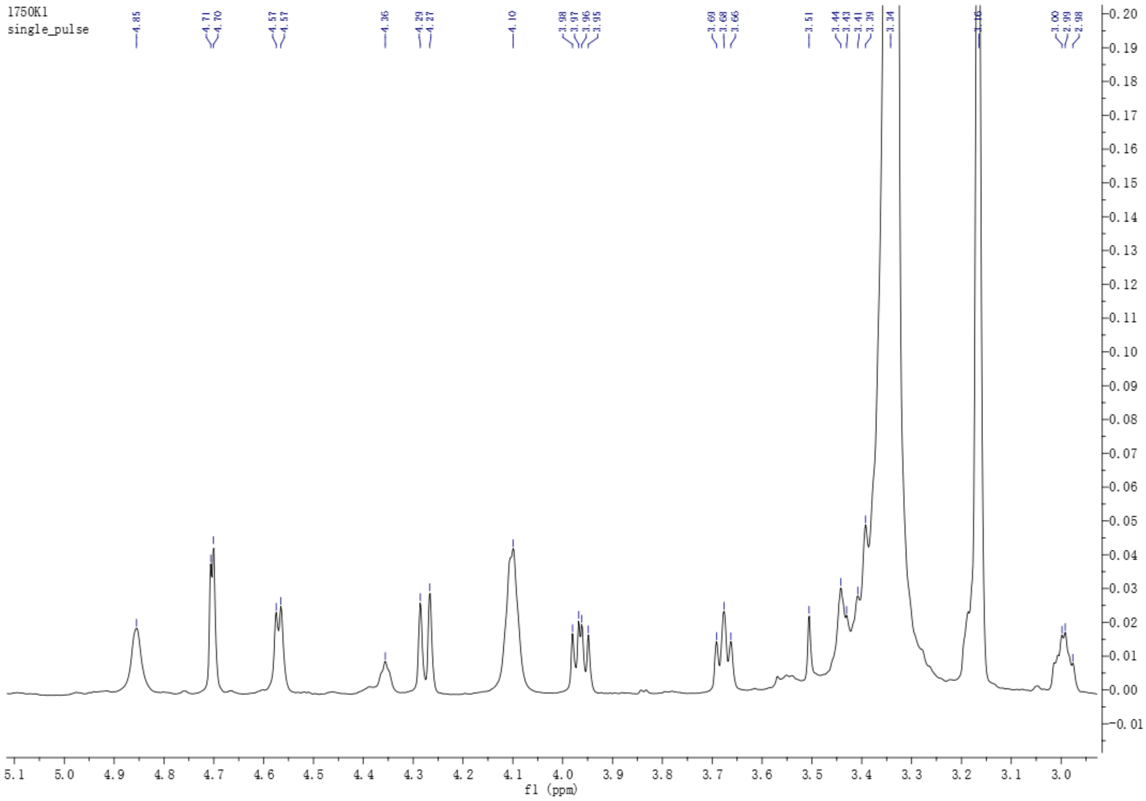

Figure S85. ^{1}H NMR spectrum of peniresorcinoside D (**4**, 600 MHz, in DMSO-d_6)

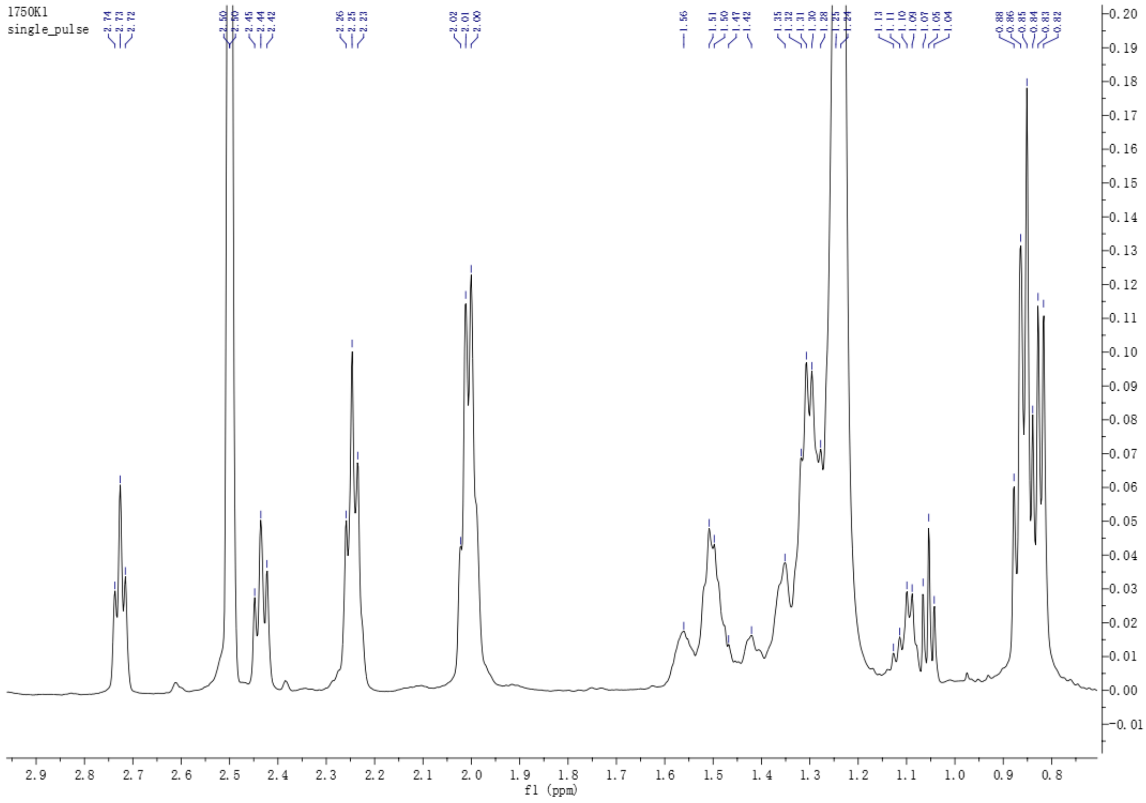

Figure S86. ^{13}C NMR spectrum of peniresorcinoside D (**4**, 150 MHz, in DMSO-d_6)

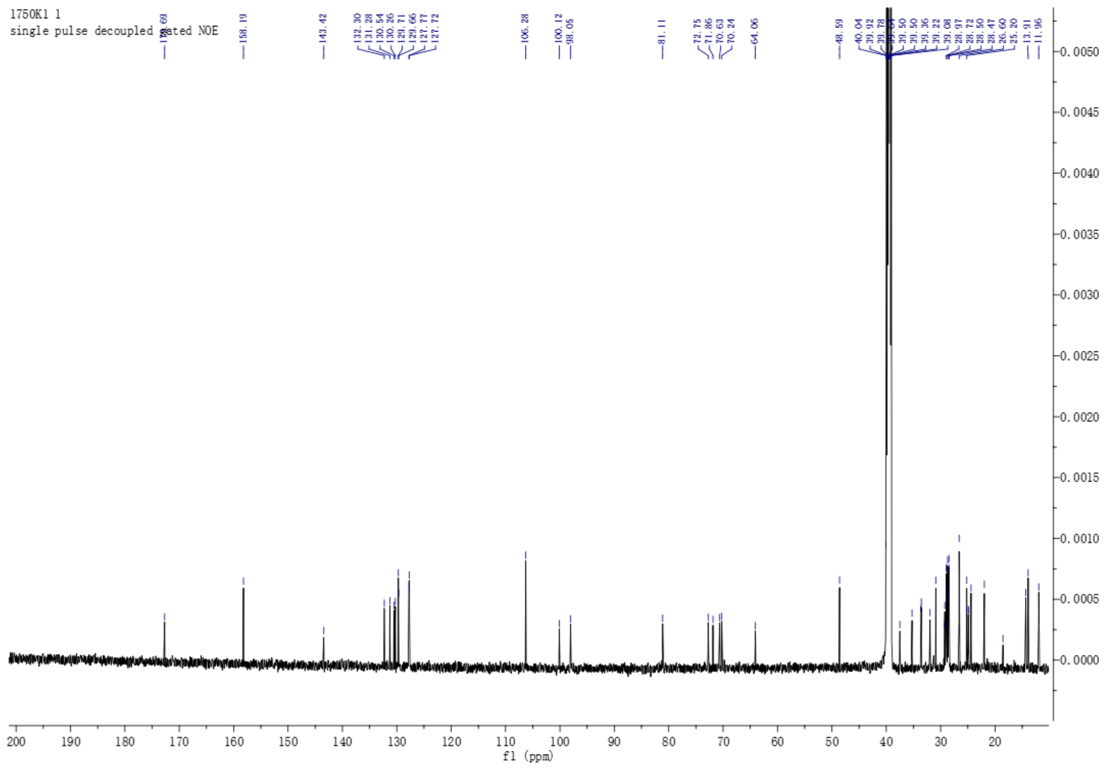

Figure S87. ^{13}C NMR spectrum of peniresorcinoside D (**4**, 150 MHz, in DMSO-d_6)

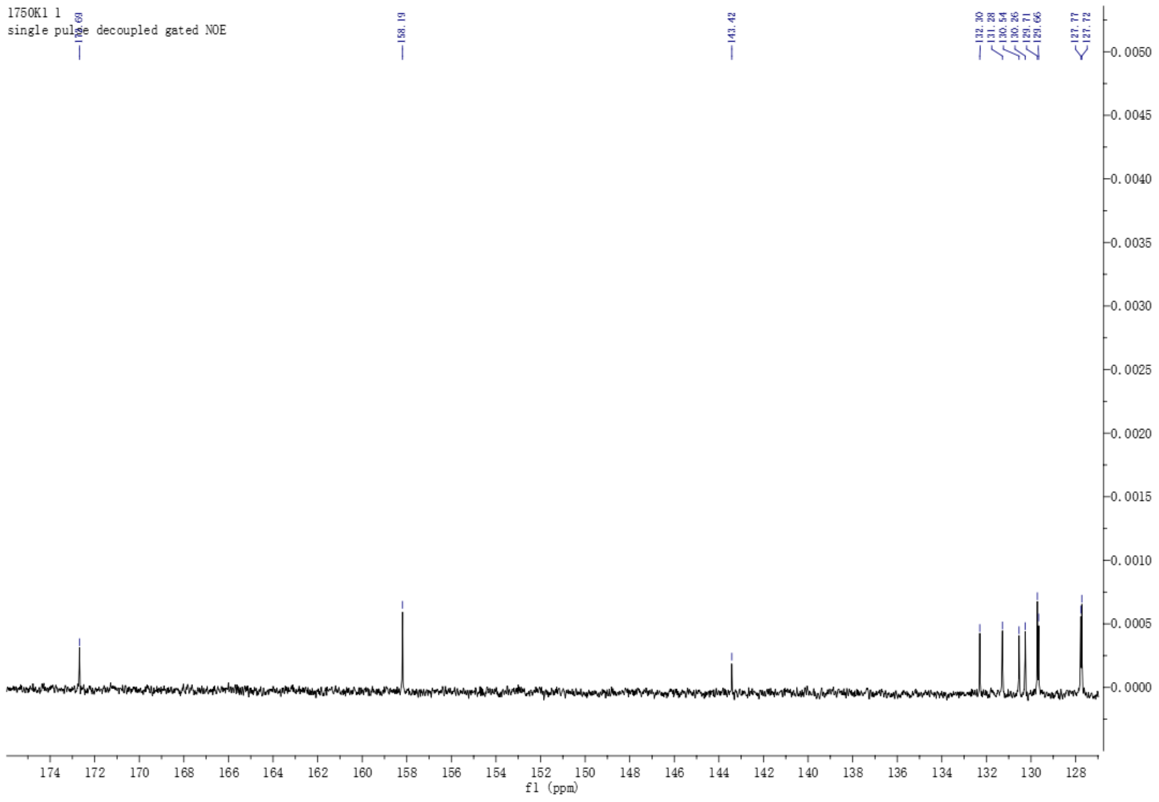

Figure S88. ^{13}C NMR spectrum of peniresorcinoside D (**4**, 150 MHz, in DMSO-d_6)

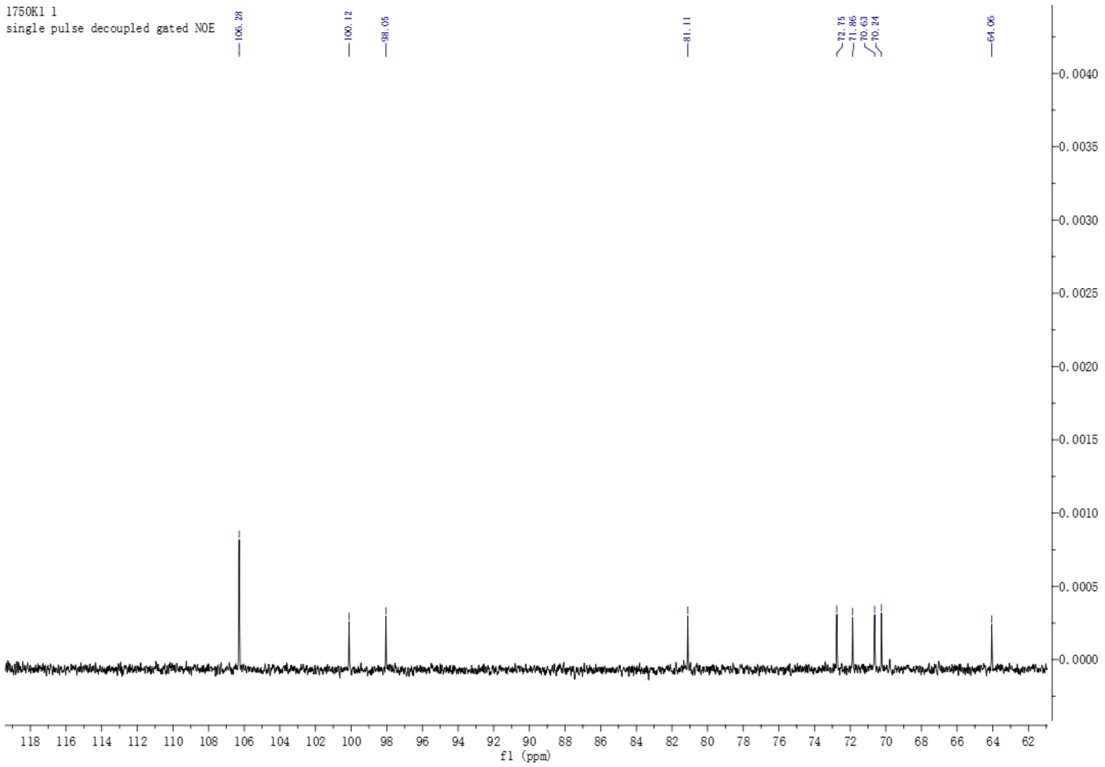

Figure S89. ^{13}C NMR spectrum of peniresorcinoside D (**4**, 150 MHz, in DMSO-d_6)

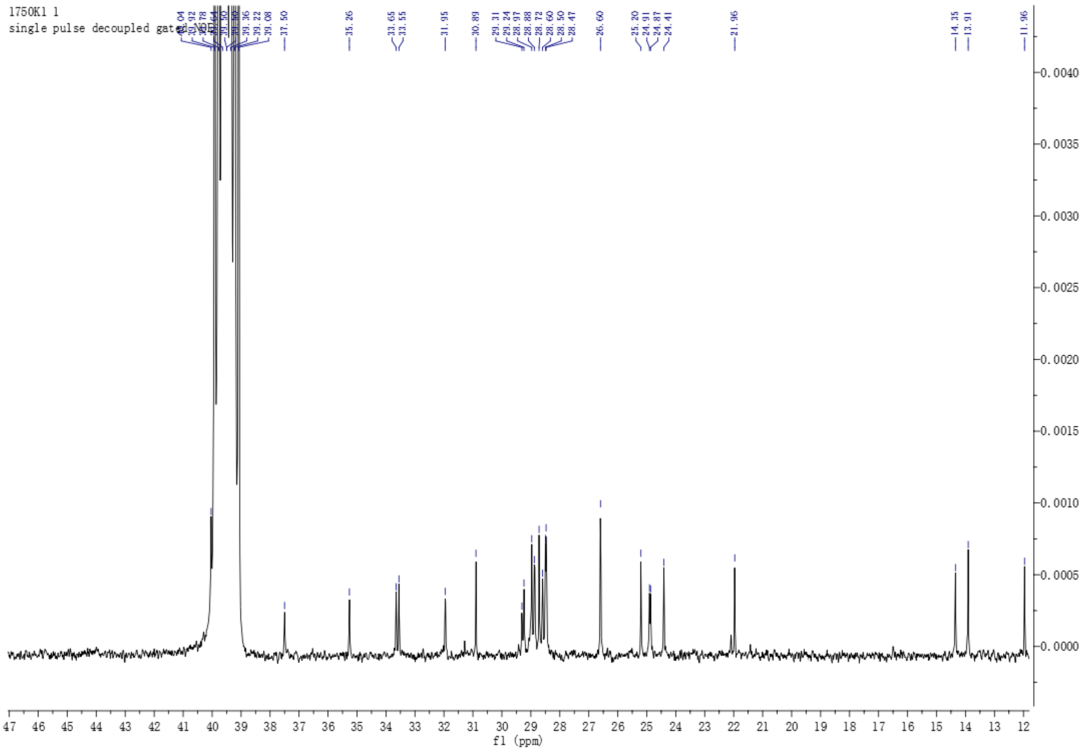

Figure S90. HMQC spectrum of peniresorcinoside D (**4**, in DMSO-d_6)

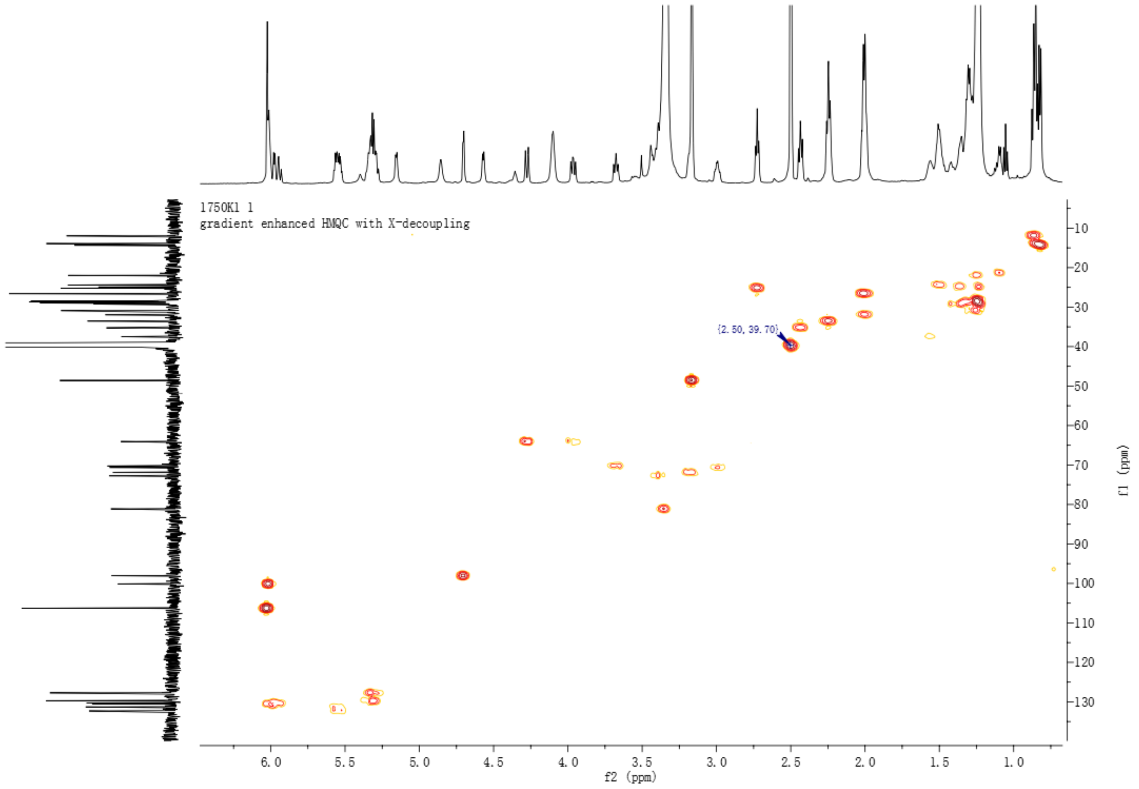

Figure S91. HMQC spectrum of peniresorcinoside D (**4**, in DMSO-d_6)

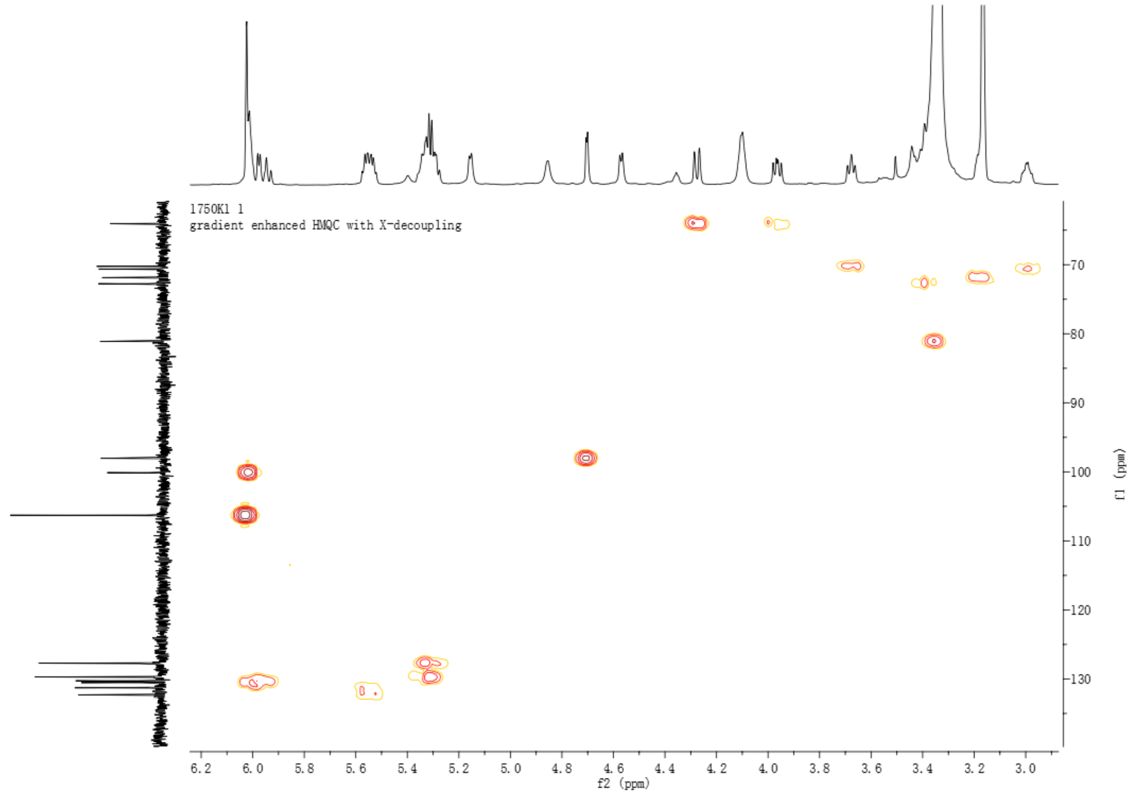

Figure S92. HMQC spectrum of peniresorcinoside D (**4**, in DMSO-d_6)

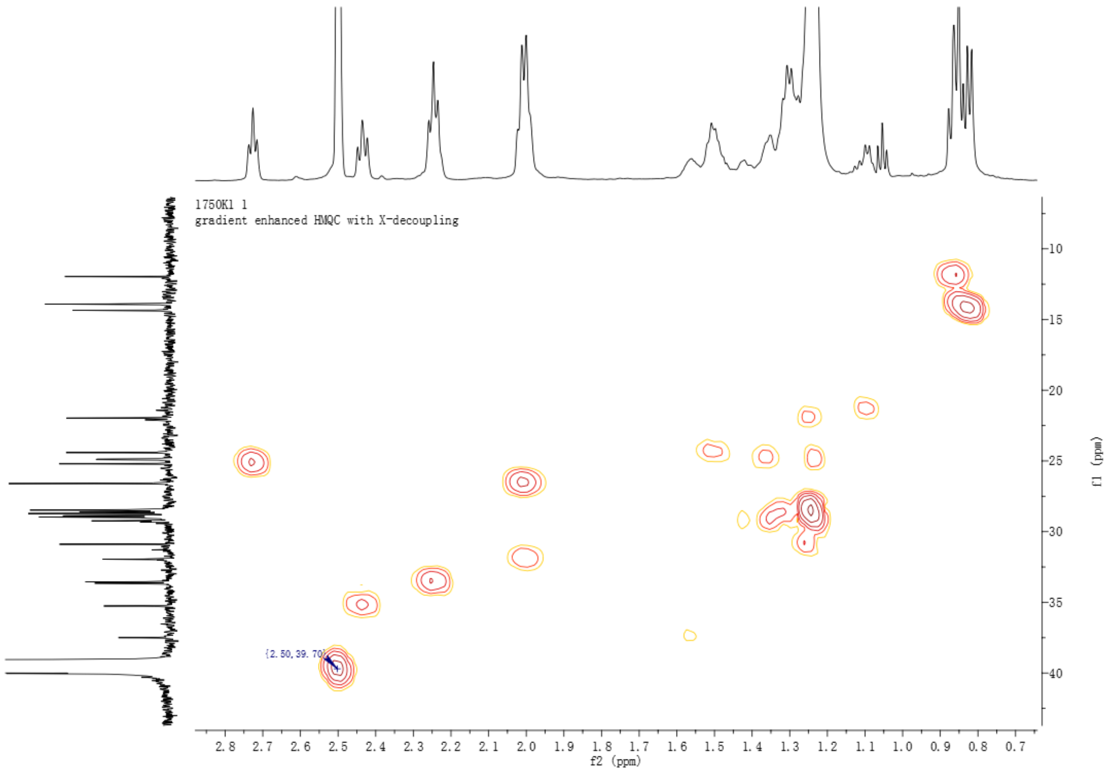

Figure S93. COSY spectrum of peniresorcinoside D (**4**, in DMSO-d_6)

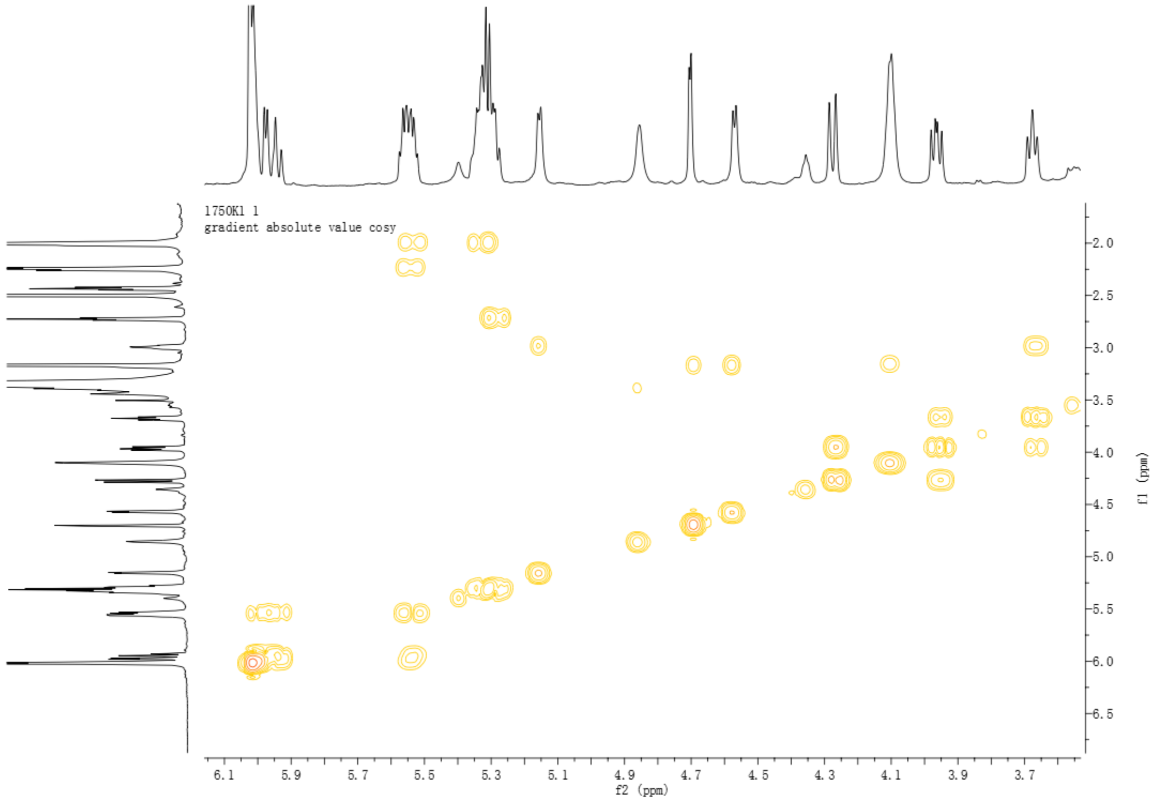

Figure S94. COSY spectrum of peniresorcinoside D (**4**, in DMSO-d_6)

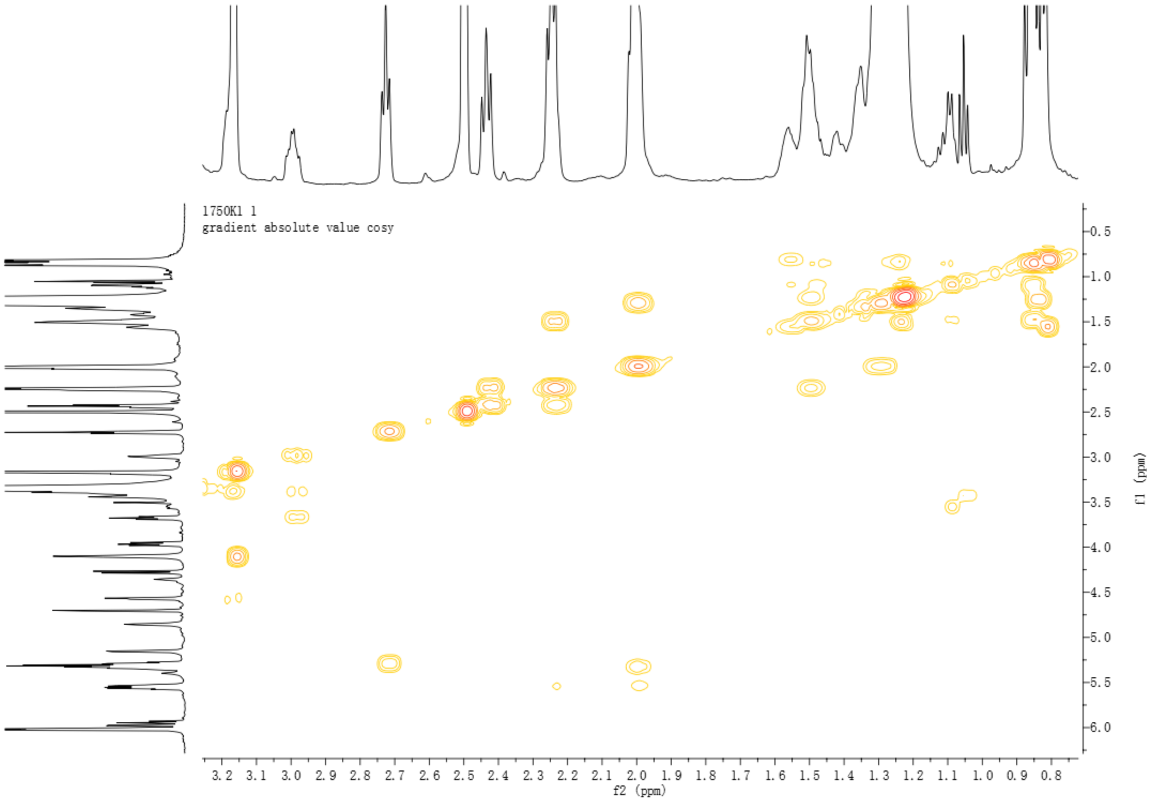

Figure S95. HMBC spectrum of peniresorcinoside D (**4**, in DMSO-d_6)

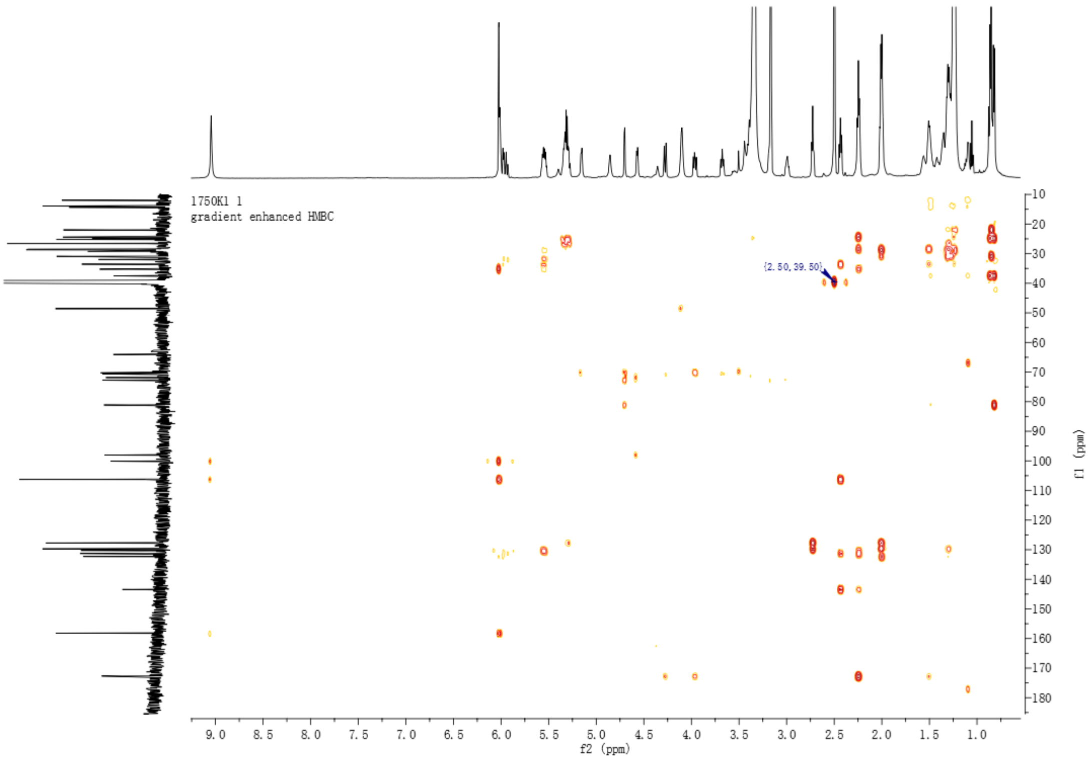

Figure S96. HMBC spectrum of peniresorcinoside D (**4**, in DMSO-d_6)

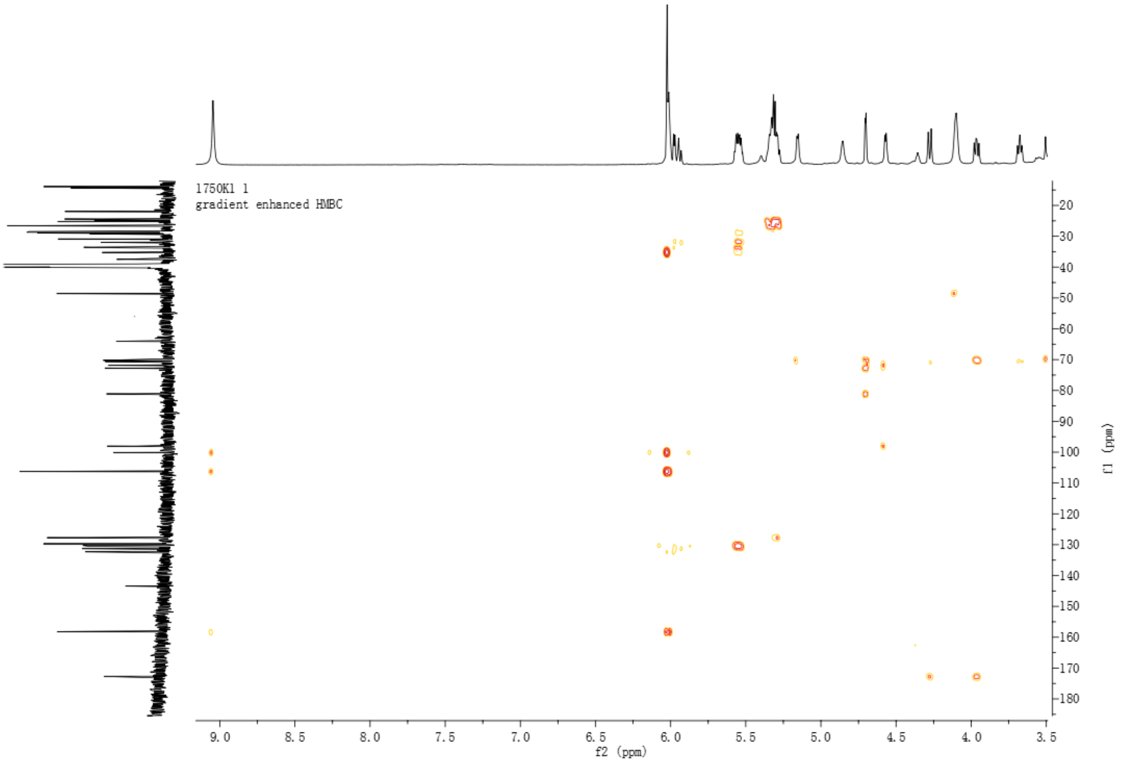

Figure S97. HMBC spectrum of peniresorcinoside D (**4**, in DMSO-d_6)

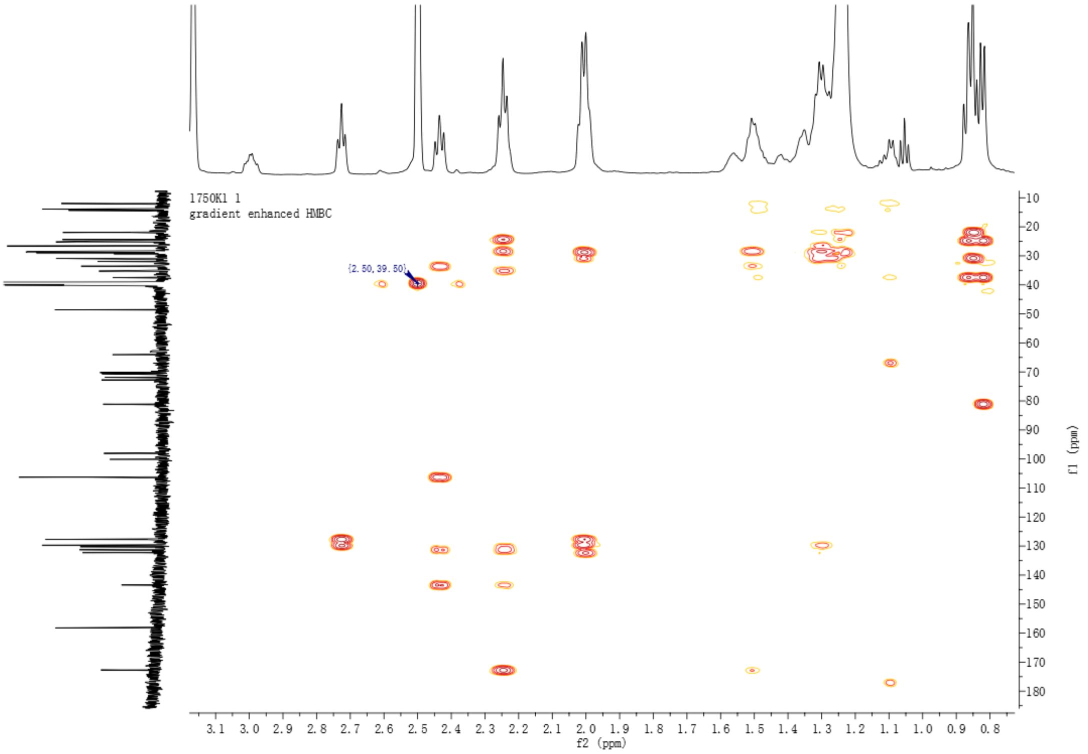

Figure S98. HRESIMS spectrum of peniresorcinoside D (**4**)

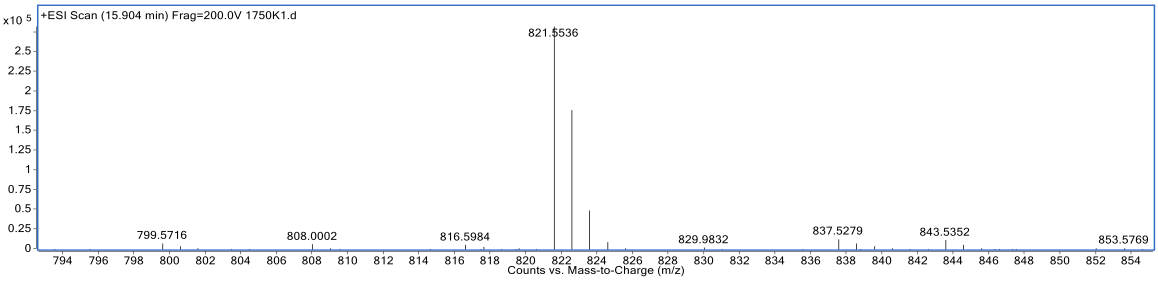

[M+H]$^+$: 799.5716 (calcd for $C_{48}H_{79}O_9$, 799.5724); [M+Na]$^+$: 821.5536 (calcd for $C_{48}H_{78}NaO_9$, 821.5544).

Figure S99. UV (MeOH) spectrum of peniresorcinoside D (**4**)

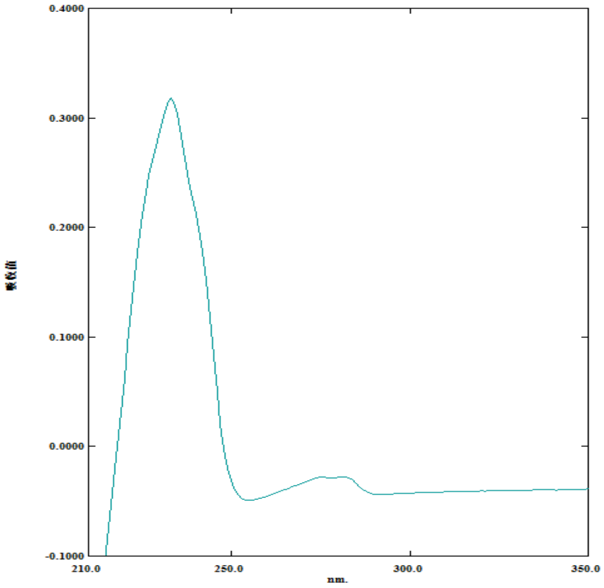

Figure S100. IR (ATR) spectrum of peniresorcinoside D (**4**)

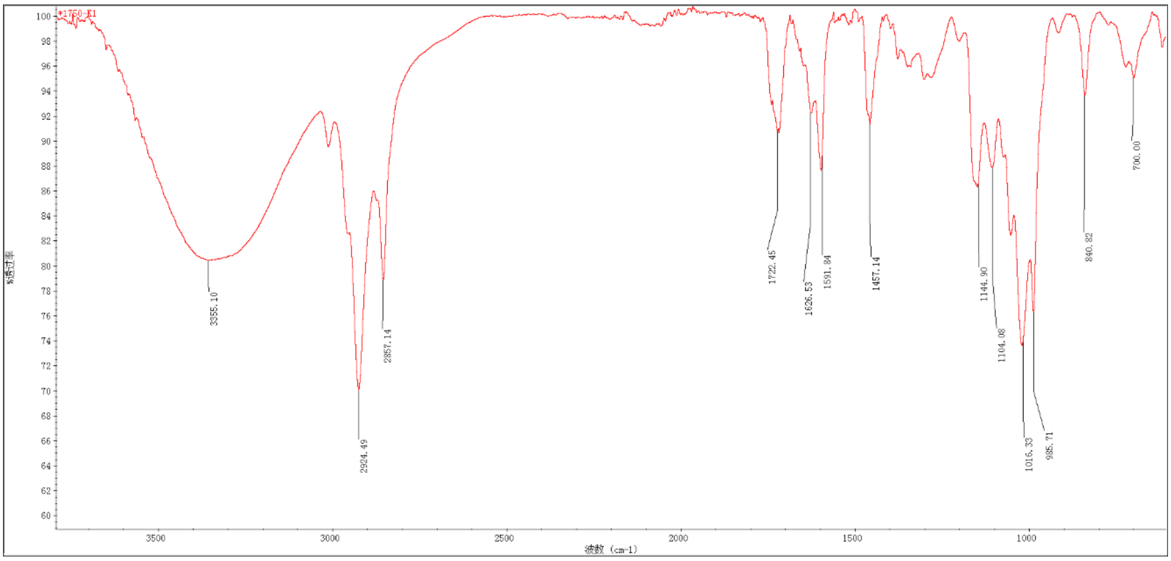

Figure S101. ^{1}H NMR spectrum of peniresorcinoside E (**5**, 600 MHz, in DMSO-d_6)

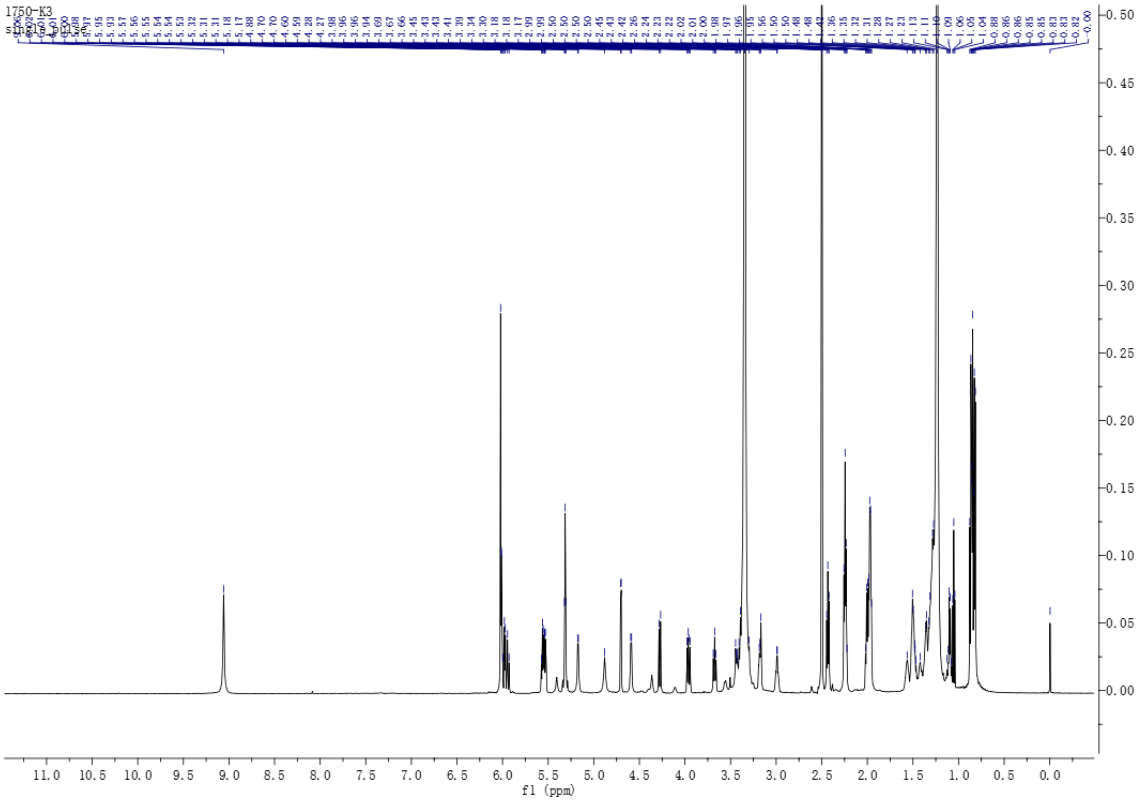

Figure S102. ^{1}H NMR spectrum of peniresorcinoside E (**5**, 600 MHz, in DMSO-d_6)

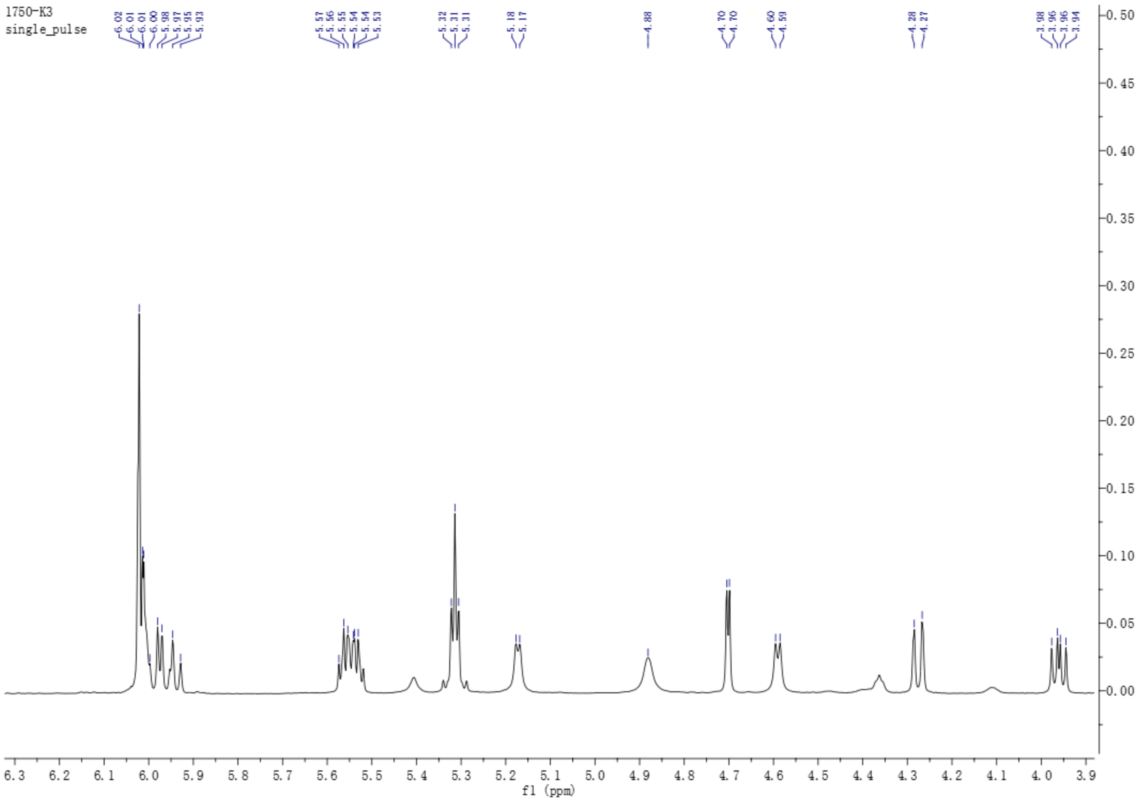

Figure S103. ^{1}H NMR spectrum of peniresorcinoside E (**5**, 600 MHz, in DMSO-d_6)

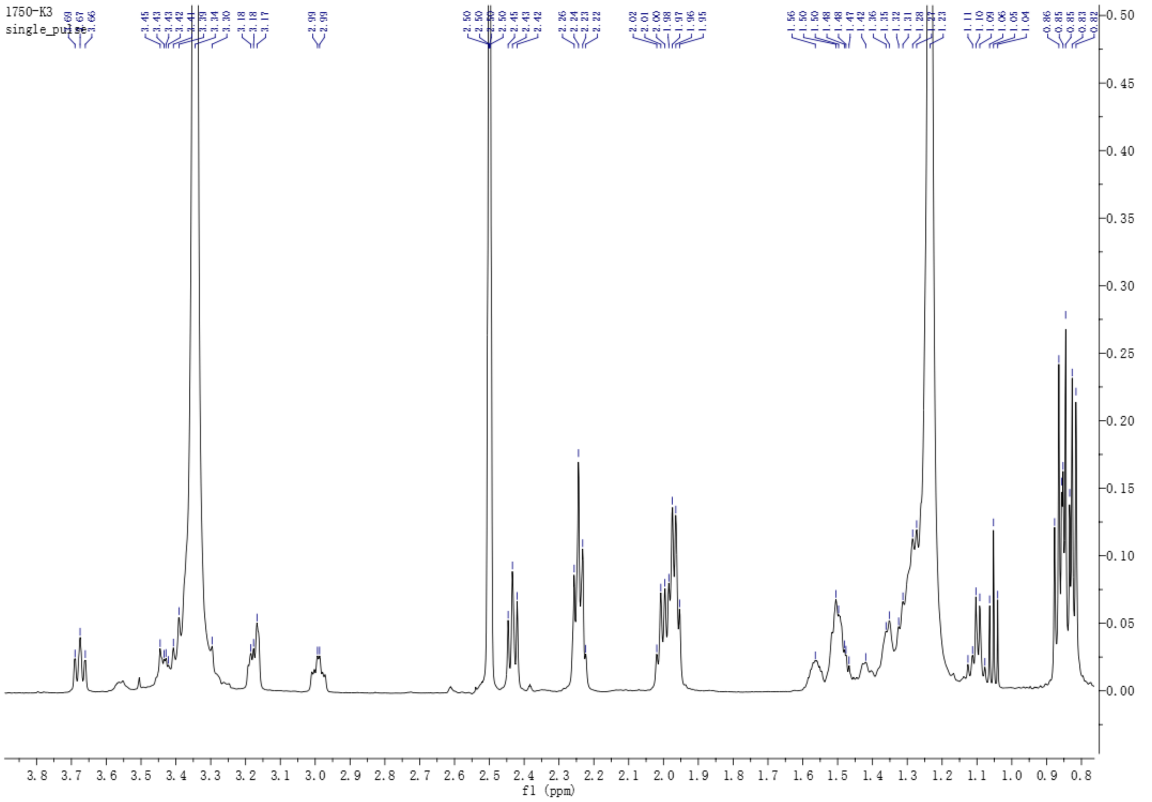

Figure S104. ^{13}C NMR spectrum of peniresorcinoside E (**5**, 150 MHz, in DMSO-d_6)

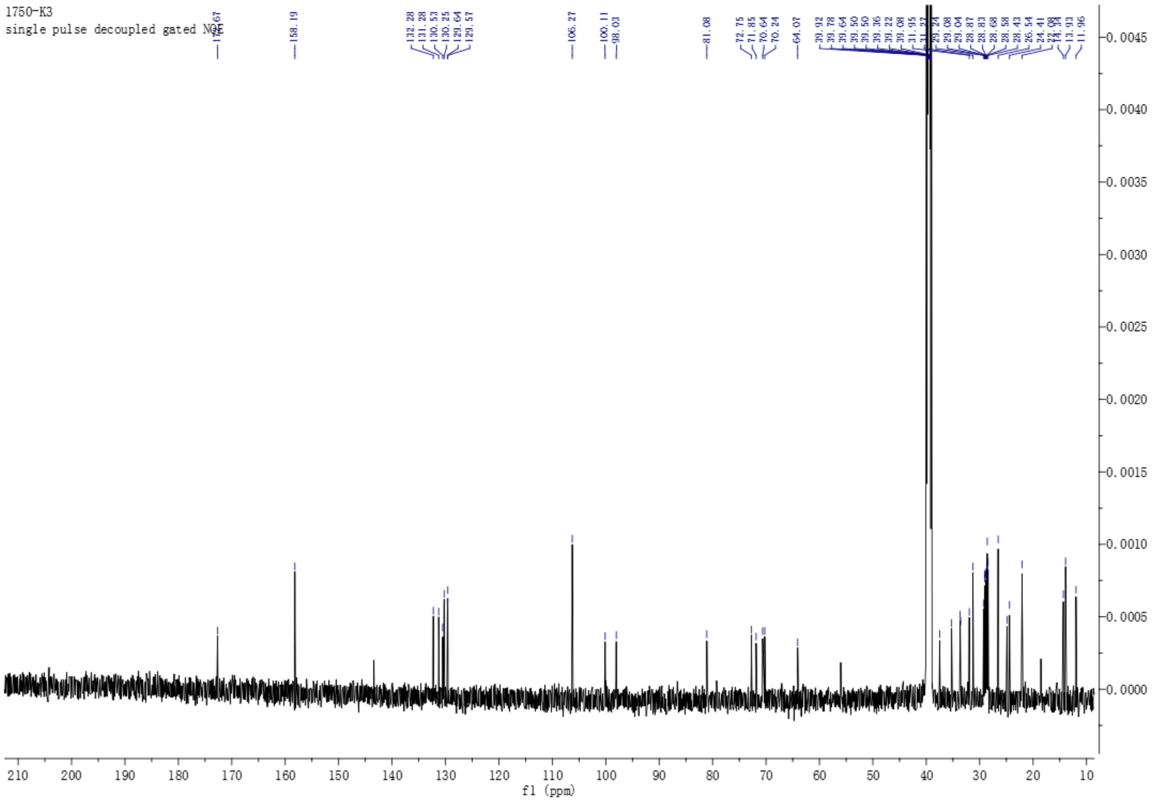

Figure S105. ^{13}C NMR spectrum of peniresorcinoside E (**5**, 150 MHz, in DMSO-d_6)

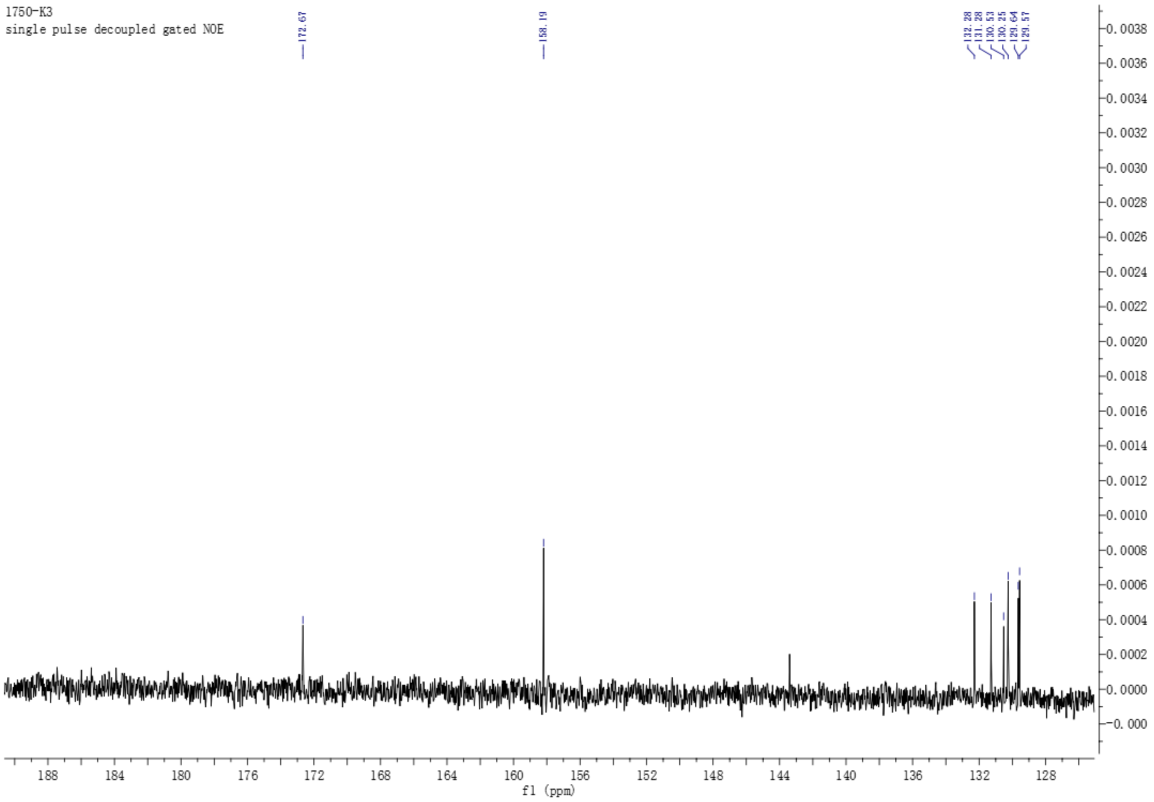

Figure S106. ^{13}C NMR spectrum of peniresorcinoside E (**5**, 600 MHz, in DMSO-d_6)

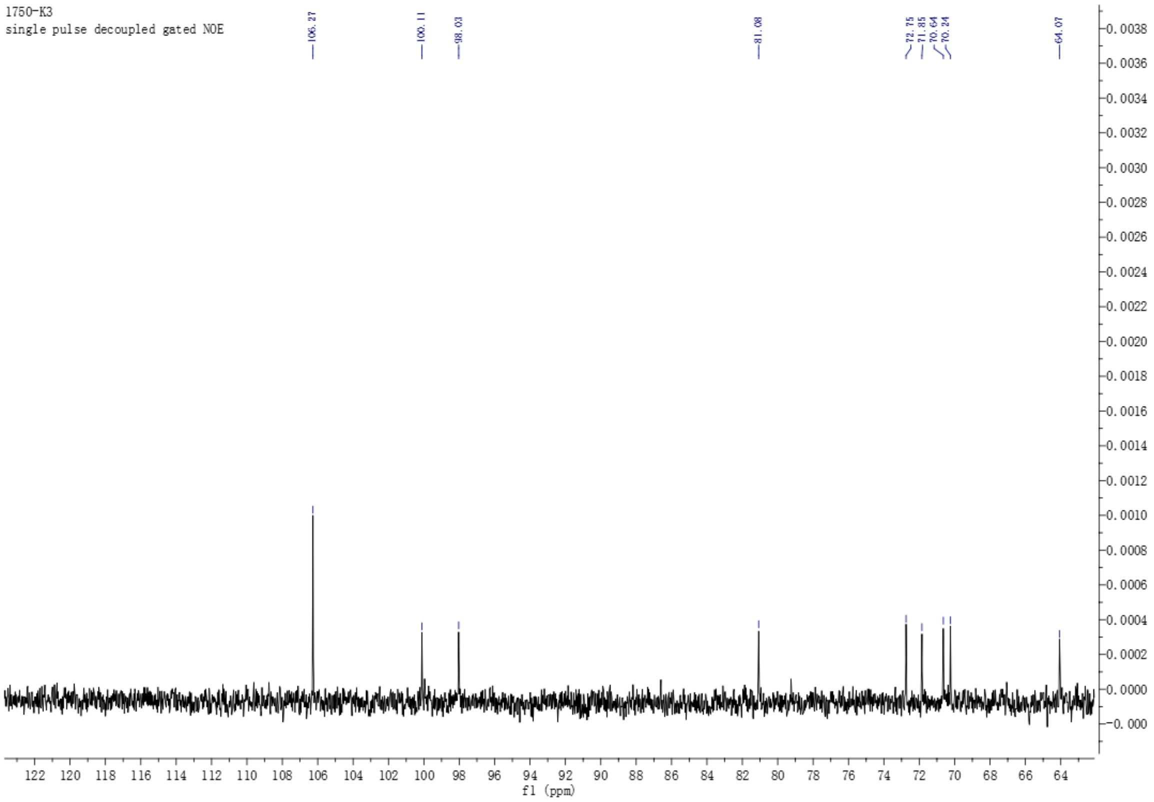

Figure S107. ^{13}C NMR spectrum of peniresorcinoside E (**5**, 600 MHz, in DMSO-d_6)

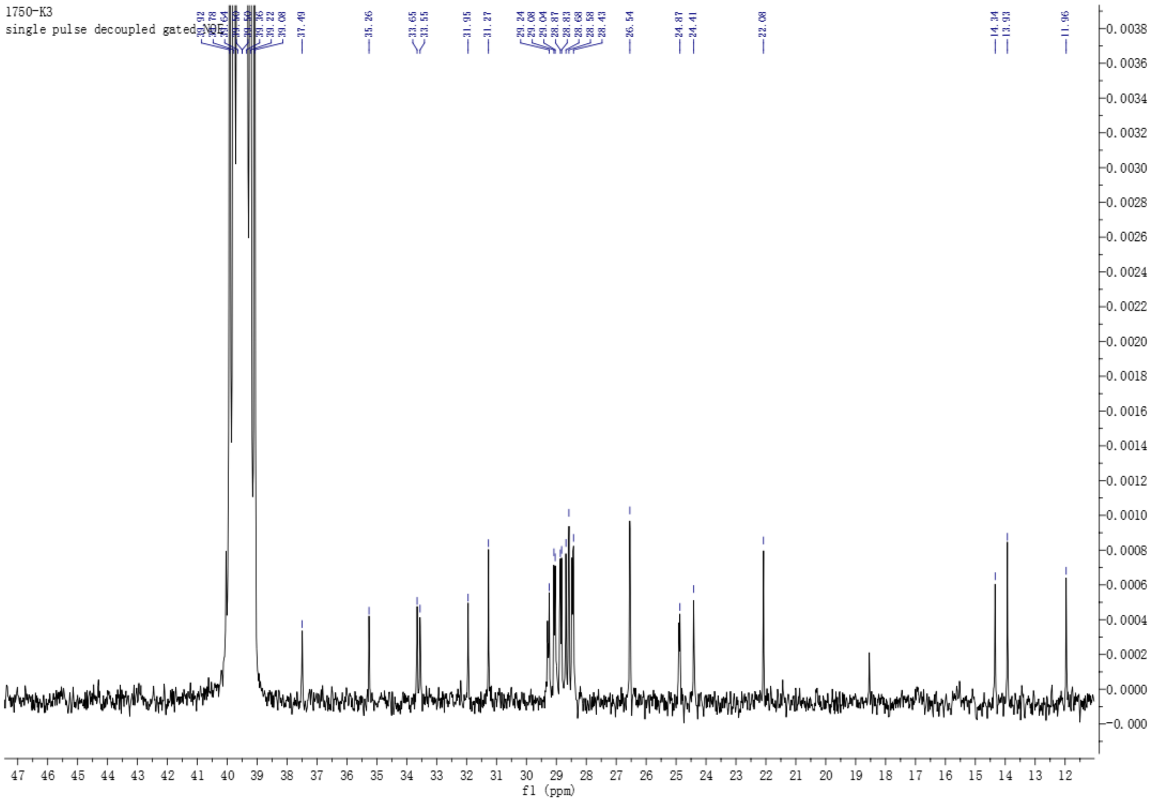

Figure S108. HMQC spectrum of peniresorcinoside E (**5**, in DMSO-d_6)

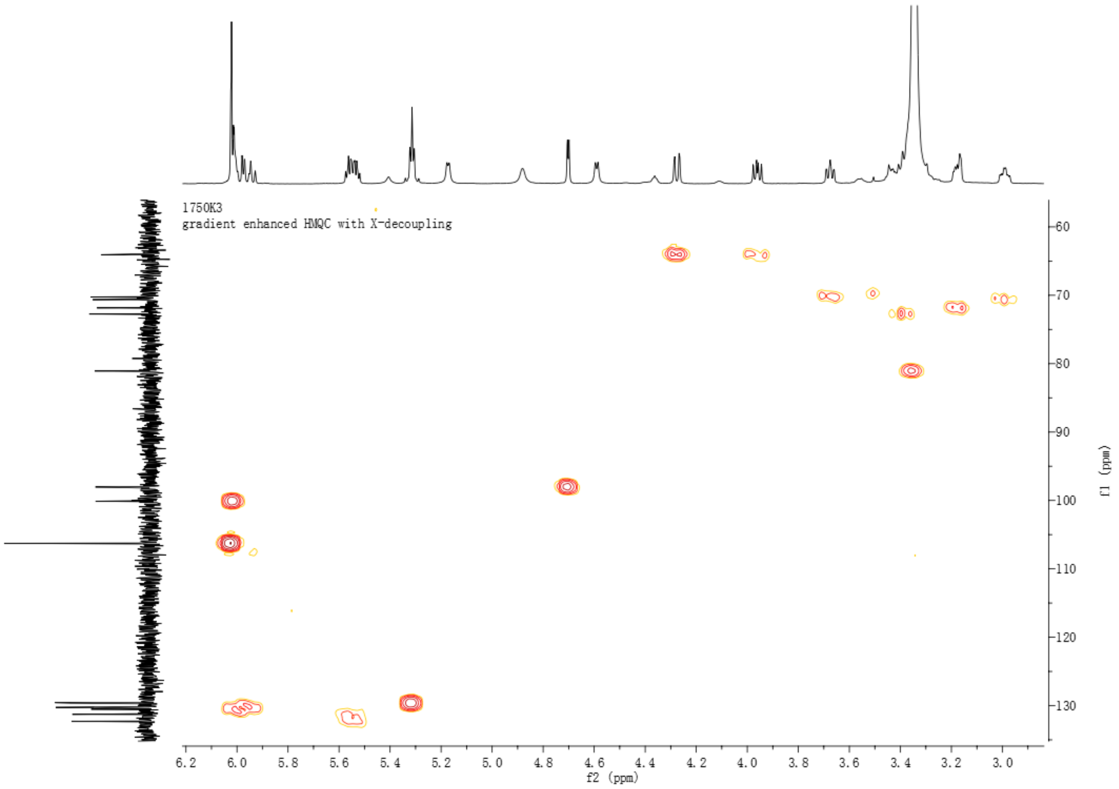

Figure S109. HMQC spectrum of peniresorcinoside E (**5**, in DMSO-d_6)

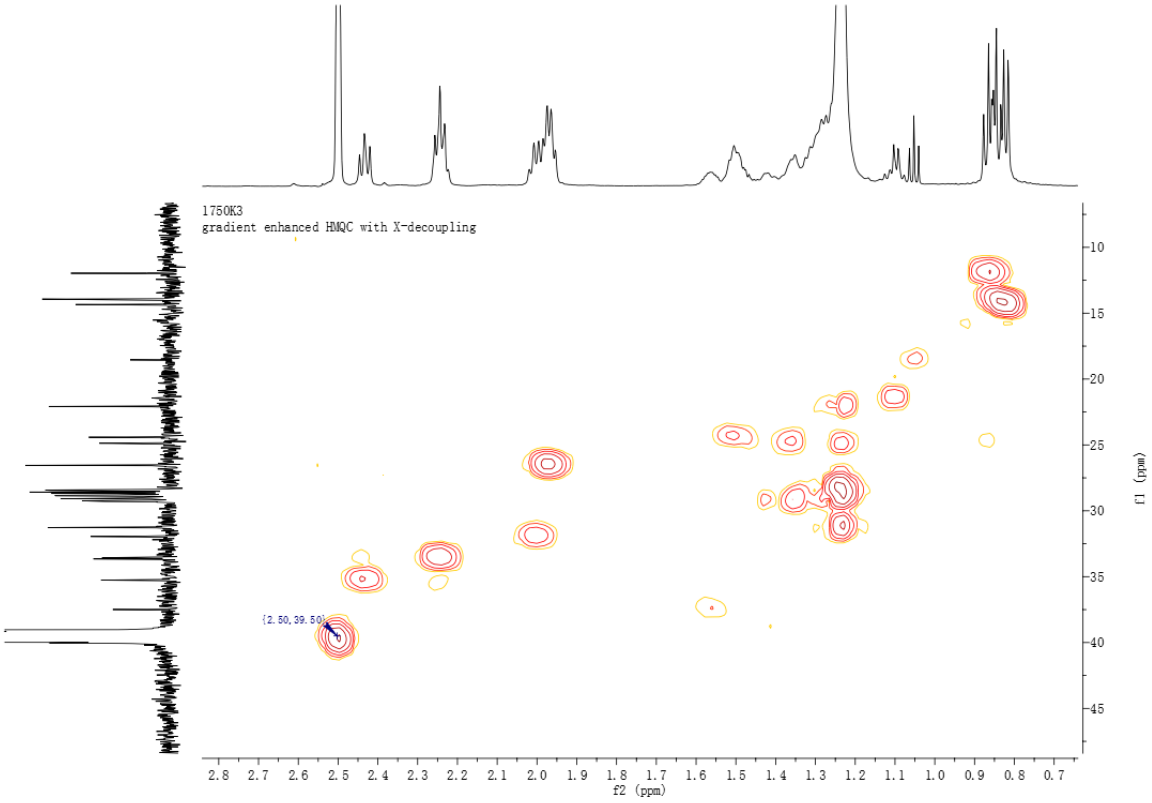

Figure S110. COSY spectrum of peniresorcinoside E (**5**, in DMSO-d_6)

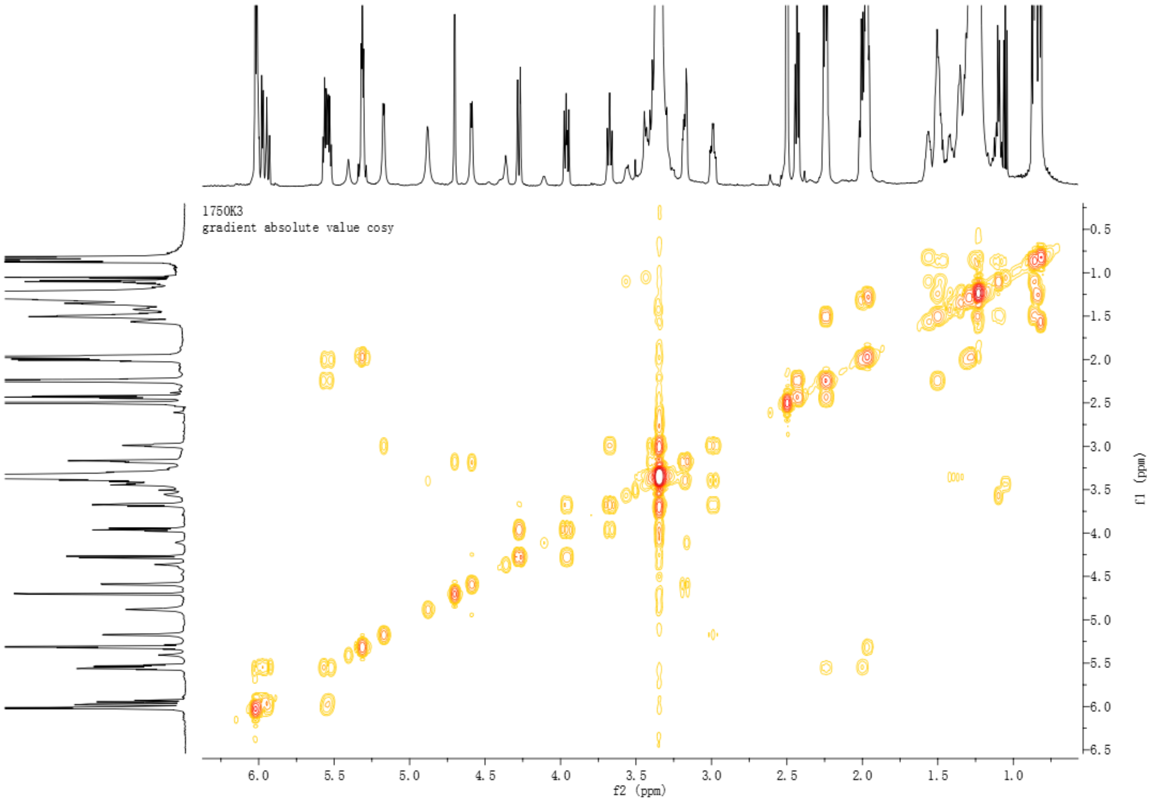

Figure S111. HMBC spectrum of peniresorcinoside E (**5**, in DMSO-d_6)

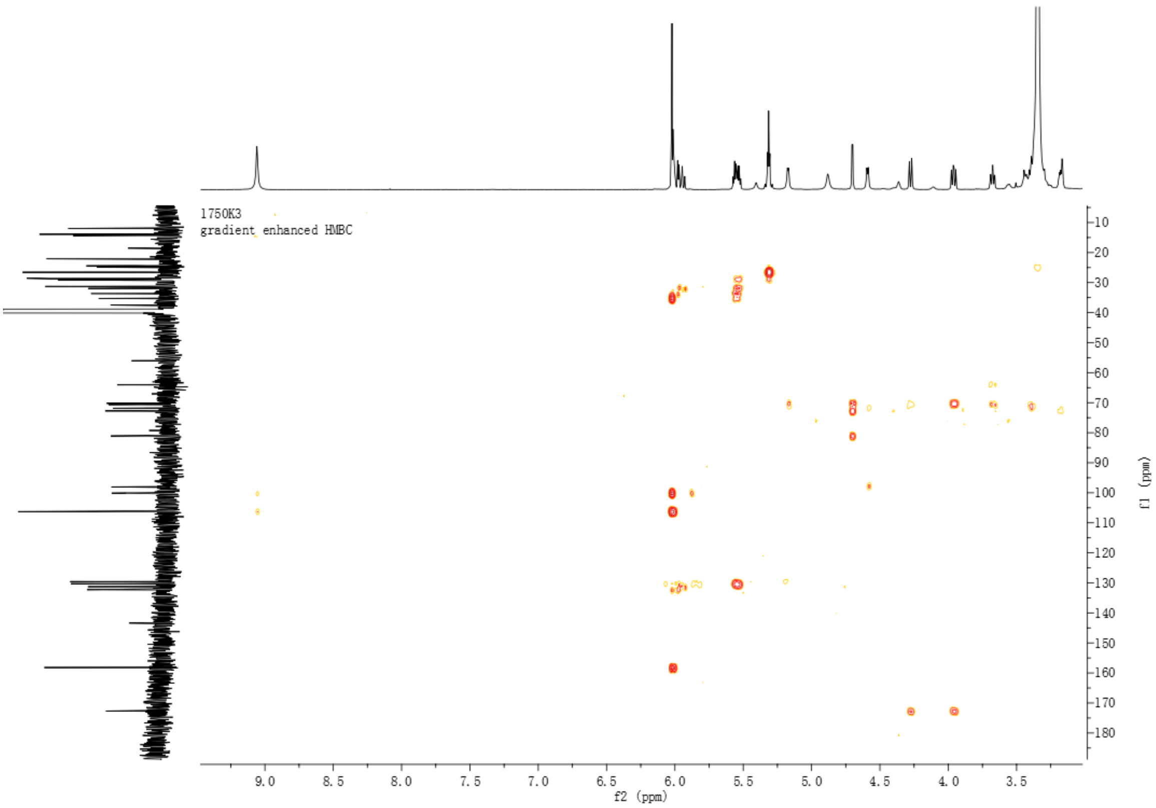

Figure S112. HMBC spectrum of peniresorcinoside E (**5**, in DMSO-d_6)

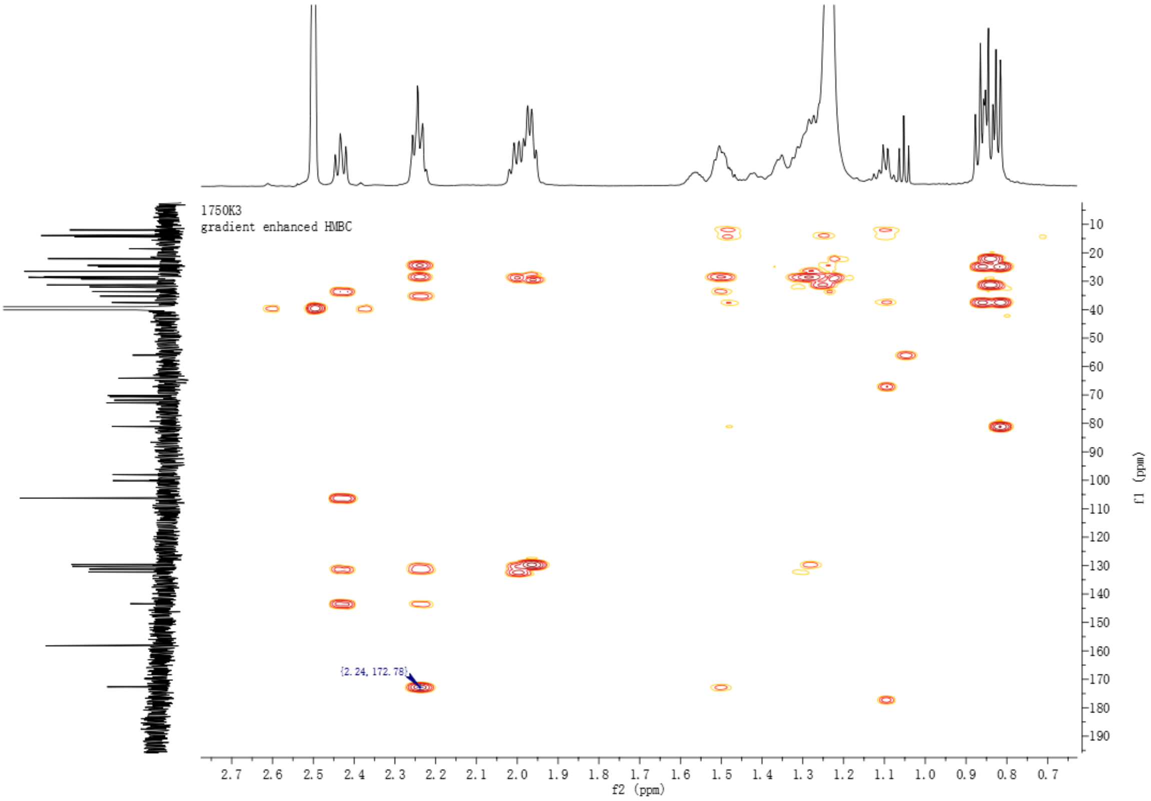

Figure S113. HRESIMS spectrum of peniresorcinoside E (**5**)

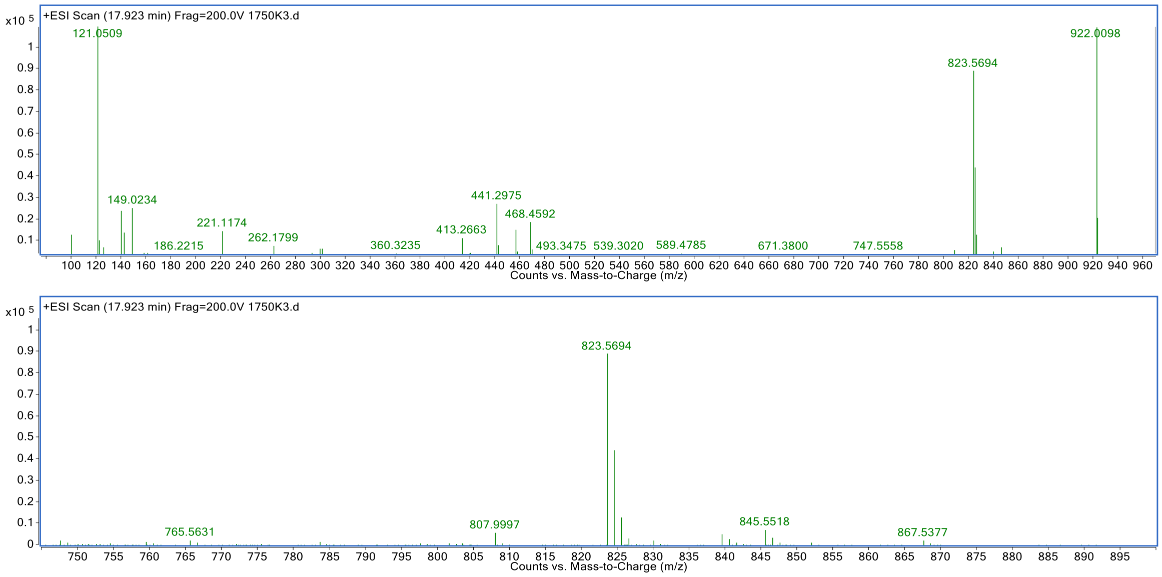

[M+Na]$^{+}$: 823.5694 (calcd for $C_{48}H_{80}NaO_9$, 823.5700).

Figure S114. UV (MeOH) spectrum of peniresorcinoside E (**5**)

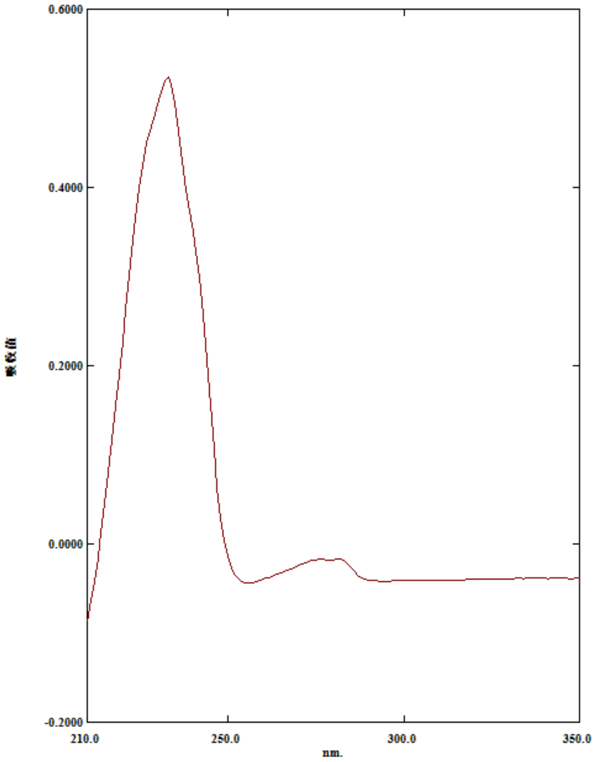

Figure S115. IR (ATR) spectrum of peniresorcinoside E (**5**)

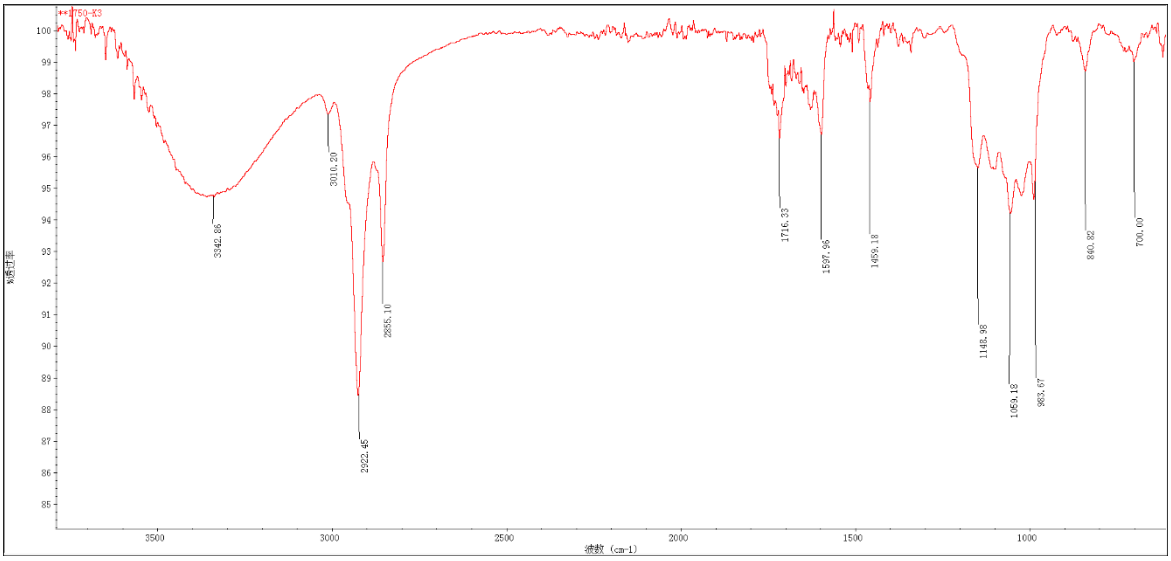

Figure S116. ^{1}H NMR spectrum of penidifarnesylin A (**6**, 600 MHz, in DMSO-d_6)

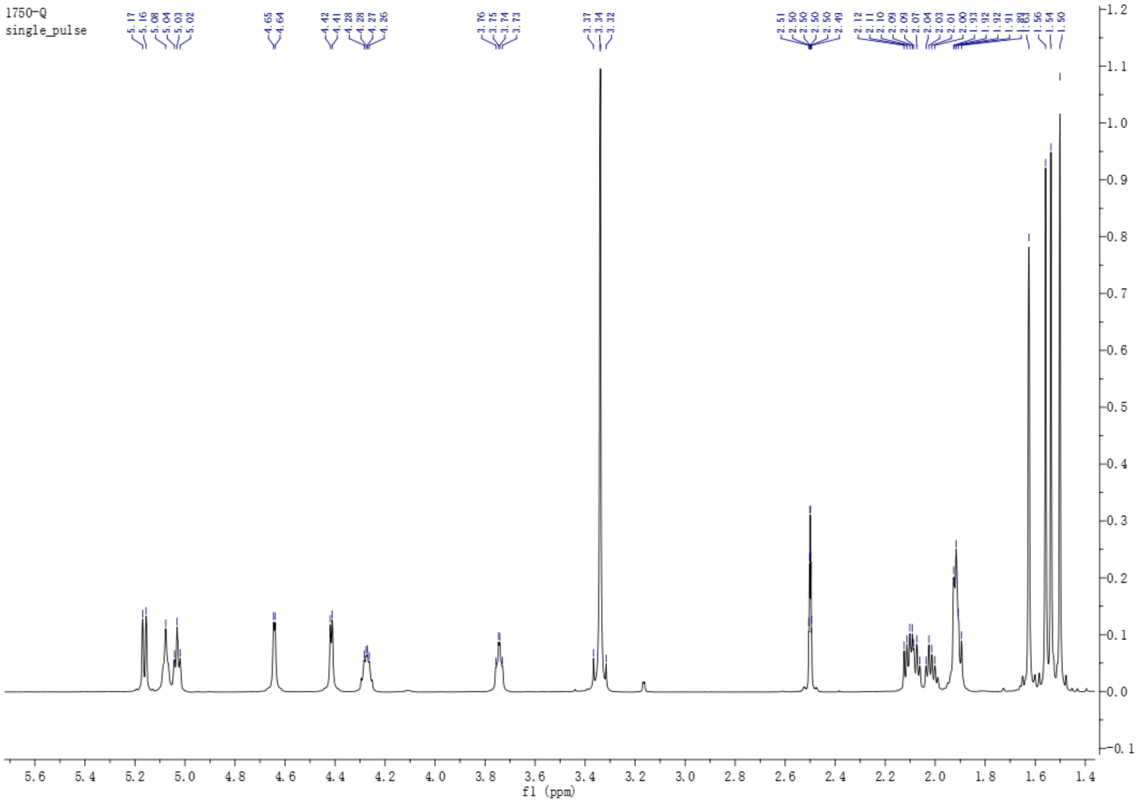

Figure S117. ^{1}H NMR spectrum of penidifarnesylin A (**6**, 600 MHz, in DMSO-d_6)

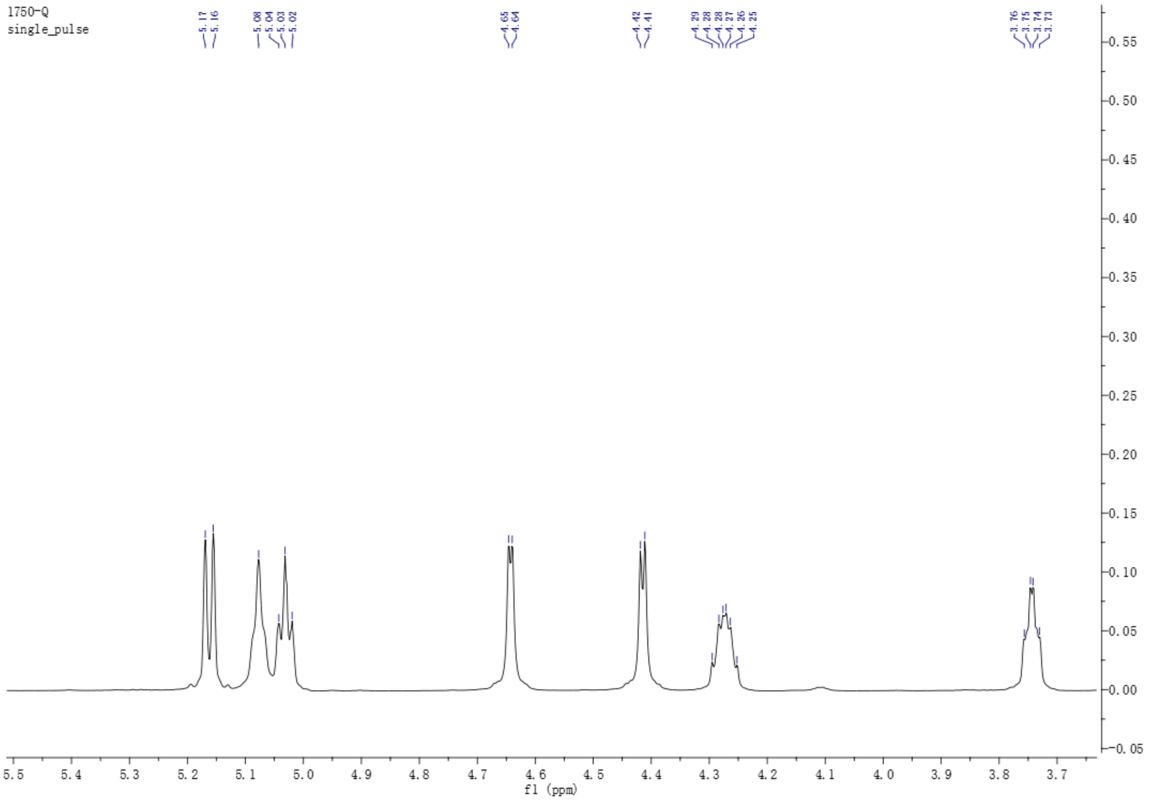

Figure S118. ^{1}H NMR spectrum of penidifarnesylin A (**6**, 600 MHz, in DMSO-d_6)

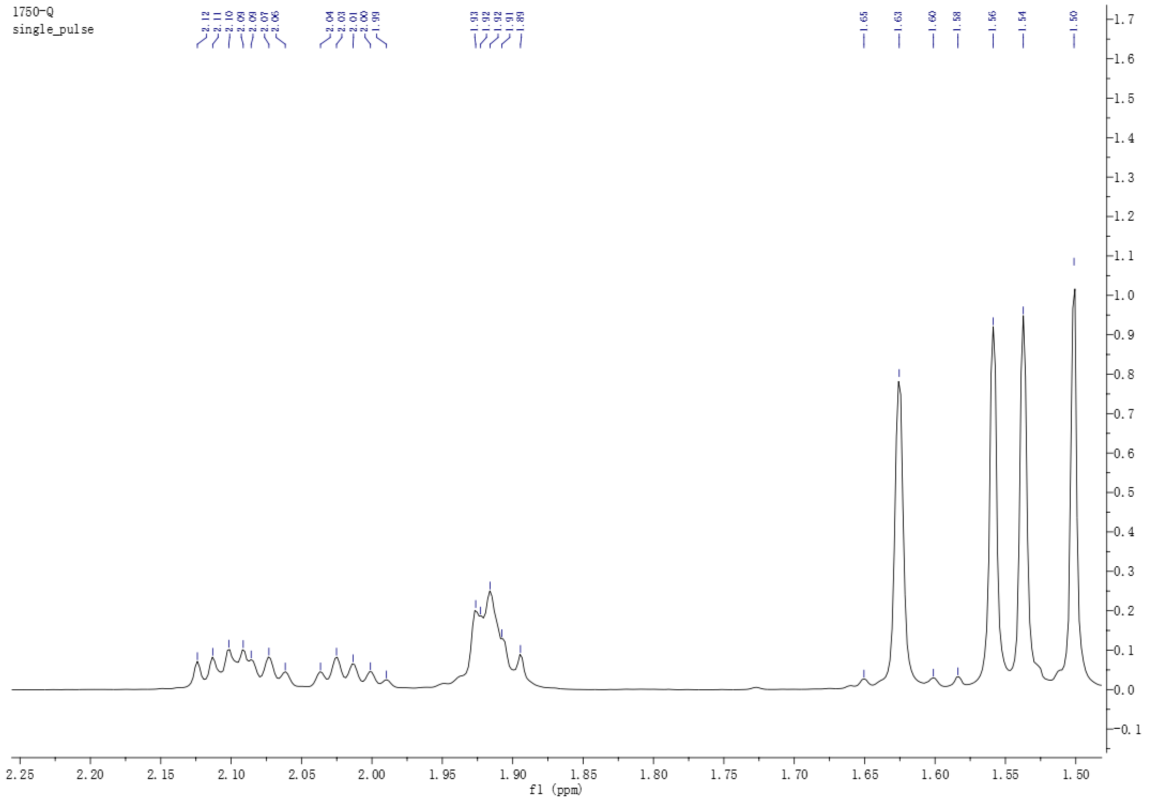

Figure S119. ^{13}C NMR spectrum of penidifarnesylin A (**6**, 150 MHz, in DMSO-*d*$_6$)

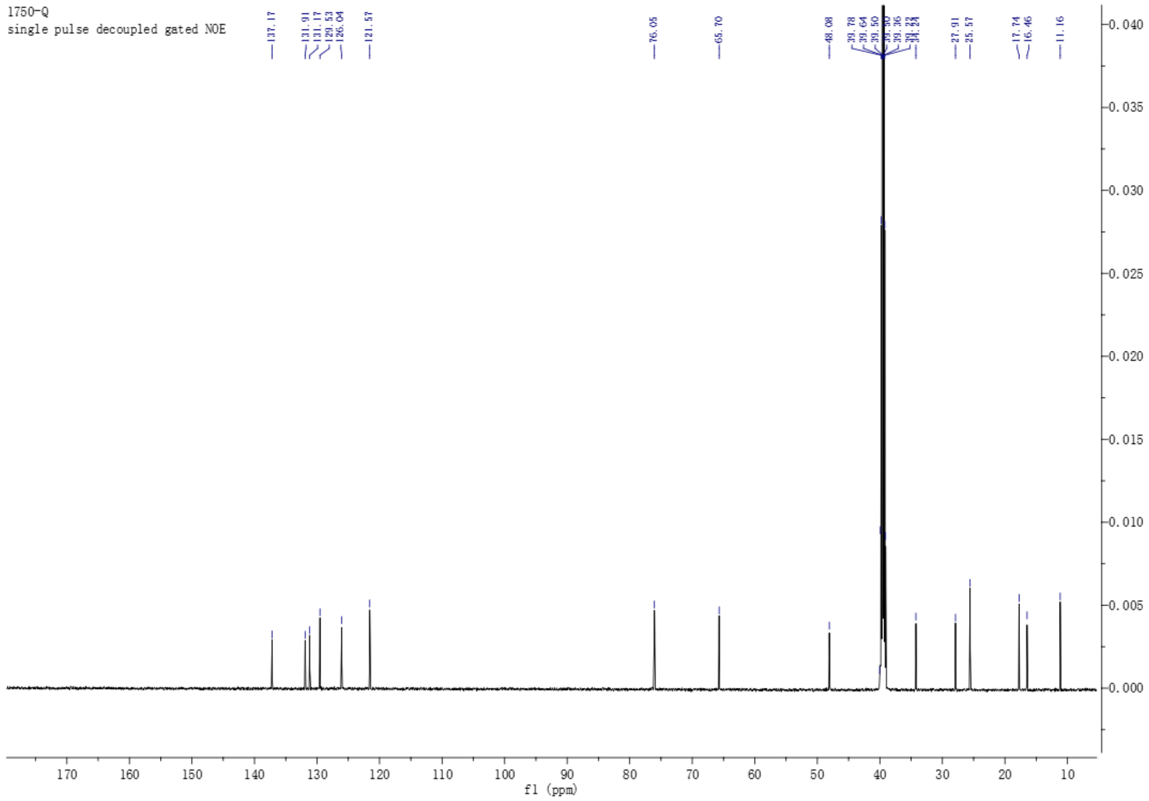

Figure S120. ^{13}C NMR spectrum of penidifarnesylin A (**6**, 500 MHz, in DMSO-*d*$_6$)

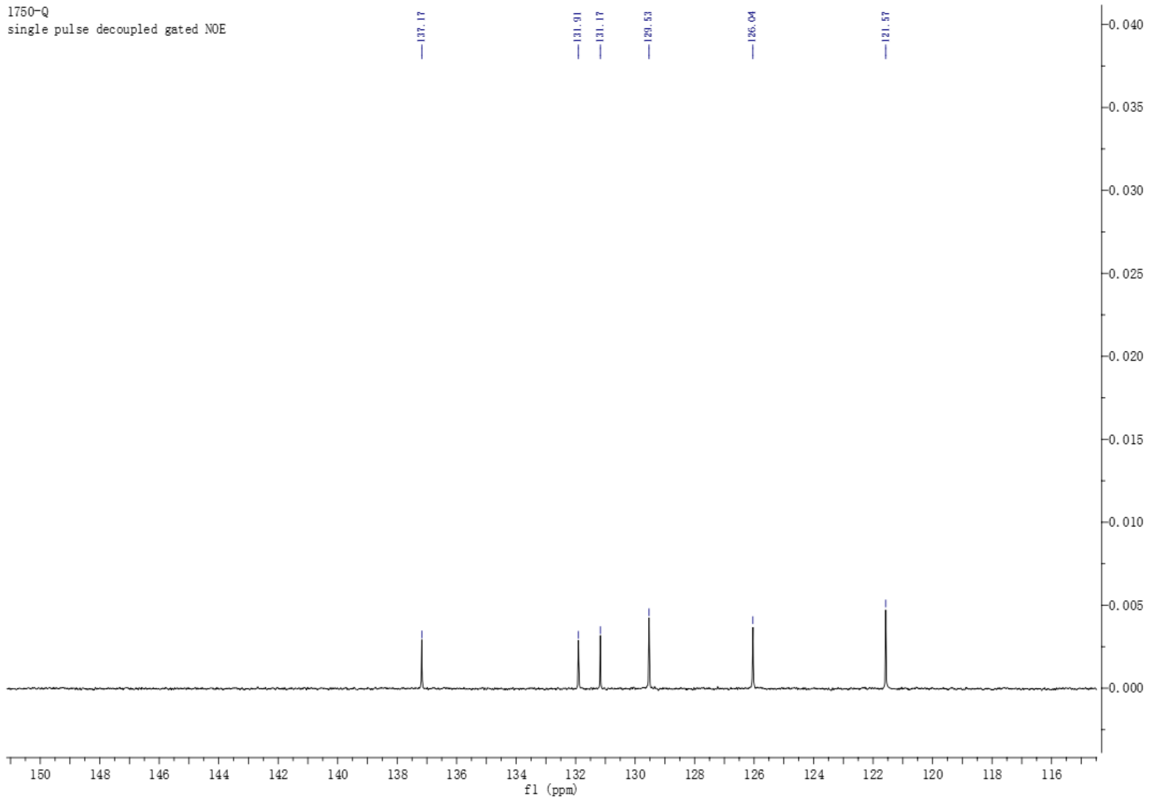

Figure S121. ^{13}C NMR spectrum of penidifarnesylin A (**6**, 150 MHz, in DMSO-d_6)

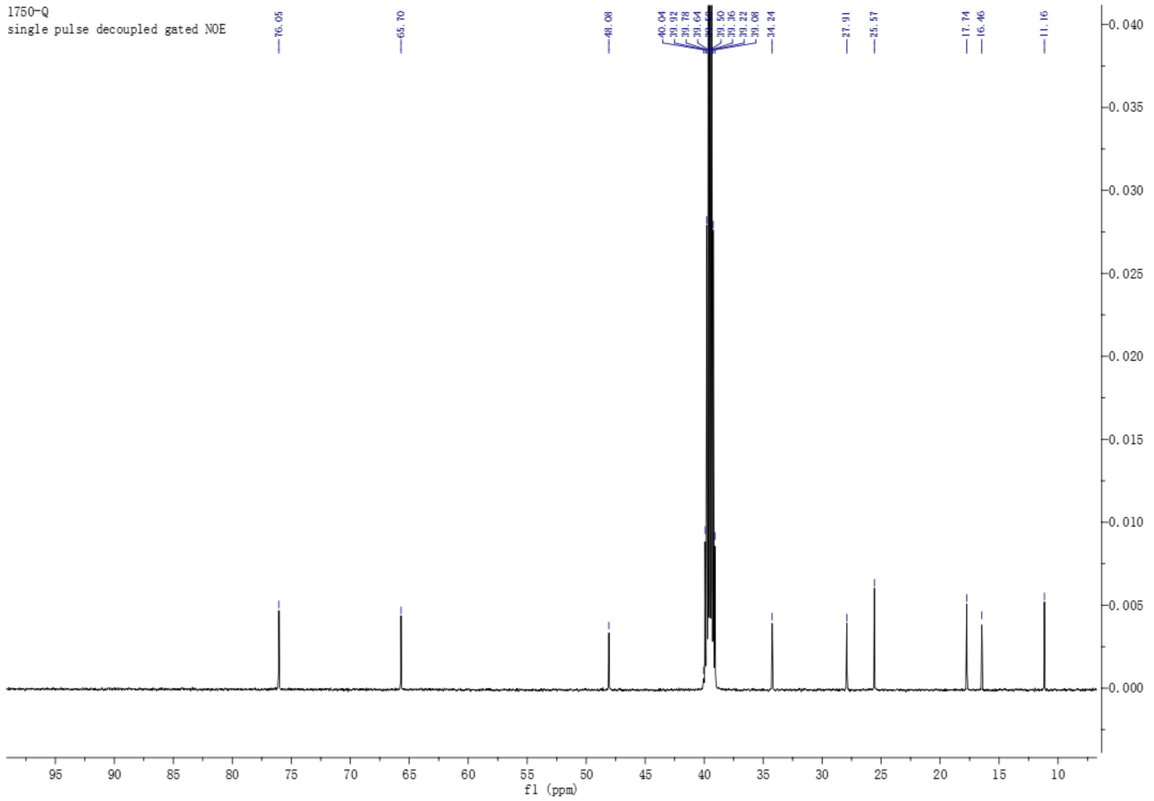

Figure S122. HMQC spectrum of penidifarnesylin A (**6**, in DMSO-d_6)

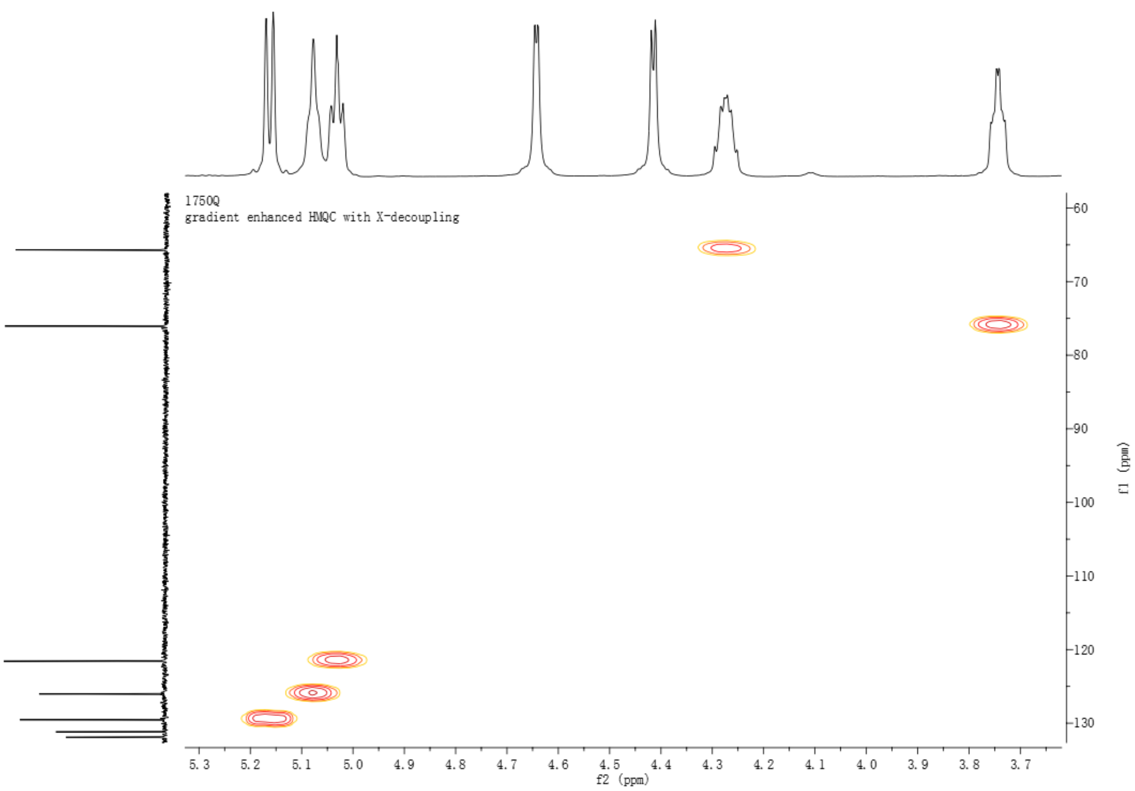

Figure S123. HMQC spectrum of penidifarnesylin A (**6**, in DMSO-d_6)

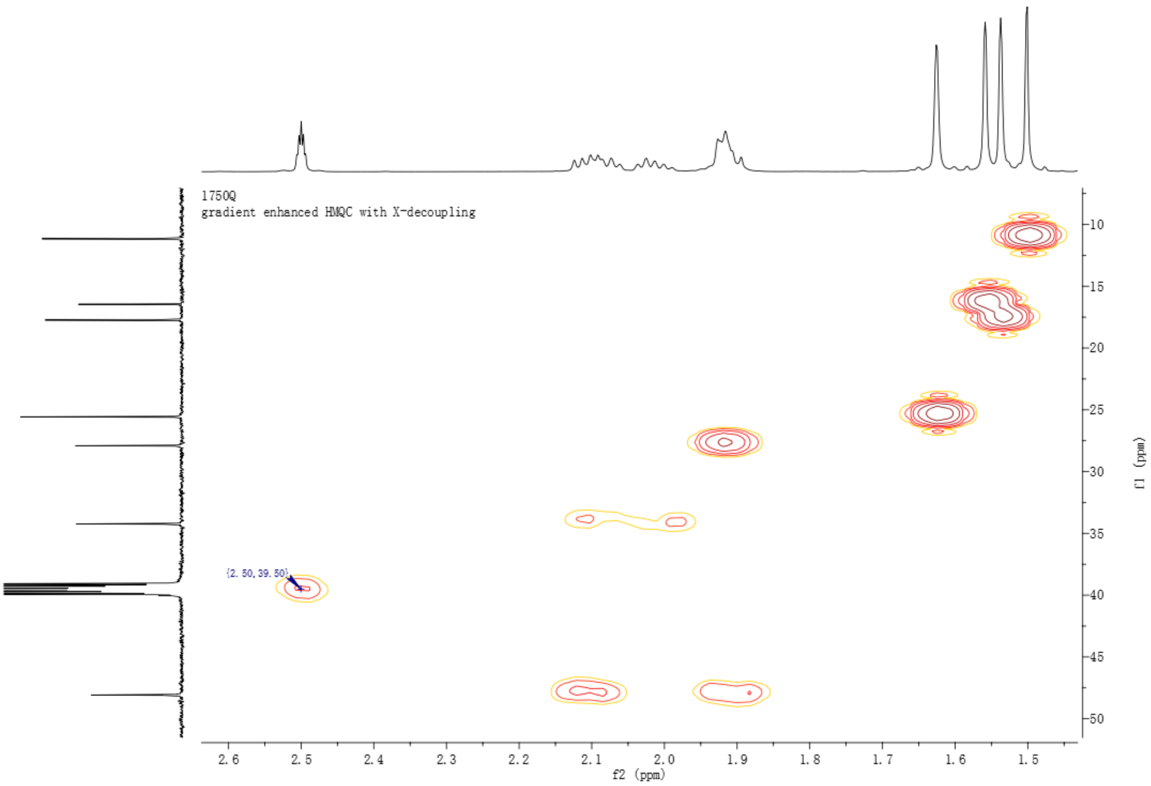

Figure S124. COSY spectrum of penidifarnesylin A (**6**, in DMSO-d_6)

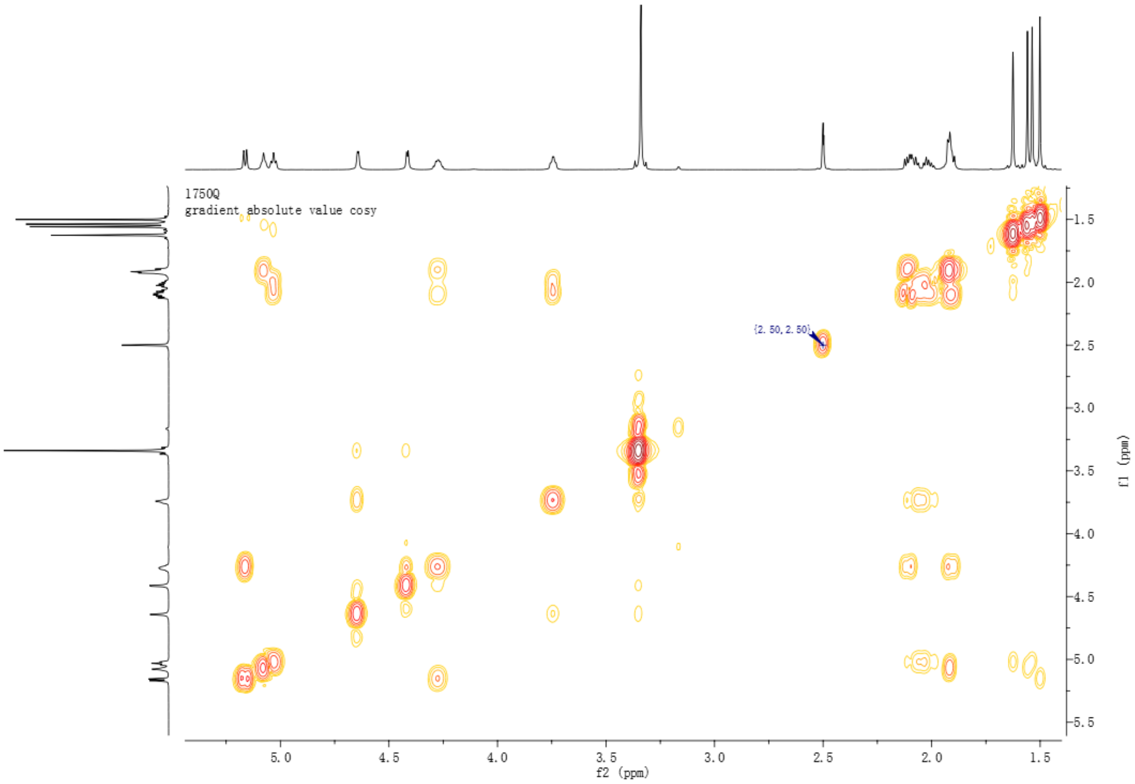

Figure S125. HMBC spectrum of penidifarnesylin A (**6**, in DMSO-d_6)

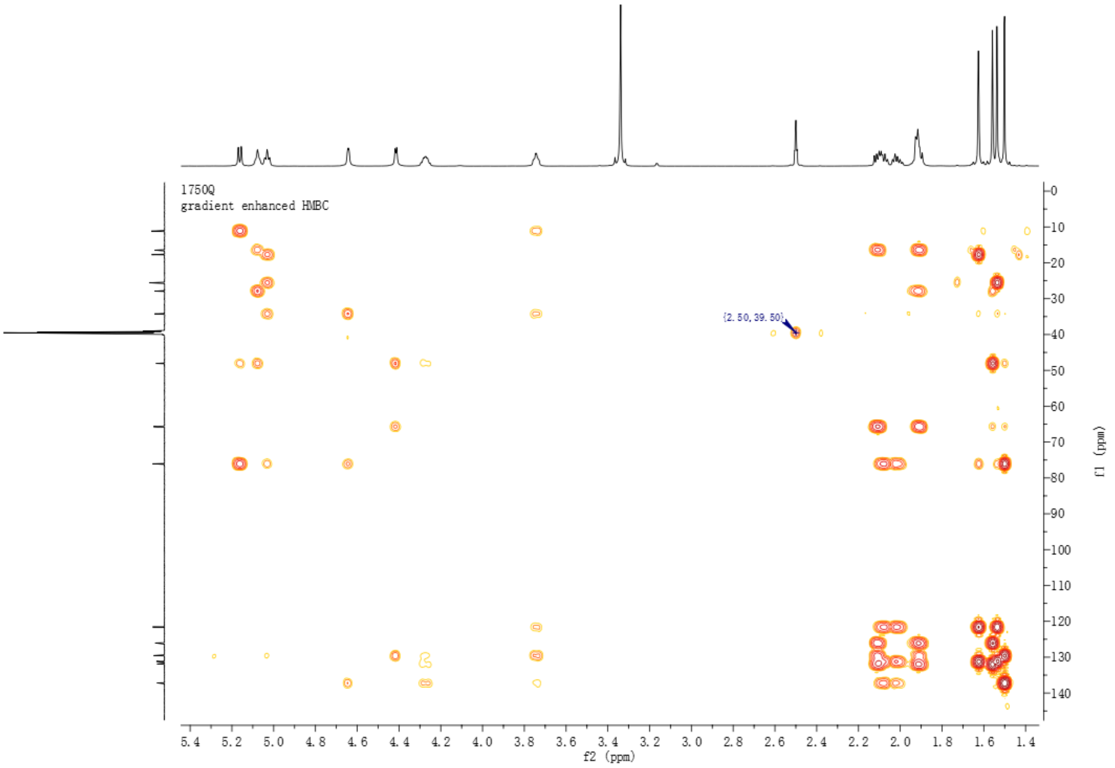

Figure S126. HMBC spectrum of penidifarnesylin A (**6**, in DMSO-d_6)

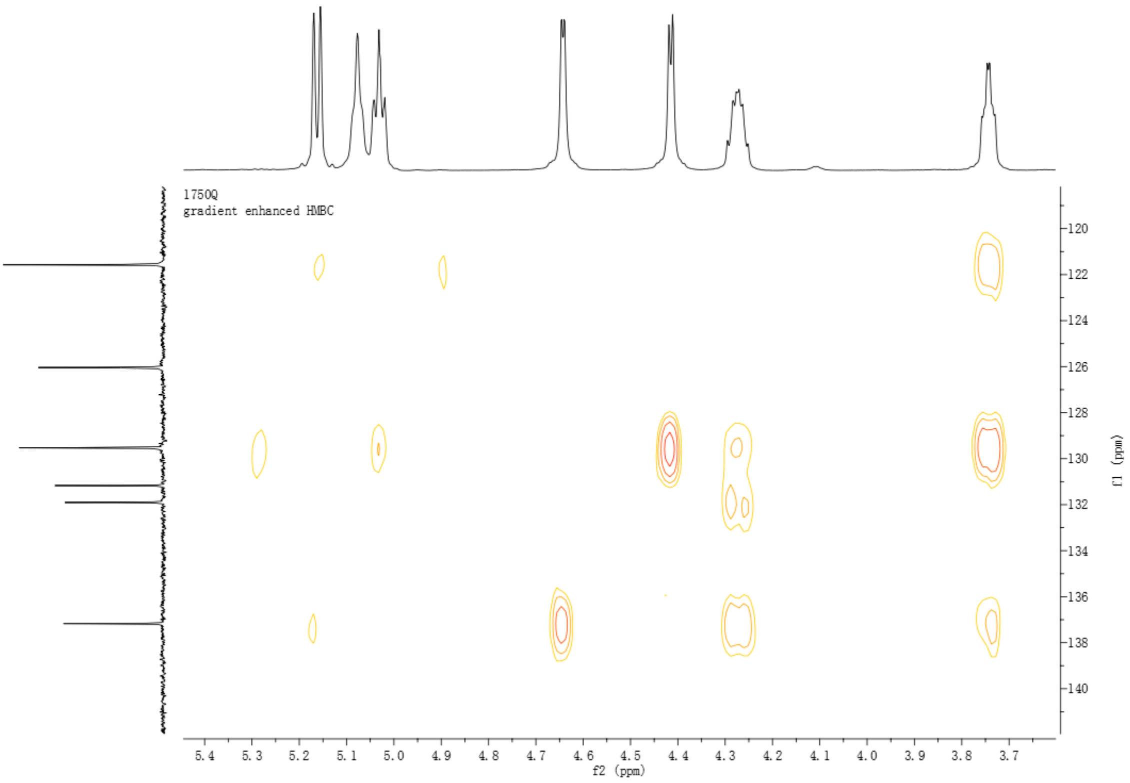

Figure S127. HMBC spectrum of penidifarnesylin A (**6**, in DMSO-d_6)

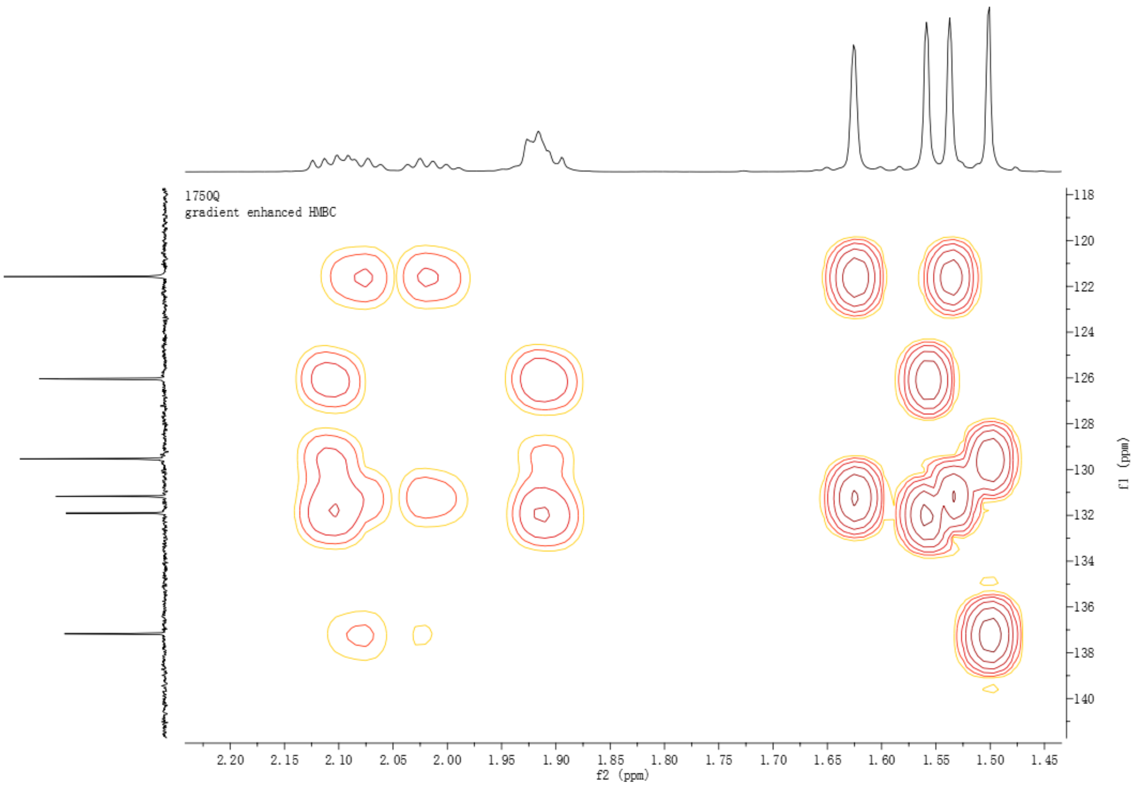

Figure S128. HMBC spectrum of penidifarnesylin A (**6**, in DMSO-d_6)

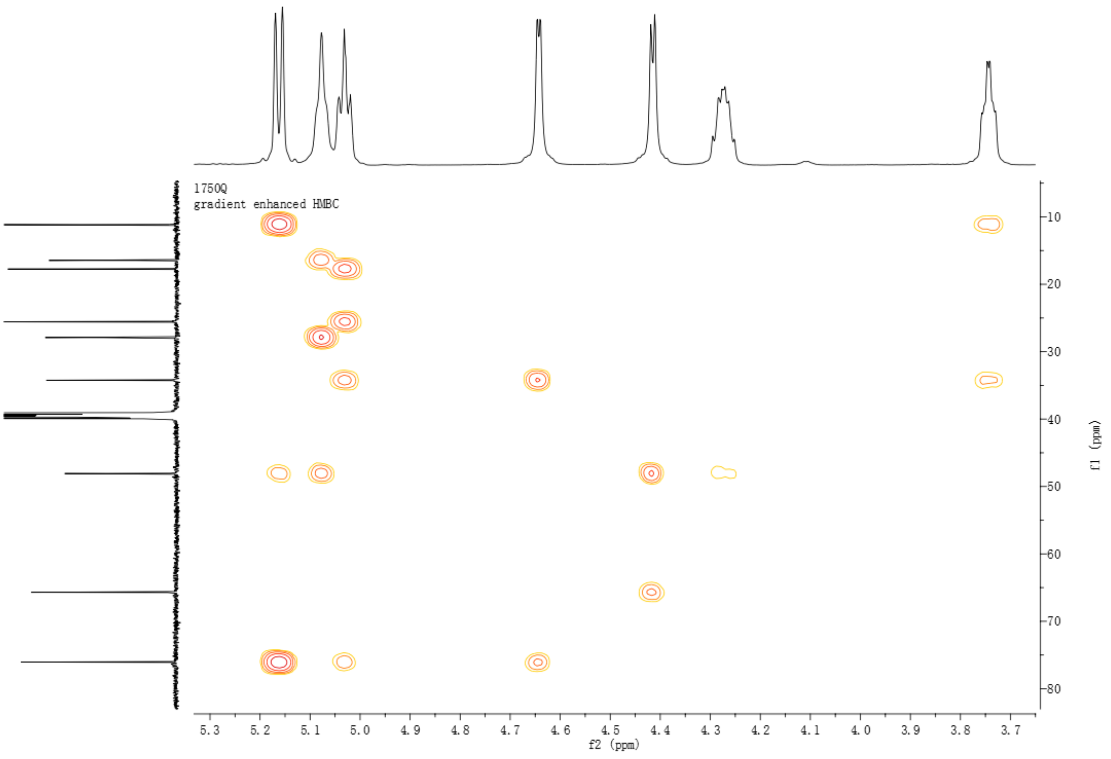

Figure S129. HMBC spectrum of penidifarnesylin A (**6**, in DMSO-d_6)

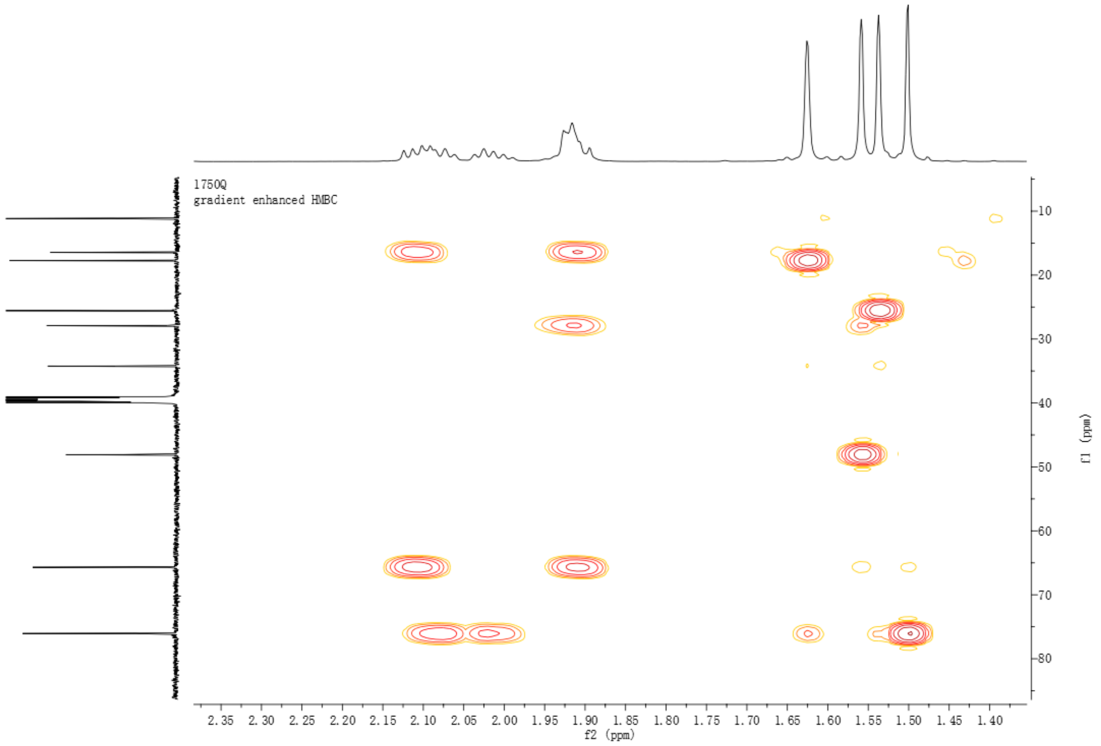

Figure S130. HRESIMS spectrum of penidifarnesylin A (**6**)

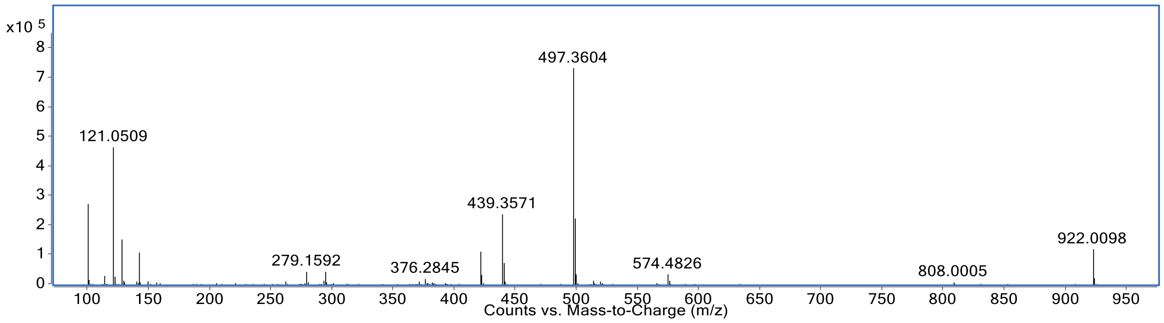

$[M+Na]^+$: 497.3604 (calcd for $C_{30}H_{50}NaO_4$, 497.3607).

Figure S131. UV (MeOH) spectrum of penidifarnesylin A (**6**)

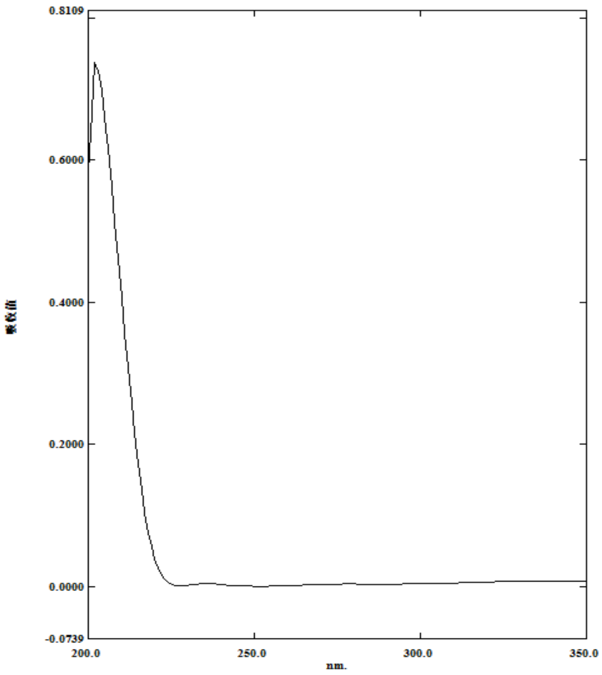

Figure S132. IR (ATR) spectrum of penidifarnesylin A (**6**)

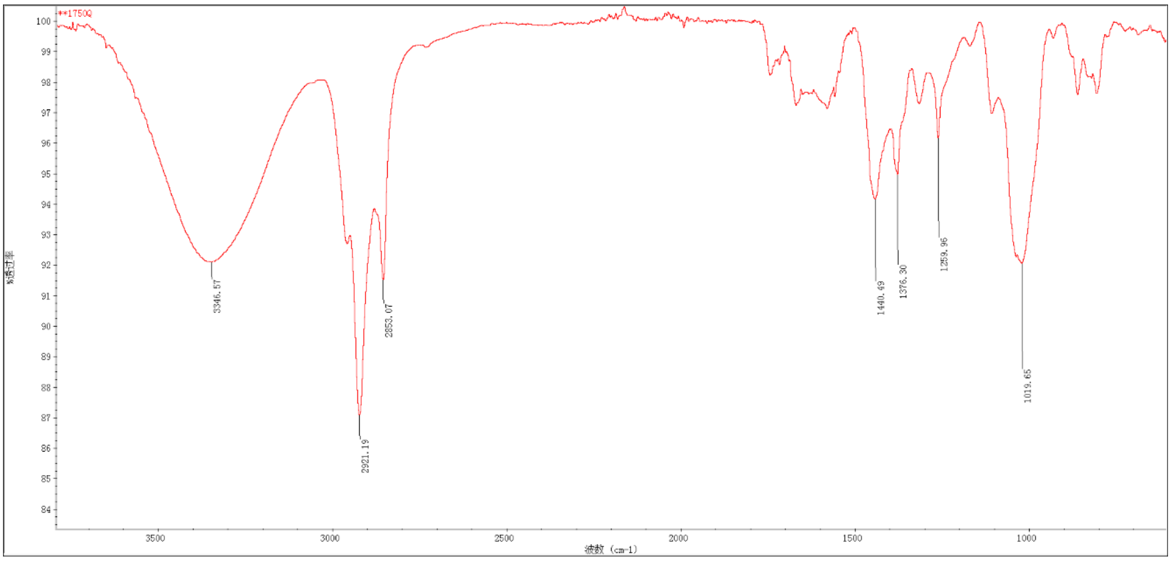

Figure S133. ^{1}H NMR spectrum of penipyridinone A (**7**, 600 MHz, in DMSO-d_6)

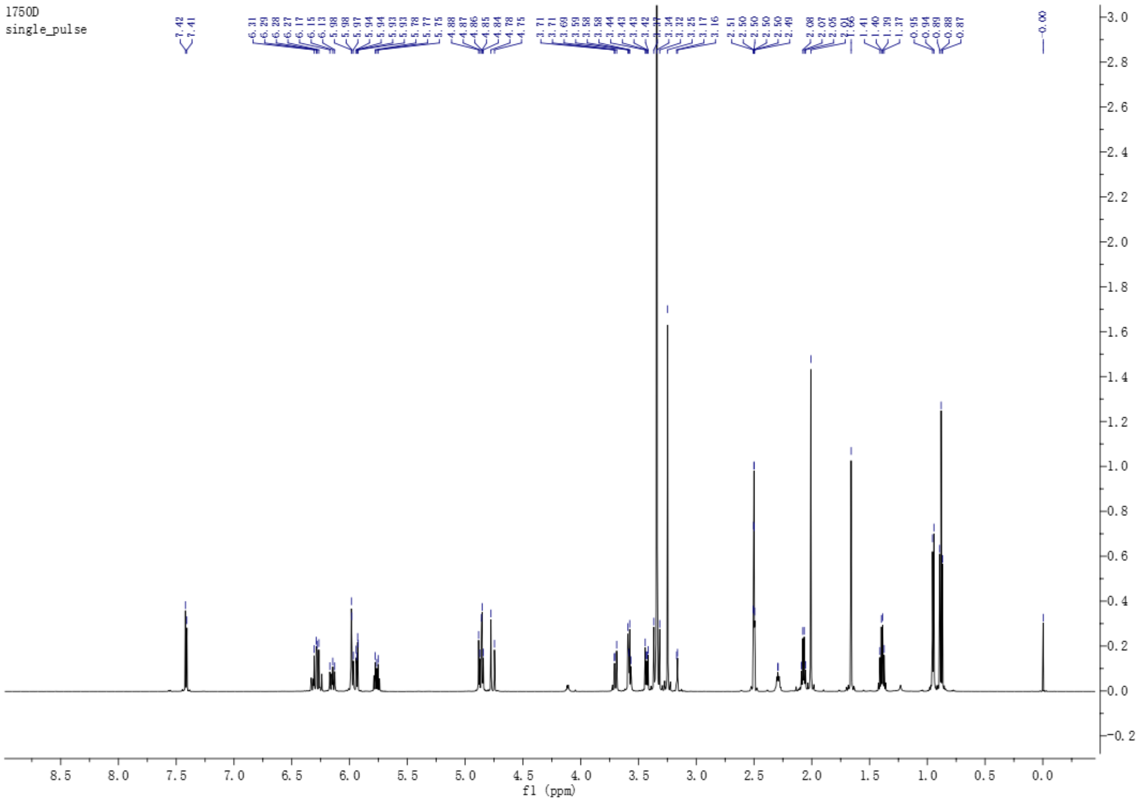

Figure S134. ^{1}H NMR spectrum of penipyridinone A (**7**, 600 MHz, in DMSO-d_6)

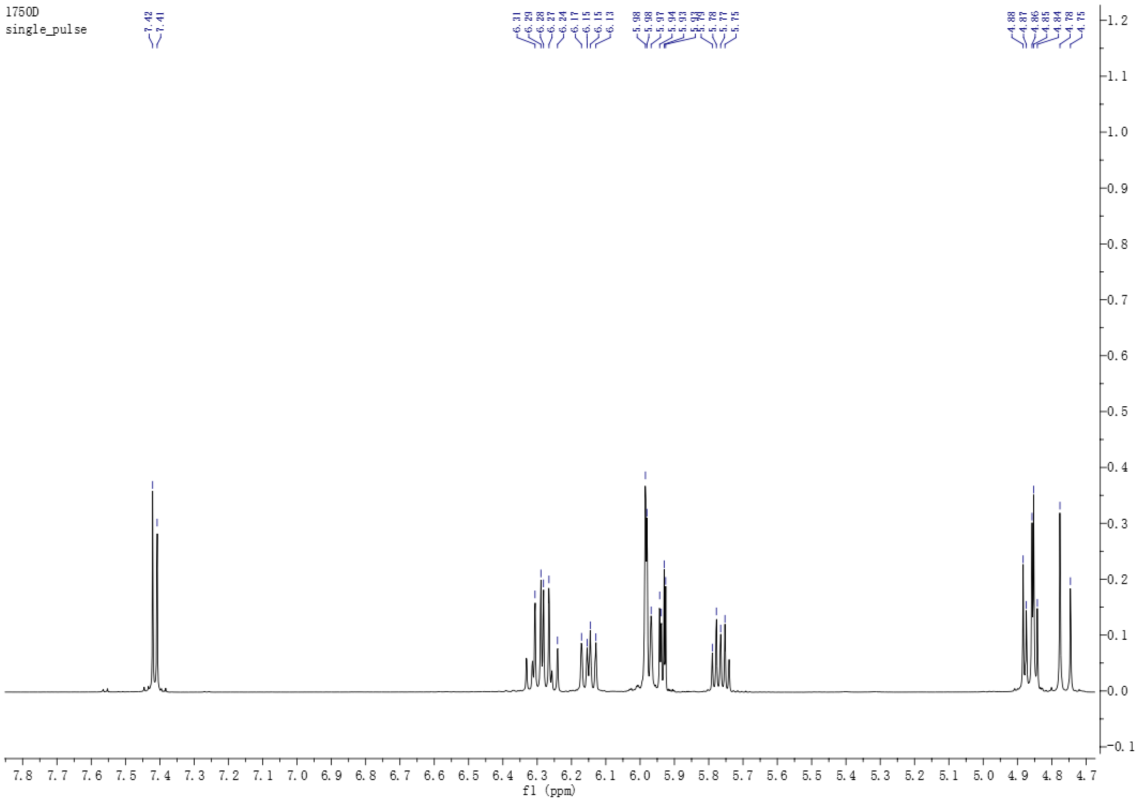

Figure S135. ^{1}H NMR spectrum of penipyridinone A (**7**, 600 MHz, in DMSO-d_6)

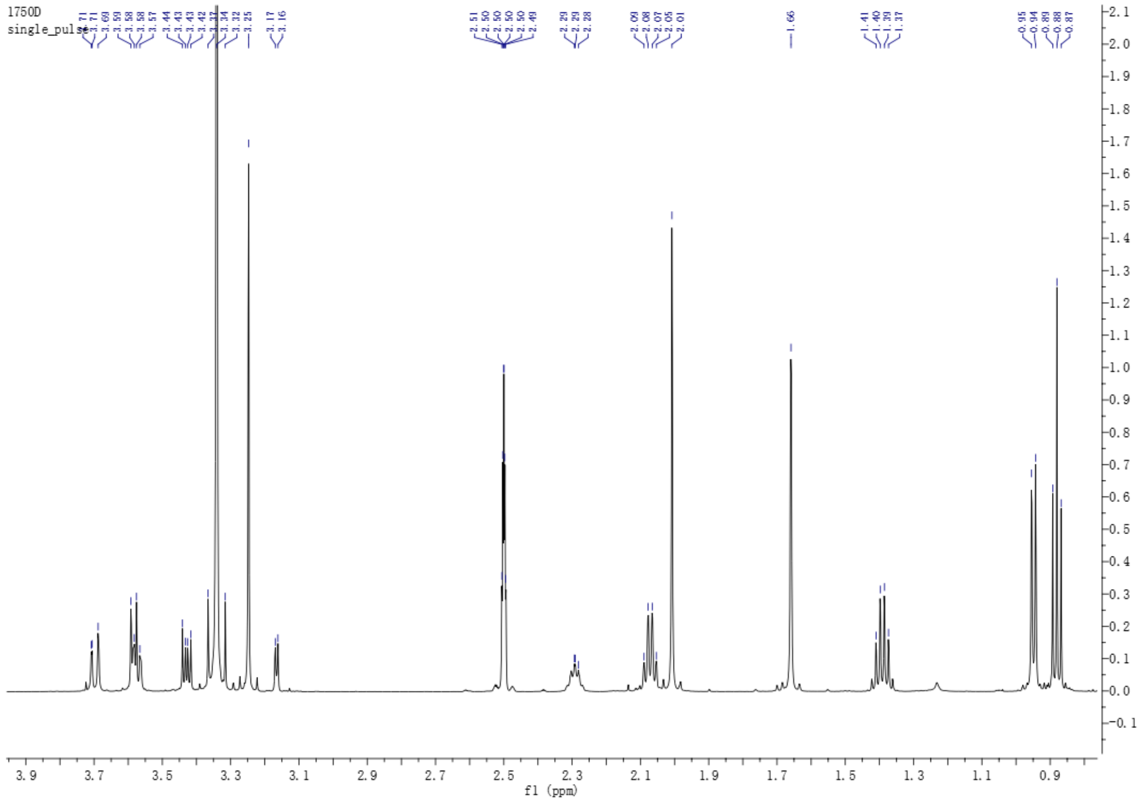

Figure S136. ^{13}C NMR spectrum of penipyridinone A (**7**, 150 MHz, in DMSO-d_6)

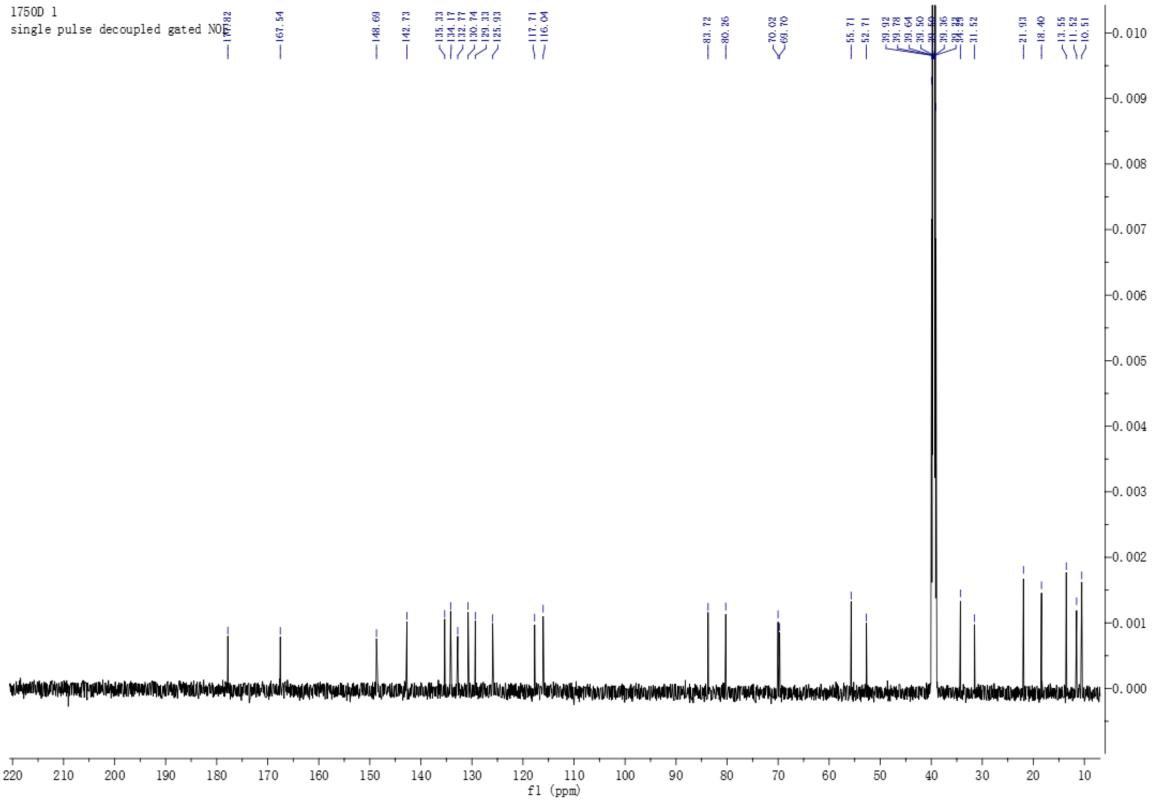

Figure S137. ^{13}C NMR spectrum of penipyridinone A (**7**, 150 MHz, in DMSO-d_6)

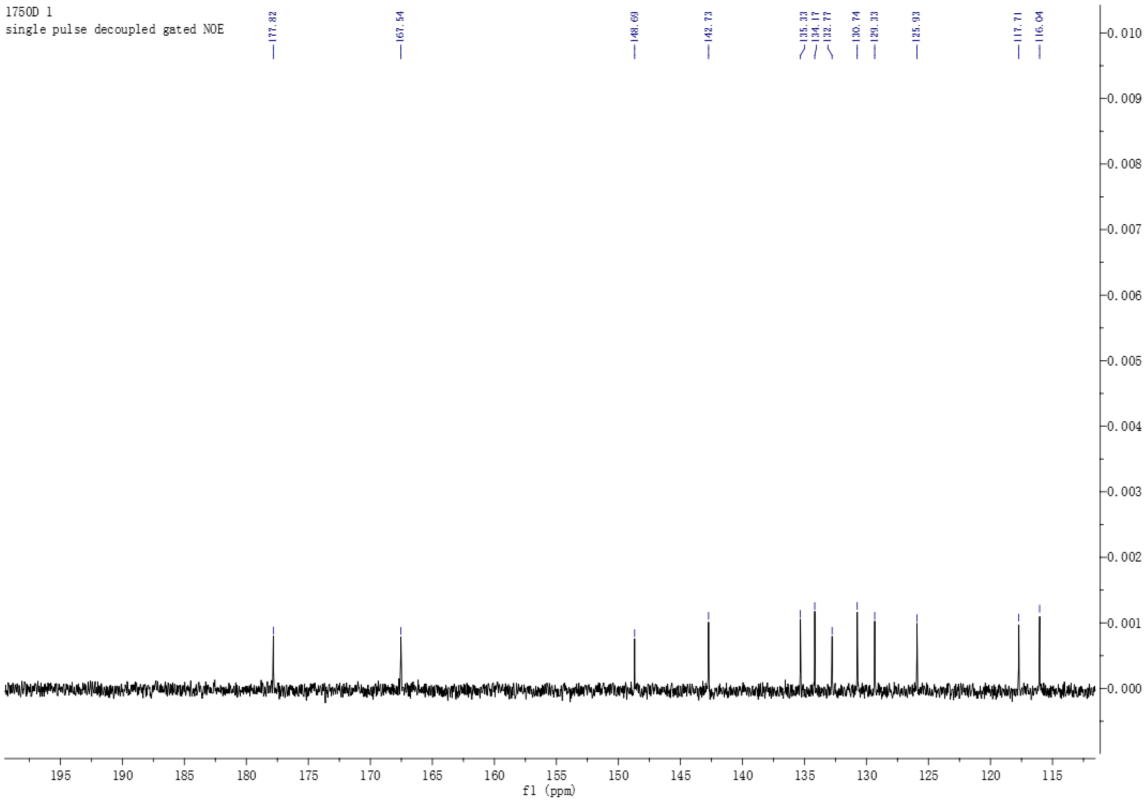

Figure S138. ^{13}C NMR spectrum of penipyridinone A (**7**, 150 MHz, in DMSO-d_6)

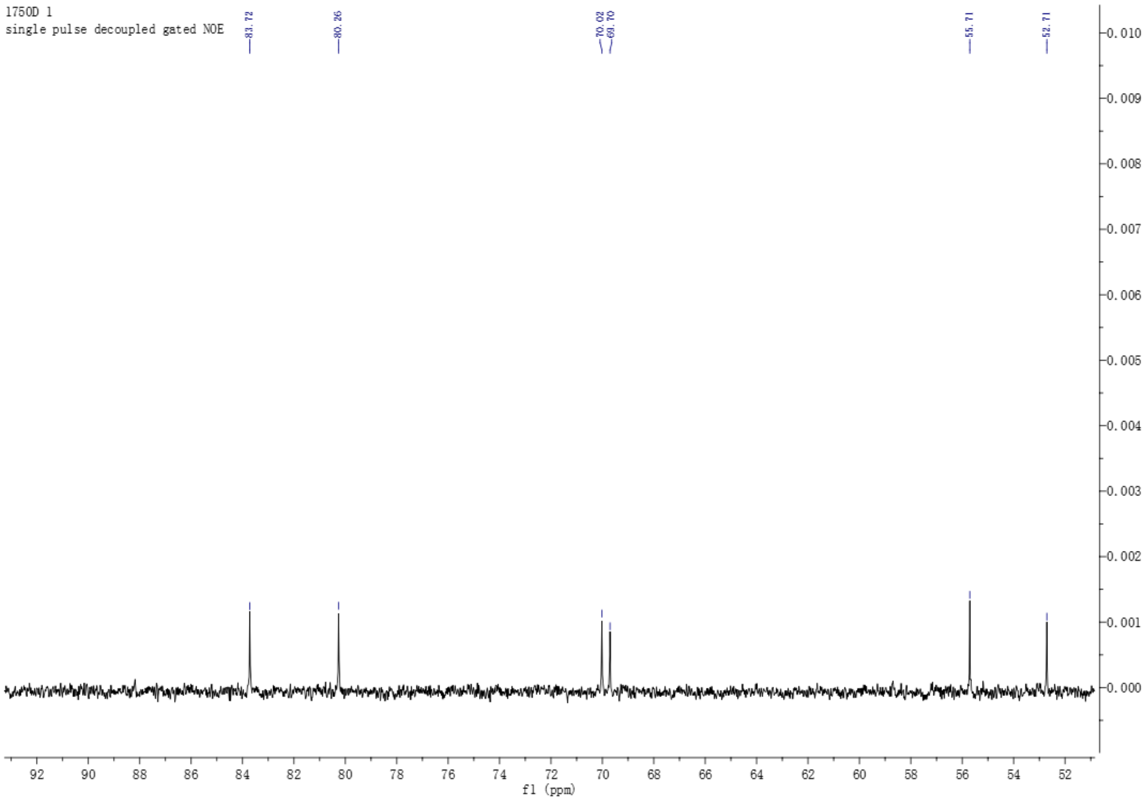

Figure S139. ^{13}C NMR spectrum of penipyridinone A (**7**, 150 MHz, in DMSO-d_6)

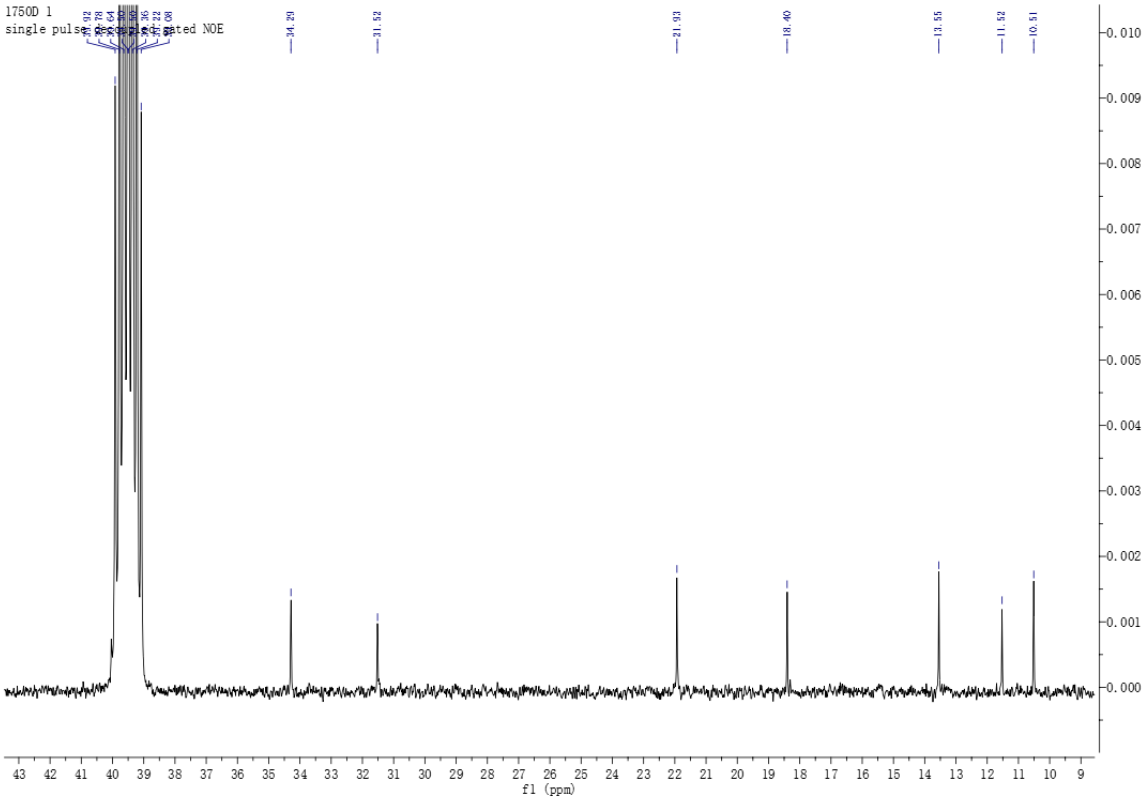

Figure S140. HMQC spectrum of penipyridinone A (**7**, in DMSO-d_6)

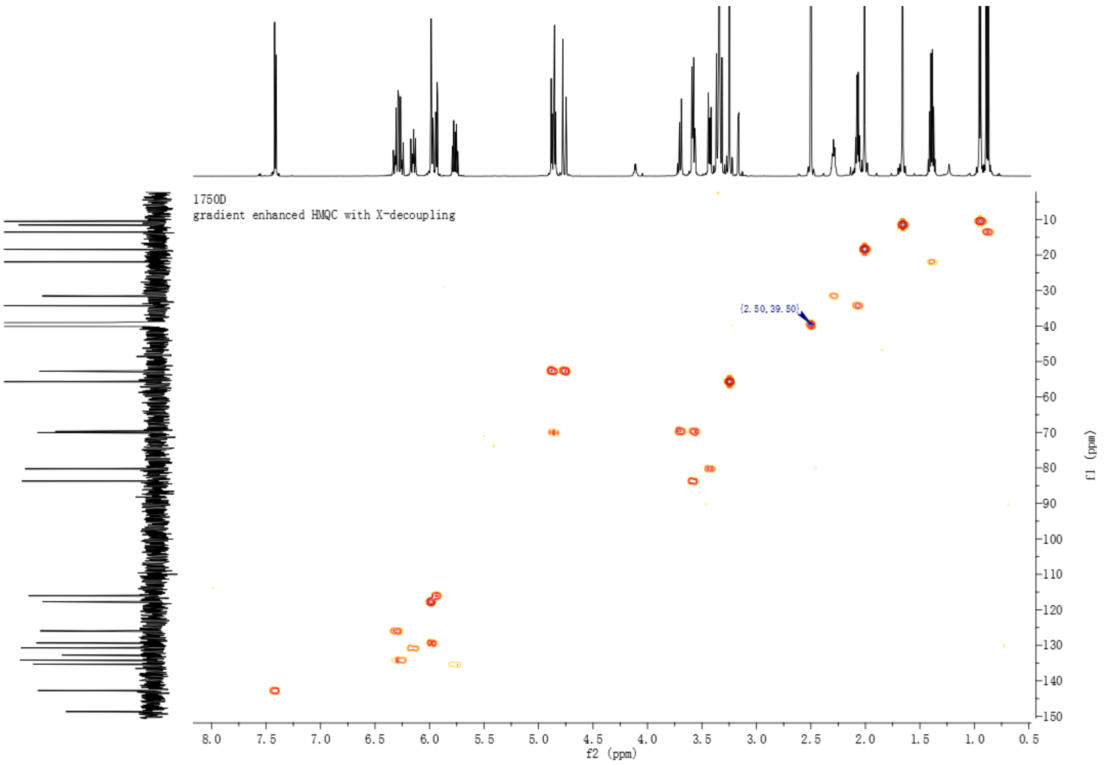

Figure S141. HMQC spectrum of penipyridinone A (**7**, in DMSO-d_6)

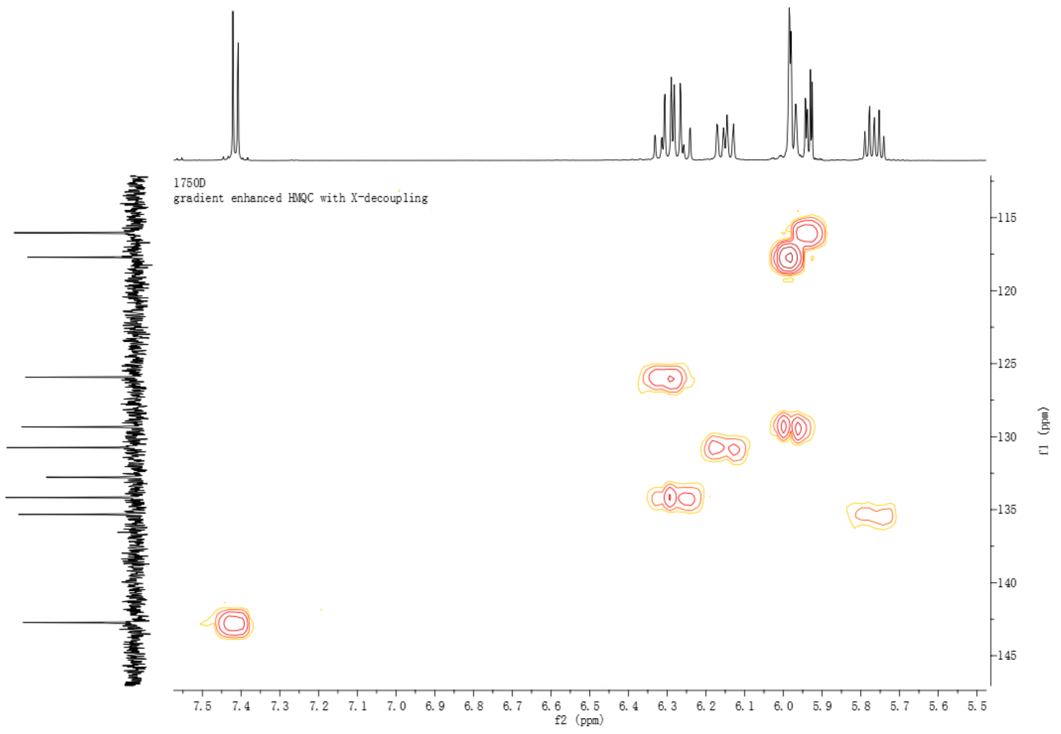

Figure S142. HMQC spectrum of penipyridinone A (**7**, in DMSO-d_6)

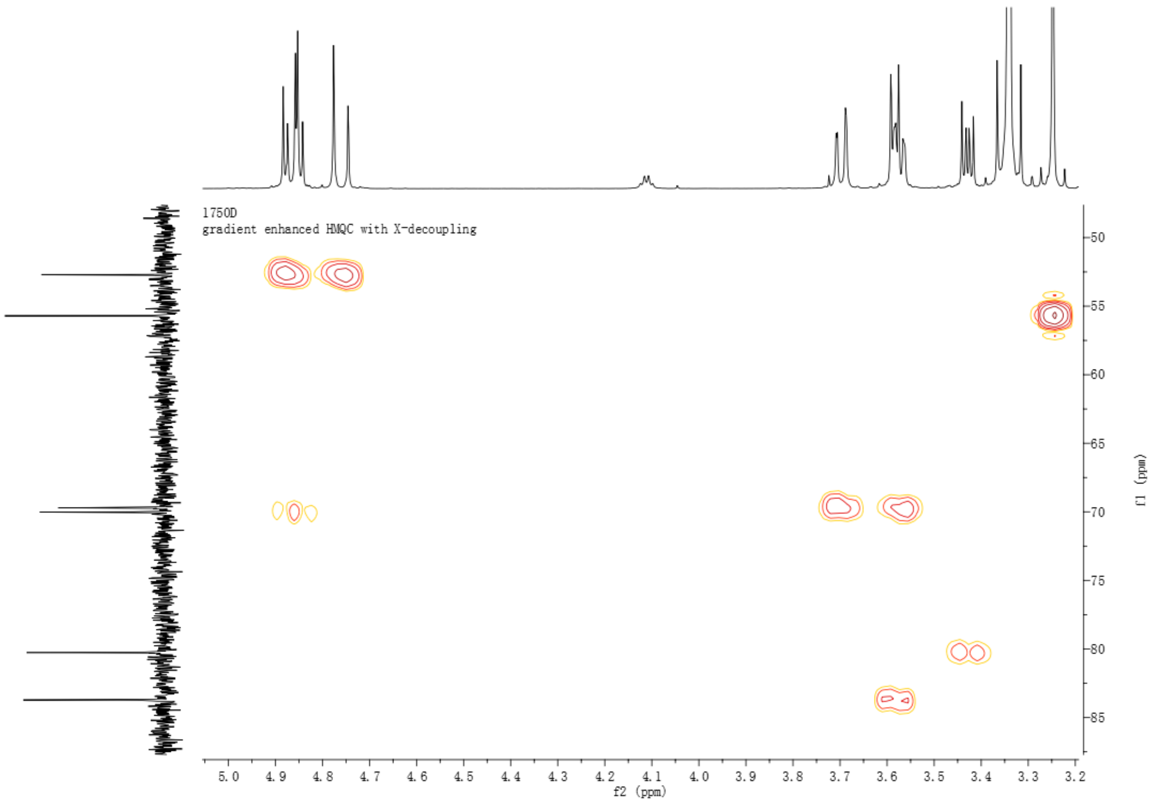

Figure S143. HMQC spectrum of penipyridinone A (**7**, in DMSO-d_6)

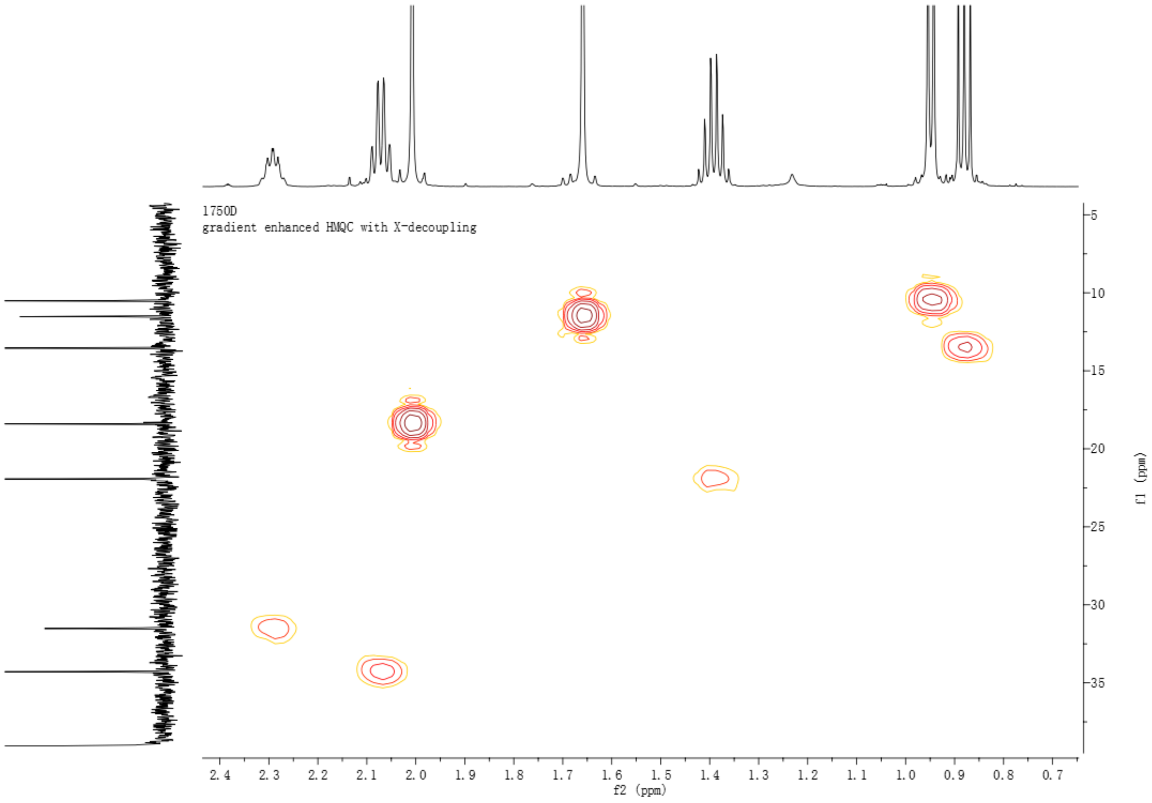

Figure S144. COSY spectrum of penipyridinone A (**7**, in DMSO-d_6)

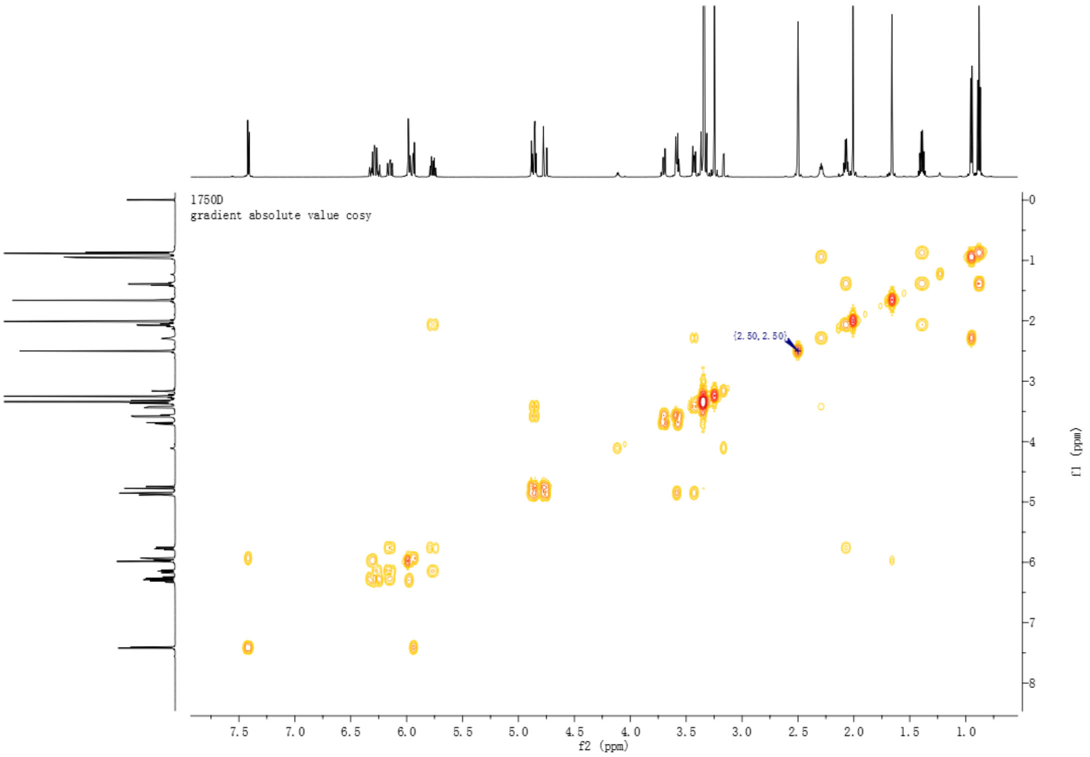

Figure S145. COSY spectrum of penipyridinone A (**7**, in DMSO-d_6)

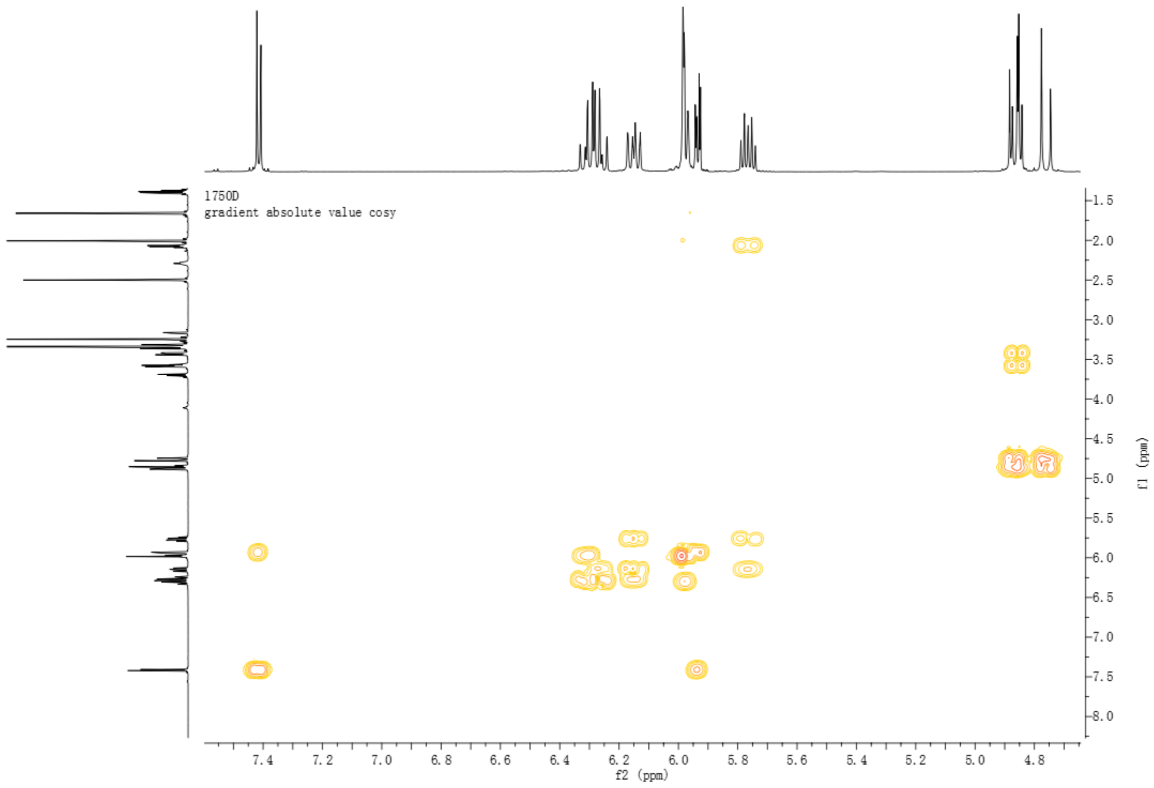

Figure S146. COSY spectrum of penipyridinone A (**7**, in DMSO-d_6)

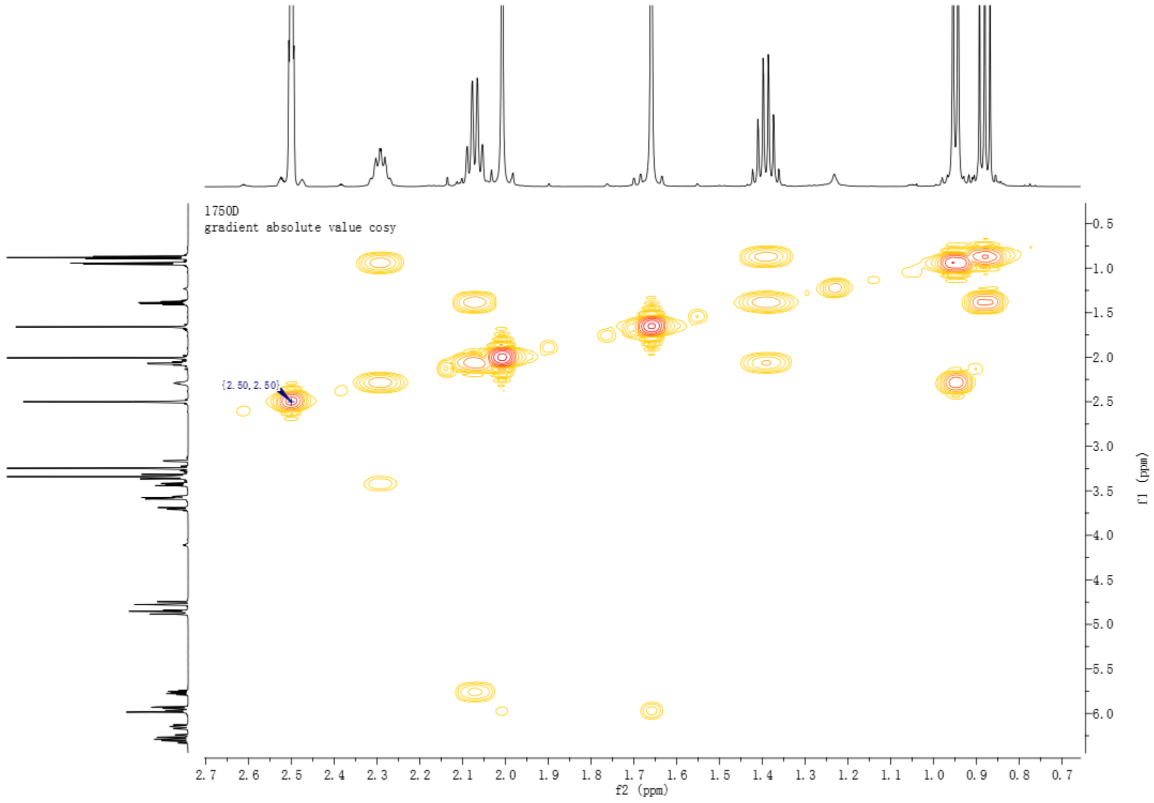

Figure S147. HMBC spectrum of penipyridinone A (**7**, in DMSO-d_6)

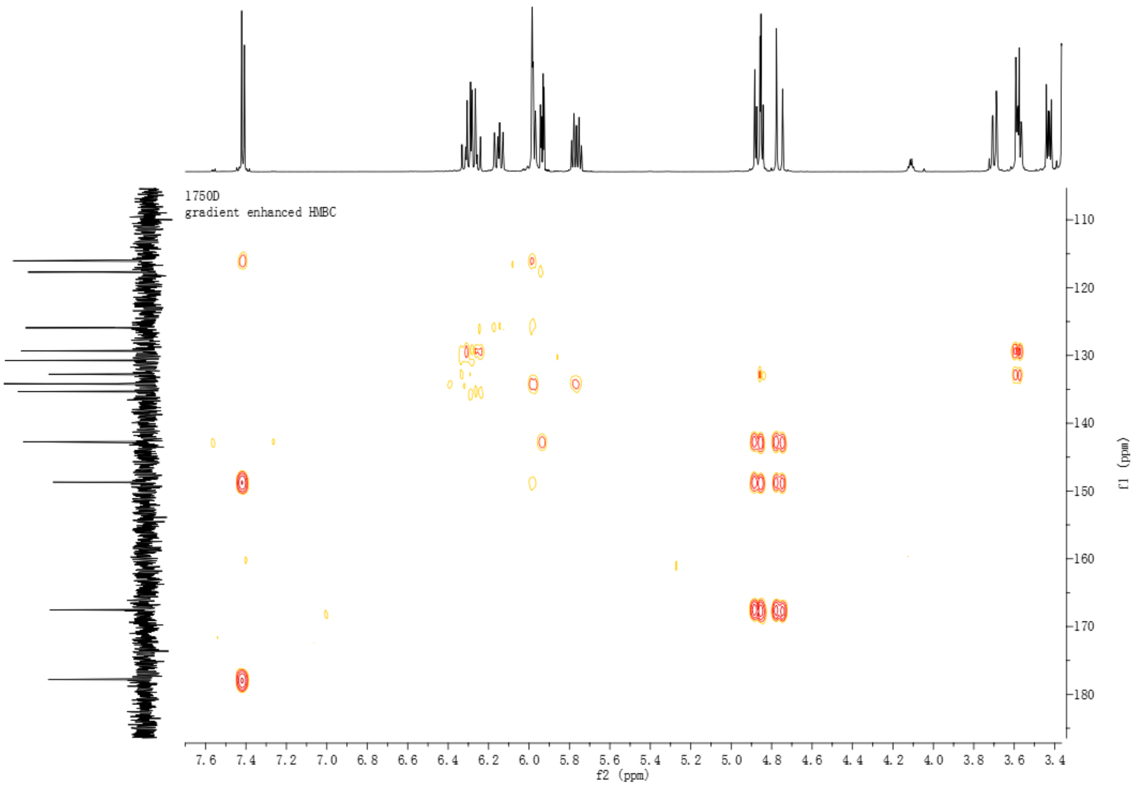

Figure S148. HMBC spectrum of penipyridinone A (**7**, in DMSO-d_6)

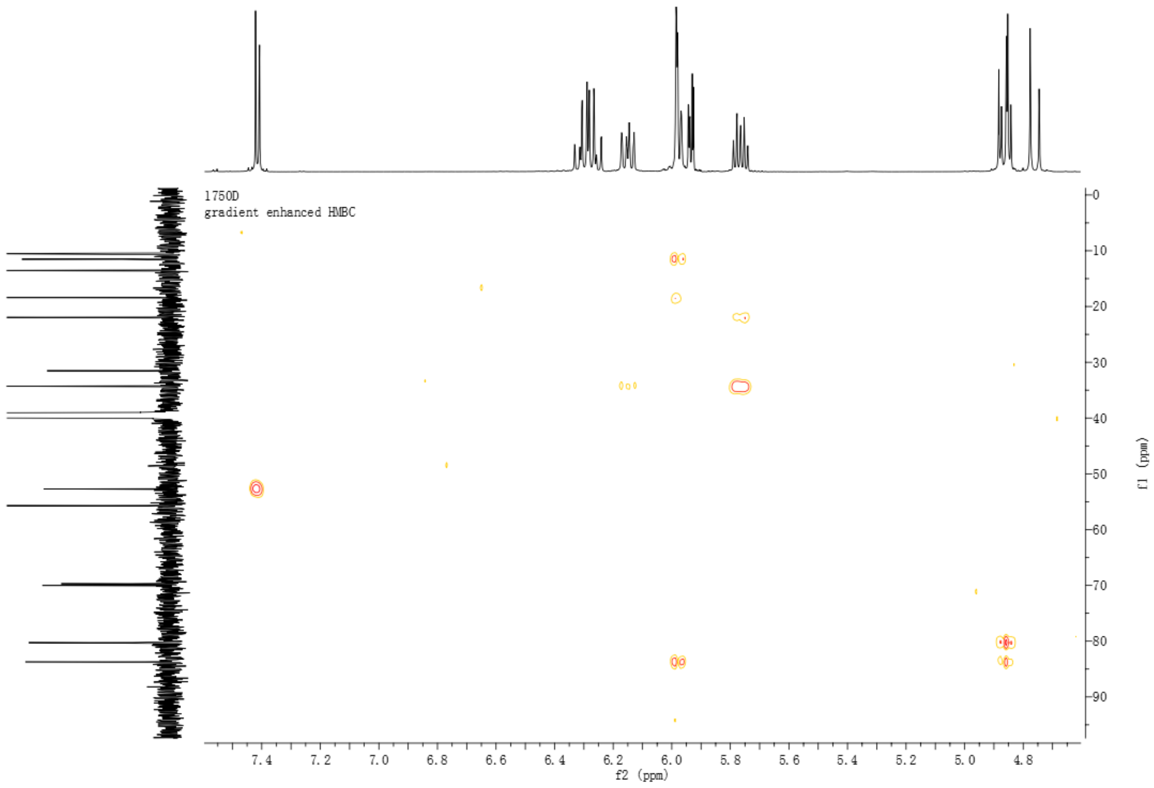

Figure S149. HMBC spectrum of penipyridinone A (**7**, in DMSO-d_6)

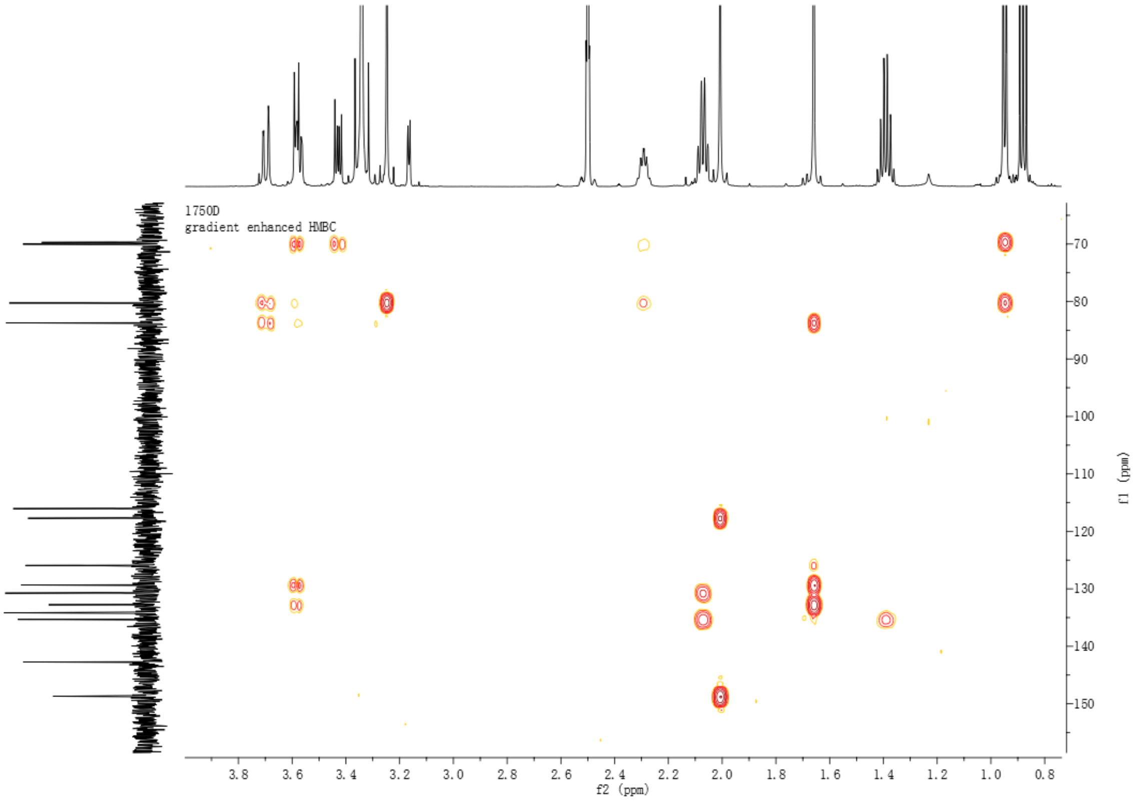

Figure S150. HMBC spectrum of penipyridinone A (**7**, in DMSO-d_6)

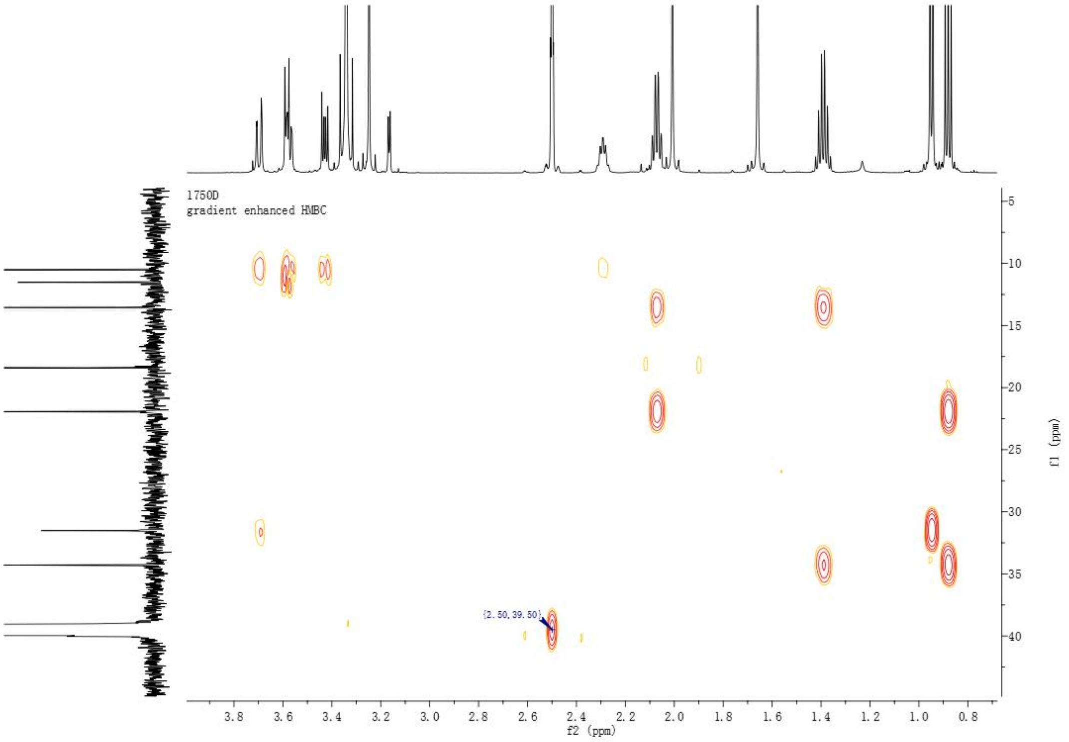

Figure S151. NOESY spectrum of penipyridinone A (**7**, in DMSO-*d*6)

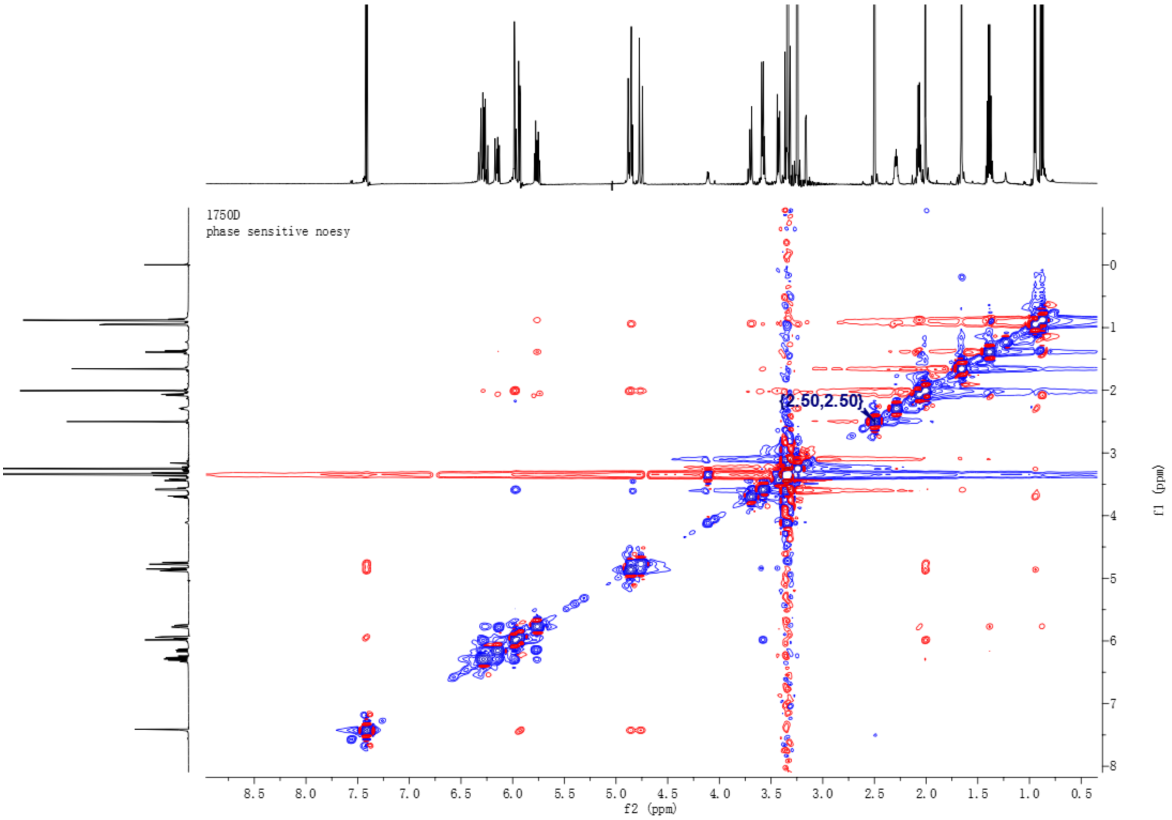

Figure S152. HRESIMS spectrum of penipyridinone A (**7**)

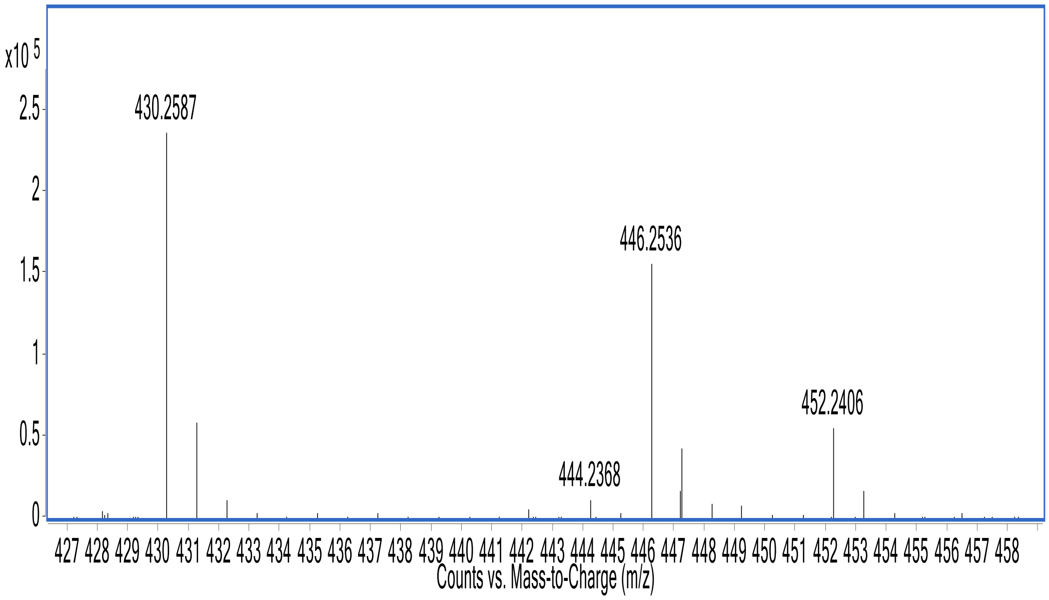

[M+H]$^+$: 430.2587 (calcd for $C_{25}H_{36}NO_5$, 430.2593), [M+Na]$^+$: 452.2406 (calcd for $C_{25}H_{35}NNaO_5$, 452.2413).
